# Supplementary material for: Effectiveness of influenza vaccines in adults with chronic liver disease: a systematic review and meta-analysis
Source: BMJ Open. 2019 Sep 6;9(9):e031070. doi: 10.1136/bmjopen-2019-031070 (PMC6731888; doi:10.1136/bmjopen-2019-031070)
Supplement: Supplementary data [file bmjopen-2019-031070supp001.pdf]

SUPPLEMENTARY MATERIALS

Table 1. Differences between protocol and review
Table 2. Excluded test-negative case-control studies with hospitalised patients that included/are likely to have included liver patients
Table 3. Full MEDLINE (Ovid) search
Table 4. Medical subject headings used in Embase (Ovid), Pubmed and Cochrane Central Register of Controlled Trials
Table 5. Studies excluded due to uncertainty over whether the study included liver disease patients and the number of liver disease patients included
Table 6. Risk of bias in individual studies
Table 7. Risk of selective outcome reporting in the included studies
Table 8. GRADE evidence profile
Table 9. HI antibody responses in liver disease patients before and after vaccination
Figure 1. Seroprotection rate in liver disease patients before influenza vaccination
Figure 2. Sensitivity analysis: Mean difference in log HI antibody GMTs before and after vaccination in cirrhotic patients only
Figure 3. Sensitivity analysis: Seroconversion rate after vaccination in cirrhotic patients only
Figure 4. Sensitivity analysis: Seroprotection rate after vaccination in cirrhotic patients only
Figure 5. Sensitivity analysis: Seroprotection rate before vaccination in cirrhotic patients only
Figure 6. Additional analysis: Mean difference between HI antibody GMT after influenza vaccination in liver disease patients compared to healthy individuals
Figure 7. Additional analysis: Mean difference between geometric mean HI antibody titres before influenza vaccination: liver disease patients compared to healthy individuals
Figure 8. Additional analysis: Mean difference between geometric mean HI antibody titre ratio after influenza vaccination in liver disease patients compared to healthy individuals
Figure 9. Additional analysis: Probability of seroconversion after influenza vaccination in liver disease patients compared to healthy individuals
Figure 10. Additional analysis: Probability of seroprotection after influenza vaccination in liver disease patients compared to healthy individuals
Figure 11. Additional analysis: Difference in seroprotection rate before vaccination in liver disease patients compared to healthy individuals
Table 10. Risk of all-cause mortality in the general population of the included studies where liver patients formed a subgroup
References

Härmälä S, *et al. BMJ Open* 2019; 9:e031070. doi: 10.1136/bmjopen-2019-031070

Table 1. Differences between protocol and review

Review section	Protocol	Review
**Search strategy**	1. The review protocol was specified in advance as part of a wider review plan that also includes a study of the effectiveness of pneumococcal vaccine. The search strategy in this wider plan includes search terms for both influenza vaccine and pneumococcal vaccine.<br><br>2. We planned to search the Cochrane Central Register of Controlled Trials (CENTRAL), MEDLINE, EMBASE, PubMed, The Cochrane Hepato-Biliary Group Specialised Register and The Cochrane Acute Respiratory Infections Group Register of Trials.<br><br>3. Our planned search terms included filters for adults and study type.<br><br>4. We planned to complement the electronic searches by manually searching the reference lists of included studies for additional references.	1. To keep the message of the manuscript clear and concise, this manuscript focuses on influenza vaccine. The search and the results reported here include only those using search terms for influenza vaccine and not for pneumococcal vaccine.<br><br>2. MEDLINE, EMBASE and PubMed were searched as planned but instead of CENTRAL, The Cochrane Hepato-Biliary Group Specialised Register and The Cochrane Acute Respiratory Infections Group Register of Trials, only CENTRAL was searched to maintain the specificity of the search. In the preliminary searches, the number of records found in CENTRAL and the other wide focus databases was large and loosening the search terms for the two specialist sub-collections within CENTRAL was not deemed necessary (the records included in these sub-collections are also in included in CENTRAL and using the same search terms in CENTRAL and the two specialist sub-databases would result in duplicate records only).<br><br>3. In the final search, we decided to additionally filter for publication type to increase the specificity of the search.<br><br>4. In the final search, we further complemented the search by manually searching also through the recent WHO, ECDC and CDC influenza vaccine guidelines for additional references.
**Risk of bias assessment and quality of evidence**	1. We planned to use Cochrane Collaboration's tool to assess risk of bias in all studies.<br><br>2. We planned to consider age, sex, severity and aetiology of liver disease as the most important confounders in the assessment of observational studies.	1. In the review, we used Cochrane Collaboration's tool to assess risk of bias in randomized controlled trials and followed their recommendation to use the Newcastle-Ottawa scale to assess risk of bias in observational studies.<br><br>2. In the review, based on the recommendations from the peer-review of the protocol, we additionally considered as potential confounders: chronic co-morbidities and previous seasonal influenza vaccination during the same season as additional confounders in the assessment of studies that investigated the clinical effects of a pandemic (monovalent) vaccine. In assessing the quality of evidence, we considered the lack of clear effect in the influenza season as a sign of imprecision of the effect estimate of clinical outcomes.
**Data analysis**	1. We did not plan to report serological outcome measures categorised by virus subtypes.	1. In the review, we reported the serological outcome measures categorised by virus subtype as there may be differences in antibody response between them.

Table 2: Excluded test-negative case-control studies with hospitalised patients that included/are likely to have included liver patients

Study	Location	Cases	Controls	Liver patients
Castilla et al (2011)[1]	Spain	Patients with medically-attended influenza-like illness (MA-ILI), negative for influenza in laboratory tests.	Patients with medically-attended influenza-like illness (MA-ILI), positive for influenza in laboratory tests.	Study included hospitalised patients (34 cases, 280 controls. Eligibility criteria included patients with cirrhosis but number of patients with cirrhosis not reported.
Cheng et al (2013)[2]	Australia	Patients hospitalised for ILI, negative for influenza in laboratory tests.	Patients hospitalised for ILI, positive for influenza in laboratory tests.	Study included 38 cases and 71 controls with cirrhosis. Number of vaccinated/unvaccinated cirrhosis patients not reported.
Cheng et al (2014)[3]	Australia	Patients hospitalised for ILI, negative for influenza in laboratory tests.	Patients hospitalised for ILI, positive for influenza in laboratory tests.	Study included 38 cases with cirrhosis, number of controls with cirrhosis not reported. Number of vaccinated/unvaccinated cirrhosis patients not reported.
Choi et al (2013)[4]	South Korea	Patients with medically-attended influenza-like illness (MA-ILI), negative for influenza in laboratory tests.	Patients with medically-attended influenza-like illness (MA-ILI), positive for influenza in laboratory tests.	Study included 19 cases and 25 controls with liver disease. Number of hospitalised liver patients not reported. Number of vaccinated/unvaccinated liver patients not reported.
Choi et al (2015)[5]	South Korea	Patients with medically-attended influenza-like illness (MA-ILI), negative for influenza in laboratory tests.	Patients with medically-attended influenza-like illness (MA-ILI), positive for influenza in laboratory tests.	Study included 40 cases and 39 controls with liver disease. Number of hospitalised liver patients not reported. Number of vaccinated/unvaccinated liver patients not reported.

Härmälä S, *et al. BMJ Open* 2019; 9:e031070. doi: 10.1136/bmjopen-2019-031070

Table 3. Full MEDLINE (Ovid) search

Search concept	Search terms
Influenza vaccine	1. exp Influenza Vaccines/
	2. exp Influenza, Human/pc [Prevention & Control]
	3. ((anti?influenza or influenza or seasonal or anti?flu or flu) adj5 (vaccin* or immuni*)).mp.
	4. ((TIV or QIV or trivalent or quadrivalent or 3?valent or 4?valent) adj5 (vaccin* or immuni*)).mp.
	5. 1 or 2 or 3 or 4
Liver disease	6. exp Liver Diseases/
	7. ((liver or hepat*) adj3 disease*).mp.
	8. ("chronic liver" or "chronic hepat*").mp.
	9. cirrho*.mp.
	10. 6 or 7 or 8 or 9
Adult participants	11. exp Adult/
	12. adult.mp.
	13. (middle?aged or aged).sh.
	14. age*.tw.
	15. 11 or 12 or 13 or 14
Randomised controlled trials	16. randomized controlled trial.pt.
	17. randomi*.ab,ti.
	18. randomly.ab,ti.
	19. controlled clinical trial.pt.
	20. trial.ab,ti.
	21. groups.ab,ti.
	22. drug therapy.fs.
	23. placebo.ab,ti.
	24. 16 or 17 or 18 or 19 or 20 or 21 or 22 or 23
	25. (review or "practice guideline" or "patient education handout" or guideline or editorial or "case reports").pt.
	26. 25 not (25 and (16 and 19))
	27. Animals/
	28. Humans/
	29. 27 not (27 and 28)
	30. 24 not (26 or 29)
Case-control and cohort studies	31. Epidemiologic Studies/
	32. exp Case control studies/
	33. exp Cohort studies/
	34. Longitudinal studies/

	35. Follow up studies/
	36. Prospective studies/
	37. Retrospective studies/
	38. Control groups/
	39. Matched-Pair Analysis/
	40. (Case* adj5 control*).ti,ab,kw.
	41. (Case* adj5 comparison*).ti,ab,kw.
	42. Control group*.ti,ab,kw.
	43. (Cohort adj (study or studies)).ti,ab.
	44. Cohort anal*.ti,ab.
	45. (Follow up adj (study or studies)).ti,ab.
	46. (Observational adj (study or studies)).ti,ab.
	47. Longitudinal.ti,ab.
	48. Retrospective.ti,ab.
	49. Prospective.ti,ab.
	50. 31 or 32 or 33 or 34 or 35 or 36 or 37 or 38 or 39 or 40 or 41 or 42 or 43 or 44 or 45 or 46 or 47 or 48 or 49
	51. (review or "practice guideline" or "patient education handout" or guideline or editorial or "case reports").pt.
	52. 51 not (51 and (31 or 32 or 33 or 34 or 35 or 36 or 37 or 38 or 39))
	53. Animals/
	54. Humans/
	55. 53 not (53 and 54)
	56. 50 not (52 or 55)
	57. 30 or 56
	58. 5 and 10 and 15 and 57
Results: Liver disease-specific search	59. limit 58 to medline
	60. 5 and 15 and 30
Results: Influenza vaccine studies search	61. limit 60 to medline

Table 4. Medical subject headings used in Embase (Ovid), Pubmed and Cochrane Central Register of Controlled Trials

Database	Medical subject headings				
	**Influenza**	**Liver disease**	**Adults**	**Study type**	**Animal/Human study**
Embase (Ovid)	exp influenza vaccine/<br>exp influenza/pc [Prevention]	exp liver disease/<br>exp liver cirrhosis/	exp adult/	randomized controlled trial/<br>controlled clinical trial/<br>epidemiology/<br>exp case control study/<br>exp cohort analysis/<br>longitudinal study/<br>follow up/<br>prospective study/<br>retrospective study/<br>control group/	exp animal/<br>exp animal cell/<br>exp animal disease/<br>exp animal experiment/<br>exp animal model/<br>exp animal tissue/<br>human/
Pubmed	Influenza Vaccines[MH]<br>"influenza human/prevention and control"[MH]	liver disease[MH]	"adult"[MH]<br>"middle aged"[MH:noexp]<br>"aged"[MH:noexp]	"Epidemiologic Studies"[MH:noexp]<br>"Case-Control Studies"[MH]<br>"retrospective studies"[MH:noexp] "Control Groups"[MH:noexp]<br>"matched-pair analysis"[MH:noexp]<br>"longitudinal studies"[MH:noexp]<br>"follow-up studies"[MH:noexp]<br>"prospective studies"[MH:noexp]<br>"cohort studies"[MH]	"animals"[MH]<br>"humans"[MH]
Cochrane Central Register of Controlled Trials	[mh "Influenza Vaccines"]<br>[mh "Influenza, human"/PC]	[mh "Liver Diseases"]	[mh Adult]	Not relevant	Not relevant

Table 5. Studies excluded due to uncertainty over whether the study included liver disease patients and the number of liver disease patients included

Study	Location	Study design	Review outcomes	Reason for uncertainty and notes
Castilla et al (2012)[6]	Spain	Cohort study	Influenza illness/ILI-related hospitalisations	The inclusion criteria included patients with liver disease. to confirm the actual study population also included liver disease patients we contacted but could not reach the corresponding author.
Castilla et al (2013)[7]	Spain	Cohort study	Influenza illness/ILI-related hospitalisations	The inclusion criteria included patients with liver disease. to confirm the actual study population also included liver disease patients we contacted but could not reach the corresponding author.
DiazGranados et al (2013)[8]	United States	Randomised, controlled trial	Serological response	Study population excluded patients with known or suspected hepatitis B or C infection and patients who abused alcohol but included patients with hepatobiliary disorders: 522 in high dose vaccination group and 257 in normal dose vaccination group. We attempted to contact but could not reach the corresponding author.
Emborg et al (2012)[9]	Denmark	Cohort study	Influenza illness/ILI-related hospitalisations	The inclusion criteria included patients with liver disease. To confirm the actual study population also included liver disease patients we contacted but could not reach the corresponding author.
Mangtani et al (2004)[10]	United Kingdom	Cohort study	Acute respiratory illness-related hospitalization<br><br>Acute respiratory illness-related mortality	The inclusion criteria included patients with liver disease as part of the risk group for influenza complications. We contacted the corresponding author to confirm the actual study population also included liver disease patients. The patient data for the study had only been provided on the level of at-risk individuals not on the level of a single disease and so the author had no access to this information.
Olafsdottir et al (2018)[11]	Iceland	Cohort study	Serological response	The study population was the general population, however, the study data collected included a record of primary biliary cirrhosis. We contacted but could not reach the corresponding author to confirm the actual study population also included liver disease patients.
Simpson et al (2013)[12]	Scotland, United Kingdom	Cohort study	All-cause hospitalisation<br><br>All-cause mortality<br><br>Acute respiratory illness-related hospitalisation (pneumonia & influenza)<br><br>Acute respiratory illness-related mortality (pneumonia & influenza)	Number of patients was not reported. Liver disease patients contributed 3727 person-seasons vaccinated/5377 person-seasons unvaccinated (over 9 seasons) to the study. The study, however, also included under 18 year-olds and ≤14 year-olds contributed 4988 person-seasons vaccinated and 281550 person-seasons unvaccinated to the study. Authors were contacted to confirm the number of patients and to obtain subgroup data, however, they were unable to provide this information. It is likely liver disease was more common among over ≥18 year-olds and so the study potentially included over 400 vaccinated and over 500 unvaccinated adult liver disease patients, however, this could not be confirmed.
Wang et al (2004)[13]	Taiwan	Cohort study	All-cause hospitalisation<br><br>Acute respiratory illness-related hospitalisation (pneumonia & influenza)<br><br>Liver disease complication-related	The inclusion criteria included patients with liver disease as part of the risk group for influenza complications. It seems that the patient data for the study had only been provided on the level of at-risk individuals not on the level of a single diseases and so the authors had no access to this information. We attempted to contact but could not reach the corresponding author to confirm.

Härmälä S, *et al. BMJ Open* 2019; 9:e031070. doi: 10.1136/bmjopen-2019-031070

			hospitalisation (chronic liver disease and cirrhosis)	
Wang et al (2007)[14]	Taiwan	Cohort study	All-cause mortality<br><br>Acute-respiratory illness-related mortality (pneumonia)<br><br>Liver disease complication-related mortality (chronic liver disease)	The inclusion criteria included patients with liver disease as part of the risk group for influenza complications. It seems that the patient data for the study had only been provided on the level of at-risk individuals not on the level of a single diseases and so the authors had no access to this information. We contacted but could not reach the corresponding author to confirm.
Zivich et al (2017)[15]	United States	Cohort study	All-cause mortality	The study included at least 45 vaccinated and 41 unvaccinated liver disease patients. The study, however, also included under 18 year-old patients (121 were ≤4 year-old and 144 were ≤24 year-old. Authors were contacted to confirm the number of patients and to obtain subgroup data, however, they no longer have access to the study data and were unable to provide this information. It is likely liver disease was more common among over ≥18 year-olds and so the study potentially included over 40 adult liver disease patients in each group. This, however, could not be confirmed.

Härmälä S, *et al. BMJ Open* 2019; 9:e031070. doi: 10.1136/bmjopen-2019-031070

Table 6. Risk of bias in the included studies

A. Risk of bias in observational studies of serological outcomes

Study	**Selection**[a]<br>*** = low risk<br>** = moderate risk<br>- and * = high risk	**Outcome**<br>*** = low risk<br>** = moderate risk<br>- and * = high risk
Cheong et al (2006)	*** Low	* High[b,c]
Duchini et al (2001)	*** Low	** Moderate[c]
Hernández-Guerra et al (2012)	*** Low	*** Low
Ohfuji et al (2013)	*** Low	*** Low
Sayyad et al (2012)	** Moderate[d]	** Moderate[b]
Soesman et al (2000)	** Moderate[e]	** Moderate[c]

[a] Selection was assessed considering the selection of the liver disease group only.
[b] High drop-out rate.
[c] No description of independent blind assessment of outcome.
[d] Source/selection of liver patient groups not clear.
[e] Pre-vaccination influenza infection status not clear and pre-vaccination seroprotection rates high.

B. Risk of bias in observational studies of clinical outcomes

Study	**Selection**<br>**** = low risk<br>** and *** = moderate risk<br>- and * = high risk	**Comparability**[a]<br>**** and ***** = low risk<br>** and *** = moderate risk<br>- and * = high risk	**Outcome**<br>*** = low risk<br>** = moderate risk<br>- and * = high risk
Campitelli et al (2010)	*** Moderate[b]	Unclear[c]	*** Low
Castilla et al (2105)	**** Low	Unclear[c]	*** Low
Ohfuji et al (2014)	*** Moderate[b]	**** Moderate[d,e]	*** Low
Su et al (2016)	**** Low	***** Low	*** Low
Vila-Córcoles et al (2007)	**** Low	Unclear[c]	*** Low

[a] Comparability was considered between vaccinated and unvaccinated groups. Confounders considered were: age, sex, liver disease aetiology, liver disease severity, chronic co-morbidities and previous seasonal influenza vaccination in the same season if study vaccine pandemic vaccine.
[b] Self-reported vaccination (although in Campitelli et al 2010, vaccination was ascertained from physician billing claims, these are based on self-reporting).
[c] No information on characteristics of the vaccinated and unvaccinated groups.
[d] Sex not comparable between groups.
[e] Seasonal influenza vaccination in the same season than pandemic vaccine given to some but balance unclear between groups.

C. Risk of bias in randomised controlled studies

Study	Random sequence generation	Allocation concealment	Blinding of participants and personnel	Blinding of outcome assessment	Incomplete outcome data	Selective reporting	Other bias
Song et al (2007)	Low	Low	High[a]	Low	High[b]	Low	Unclear[c]

[a] Patients were not blinded and could not have been blinded when only the intervention group received the vaccination.
[b] High drop-out rate.
[c] Details on study funding and conflicts of interest were not provided.

Table 7. Risk of selective outcome reporting in the included studies

Risk of bias arising from the lack of inclusion of results when a study was excluded from a meta-analysis or not fully reported in a review because the data were unavailable.

Level of reporting: √ = Full reporting of results, × = No reporting of results, o = Partial reporting of results

Classifications and descriptions of risk of bias found in the assessment of missing or complete outcome reporting:

Clear that the outcome was measured and analysed:	Level of reporting	Risk of bias
C = Trial report states that outcome was analysed but insufficient data were presented for the trial to be included in meta-analysis or to be considered to be fully tabulated.	o	Low Risk
Clear that the outcome was measured:		
E = Clear that the outcome was measured. Judgment says outcome likely to have been analysed but not reported because of non-significant results.	×	High Risk
Unclear whether the outcome was measured:		
G = Not mentioned but clinical judgment says likely to have been measured and analysed but not reported on the basis of non-significant results.	×	High Risk
H = Not mentioned but clinical judgment says unlikely to have been measured at all.	×	Low Risk
Clear that the outcome was not measured:		
I = Clear that the outcome was not measured.	×	No Risk

Härmälä S, *et al. BMJ Open* 2019; 9:e031070. doi: 10.1136/bmjopen-2019-031070

A. Risk of selective outcome reporting in studies of serological outcomes

	**Review secondary outcomes**				**Other outcomes reported**
**Study**	**GMT**	**GMTR**	**Seroprotection %**	**Seroconversion %**	
Cheong et al (2006)	√	×<br>E – High risk[a]	o<br>C – Low risk[b]	o<br>C – Low risk[b]	Cellular immune response to vaccine (interferon-γ secretion)
Duchini et al (2001)	√	√	×<br>E – High risk[c]	√	None
Hernández-Guerra et al (2012)	o<br>C – Low risk[d]	√	o<br>C – Low risk[e]	√	Systemic adverse events, acceptance and tolerance of vaccination
Ohfuji et al (2013)	√	√	√	√	Serious adverse events
Sayyad et al (2012)	√	×<br>E – High risk[a]	o<br>C – Low risk[f]	o<br>C – Low risk[f]	None
Soesman et al (2000)	√	×<br>E – High risk[a]	√	√	Serological response (HI antibody) to a non-vaccine strain

[a] Pre- and post-vaccination GMT was measured and it is likely that their ratio was analysed but not reported.
[b] Only percentages were reported so we have assumed the highest possible number of individuals closest to the reported percentage for pre-vaccination measures and lowest possible number of individuals closest to the reported percentage for post-vaccination measures.
[c] Pre- and post-vaccination GMT was measured and seroconversion rate was analysed. It is likely that the seroprotection rate was also analysed but not reported.
[d] Pre-vaccination GMT was measured but not fully reported. It is likely this was low as only one individual had 1:40 antibody level (seroprotective level).
[e] Pre-vaccination seroprotection rate was not fully reported but the rate was low only one individual had a seroprotective level of antibodies. It was not specified in which comparison group so we have assumed it was one individual in each group.
[f] The percentages and numbers of individuals did not add up in the reported results so we have assumed the highest possible number of individuals closest to the reported percentage for pre-vaccination measures and lowest possible number of individuals closest to the reported percentage for post-vaccination measures.

Härmälä S, *et al. BMJ Open* 2019; 9:e031070. doi: 10.1136/bmjopen-2019-031070

B. Risk of selective outcome reporting in studies of clinical outcomes

	**Review primary outcomes**		**Review secondary outcomes**						**Other outcomes reported**
**Study**	**All-cause hospitalisation**	**All-cause mortality**	**Acute respiratory illness-related hospitalisation**	**Influenza illness/ILI-related hospitalisation**	**Liver disease complication related-hospitalisation**	**Acute respiratory illness-related mortality**	**Influenza illness/ILI-related mortality**	**Liver disease complication related-mortality**	
Campitelli et al (2010)	×<br>H – Low risk[a]	√	o<br>C – Low risk[b]	×<br>I – No risk	×<br>I – No risk	×<br>I – No risk	×<br>I – No risk	×<br>I – No risk	None
Castilla et al (2105)	×<br>I – No risk	√	×<br>I – No risk	×<br>I – No risk	×<br>I – No risk	×<br>I – No risk	×<br>I – No risk	×<br>I – No risk	ILI incidence
Ohfuji et al (2014)	√	×<br>I – No risk	×<br>I – No risk	×<br>I – No risk	×<br>I – No risk	×<br>I – No risk	×<br>I – No risk	×<br>I – No risk	None
Song et al (2007)	×<br>I – No risk	×<br>G – High risk[c]	×<br>I – No risk	×<br>I – No risk	×<br>I – No risk	×<br>I – No risk	√	×<br>I – No risk	ILI incidence, clinical manifestations of ILI, liver disease complications secondary to influenza
Su et al (2016)	√	√	×<br>I – No risk	×<br>I – No risk	√	×<br>I – No risk	×<br>I – No risk	×<br>I – No risk	Pneumonia & Influenza incidence, heart disease related hospitalisation, respiratory failure, intensive care unit admission
Vila-Córcoles et al (2007)	×<br>I – No risk	√	×<br>I – No risk	×<br>I – No risk	×<br>I – No risk	×<br>I – No risk	×<br>I – No risk	×<br>I – No risk	ILI incidence

[a] The main interest in the study was all-cause mortality and cause-specific hospitalisation outcome was used to understand the whether the mortality may have been linked to influenza. Accessing the data required an approved study protocol and it is unlikely that data to study all-cause hospitalisation was available when not included in the protocol.
[b] Study authors were unable to provide exact event numbers for this outcome as their institution's privacy policy does not allow providing data in case of five or fewer events.
[c] Authors did measure influenza-related mortality in this trial and it is likely that they also measured all-cause mortality. Authors were contacted but we were unable to obtain a response.

Härmälä S, *et al. BMJ Open* 2019; 9:e031070. doi: 10.1136/bmjopen-2019-031070

Table 8. GRADE evidence profile

Effectiveness of influenza vaccination in preventing all-cause hospitalisation and all-cause mortality in adults with chronic liver disease

Number of studies (design)	Limitations	Inconsistency	Indirectness	Imprecision	Publication bias	No vaccine	Vaccine	Relative risk/ Rate ratio	Control risk/ Control rate	Risk difference	Quality
All-cause hospitalisation, influenza season											
1 (Cohort study)	Serious limitations[a]	Not judged (only 1 study)	No serious indirectness	Serious imprecision[b]	Suspected[c]	22/276	6/132	0·57 (0·24, 1.37)	80 per 1000	-0·03 (-0·08, 0·01)	Very low
All-cause hospitalisation, all year											
1 (Cohort study)	No serious limitations	Not judged (only 1 study)	No serious indirectness	No serious imprecision	Suspected[c]	748/3646	661/4434	0·73 (0·66, 0·80)	205 per 1000	-0·06 (-0·07-0·04)	Very low
All-cause mortality, influenza season											
3 (Cohort study)	Serious limitations[d]	Serious inconsistency[e]	No serious indirectness	Serious imprecision[f]	Suspected[c]	42/2239 person-seasons	66/3736 person-seasons	0·80 (0·43, 1·50)	17 per 1000 person-seasons	-0·22 (-0·85, 0·41)	Very low
All-cause mortality, all year											
2 (Cohort study)	Serious limitations[g]	No serious inconsistency	No serious indirectness	Serious imprecision[e,f]	Suspected[c]	318/3687 person-years	105/4718 person-years	0·41 (0·11, 1·52)	85 per 1000 person-years	-0·89 (-2·19, 0·42)	Very low

[a] Moderate risk of bias in selection and comparability of groups.
[b] Imprecision of the estimate with large 95% CIs.
[c] Due to study language restrictions in the review and exclusion of studies due to uncertainty of eligibility.
[d] Moderate risk of bias in selection in one study and unclear bias in comparability of groups in all studies.
[e] Study effect estimates varied largely.
[f] Control risks varied largely between studies.
[g] Unclear bias in comparability of groups in one study and uncertain timing of the vaccine effect due to Kaplan-Meier plot showing increasing difference in mortality rates outside the influenza season.

Table 9. HI antibody responses in liver disease patients before and after vaccination

Influenza A (H1N1)						
**Study**	**Pre-vaccination GMT (95% CI)**	**Post-vaccination GMT (95% CI)**	**GMTR**	**Seroconversion rate (%)**	**Pre-vaccination seroprotection rate (%)**	**Post-vaccination seroprotection rate (%)**
Cheong et al (2006)[16]	15·6 (SE ±1·8)	114·7 (SE ±4·1)	Not reported	40/50 (80%)	5/50 (10%)	39/50 (79%)
Duchini et al (2001)[17]	20·0 (10.0, 40.0)	61·0 (30·0, 125·0)	3·0 (1·7, 5·4)	6/14 (43%)	Not reported	Not reported
Hernández-Guerra et al (2012)[18]	Not reported	229·0 (55·0, 957·0)	Treated: 43·0 (10·0, 180·0)<br>Untreated: 32·0 (7·0, 137·0)	24/25 (96%)	1/25 (4%)	24/25 (96%)
Ohfuji et al (2013)[19]	8·0 (7·0, 9·0)	226·0 (69·0-743·0)	10·3 (7·2, 14·9)	57/59 (97%)	4/79 (5%)	56/79 (71%)
Sayyad et al (2012)[20]	Cirrhotic: 31·2 (SE ±2·0)<br>HBV carrier: 24·1 (SE ±2·4)	Cirrhotic: 170·7 (SE ±1·6)<br>HBV carrier: 177.7 (SE ±1·8)	Not reported	Cirrhotic: 26/28 (93%)<br>HBV carrier: 28/31 (90%)	Cirrhotic: 19/28 (68%)<br>HBV carrier: 16/31 (52%)	Cirrhotic: 28/28 (100%)<br>HBV carrier: 31/31 (100%)
Soesman et al (2000)[21]	60·0 (37·0, 97·0)	795·0 (484·0, 1303·0)	Not reported	23/35 (66%)	26/36 (72%)	35/35 (100%)
Influenza A (H3N2)						
**Study**	**Pre-vaccination GMT (95% CI)**	**Post-vaccination GMT (95% CI)**	**GMTR**	**Seroconversion rate (%)**	**Pre-vaccination seroprotection rate (%)**	**Post-vaccination seroprotection rate (%)**
Cheong et al (2006)[16]	36·8 (SE ±3·2)	75·7 (SE ±2·6)	Not reported	23/50 (46%)	13/50 (26%)	37/50 (74%)
Duchini et al (2001)[17]	87·0 (37·0, 203.0)	216·0 (95·0, 489·0)	2·4 (1·6, 3·8)	3/14 (21%)	Not reported	Not reported
Sayyad et al (2012)[20]	Cirrhotic: 36·6 (SE ±2·4)<br>HBV carrier: 24·5 (SE ±2·3)	Cirrhotic: 121·5 (SE ±1·9)<br>HBV carrier: 111·1 (SE ±2·4)	Not reported	Cirrhotic: 20/28 (71%)<br>HBV carrier: 22/31 (71%)	Cirrhotic: 20/28 (71%)<br>HBV carrier: 16/31 (52%)	Cirrhotic: 28/28 (100%)<br>HBV carrier: 28/31 (90%)
Soesman et al (2000)[21]	34·0 (19·0, 61·0)	331·0 (207·0, 531·0)	Not reported	21/35 (60%)	21/36 (58%)	34/35 (97%)
Influenza B						
**Study**	**Pre-vaccination GMT (95% CI)**	**Post-vaccination GMT (95% CI)**	**GMTR**	**Seroconversion rate (%)**	**Pre-vaccination seroprotection rate (%)**	**Post-vaccination seroprotection rate (%)**
Cheong et al (2006)[16]	17·4 (SE ±1·7)	82·3 (SE ±2·3)	Not reported	43/50 (86%)	5/50 (10%)	44/50 (88%)

Duchini et al (2001)[17]	18·0 (9·0, 35·0)	119·0 (48·0, 292·0)	6·0 (2·6, 19·0)	9/14 (64%)	Not reported	Not reported
Sayyad et al (2012)[20]	Cirrhotic: 27·1 (SE ±2·1)<br>HBV carrier: 14·9 (SE ±2·1)	Cirrhotic: 210·6 (SE ±1·7)<br>HBV carrier: 134·3 (SE ±2·0)	Not reported	Cirrhotic: 28/28 (100%)<br>HBV carrier: 29/31 (94%)	Cirrhotic: 18/28 (64%)<br>HBV carrier: 6/31 (19%)	Cirrhotic: 28/28 (100%)<br>HBV carrier: 31/31 (100%)
Soesman et al (2000)[21]	12·0 (8·0, 19·0)	110·0 (61·0, 199·0)	Not reported	25/35 (71%)	10/36 (28%)	29/35 (83%)

Figure 1. Seroprotection rate in liver disease patients before influenza vaccination

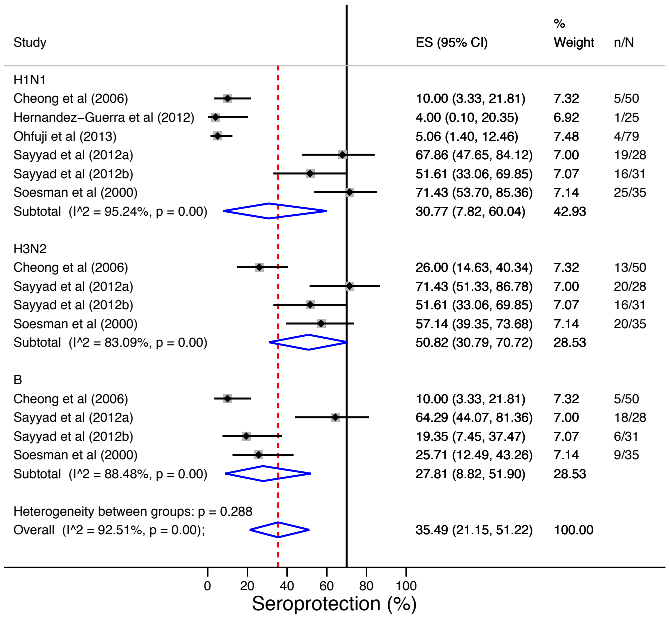

Figure 2. Sensitivity analysis: Mean difference in log HI antibody GMTs before and after vaccination in cirrhotic patients only

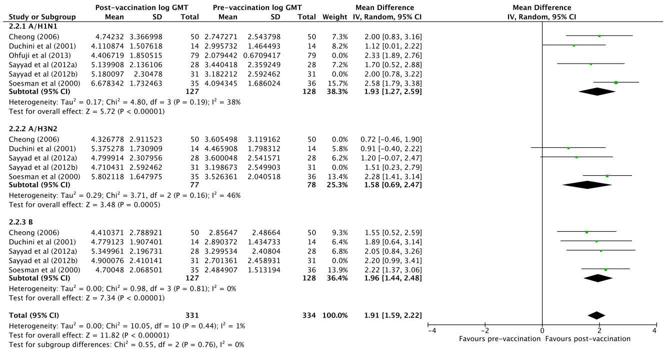

Note: Liver patient results from the studies with non-cirrhotic patients only and studies with a mix of cirrhotic and non-cirrhotic patients are visible but do not affect the outcome of analysis. Only the results from the studies with cirrhotic patients Cheong et al (2006), Duchini et al (2001) Sayyad et al (2012a) and Soesman are pooled in this analysis. Sayyad et al (2012b) includes non-cirrhotic patients only.

Härmälä S, *et al*. *BMJ Open* 2019; 9:e031070. doi: 10.1136/bmjopen-2019-031070

Figure 3. Sensitivity analysis: Seroconversion rate after vaccination in cirrhotic patients only

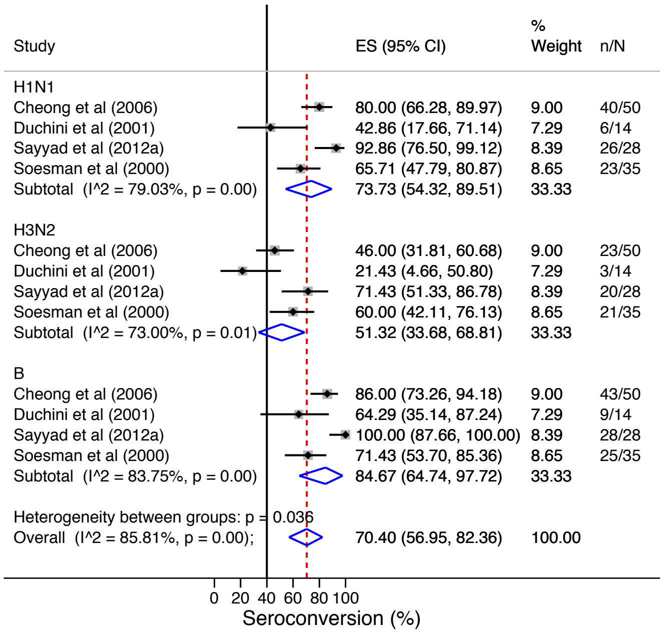

Figure 4. Sensitivity analysis: Seroprotection rate after vaccination in cirrhotic patients only

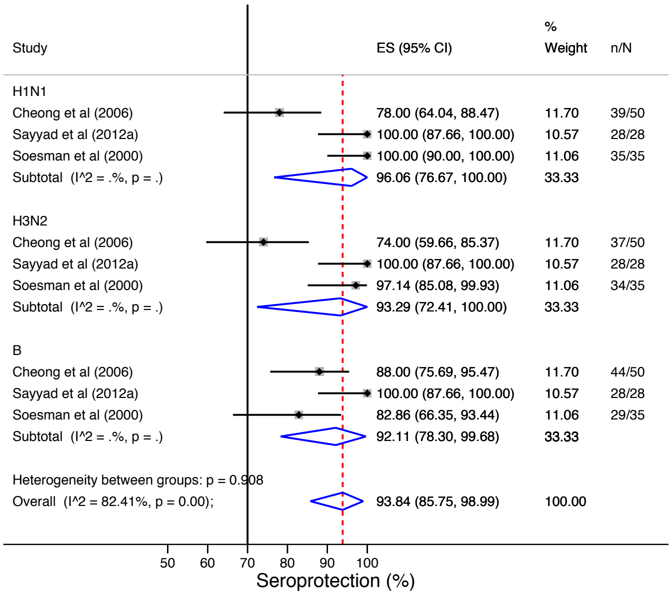

Härmälä S, *et al. BMJ Open* 2019; 9:e031070. doi: 10.1136/bmjopen-2019-031070

Figure 5. Sensitivity analysis: Seroprotection rate before vaccination in cirrhotic patients only

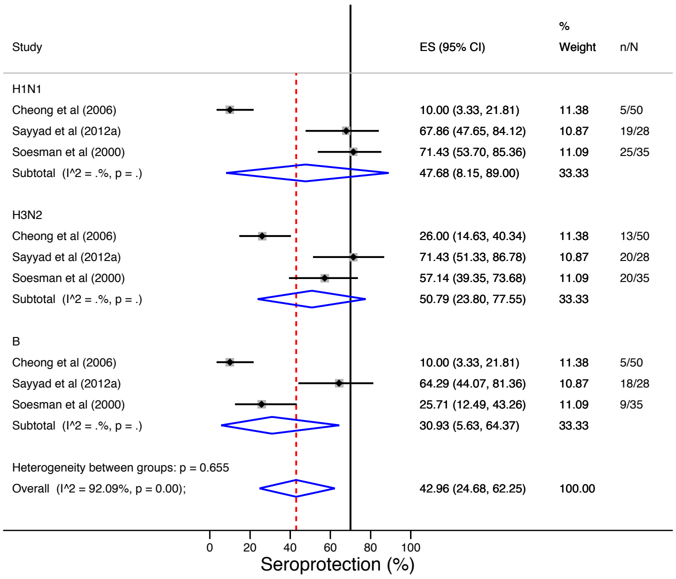

Figure 6. Additional analysis: Mean difference between HI antibody GMT after influenza vaccination in liver disease patients compared to healthy individuals

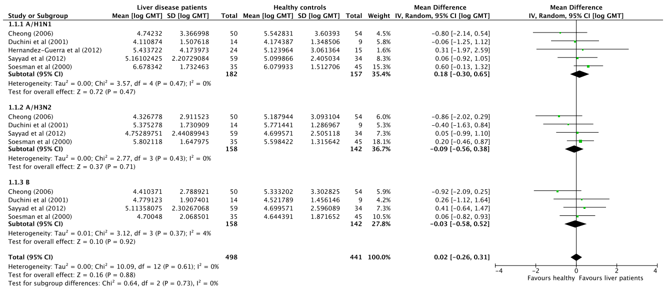

Study or Subgroup	Liver disease patients Mean [log GMT]	SD [log GMT]	Total	Healthy controls Mean [log GMT]	SD [log GMT]	Total	Weight	Mean Difference IV, Random, 95% CI [log GMT]
**1.1.1 A/H1N1**								
Cheong (2006)	4.74232	3.366998	50	5.542831	3.60393	54	4.5%	−0.80 [−2.14, 0.54]
Duchini et al (2001)	4.110874	1.507618	14	4.174387	1.348506	9	5.8%	−0.06 [−1.25, 1.12]
Hernandez-Guerra et al (2012)	5.433722	4.173973	24	5.123964	3.061364	15	1.6%	0.31 [−1.97, 2.59]
Sayyad et al (2012)	5.16102425	2.20729084	59	5.099866	2.405034	34	8.3%	0.06 [−0.92, 1.05]
Soesman et al (2000)	6.678342	1.732463	35	6.079933	1.512706	45	15.3%	0.60 [−0.13, 1.32]
**Subtotal (95% CI)**			**182**			**157**	**35.4%**	**0.18 [−0.30, 0.65]**
Heterogeneity: Tau² = 0.00; Chi² = 3.57, df = 4 (P = 0.47); I² = 0%								
Test for overall effect: Z = 0.72 (P = 0.47)								
**1.1.2 A/H3N2**								
Cheong (2006)	4.326778	2.911523	50	5.187944	3.093104	54	6.0%	−0.86 [−2.02, 0.29]
Duchini et al (2001)	5.375278	1.730909	14	5.771441	1.286967	9	5.3%	−0.40 [−1.63, 0.84]
Sayyad et al (2012)	4.75289751	2.44089943	59	4.699571	2.505118	34	7.3%	0.05 [−0.99, 1.10]
Soesman et al (2000)	5.802118	1.647975	35	5.598422	1.315642	45	18.1%	0.20 [−0.46, 0.87]
**Subtotal (95% CI)**			**158**			**142**	**36.7%**	**−0.09 [−0.56, 0.38]**
Heterogeneity: Tau² = 0.00; Chi² = 2.77, df = 3 (P = 0.43); I² = 0%								
Test for overall effect: Z = 0.37 (P = 0.71)								
**1.1.3 B**								
Cheong (2006)	4.410371	2.788921	50	5.333202	3.302825	54	5.9%	−0.92 [−2.09, 0.25]
Duchini et al (2001)	4.779123	1.907401	14	4.521789	1.456146	9	4.2%	0.26 [−1.12, 1.64]
Sayyad et al (2012)	5.11358075	2.30267068	59	4.699571	2.596089	34	7.3%	0.41 [−0.64, 1.47]
Soesman et al (2000)	4.70048	2.068501	35	4.644391	1.871652	45	10.5%	0.06 [−0.82, 0.93]
**Subtotal (95% CI)**			**158**			**142**	**27.8%**	**−0.03 [−0.58, 0.52]**
Heterogeneity: Tau² = 0.01; Chi² = 3.12, df = 3 (P = 0.37); I² = 4%								
Test for overall effect: Z = 0.10 (P = 0.92)								
**Total (95% CI)**			**498**			**441**	**100.0%**	**0.02 [−0.26, 0.31]**
Heterogeneity: Tau² = 0.00; Chi² = 10.09, df = 12 (P = 0.61); I² = 0%								
Test for overall effect: Z = 0.16 (P = 0.88)								
Test for subgroup differences: Chi² = 0.64, df = 2 (P = 0.73), I² = 0%								

Note: Results from the non-cirrhotic and cirrhotic patient groups in Sayyad et al (2012) have been combined using the formulae recommended in Cochrane Handbook [22].

Figure 7. Additional analysis: Mean difference between geometric mean HI antibody titres before influenza vaccination in liver disease patients compared to healthy individuals

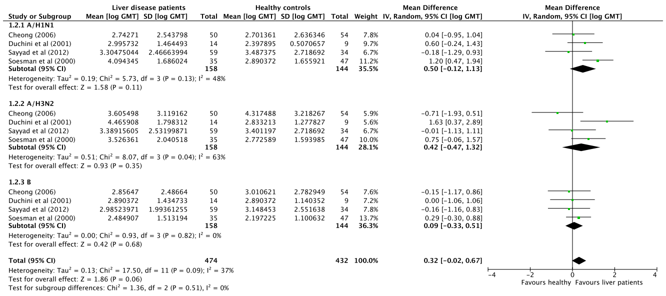

Study or Subgroup	Liver disease patients Mean [log GMT]	SD [log GMT]	Total	Healthy controls Mean [log GMT]	SD [log GMT]	Total	Weight	Mean Difference IV, Random, 95% CI [log GMT]
**1.2.1 A/H1N1**								
Cheong (2006)	2.74271	2.543798	50	2.701361	2.636346	54	7.8%	0.04 [−0.95, 1.04]
Duchini et al (2001)	2.995732	1.464493	14	2.397895	0.5070657	9	9.7%	0.60 [−0.24, 1.43]
Sayyad et al (2012)	3.30475044	2.46663994	59	3.487375	2.718692	34	6.7%	−0.18 [−1.29, 0.93]
Soesman et al (2000)	4.094345	1.686024	35	2.890372	1.655921	47	11.2%	1.20 [0.47, 1.94]
**Subtotal (95% CI)**			**158**			**144**	**35.5%**	**0.50 [−0.12, 1.13]**
Heterogeneity: Tau² = 0.19; Chi² = 5.73, df = 3 (P = 0.13); I² = 48%								
Test for overall effect: Z = 1.58 (P = 0.11)								
**1.2.2 A/H3N2**								
Cheong (2006)	3.605498	3.119162	50	4.317488	3.218267	54	5.9%	−0.71 [−1.93, 0.51]
Duchini et al (2001)	4.465908	1.798312	14	2.833213	1.277827	9	5.6%	1.63 [0.37, 2.89]
Sayyad et al (2012)	3.38915605	2.53199871	59	3.401197	2.718692	34	6.7%	−0.01 [−1.13, 1.11]
Soesman et al (2000)	3.526361	2.040518	35	2.772589	1.593985	47	10.0%	0.75 [−0.06, 1.57]
**Subtotal (95% CI)**			**158**			**144**	**28.1%**	**0.42 [−0.47, 1.32]**
Heterogeneity: Tau² = 0.51; Chi² = 8.07, df = 3 (P = 0.04); I² = 63%								
Test for overall effect: Z = 0.93 (P = 0.35)								
**1.2.3 B**								
Cheong (2006)	2.85647	2.48664	50	3.010621	2.782949	54	7.6%	−0.15 [−1.17, 0.86]
Duchini et al (2001)	2.890372	1.434733	14	2.890372	1.140352	9	7.2%	0.00 [−1.06, 1.06]
Sayyad et al (2012)	2.98523971	1.99361255	59	3.148453	2.551638	34	7.8%	−0.16 [−1.16, 0.83]
Soesman et al (2000)	2.484907	1.513194	35	2.197225	1.100632	47	13.7%	0.29 [−0.30, 0.88]
**Subtotal (95% CI)**			**158**			**144**	**36.3%**	**0.09 [−0.33, 0.51]**
Heterogeneity: Tau² = 0.00; Chi² = 0.93, df = 3 (P = 0.82); I² = 0%								
Test for overall effect: Z = 0.42 (P = 0.68)								
**Total (95% CI)**			**474**			**432**	**100.0%**	**0.32 [−0.02, 0.67]**
Heterogeneity: Tau² = 0.13; Chi² = 17.50, df = 11 (P = 0.09); I² = 37%								
Test for overall effect: Z = 1.86 (P = 0.06)								
Test for subgroup differences: Chi² = 1.36, df = 2 (P = 0.51), I² = 0%								

Note: Results from the non-cirrhotic and cirrhotic patient groups in Sayyad et al (2012) have been combined using the formulae recommended in Cochrane Handbook [22].

Härmälä S, *et al. BMJ Open* 2019; 9:e031070. doi: 10.1136/bmjopen-2019-031070

Figure 8. Additional analysis: Mean difference between geometric mean HI antibody titre ratio after influenza vaccination in liver disease patients compared to healthy individuals

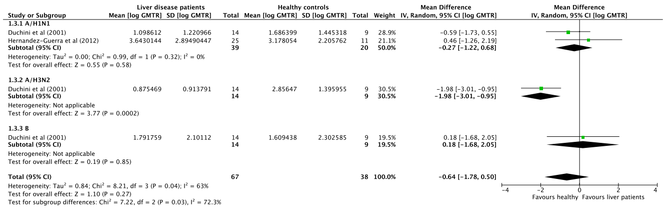

Note: Results from the non-cirrhotic and cirrhotic patient groups in Sayyad et al (2012) have been combined using the formulae recommended in Cochrane Handbook [22].

Figure 9. Additional analysis: Probability of seroconversion after influenza vaccination in liver disease patients compared to healthy individuals

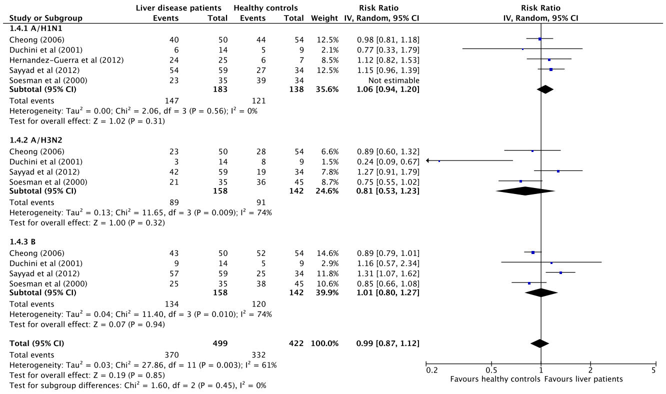

Note: Results from the non-cirrhotic and cirrhotic patient groups in Sayyad et al (2012) have been combined using the formulae recommended in Cochrane Handbook [22].

Figure 10. Additional analysis: Probability of seroprotection after influenza vaccination in liver disease patients compared to healthy individuals

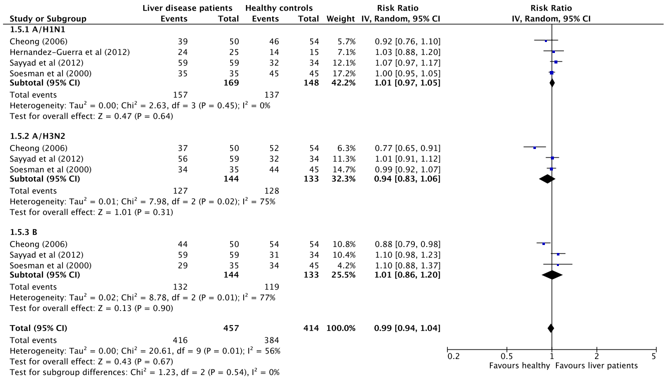

Note: Results from the non-cirrhotic and cirrhotic patient groups in Sayyad et al (2012) have been combined using the formulae recommended in Cochrane Handbook [22].

Härmälä S, *et al*. *BMJ Open* 2019; 9:e031070. doi: 10.1136/bmjopen-2019-031070

Figure 11. Additional analysis: Difference in seroprotection rate before vaccination in liver disease patients compared to healthy individuals

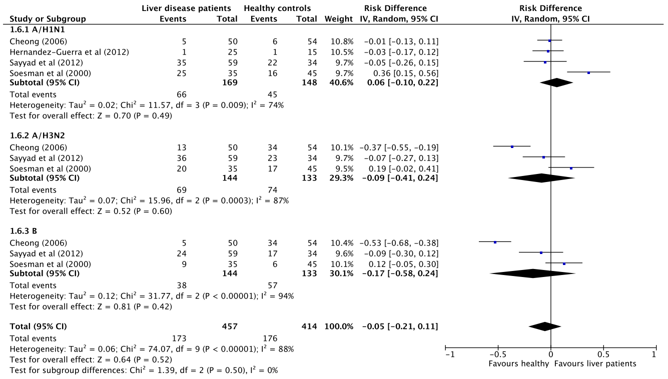

Note: Results from the non-cirrhotic and cirrhotic patient groups in Sayyad et al (2012) have been combined using the formulae recommended in Cochrane Handbook [22].

Table 10. Risk of all-cause mortality in the general population of the included studies where liver patients formed a subgroup

Study	Number of vaccinated/unvaccinated	Adjusted HR (95%CI) in the influenza season	Notes
Campitelli et al (2010)	14512/11410	0·61 (0·47–0·79)	Protective effect was present also outside the influenza season indicating presence of frailty bias.
Castilla et al (2015)	60400/42756[a]	0·84 (0·76–0·93)	Effect outside the seasons was not protective
Vila-Córcoles et al (2007	6051/5189	0·63 (0·54–0·74)	Effect outside the seasons was not protective

[a]These numbers are for the first of the two seasons studied. Number of patients and the % of vaccinated were similar for the second season.

References

1 Castilla J, Morán J, Martínez-Artola V, *et al*. Effectiveness of the monovalent influenza A(H1N1) 2009 vaccine in Navarre, Spain, 2009–2010: Cohort and case-control study. *Vaccine* 2011;**29**:5919–24. doi:10.1016/j.vaccine.2011.06.063

2 Cheng AC, Brown SG, Waterer GW, *et al*. Influenza epIdemIology, vaccIne coverage and vaccIne effectIveness in sentInel AustralIan hospItals in 2012: the Influenza ComplIcatIons Alert Network (FluCAN). *Commun Dis Intell* 2013;**37**:E246-252.

3 Cheng AC, Dwyer DE, Holmes M, *et al*. Influenza epIdemIology, vaccIne coverage and vaccIne effectIveness in sentInel AustralIan hospItals in 2013: the Influenza ComplIcatIons Alert Network. *Commun Dis Intell* 2014;**38**:E143-149.

4 Choi WS, Noh Y, Seo Y bin, *et al*. Case-Control Study of the Effectiveness of the 2010-2011 Seasonal Influenza Vaccine for Prevention of Laboratory-Confirmed Influenza Virus Infection in the Korean Adult Population. *Clin Vaccine Immunol* 2013;**20**:877–81. doi:10.1128/CVI.00009-13

5 Choi WS, Noh JY, Baek JH, *et al*. Suboptimal Effectiveness of the 2011 – 2012 Seasonal Influenza Vaccine in Adult Korean Populations. *PLoS One* 2015;**10**:e0098716. doi:10.1371/journal.pone.0098716

6 Castilla J, Martinez-Artola V, Salcedo E, *et al*. Vaccine effectiveness in preventing influenza hospitalizations in Navarre, Spain, 2010-2011: Cohort and case-control study. Vaccine. 2012;**30**:195–200.

7 Castilla J, Godoy P, Dominguez A, *et al*. Influenza vaccine effectiveness in preventing outpatient, inpatient, and severe cases of laboratory-confirmed influenza. Clin. Infect. Dis. 2013;**57**:167–75. doi:10.1093/cid/cit194

8 DiazGranados CA, Dunning AJ, Jordanov E, *et al*. High-dose trivalent influenza vaccine compared to standard dose vaccine in elderly adults: Safety, immunogenicity and relative efficacy during the 2009-2010 season. *Vaccine* 2013;**31**:861–6. doi:http://dx.doi.org/10.1016/j.vaccine.2012.12.013

9 Emborg H-D, Krause TG, Hviid A, *et al*. Effectiveness of vaccine against pandemic influenza A/H1N1 among people with underlying chronic diseases: Cohort study, Denmark, 2009-10. BMJ. 2012;**344**:d7901. doi:https://doi.org/10.1136/bmj.d7901

10 Mangtani P, Cumberland P, Hodgson CR, *et al*. A cohort study of the effectiveness of influenza vaccine in older people, performed using the United Kingdom general practice research database. J. Infect. Dis. 2004;**190**:1–10.

11 Olafsdottir TA, Alexandersson KF, Sveinbjornsson G, *et al*. Age and Influenza-Specific Pre-Vaccination Antibodies Strongly Affect Influenza Vaccine Responses in the Icelandic Population whereas Disease and Medication Have Small Effects. *Front Immunol* 2017;**8**:1872. doi:10.3389/fimmu.2017.01872

12 Simpson C, Lone N, Kavanagh K, *et al*. Seasonal Influenza Vaccine Effectiveness (SIVE): an observational retrospective cohort study - exploitation of a unique community-based national-linked database to determine the effectiveness of the seasonal trivalent influenza vaccine (Structured abstrac. *Heal Technol Assess Database* 2013;**1**.

13 Wang CS, Wang ST, Lai Lee CT, *et al*. Reducing major cause-specific hospitalization rates and shortening hospital stays after influenza vaccination. *Clin Infect Dis* 2004;**39**:1604–10. doi:http://dx.doi.org/10.1086/425323

14 Wang CS, Wang ST, Lai CT, *et al*. Impact of influenza vaccination on major cause-specific mortal. Vaccine. 2007;**25**:1196–203.

15 Zivich PN, Tatham L, Lung K, *et al*. Influenza vaccination status and outcomes among influenza-associated hospitalizations in Columbus, Ohio (2012-2015). *Epidemiol Infect* 2017;**145**:3284–93. doi:10.1017/S0950268817002163

16 Cheong H-J, Song J-Y, Park J-W, *et al*. Humoral and cellular immune responses to influenza vaccine in patients with advanced cirrhosis. *Vaccine* 2006;**24**:2417–22. doi:10.1016/j.vaccine.2005.11.064

17 Duchini A, Hendry RM, Nyberg LM, *et al*. Immune response to influenza vaccine in adult liver transplant recipients. *Liver Transplant* 2001;**7**:311–3. doi:10.1053/jlts.2001.23010

18 Hernandez-Guerra M, Gonzalez-Mendez Y, de Molina P, *et al*. Immunogenicity and Acceptance of Influenza A (H1N1) Vaccine in a Cohort of Chronic Hepatitis C Patients Receiving Pegylated-Interferon Treatment. *PLoS One* 2012;**7**:e48610. doi:10.1371/journal.pone.0048610

19 Ohfuji S, Fukushima W, Tamori A, *et al*. Immunogenicity of influenza A(H1N1)pdm09 vaccine and the associated factors on lowered immune response in patients with hepatitis C. *Influenza Other Respi Viruses* 2013;**7**:456–65. doi:10.1111/j.1750-2659.2012.00424.x

20 Sayyad B, Alavian SM, Najafi F, *et al*. Efficacy of Influenza Vaccination in Patients with Cirrhosis and Inactive Carriers of Hepatitis B Virus Infection. *Iran Red Crescent Med J* 2012;**14**:623–30.

21 Soesman NMR, Rimmelzwaan GF, Nieuwkoop NJ, *et al*. Efficacy of influenza vaccination in adult liver transplant recipients. *J Med Virol* 2000;**61**:85–93. doi:https://doi.org/10.1002/(SICI)1096-

9071(200005)61:1<85::AID-JMV14>3.0.CO;2-H
22 The Cochrane Collaboration. Cochrane Handbook for Systematic Reviews of Interventions. Version 5.1.0 [updated March 2011]. 2011.http://handbook-5-1.cochrane.org/ (accessed 25 Jul 2018).

Härmälä S, *et al. BMJ Open* 2019; 9:e031070. doi: 10.1136/bmjopen-2019-031070
