# Supplementary material for: Effectiveness of influenza vaccines in adults with chronic liver disease: a systematic review and meta-analysis
Source: BMJ Open. 2019 Sep 6;9(9):e031070. doi: 10.1136/bmjopen-2019-031070 (PMC6731888; doi:10.1136/bmjopen-2019-031070)
Supplement: Author's manuscript [file bmjopen-2019-031070.draft_revisions.pdf]

BMJ Open

BMJ Open is committed to open peer review. As part of this commitment we make the peer review history of every article we publish publicly available.

When an article is published we post the peer reviewers' comments and the authors' responses online. We also post the versions of the paper that were used during peer review. These are the versions that the peer review comments apply to.

The versions of the paper that follow are the versions that were submitted during the peer review process. They are not the versions of record or the final published versions. They should not be cited or distributed as the published version of this manuscript.

BMJ Open is an open access journal and the full, final, typeset and author-corrected version of record of the manuscript is available on our site with no access controls, subscription charges or pay-per-view fees (http://bmjopen.bmj.com).

If you have any questions on BMJ Open's open peer review process please email info.bmjopen@bmj.com

BMJ Open

The effectiveness of influenza vaccines in adults with chronic liver disease: a systematic review and meta-analysis

Journal:	*BMJ Open*
Manuscript ID	bmjopen-2019-031070
Article Type:	Research
Date Submitted by the Author:	15-Apr-2019

Complete List of Authors:	Härmälä, Suvi; University College London, Institute of Health Informatics<br>Parisinos, Constantinos; University College London, Institute of Health Informatics<br>Shallcross, Laura; University College London, Institute of Health Informatics<br>O'Brien, Alastair; UCL, Division of Medicine<br>Hayward, Andrew; University College London, Institute of Epidemiology and Health Care

Keywords:	Cirrhosis, Vaccine effectiveness, Hospitalisation, Seroprotection, Liver disease, Influenza vaccine

SCHOLARONE™
Manuscripts

Title: The effectiveness of influenza vaccines in adults with chronic liver disease: a systematic review and meta-analysis

Authors:

Suvi Härmälä, Institute of Health Informatics, University College London, 222 Euston Road, London NW1 2DA, United Kingdom. Email: suvi.harmala.14@ucl.ac.uk

Constantinos A. Parisinos, Institute of Health Informatics, University College London, 222 Euston Road, London NW1 2DA, United Kingdom. Email: c.parisinos@ucl.ac.uk

Laura Shallcross, Institute of Health Informatics, University College London, 222 Euston Road, London NW1 2DA, United Kingdom. Email: l.shallcross@ucl.ac.uk

Alastair O'Brien, Division of Medicine, University College London, Rayne Building, 5 University Street, London WC1E 6JJ, United Kingdom. Email: a.o'brien@ucl.ac.uk

Andrew Hayward, Institute of Epidemiology and Health Care, University College London, 1-19 Torrington Place, London WC1E 7HB, United Kingdom. Email: a.hayward@ucl.ac.uk

Correspondence to: Suvi Härmälä, Institute of Health Informatics, University College London, 222 Euston Road, London NW1 2DA, United Kingdom. Tel: +44 (0) 782 430 3142. Email: suvi.harmala.14@ucl.ac.uk

For peer review only

ABSTRACT

Objectives: Patients with liver disease frequently require hospitalisation with infection often the trigger. Influenza vaccination is an effective infection prevention strategy in healthy and elderly populations but is often perceived less beneficial in liver disease. We therefore investigated whether influenza vaccination triggered serological response and prevented hospitalisation and death in adults with chronic liver disease.

Data sources and data extraction: MEDLINE, EMBASE, Pubmed and CENTRAL were searched up to January 2019. Two reviewers screened studies, extracted data and assessed risk of bias and quality of evidence.

Primary and secondary outcome measures: Primary outcomes were all-cause hospitalisation and mortality. Secondary outcomes were cause-specific hospitalisation and mortality, and serological vaccine response. Random effects meta-analysis was used to estimate pooled effects of vaccination.

Results: Our searches provided 10649 unique records, 286 were eligible for full-text review and 12 studies were included. Majority of the patients had viral liver disease. Both non-cirrhotic and cirrhotic patients mounted an antibody response to the vaccine and the response was similar to that of healthy. Current evidence on clinical outcomes is limited but shows that seasonal influenza vaccination is associated with a reduction in risk of hospital admission from 205/1000 to 149/1000 (risk difference -0·06, 95% CI [-0·07, -0·04]) in patients with viral liver disease. Vaccinated patients were 27% less likely to be admitted to hospital compared to unvaccinated patients (risk ratio 0·73,

For peer review only

95% CI [0·66, 0·80]). Vaccination had no effect on all-cause or cause-specific mortality or cause-specific hospitalisation.

Conclusions Influenza vaccination represents a safe, low-cost and readily available treatment to prevent frequent hospitalisations in liver disease but its uptake is poor. Our review provides sufficient evidence of vaccine effectiveness for physicians and public health agencies to actively promote vaccination of liver patients, a group at a high risk of serious outcomes from influenza.

PROSPERO registration number CRD42017067277

Keywords: cirrhosis, hospitalisation, influenza vaccine, liver disease, seroprotection, vaccine effectiveness

Abbreviations: CI, confidence interval; GMT, geometric mean titre; GMTR, geometric mean titre ratio; GRADE, Grading of Recommendations Assessment, Development and Evaluation; HBV, hepatitis B virus; HCV, hepatitis C virus; HI, hemagglutinisation-inhibition; ILI, influenza-like illness; MD, mean difference; NOS, Newcastle-Ottawa scale; ORBIT, Outcome Reporting Bias in Trials; RR, risk ratio; WHO, World Health Organization

For peer review only

STRENGTHS AND LIMITATIONS OF THIS STUDY

- The review was conducted following established guidelines and a prospectively specified protocol
- The review focused on all-cause outcomes to reduce the risk of underestimating the vaccine impact that exists due to potential difficulties in identifying and accurately recording the cause of hospitalisation and death in patients with an underlying chronic disease.
- To reduce the risk of missing relevant studies and relevant data and to increase the objectivity of the review, two independent reviewers screened the studies, extracted data and assessed the risk of bias and quality of evidence.
- Differences in studies included in meta-analyses, publication bias, restriction on the publication language and varying response to queries about data on liver disease subgroups may have affected the effects observed in this review.

For peer review only

INTRODUCTION

Greater than 45 million people globally, including more than 8 million people in the US and Europe, are affected by chronic liver disease[1]. The prevalence of liver disease is increasing, driven by obesity and alcohol consumption.

The progression of liver disease is associated with immune dysregulation[2–5] and complications from common acute infections such as influenza cause significant morbidity and mortality. A 2-fold increased risk of hospital admission for influenza was observed in liver disease patients in 19 hospitals in Russia, Turkey, China, and Spain during the 2013/2014 season[6]. Similarly, an analysis of data on laboratory-confirmed influenza cases collected from several World Health Organization (WHO) member states during the 2009 influenza A (H1N1) pandemic found liver disease patients to have a greater than 5-fold increased risk of influenza-related hospitalisation and over 17-fold increased risk of death compared to that of healthy individuals[7]. Influenza infection, while not directly targeting the liver, may cause collateral liver damage[8] and trigger hepatic decompensation in liver disease[9].

Influenza vaccine introduces a whole or part of the influenza virion into the body stimulating antibody production against the virus, similar to an actual infection. In healthy adult and elderly populations, seasonal influenza vaccination is an effective way to prevent influenza infection[10,11]. People with liver disease, due to the high risk of severe health complications from influenza infection, are often a target of influenza vaccination policies[12] but randomised trials on vaccine effectiveness in liver patients are lacking as withholding the vaccine may place patients' safety and health at risk. For

For peer review only

example, a previous systematic review of randomised trials of influenza vaccine effectiveness in patients with chronic diseases, conducted in 2011[13], identified only one study in liver patients. Observational studies offer an alternative way of investigating vaccine effectiveness but without randomisation of patients the results are vulnerable to confounding and bias, in particular frailty bias. Frailty bias exist when some or all of the reduction in the risk of health complications may be due to the preferential receipt of vaccine by individuals who are in general more healthy. One solution to minimise the effect of frailty bias is to observe the vaccine effect both within and outside of the influenza season. If frailty bias exist, we would see higher rates of health complications in unvaccinated individuals outside of the influenza season. This would indicate that any protective effect observed within the season may be partially or fully due to the vaccinated individuals being healthier overall and an overestimation of the effect of the vaccine. Finally, in addition to measuring clinical outcomes to understand the effects of the vaccine, the antibody response elicited by the vaccine can be measured and used as a proxy for vaccine effectiveness.

There is uncertainty amongst primary and secondary care physicians whether influenza vaccines are able to trigger an appropriate antibody response and protect patients with liver disease from health complications[14], and the vaccination coverage is poor. Just over 55% of the chronic liver disease respondents to the 2016 US National Health Interview Survey[15] had been vaccinated against influenza in the past 12 months and less than 50% of working-age patients with liver disease registered with primary care practitioners in England had received influenza vaccination during the 2015/2016 season[16]. To inform vaccination policy and to help guide decision-making regarding vaccination, our review aims to provide a systematic synthesis of the available evidence

For peer review only - http://bmjopen.bmj.com/site/about/guidelines.xhtml

from both observational studies and randomised trials on the effectiveness of influenza vaccines to prevent serious health outcomes, hospitalisation and death, in adults with chronic liver disease. To address uncertainty over the vaccines' ability to trigger antibody response in liver patients, we also assess the effects of influenza vaccines on serological response in adults with chronic liver disease.

MATERIAL AND METHODS

Search strategy and selection criteria

Studies published in peer-reviewed journals were eligible for inclusion for this systematic review and meta-analysis if they met the following criteria: study type was randomised or non-randomised controlled trial, cohort or case-control study; intervention was an injectable, inactivated whole-virus, split-virus or subunit influenza vaccine; intervention was compared to placebo, alternative intervention or no vaccination (no comparison group was required for serological response); study population included at least ten ≥18 year-old adults with chronic liver disease of any severity or aetiology (by any classification) per comparison group; outcomes included all-cause hospitalisation or all-cause mortality (or supportive outcomes hospitalisation or mortality due to acute respiratory illness, influenza/ILI or liver disease complications) or serological response to influenza vaccine. We only included studies published in English. Review articles, case reports, cross-sectional studies, animal studies, editorials, clinical guidelines, studies with liver transplant patients only

For peer review only

(response to the vaccination and clinical outcomes are likely to be strongly influenced by the immunosuppressive medication rather than the status of liver disease), studies focused on special populations (such as pregnant women, nursing home residents, or patients with other chronic diseases), and studies retracted from publication were excluded.

We searched the Cochrane Central Register of Controlled Trials, Ovid MEDLINE, Ovid Embase and PubMed (originally up to 16 July 2017 and later updated up to 20 January 2019) for articles containing variations of terms "influenza", "flu", "seasonal", "TIV", "QIV", "3-valent", "4-valent", "trivalent" or "quadrivalent" in combination with terms "vaccine" or "immunisation" and "liver disease", "hepatic disease", "chronic liver", "chronic hepatic", or "cirrhosis". The search also included medical subject headings "Influenza Vaccines" (exploded), "Influenza, human /Prevention and Control", and "Liver Diseases" (exploded). The search was filtered by study design, age group of participants, and publication type. To capture studies where liver disease patients may have been included as part of a general population, the search was repeated leaving out the liver disease-specific terms and with the study design filter restricted to trials only. The full search strategy in MEDLINE and the medical subject headings for all databases searched are provided in Supplementary materials, Table 2 and Table 3. Electronic searches were complemented by manually searching recent influenza vaccine guidelines from Centers for Disease Control and Prevention, European Centre for Disease Control and Prevention, and WHO, and the reference lists of the included studies.

For peer review only - http://bmjopen.bmj.com/site/about/guidelines.xhtml

Titles of the articles identified through the search of the studies of influenza vaccination in the general population were first pre-screened by one reviewer. Abstracts and titles of articles meeting the pre-screening criteria and of all articles identified through the liver disease-specific search were then screened by two independent reviewers. Studies deemed eligible were included in the full-text review by two independent reviewers. Disagreements were resolved by discussion and the reasons for exclusion were recorded. In case of uncertainty over eligibility, study authors were contacted. Studies for which this uncertainty could not be resolved were excluded (Supplementary materials, Table 4).

Data analysis

Using standardised online forms, two review authors independently extracted data on: study participants (inclusion and exclusion criteria, method of recruitment/selection, study population characteristics), interventions and comparators (vaccine type, comparison treatment, dose, route of delivery, number and timing of vaccinations/comparator treatments, number of individuals and follow-up time in intervention and comparison groups), outcomes (definition, time points measured and reported, unit of measurement, number of outcomes in the intervention and control group, unadjusted and adjusted effect measures, covariates that the effect measures were adjusted for, comparisons, missing data and reasons for missingness, statistical methods used and processes for randomisation), study design (study type, country and setting, date of study, study duration, aim of study and withdrawals), study quality, study bias, study funding, and conflicts of interest. We contacted study authors to obtain

For peer review only

subgroup data, missing data, and to clarify unclear data. Effect measures were collected in the format in which they were reported and transformed for analysis as required.

Two review authors independently assessed the risk of bias. We used the Cochrane Collaborations tool[17] to assess randomised trials and the Newcastle-Ottawa scale (NOS)[18] to assess observational studies. Age, sex, severity, and aetiology of liver disease and presence of co-morbidities were considered the most important confounders in the assessment of observational studies. Additionally, in studies of pandemic vaccine effect on clinical outcomes, we considered as a potential confounder a previous seasonal influenza vaccination in the same season the study vaccine was administered. Risk of bias assessment in serological studies focused on the study population with liver disease. Disagreements over risk of bias were resolved by discussion between reviewers.

Where more than a single study per outcome was identified and the study designs, protocols and measures of treatment effect were considered similar enough to produce a meaningful pooled effect, we used random-effects meta-analysis to summarise the average effects of vaccination. For serological vaccine response, we compared haemagglutinisation inhibition (HI) antibody responses before and after vaccination. Comparisons between titres and titre ratios were presented as a mean difference (MD) of log geometric mean titre (GMT) or log geometric mean titre ratio (GMTR) with 95% confidence intervals (CI, titres and ratios were log transformed to back to their original scale to obtain standard deviations). Seroconversion (defined as the negative pre-vaccination serum converting to a HI titre >1:40 or at least a four-fold titre increase) and seroprotection (defined as the proportion of vaccinated individuals achieving a HI

For peer review only - http://bmjopen.bmj.com/site/about/guidelines.xhtml

titre of > 1:40) levels were presented as prevalence rates (%) with 95% CI and comparisons between rates were presented as risk ratio (RR) with 95% CI. As reference levels, for a theoretically adequate response, we used 40% for seroconversion and 70% for seroprotection. Reported rates expressed only as percentages that did not correspond to an integer number of patients were imputed to the closest possible number of patients (closest integer above the reported non-integer for baseline and closest integer below for effect rates). Response measures were presented in categories by virus subtype. The vaccine effects on hospitalisation and mortality were compared between vaccinated and unvaccinated liver disease patients and presented as a crude RR with 95% CI. Effect measures from multiple corresponding seasons were combined into an overall measure within each study. Presence of frailty bias were evaluated by contrasting the effect estimates in and out of influenza season. Person-time was presented as person-seasons for seasonal and as person-years for whole-year effect estimates.

We regarded heterogeneity between studies as substantial if τ^2 was greater than 0, I^2 was more than 30% and the p-value for Q-statistic was less than 0.10. Subgroup analyses and meta-regression were not carried out due to the low number of studies. Sensitivity analyses were carried out to investigate whether the serological vaccine effects were similar in less severe (non-cirrhotic disease) and advanced liver disease (cirrhotic disease) comparing response in all patients to that in cirrhotic patients only. We were not able to conduct sensitivity analyses based on aetiology of disease as the study populations were mainly of mixed aetiologies. In studies that included a healthy control population, we compared antibody responses between liver disease patients and the controls. Statistical analyses were performed using Stata 14.0 and RevMan 5.3.

For peer review only - http://bmjopen.bmj.com/site/about/guidelines.xhtml

For each of the review outcomes, we evaluated the risk of selective outcome reporting bias using the Outcome Reporting Bias in Trials (ORBIT) classification system[19]. Presence of publication bias was assessed based on this evaluation and our review design (funnel plots or formal tests were not used because of the low number of studies). The Grading of Recommendations Assessment, Development and Evaluation (GRADE) Working Group system[20] was used to assess and report the overall quality of evidence. In this assessment, we considered a vaccine effect outside of the influenza season (suggesting the effect of frailty bias) for clinical outcomes as a marker of imprecision. All assessments were completed by two independent reviewers and disagreements were resolved by discussion between reviewers.

This systematic review and meta-analysis followed the Preferred Reporting Items for Systematic Review and Meta-Analyses (PRISMA)[21]. The review protocol was specified in advance as part of a wider review plan that also includes a study of the effectiveness of pneumococcal vaccine. The protocol was registered in PROSPERO 13th June 2017 (registration number CRD42017067277) and published after peer-review on 16th March 2018[22]. Based on the peer-review feedback, we made minor amendments to the initial protocol (Supplementary materials, Table 1).

Role of the funding source

The funder of this study had no role in study design, data collection, data analysis, data interpretation, or writing of the report. The corresponding author had full access to all

For peer review only - http://bmjopen.bmj.com/site/about/guidelines.xhtml

the data in the study and had final responsibility for the decision to submit for publication.

Patient and public involvement

This review was conducted without patient involvement.

RESULTS

The search of influenza studies in the general population provided a total of 10649 unique study records. After title screening, 1607 of these records and 608 unique records from the liver disease-specific search were included in the abstract and title screening. The full text of 286 studies was examined in detail, and of these, ten studies met the inclusion criteria for the review. An additional two studies were identified through recent influenza vaccine guidelines, making the total number of included studies 12. The study flowchart is presented in Figure 1.

The included studies consisted of one randomized controlled trial and 11 cohort studies. Six studies included serological outcomes (Table 1) and six included clinical outcomes (Table 2). The studies with serological outcomes had low (4 studies) to moderate bias (2 studies) in selection of study population and low (2 studies), moderate (3 studies) and high bias (1 study) in assessment of the outcomes (bias mostly due to lack of description of independent, blind outcome assessment). Individual studies with clinical outcomes had moderate to low risk of bias in selection of the study population (in two studies vaccination status was self-reported) and low risk of bias in outcome

For peer review only

assessment (majority used medical records). In most studies, the presence of confounding due to demographic and clinical characteristics was unclear (we did not have characteristics for the liver patient subgroups obtained from the studies conducted in the general population) and the results for control periods (non-influenza periods) were available only from two studies. High risk of selective outcome reporting was suspected in only one clinical study (outcome all-cause mortality) but mainly in serological studies (outcome GMTR). Evidence for all outcomes was judged as very low quality. Risk of bias and selective outcome reporting assessments, and the GRADE evidence profile are provided in Supplementary materials, Table 6, Table 7 and Table 8.

Table 1. Characteristics of the included studies on serological response to influenza vaccine

Study	Location	Design	Liver disease participants (% cirrhosis, % viral aetiology)	Sample size	Vaccine	Follow-up	Outcomes
Cheong et al (2006)[23]	South Korea	Cohort study with comparison group	Advanced cirrhosis patients (100% cirrhosis 72% viral aetiology)	50 (54 healthy controls)	Trivalent, split-virus	4 weeks	GMT<br><br>Seroconversion rate<br><br>Seroprotection rate
Duchini et al (2001)[24]	United States	Cohort study with comparison groups	Cirrhosis patients (100% cirrhosis, 56% viral aetiology	14 (9 healthy controls)	Trivalent, split-virus	6 weeks	GMT<br><br>GMTR<br><br>Seroconversion rate
Hernández-Guerra et al (2012)[25]	Canary Islands, Spain	Cohort study with comparison groups	Chronic HCV patients (0% cirrhosis, 100% viral aetiology)	25 (15 healthy controls)	Monovalent, split virus	4-11 weeks	GMT<br><br>GMTR<br><br>Seroconversion rate<br><br>Seroprotection rate
Ohfuji et al (2013)[26]	Japan	Cohort study	Chronic HCV patients (25% cirrhosis,	79	Monovalent, split virus	3 weeks	GMT<br><br>GMTR

For peer review only

			100% viral aetiology)				Seroconversion rate<br><br>Seroprotection rate
Sayyad et al (2012)[27]	Iran	Cohort study with comparison group	Cirrhosis patients and inactive HBV carriers (47% cirrhosis, 100% viral aetiology in non-cirrhotics, aetiologies not provided in cirrhotics)	28, 31 (34 healthy controls)	Trivalent, subunit	4 weeks	GMT<br><br>Seroconversion rate<br><br>Seroprotection rate
Soesman et al (2000)[28]	The Netherlands	Cohort study with comparison groups	Cirrhosis patients (100% cirrhosis, aetiologies not provided)	36 (45 healthy controls)	Trivalent, subunit	4 weeks	GMT<br><br>Seroconversion rate<br><br>Seroprotection rate

Table 2. Characteristics of the included studies on clinical outcomes after influenza vaccination

Study	Location	Design	Liver disease participants	Sample size (liver disease patients)	Study period	Outcomes
Campitelli et al (2010)[29]	Canada	Cohort study	Chronic liver disease patients among the community-dwelling ≥65 year-olds	205 vaccinated/124 unvaccinated	8 influenza seasons 1996-1998 and 2001-2007	All-cause mortality<br><br>Acute respiratory illness-related hospitalisation (pneumonia & influenza)
Castilla et al (2015)[30]	Spain	Cohort study	Cirrhosis patients among the community-dwelling ≥65 year-olds	3126 person-seasons vaccinated/1804 person-seasons unvaccinated<br><br>Number of patients not reported.	2 influenza seasons 2011-2013	All-cause mortality
Ohfuji et al (2014)[31]	Japan	Cohort study	Chronic HCV hospital outpatients (64% ≥65 year-olds)	132 vaccinated/276 unvaccinated	1 influenza season	All-cause hospitalisation
Song et al (2007)[32]	South Korea	Randomised, controlled trial	Cirrhotic hospital in- and	175 vaccinated/90 unvaccinated	1 influenza season	Influenza illness-related mortality

For peer review only - http://bmjopen.bmj.com/site/about/guidelines.xhtml

			outpatients (50% ≥55 year-olds)		2004-2005	(laboratory-confirmed influenza)
Su et al (2016)[33]	Taiwan	Cohort study	Chronic HBV hospital outpatients (38% ≥60 year-olds)	4434 vaccinated/3646 unvaccinated	9 influenza seasons 2000-2009	All-cause hospitalisation<br><br>All-cause mortality<br><br>Liver disease complication related hospitalisation (septicaemia, bacteraemia, viraemia)
Vila-Córcoles et al (2007)[34]	Spain	Cohort study	Cirrhosis patients among the community-dwelling ≥65 year-olds	100-117 vaccinated/73-104 unvaccinated (numbers varied per year)	4 influenza seasons, 3 non-influenza periods, 3 full years 2002-2005	All-cause mortality

The serological response to influenza vaccine was assessed in six studies including a total of 262 liver disease patients (Table 1). Each study employed a cohort design in which all study participants were vaccinated. Mean age of liver disease participants ranged between 42 and 65 years (one study did not report baseline characteristics of participants). Three studies enrolled exclusively cirrhotic patients in their liver disease group, two studies enrolled both patients with and without cirrhosis and one study had separate groups for cirrhotic and non-cirrhotic patients. Hepatitis C was the main cause of liver disease in three studies and hepatitis B in two studies. In one study, aetiology was not specified. Four studies immunised the participants with a trivalent seasonal vaccine and two used a monovalent pandemic vaccine. In all studies, blood samples for post-vaccination measurements were drawn after minimum of three weeks and

For peer review only - http://bmjopen.bmj.com/site/about/guidelines.xhtml

antibody titres measured using a traditional HI assay (antibody titre data is provided in Supplementary materials, Table 5).

HI antibody levels of liver disease patients increased in response to vaccination (pooled random-effects MD of log GMTs 1·92, 95% CI [1·65, 2·19]) (Figure 2). There was no substantial heterogeneity between individual estimates (τ^2=0·04, I^2=15%, p=0·28) or levels against influenza A and B viruses (p=0·16). Seroconversion rate in liver disease patients to both A/H1N1 and B subtypes was clearly above the 40% reference level (pooled random-effects prevalence for A/H1N1 79·95%, 95% CI [68·19%, 89·70%] and pooled random-effects prevalence for B 86·82%, 95% CI [71·87%, 97·07%]) and around the 40% in the case of subtype A/H3N2 (pooled random-effects prevalence 55·53%, 95% CI [39·92%, 70·63%]). While the majority of the studies reported estimates above 40%, there was substantial heterogeneity between response rates (I^2=76·55% and p<0.01 for subtype A/H1N1; I^2=72·37% and p=0·01 for subtype A/H3N2; I^2=79·99% and p<0·01 for subtype B). Seroconversion rate is presented in Figure 3. Seroprotection rate in liver disease patients was above the 70% reference level (pooled random-effects rate 93·88%, 95% CI [87·33%, 98·40%]) (Figure 4). While the majority of studies reported a seroprotection rate above 70%, there was substantial heterogeneity (I^2=83·81%, p<0.01). Although some studies reported a substantial seroprotection level before vaccination, the pooled pre-vaccination seroprotection rate was clearly below 70% (Supplementary materials, Figure 1).

The effectiveness of influenza vaccine in preventing all-cause hospitalisation in liver disease patients was investigated in two cohort studies, published in 2014 and 2016[31,33]. A study of 408 participants from Japan[31] investigated pandemic vaccine

For peer review only

(against a single virus subtype) and enrolled exclusively chronic hepatitis C virus (HCV) patients. A study of 8080 participants from Taiwan[33] investigated seasonal vaccine (against multiple virus subtypes) exclusively in chronic hepatitis B virus (HBV) patients. In the study of HCV patients, patients were prospectively recruited from those who were under clinical follow-up for their liver disease but vaccination status was self-reported[31]. In the study of HBV patients, both liver disease and vaccination status were determined from electronic medical claim records[33]. One study presented results for one influenza season and the other for an entire year after vaccination and pooled the results from a period of nine years (Figure 5). Both studies compared the rate of all-cause hospital admission (first incidence) between vaccinated and unvaccinated individuals.

Vaccinated HCV patients, were no less likely to be hospitalised than unvaccinated patients during an influenza season after pandemic influenza A vaccination (RR 0·57, 95% CI [0·24, 1·37])[31]. In this study, hospitalisation outside the influenza season was not measured. Adjusting the effect estimate (for chronic co-morbidities, steroid treatment within the preceding 6 months and albumin level), calculated in the study as the odds ratio, did not reveal a significant effect[31]. Seasonal vaccination, however, was associated with a drop from 205/1000 patients to 149/1000 patients being hospitalised during a full year of follow-up (RD -0·06, 95% CI [-0·07, -0·04]). Vaccinated HBV patients were 27% less likely to be admitted to the hospital than unvaccinated patients (RR 0·73, 95% CI [0·66, 0·80])[33]. Both the crude and the adjusted time-to-event estimate that accounted for cirrhosis status, age, sex, pneumococcal vaccine, comorbidities and recent hospitalisation, calculated in the study as the hazard ratio (HR), showed a protective effect (crude HR 0·68, 95% CI [0·62, 0·76], adjusted HR 0.56,

For peer review only - http://bmjopen.bmj.com/site/about/guidelines.xhtml

95% CI [0·50, 0·62])[33]. Based on a Kaplan-Meier plot, the difference in event-free survival probability between vaccinated and unvaccinated was established during the influenza season, approximately within the first three months after vaccination[33]. The effect of influenza vaccine on cause-specific hospitalisation was investigated in two studies[29,33]. A cohort study from Canada, published in 2010, measured acute respiratory illness-related hospitalisation during influenza season (defined as influenza/pneumonia hospitalisation) using medical claims and hospital records[29]. A total of 329 liver disease patients were included as part of a general population, however, as there were five or fewer hospitalisation events in both groups, the study authors were unable to release the results (reason: institution's data policy, personal communication). A liver disease complication-related hospitalisation outcome was included in the Taiwanese study of HBV patients[33]. Vaccinated liver disease patients were 57% less likely to be admitted to hospital for unspecified septicaemia, bacteraemia and viraemia than unvaccinated patients (RR 0·43, 95% CI [0·31, 0·59]). Vaccination was associated with a fall from 26/1000 patients to 11/1000 patients being hospitalised (RD -0·02, 95% [CI -0·02, -0·01]). It appeared, however, that a difference in event-free survival probability was not established during the influenza season (Kaplan-Meier plot)[33]. No studies with the outcome influenza illness/ILI-related hospitalisation were identified.

The effectiveness of influenza vaccine in preventing all-cause mortality in liver disease patients was investigated in five cohort studies[29,30,33,34], published between 2007 and 2016. Only the Taiwanese study of HBV patients, enrolled exclusively liver disease patients[33]. Two studies from Spain[30,34] and one from Canada[29] enrolled liver disease patients as part of the general, community-dwelling population. All studies

For peer review only - http://bmjopen.bmj.com/site/about/guidelines.xhtml

ascertained liver disease diagnosis and vaccination status from electronic health or medical claim records. In one study, the record-based vaccination status was based on self-reporting. The Spanish studies followed patients over multiple seasons from the start of the study allowing them to contribute to both the unvaccinated and vaccinated follow-up time for different seasons [30,34] whereas the other two studies followed patients up for a single season/year[29,33], although this was over multiple seasons/years. All studies compared all-cause mortality rates between vaccinated and unvaccinated individuals.

Results are presented in Figure 6. Vaccinated patients were no less likely to die during influenza season (pooled random effects RR 0·80, 95% CI [0·43, 1·50]), or the entire year (pooled random effects RR 0·41, 95% CI [0·11, 1·52]). All the studies that contributed to the influenza season-specific estimate reported non-significant effects and there was no substantial heterogeneity (τ^2=0·12, I^2=33·00%, p=0·23). The studies that contributed to the whole-year estimate were substantially heterogeneous (τ^2=0·83, I^2=94.00%, p<0.00), with the smaller Spanish study[34] reporting a non-significant effect (personal communication) and the larger Taiwanese study reporting a large protective effect (RR 0·22, 95% CI [0·17, 0·28])[33]. The Spanish study reported no effect outside the influenza period (RR 0·52, 95% CI [0·15, 1·83]). The Canadian study also measured the effect outside the non-influenza period but as there were five or fewer deaths in both groups, the authors were unable to provide the results (institution's data policy, personal communication). Based on a Kaplan-Meier plot presented in the Taiwanese study, the difference in survival probability was mainly established approximately in the first five months after vaccination[33], however, it was not clear whether the increase in death rate fully levelled off after the influenza season

For peer review only - http://bmjopen.bmj.com/site/about/guidelines.xhtml

and it may be that this to some extent contributed to the size of the yearly effect estimate in this study. The effect of influenza vaccine on the rate of cause-specific mortality was investigated in one study[32]. The effect of influenza vaccine in preventing influenza illness/ILI-related mortality was included as an outcome in a South Korean randomised, controlled trial of cirrhotic patients, published in 2007[32]. No deaths due to laboratory-confirmed influenza occurred among the 175 vaccinated or the 90 unvaccinated patients during the influenza season[32]. No studies investigating mortality related to acute respiratory illness or liver disease complications were identified.

To understand whether the vaccine effects were different based on the patients' cirrhosis status, we conducted sensitivity analyses. The serological response in cirrhotic patients did not differ from the results of our main analyses (Supplementary materials, Figures 2-5). We did not have the data and therefore were not able to conduct similar analyses for the clinical outcomes.

In addition to comparing the vaccine effects within liver patients, the studies of serological response (5/6 studies) also compared the outcomes between liver disease patients and healthy controls (individuals without liver disease). The antibody titres before or after vaccination, seroconversion rate and seroprotection rate of liver patients did not differ from those of healthy individuals. Pre- to post-vaccination titre ratios (GMTRs) reported were similar between liver patients and healthy individuals except for influenza subtype A/H3N2 where the mean fold-increase in antibody levels of liver patients was lower than that of healthy individuals. The pooled results for these comparisons are provided in Supplementary materials, Figures 6-11. The studies on

For peer review only - http://bmjopen.bmj.com/site/about/guidelines.xhtml

clinical outcomes in liver patients did not include healthy comparison groups and while we did not seek these data from the studies that included liver patients as part of a general population, the all-cause mortality results in the entire general population can be found in Supplementary materials, Table 9.

DISCUSSION

Six cohort studies from three continents (Asia, Europe and North America) investigated the serological vaccine response in liver disease patients. Two cohort studies and one randomised controlled trial, all from Asia, investigated the vaccine effect on hospitalisation and/or mortality in liver disease patients exclusively. In addition, there are at least three cohort studies, two from Europe and one from North America, that have assessed the vaccine effect on these outcomes in a general community-dwelling elderly population including a subgroup with liver disease. Overall, the available evidence suggests that while influenza vaccine may not protect against all-cause mortality, it triggers an effective antibody response and may reduce the risk of all-cause hospitalisation in liver disease patients (findings for clinical outcomes summarised in Table 3, maximum quality obtainable is “low” as all included studies are non-randomised).

Table 3. Summary of findings for clinical outcomes
Effectiveness of influenza vaccination in preventing all-cause hospitalisation and all-cause mortality in adults with chronic liver disease

For peer review only - http://bmjopen.bmj.com/site/about/guidelines.xhtml

For peer review only

	**Patients: Adults with chronic liver disease**<br>**Setting: General population**<br>**Intervention: Influenza vaccination (injectable, inactivated)**<br>**Comparison: No vaccination**					
**Outcome**	**Risk/rate in unvaccinated [a]**	**Risk/rate in vaccinated**	**Relative effect**	**Vaccine effectiveness**	**Number of participants/person-time (number and type of studies)**	**Quality of evidence (GRADE)**
All-cause hospitalisation, influenza season (pandemic vaccine)	80 per 1000	46 per 1000 (19-110)	0.57 (0.24, 1.37)	Not significant	408 (1 observational study)	Very low[b]
All-cause hospitalisation, all year	205 per 1000	150 per 1000 (135-164)	0.73 (0.66, 0.80)	27% (20%, 34%)[c]	8080 (1 observational study)	Very low[d]
All-cause mortality, influenza season	17 per 1000 person-seasons	14 per 1000 person-seasons (7-26)	0.80 (0.43, 1.50)	Not significant	5975 person-seasons (3 observational studies)	Very low[e]
All-cause mortality, all year	85 per 1000 person-seasons	35 per 1000 person-seasons (9-129)	0.41 (0.11, 1.52)	Not significant	8405 person-years (2 observational studies)	Very low[f]

[a] Median risk in unvaccinated across the studies.
[b] Rated down from low to very low based on study limitations, imprecision of the estimate and suspicion of publication bias.
[c] Could be applicable to HBV patients only as the study only enrolled HBV patients.
[d] Rated down from low to very low based on suspicion of publication bias.
[e] Rated down from low to very low based on study limitations, inconsistency in absolute rates, imprecision of the estimate and suspicion of publication bias.
[f] Rated down from low to very low based on study limitations, imprecision of the estimate and suspicion of publication bias.

A previous systematic review[13] on the effectiveness of inactivated influenza vaccine in different at-risk target groups identified only one randomised study in liver patients. This study of 311 cirrhosis patients, also included in our current review, found seasonal influenza vaccine effective against laboratory-confirmed influenza but not against ILI[32]. The study included hospitalised patients and could not investigate vaccine

For peer review only - http://bmjopen.bmj.com/site/about/guidelines.xhtml

effectiveness against hospitalisation. Vaccine effect on influenza-related mortality was investigated, however, no deaths occurred during the study. It is possible that the effects we found are not entirely due to the vaccine. Other factors that may have affected the clinical outcomes include frailty bias, previous vaccinations, social support, healthcare system, lifestyle, and in the severity or type of liver disease between the vaccinated and unvaccinated populations. Serological outcomes may have been further affected by variation of the antibody assays.

The data presented are predominantly from patients with viral liver disease. We cannot therefore be certain that our findings are applicable to patients with liver disease caused by alcohol, obesity or other non-viral factors. While sensitivity analyses showed that compared to non-cirrhotic liver disease, having cirrhosis did not worsen the antibody response to the vaccine, most data in the analyses of clinical outcomes come from patients with a milder form of liver disease than cirrhosis. We might expect that in severe liver disease complications from influenza are more frequent and we may have seen more outcome events if more cirrhotic patients were included. Given the proxy effectiveness in cirrhotic patients indicated by the antibody responses, this would mean that our study may have somewhat underestimated the true protective effect against clinical outcomes. However, all our data on the hospitalisation outcome are from high-income Asian countries and it is possible that the protective vaccine effect may not extend to populations from other geographical and income settings with different prevalence of co-morbidities, age distribution, influenza seasonality and vaccine formulation used.

For peer review only - http://bmjopen.bmj.com/site/about/guidelines.xhtml

Our review has several strengths. It was conducted in a transparent way following established guidelines and a prospectively specified protocol. The search was conducted over multiple databases and we searched for both studies in liver disease patients and studies in which liver disease patients were included as part of the general population. To reduce the risk of missing relevant studies and relevant data and to increase the objectivity of the review, two independent reviewers screened the studies, extracted data and assessed the risk of bias. We studied all-cause outcomes to take into account the complex interplay of a chronic condition and acute infections. An underlying diagnosis of chronic liver disease may influence how cause of death and hospitalisation is recorded. This potentially reduces the number of cases that are attributed to influenza and solely investigating diagnoses of influenza is likely to substantially underestimate the impact of the vaccine.

There are also limitations to our review. The meta-analyses combine data from studies that differ in terms of patient population, outcome definitions and vaccine used. For the studies that included liver disease patients as part of the general population, we had very limited information about the baseline characteristics of the liver patient subgroup. In addition, despite the comprehensive search, publication bias, restriction on publication language and varying response to queries about data on liver disease subgroups may have affected the effects observed. We were not able to employ translation services and may have omitted relevant studies published in languages other than English from countries such as China where influenza vaccines are manufactured and liver disease is a significant public health challenge. A number of studies potentially included liver disease patients as part of a wider study population but were unable to obtain confirmation and liver disease subgroup data from the study

For peer review only

authors. Finally, the body of evidence identified in this review consists almost exclusively of studies with a non-randomised design. While this decreases the overall quality of evidence, conducting randomised controlled trials in a high-risk population are ethically challenging.

Evidence from mainly patients with viral liver disease suggests that underlying liver disease does not prevent patients from mounting an antibody response to influenza vaccine and similar proxy effectiveness is seen both in non-cirrhotic and cirrhotic patients. We also identified a protective influenza vaccine effect against yearly all-cause hospitalisation in patients with viral liver disease. Assuming this effect – estimated in a large cohort study of over 8000 patients – is due to the vaccine, seasonal influenza vaccination of 14–24 viral liver disease patients could prevent one hospitalisation per year. It is uncertain whether the estimated effects extend to patients with liver disease of other than viral origin. No protective effect against all-cause mortality was found.

Current uptake of seasonal influenza vaccination is low. A recent systematic review identified low perceived vaccine effectiveness, and low concern and perceived risk of severe influenza infection as the most common barriers for seasonal influenza vaccination in patients with chronic illness[35]. Ideally, the evidence for influenza vaccine effectiveness in liver disease would come from a large randomised vaccine trial in liver disease patients. In the absence of this evidence, we must rely on proxy estimates for effectiveness from serological studies and evidence on clinical outcomes from observational studies. The best available evidence on the effectiveness of influenza vaccine in liver disease, summarised in this review, shows both proxy effectiveness and protection against all-cause hospitalisation. Therefore, we believe, this review provides

For peer review only - http://bmjopen.bmj.com/site/about/guidelines.xhtml

sufficient evidence for physicians and public health agencies to actively promote seasonal influenza vaccination of liver disease patients, a group at a high risk of serious outcomes from influenza infection.

Contributors

The study was conceived by SH, LS, AO and AH. SH developed the eligibility criteria, search strategy, risk of bias assessment strategy and data extraction plan with guidance from LS, AO and AH. SH (trained in systematic methods of literature searching as part of her PhD studies) performed the literature searches and pre-screening of titles. SH and CP reviewed titles and abstracts, and full text articles, extracted data, and assessed study bias and quality. SH analysed the data, and wrote the manuscript to which all authors CP, LS, AO and AH contributed.

Funding

This work was supported by the UK Biotechnology and Biological Sciences Research Council grant [grant number BBSRC BB/M009513/1] to SH.

Data sharing statement

All data relevant to the study are included in the article or uploaded as supplementary information.

Competing interests

The authors declare that no competing interests exist.

Acknowledgements

For peer review only

We wish to thank the authors of the reviewed studies for kindly responding to our enquiries and for providing liver patient subgroup data where possible.

Figure legends

Figure 1. Study selection

Figure 2. Serological response to influenza vaccination: mean difference in log HI antibody GMTs before and after vaccination. *Note: Sayyad et al (2012a) includes cirrhotic patients and Sayyad et al (2012b) includes non-cirrhotic patients only.*

Figure 3. Serological response to influenza vaccination: seroconversion rate after vaccination. *Note: Sayyad et al (2012a) includes cirrhotic patients and Sayyad et al (2012b) includes non-cirrhotic patients only.*

Figure 4. Serological response to influenza vaccination: seroprotection rate after vaccination. *Note: Sayyad et al (2012a) includes cirrhotic patients and Sayyad et al (2012b) includes non-cirrhotic patients only.*

Figure 5. Effect of influenza vaccine on clinical outcomes: all-cause hospitalisation in vaccinated compared to unvaccinated liver disease patients. Note: Ohfuji et al

For peer review only - http://bmjopen.bmj.com/site/about/guidelines.xhtml

(2014) investigated the effect of pandemic (monovalent) vaccine and Su et al (2016) of seasonal vaccine (against three or more virus subtypes).

Figure 6. Effect of influenza vaccine on clinical outcomes: all-cause mortality in vaccinated compared to unvaccinated liver disease patients.

For peer review only

For peer review only - http://bmjopen.bmj.com/site/about/guidelines.xhtml

REFERENCES

1 GBD 2016 Disease and Injury Incidence and Prevalence Collaborators. Global, regional, and national incidence, prevalence, and years lived with disability for 328 diseases and injuries for 195 countries, 1990–2016: a systematic analysis for the Global Burden of Disease Study 2016. *Lancet* 2017;**390**:1211–59. doi:10.1016/S0140-6736(17)32154-2

2 Fierer J, Finley F. Deficient Serum Bactericidal Activity Against Escherichia Coli in Patients with Cirrhosis of the Liver. *J Clin Invest* 1979;**63**:912–21. doi:10.1172/JCI109391

3 Hassner A, Kletter Y, Shlag D, *et al.* Impaired monocyte function in liver cirrhosis. *Br Med J (Clin Res Ed)* 1981;**282**:1262–3.

4 Brien AJO, Fullerton JN, Massey KA, *et al.* Prostaglandin E2 Mediates Immunosuppression in Acutely Decompensated Cirrhosis. *Nat Med* 2014;**20**:518–23. doi:10.1038/nm.3516

5 Rajkovic IA, Williams R. Abnormalities of Neutrophil Phagocytosis, Intracellular Killing and Metabolic Activity in Alcoholic Cirrhosis and Hepatitis. *Hepatology* 1986;**6**:252–62. doi:10.1002/hep.1840060217

6 Puig-Barberà J, Natividad-Sancho A, Trushakova S, *et al.* Epidemiology of Hospital Admissions with Influenza during the 2013 / 2014 Northern Hemisphere Influenza Season: Results from the Global Influenza Hospital Surveillance Network. *PLoS One* 2016;**11**:e0154970. doi:10.1371/journal.pone.0154970

7 Kerkhove MD Van, Vandemaele KAH, Shinde V, *et al.* Risk Factors for Severe Outcomes following 2009 Influenza A (H1N1) Infection: A Global Pooled Analysis. *PLoS Med* 2011;**8**:e1001053. doi:10.1371/journal.pmed.1001053

For peer review only

8 Polakos NK, Cornejo JC, Murray DA, *et al.* Kupffer Cell-Dependent Hepatitis Occurs during Influenza Infection. *Am J Pathol* 2006;**168**:1169–78. doi:10.2353/ajpath.2006.050875

9 Duchini A, Viernes ME, Nyberg LM, *et al.* Hepatic Decompensation in Patients With Cirrhosis During Infection With Influenza A. *Arch Intern Med* 2000;**160**:113–5. doi:10.1001/archinte.160.1.113

10 Demicheli V, Jefferson T, Ferroni E, *et al.* Vaccines for preventing influenza in healthy adults. *Cochrane Database Syst Rev* Published Online First: 2018. doi:10.1002/14651858.CD001269.pub6.www.cochranelibrary.com

11 Demicheli V, Jefferson T, Di Pietrantonj C, *et al.* Vaccines for preventing influenza in the elderly. *Cochrane Database Syst Rev* Published Online First: 2018. doi:10.1002/14651858.CD004876.pub4.www.cochranelibrary.com

12 European Centre for Disease Prevention and Control. Seasonal influenza vaccination and antiviral use in Europe – Overview of vaccination recommendations and coverage rates in the EU Member States for the 2013–14 and 2014–15 influenza seasons. 2016.

13 Michiels B, Govaerts F, Remmen R, *et al.* A systematic review of the evidence on the effectiveness and risks of inactivated influenza vaccines in different target groups. *Vaccine* 2011;**29**:9159–70. doi:10.1016/j.vaccine.2011.08.008

14 Leise MD, Talwalkar JA. Immunizations in chronic liver disease: what should be done and what is the evidence. *Curr Gastroenterol Rep* 2013;**15**:300.http://ovidsp.ovid.com/ovidweb.cgi?T=JS&PAGE=reference&D=medl&NEWS=N&AN=23250700

15 National Center for Health Statistics. National Health Interview Survey. Public-use data file and documentation. 2016.

For peer review only - http://bmjopen.bmj.com/site/about/guidelines.xhtml

16 Public Health England. Influenza immunisation programme for England: GP patient groups - Data collection survey Season 2015 to 2016. 2016.

17 The Cochrane Collaboration. Cochrane Handbook for Systematic Reviews of Interventions. Version 5.1.0 [updated March 2011]. 2011.http://handbook-5-1.cochrane.org/ (accessed 25 Jul 2018).

18 Wells G, Shea B, O'Connell D, *et al.* The Newcastle-Ottawa Scale (NOS) for assessing the quality of nonrandomised studies in meta-analyses. http://www.ohri.ca/programs/clinical_epidemiology/oxford.asp (accessed 25 Jul 2018).

19 Kirkham JJ, Dwan KM, Altman DG, *et al.* The impact of outcome reporting bias in randomised controlled trials on a cohort of systematic reviews. *BMJ* 2010;**340**:c365. doi:10.1136/bmj.c365

20 Guyatt GH, Oxman AD, Vist GE, *et al.* GRADE: an emerging consensus on rating quality of evidence and strength of recommendations. *BMJ* 2008;**336**:924–6. doi:10.1136/bmj.39489.470347.AD

21 Moher D, Liberati A, Tetzlaff J, *et al.* Academia and Clinic Annals of Internal Medicine Preferred Reporting Items for Systematic Reviews and Meta-Analyses: The PRISMA Statement. *Annu Intern Med* 2009;**151**:264–9. doi:10.1371/journal.pmed1000097

22 Härmälä S, Parisinos C, Shallcross L, *et al.* Effectiveness of pneumococcal and influenza vaccines to prevent serious health complications in adults with chronic liver disease: A protocol for a systematic review. *BMJ Open* 2018;**8**. doi:10.1136/bmjopen-2017-018223

23 Cheong H-J, Song J-Y, Park J-W, *et al.* Humoral and cellular immune responses to influenza vaccine in patients with advanced cirrhosis. *Vaccine* 2006;**24**:2417–22.

For peer review only

doi:10.1016/j.vaccine.2005.11.064

24 Duchini A, Hendry RM, Nyberg LM, *et al.* Immune response to influenza vaccine in adult liver transplant recipients. *Liver Transplant* 2001;**7**:311–3. doi:http://dx.doi.org/10.1053/jlts.2001.23010

25 Hernandez-Guerra M, Gonzalez-Mendez Y, de Molina P, *et al.* Immunogenicity and Acceptance of Influenza A (H1N1) Vaccine in a Cohort of Chronic Hepatitis C Patients Receiving Pegylated-Interferon Treatment. *PLoS One* 2012;**7**:e48610. doi:http://dx.doi.org/10.1371/journal.pone.0048610

26 Ohfuji S, Fukushima W, Tamori A, *et al.* Immunogenicity of influenza A(H1N1)pdm09 vaccine and the associated factors on lowered immune response in patients with hepatitis C. *Influenza Other Respi Viruses* 2013;**7**:456–65. doi:10.1111/j.1750-2659.2012.00424.x

27 Sayyad B, Alavian SM, Najafi F, *et al.* Efficacy of Influenza Vaccination in Patients with Cirrhosis and Inactive Carriers of Hepatitis B Virus Infection. *Iran Red Crescent Med J* 2012;**14**:623–30.

28 Soesman NMR, Rimmelzwaan GF, Nieuwkoop NJ, *et al.* Efficacy of influenza vaccination in adult liver transplant recipients. *J Med Virol* 2000;**61**:85–93. doi:https://doi.org/10.1002/(SICI)1096-9071(200005)61:1<85::AID-JMV14>3.0.CO;2-H

29 Campitelli MA, Rosella LC, Stukel TA, *et al.* Influenza vaccination and all-cause mortality in community-dwelling elderly in Ontario, Canada, a cohort study. *Vaccine* 2010;**29**:240–6. doi:http://dx.doi.org/10.1016/j.vaccine.2010.10.049

30 Castilla J, Guevara M, Martinez-Baz I, *et al.* Enhanced estimates of the influenza vaccination effect in preventing mortality a prospective cohort study. Med. (United States). 2015;**94**:e1240.

For peer review only

31 Ohfuji S, Fukushima W, Sasaki Y, *et al.* Influenza A(H1N1)pdm09 vaccine effectiveness and other characteristics associated with hospitalization in chronic liver disease patients. *Liver Int* 2014;**34**:700–6. doi:http://dx.doi.org/10.1111/liv.12295

32 Song JY, Cheong HJ, Ha SH, *et al.* Clinical impact of influenza immunization in patients with liver cirrhosis. *J Clin Virol* 2007;**39**:159–63. doi:10.1016/j.jcv.2007.04.018

33 Su F-H, Huang Y-L, Sung F-C, *et al.* Annual influenza vaccination reduces total hospitalization in patients with chronic hepatitis B virus infection: A population-based analysis. *Vaccine* 2016;**34**:120–7. doi:10.1016/j.vaccine.2015.10.129

34 Vila-Córcoles A, Rodriguez T, de Diego C, *et al.* Effect of influenza vaccine status on winter mortality in Spanish community-dwelling elderly people during 2002-2005 influenza periods. Vaccine. 2007;**25**:6699–707.

35 Schmid P, Rauber D, Betsch C, *et al.* Barriers of influenza vaccination intention and behavior - A systematic review of influenza vaccine hesitancy, 2005-2016. *PLoS One* 2017;**12**:e0170550. doi:10.1371/journal.pone.0170550

For peer review only

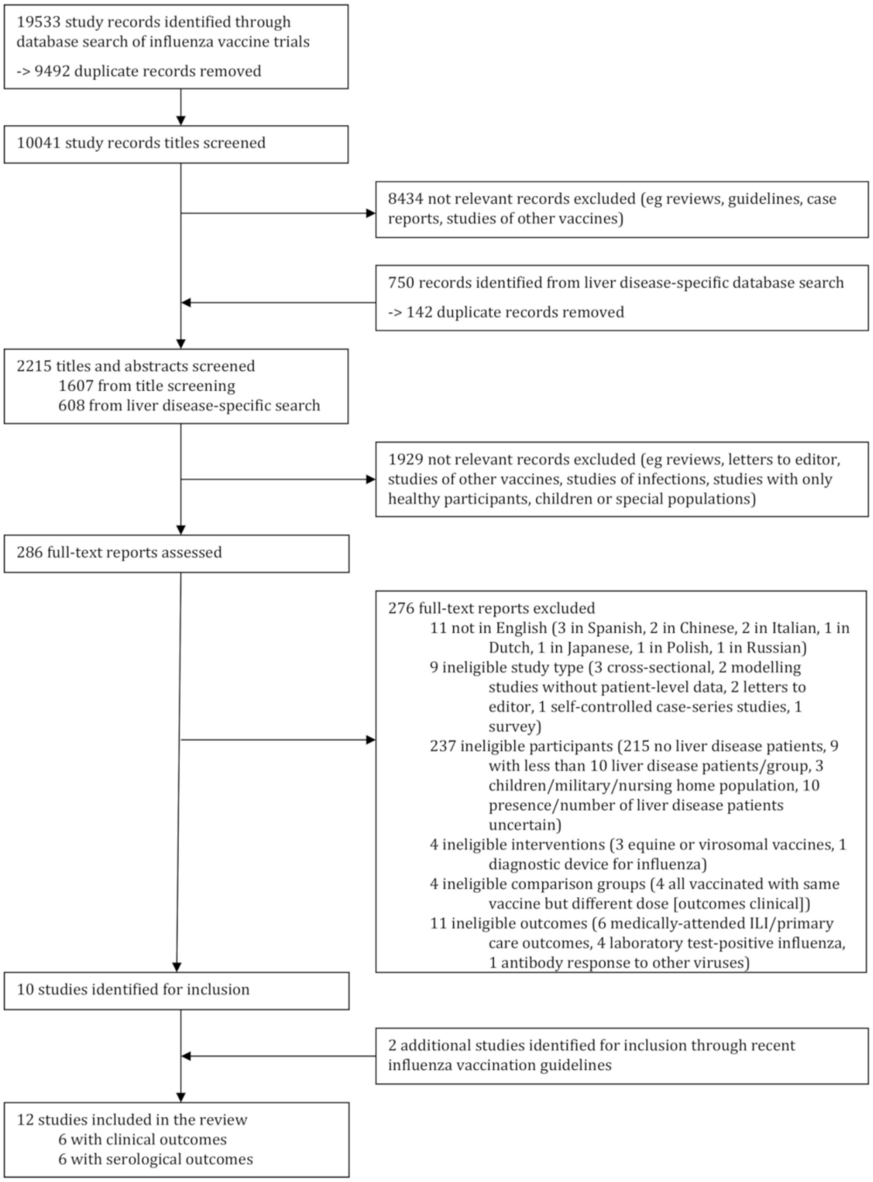

Figure 1. Study selection

150x202mm (300 x 300 DPI)

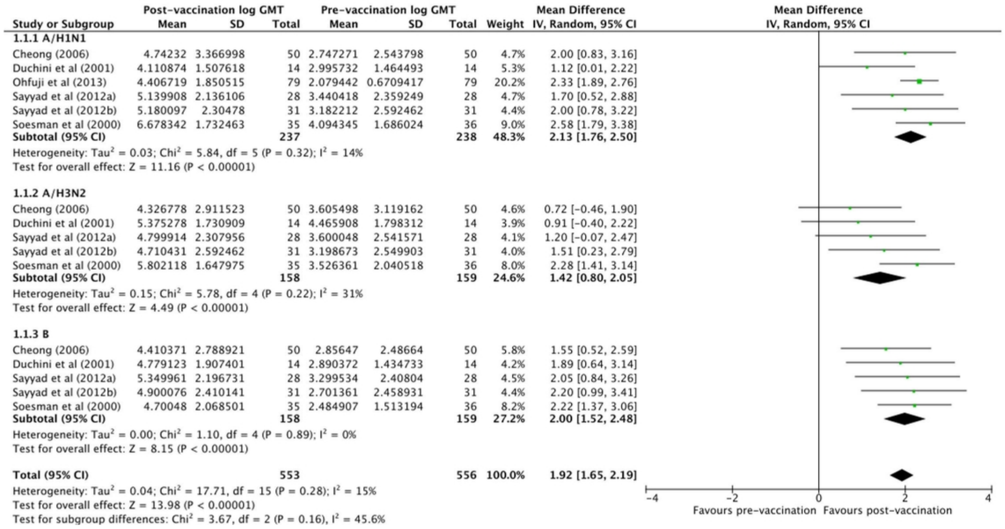

Figure 2. Serological response to influenza vaccination: mean difference in log HI antibody GMTs before and after vaccination. Note: Sayyad et al (2012a) includes cirrhotic patients and Sayyad et al (2012b) includes non-cirrhotic patients only.

96x50mm (300 x 300 DPI)

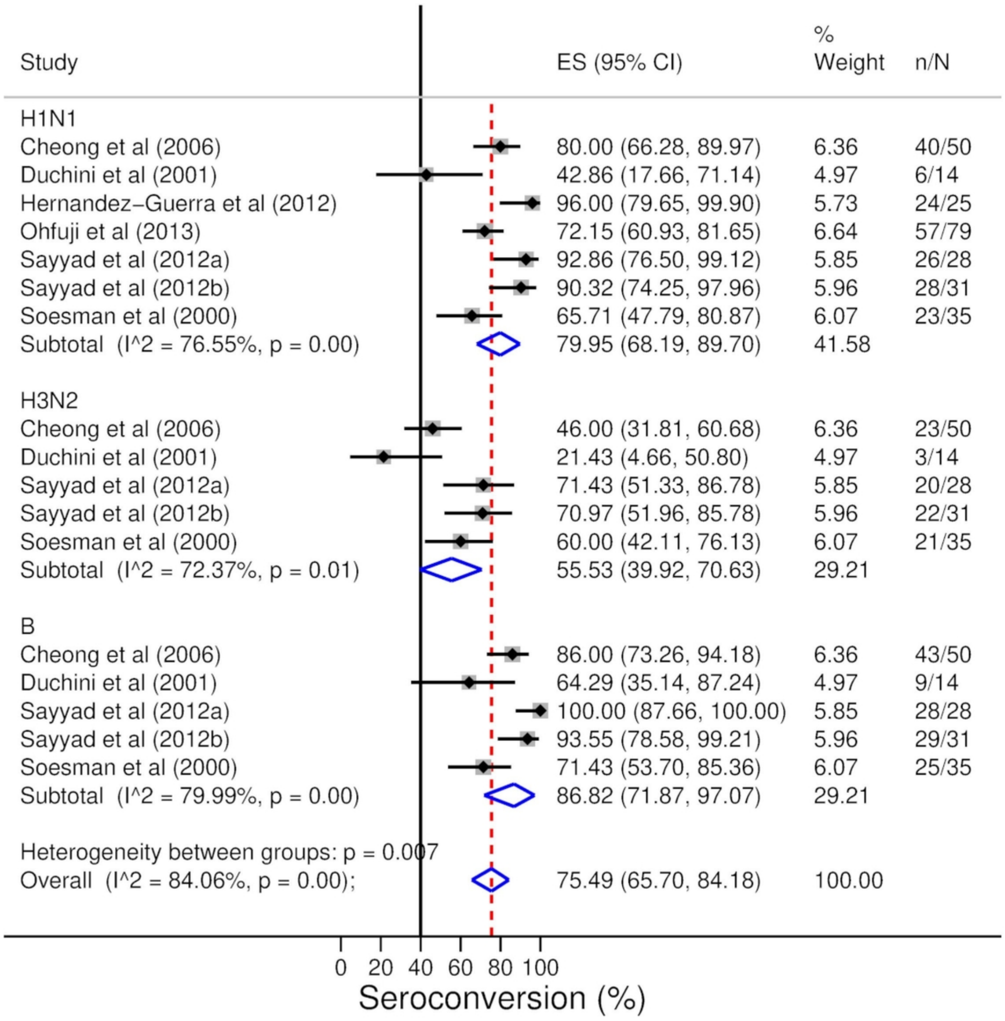

Figure 3. Serological response to influenza vaccination: seroconversion rate after vaccination. Note: Sayyad et al (2012a) includes cirrhotic patients and Sayyad et al (2012b) includes non-cirrhotic patients only.

96x97mm (300 x 300 DPI)

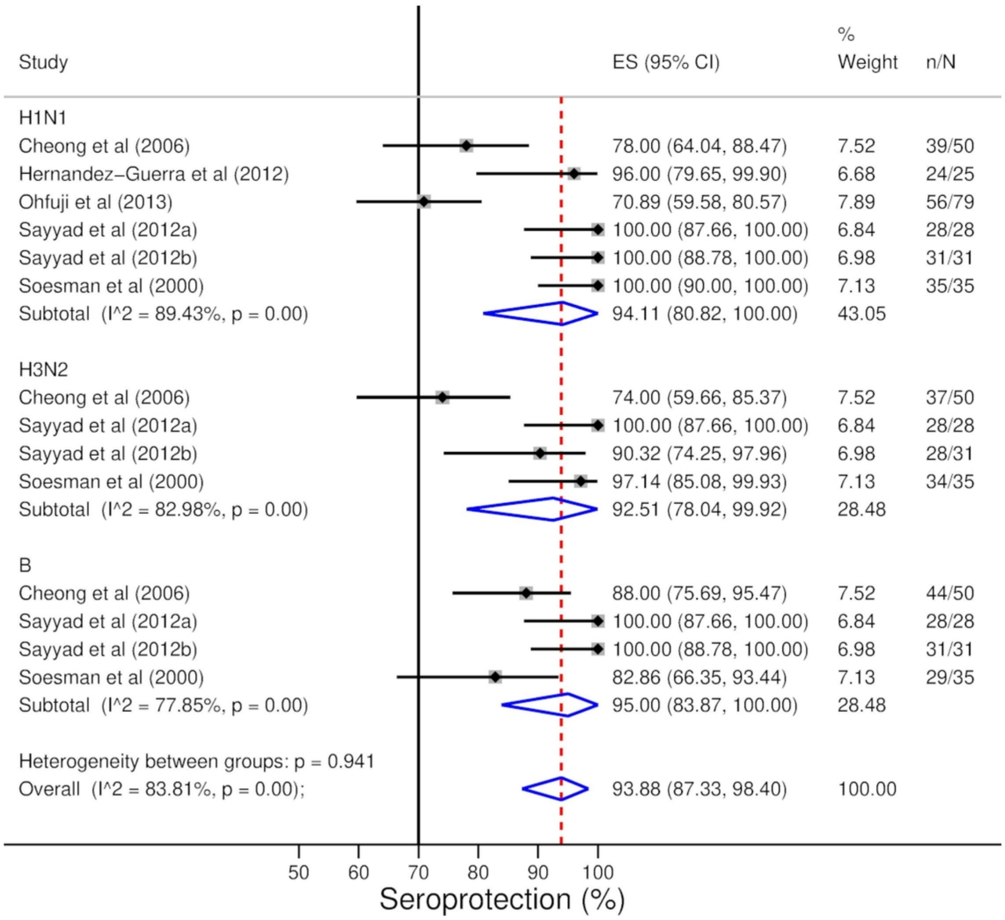

Figure 4. Serological response to influenza vaccination: seroprotection rate after vaccination. Note: Sayyad et al (2012a) includes cirrhotic patients and Sayyad et al (2012b) includes non-cirrhotic patients only.

96x87mm (300 x 300 DPI)

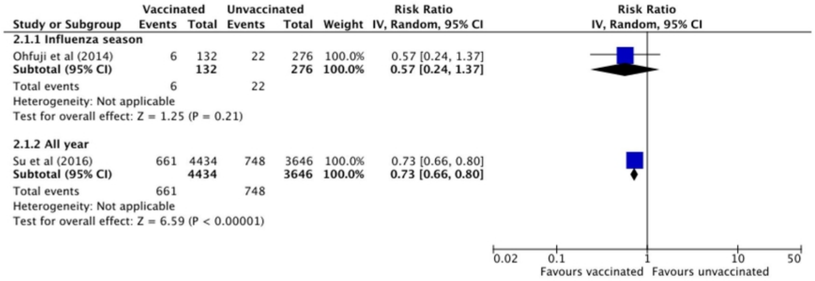

Figure 5. Effect of influenza vaccine on clinical outcomes: all-cause hospitalisation in vaccinated compared to unvaccinated liver disease patients. Note: Ohfuji et al (2014) investigated the effect of pandemic (monovalent) vaccine and Su et al (2016) of seasonal vaccine (against three or more virus subtypes).

90x47mm (300 x 300 DPI)

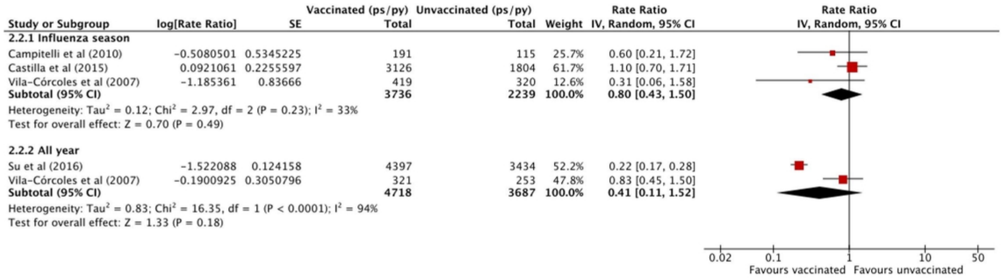

Figure 6. Effect of influenza vaccine on clinical outcomes: all-cause mortality in vaccinated compared to unvaccinated liver disease patients.

96x51mm (300 x 300 DPI)

SUPPLEMENTARY MATERIALS

Table 1. Differences between protocol and review
Table 2. Full MEDLINE (Ovid) search
Table 3. Medical subject headings used in Embase (Ovid), Pubmed and Cochrane Central Register of Controlled Trials
Table 4. Studies excluded due to uncertainty over whether the study included liver disease patients and the number of liver disease patients included
Table 5. HI antibody responses in liver disease patients before and after vaccination
Table 6. Risk of bias in individual studies
Table 7. Risk of selective outcome reporting in the included studies
Table 8. GRADE evidence profile
Figure 1. Seroprotection rate in liver disease patients before influenza vaccination
Figure 2. Sensitivity analysis: Mean difference in log HI antibody GMTs before and after vaccination in cirrhotic patients only
Figure 3. Sensitivity analysis: Seroconversion rate after vaccination in cirrhotic patients only
Figure 4. Sensitivity analysis: Seroprotection rate after vaccination in cirrhotic patients only
Figure 5. Sensitivity analysis: Seroprotection rate before vaccination in cirrhotic patients only
Figure 6. Additional analysis: Mean difference between HI antibody GMT after influenza vaccination in liver disease patients compared to healthy individuals
Figure 7. Additional analysis: Mean difference between geometric mean HI antibody titres before influenza vaccination: liver disease patients compared to healthy individuals
Figure 8. Additional analysis: Mean difference between geometric mean HI antibody titre ratio after influenza vaccination in liver disease patients compared to healthy individuals
Figure 9. Additional analysis: Probability of seroconversion after influenza vaccination in liver disease patients compared to healthy individuals
Figure 10. Additional analysis: Probability of seroprotection after influenza vaccination in liver disease patients compared to healthy individuals
Figure 11. Additional analysis: Difference in seroprotection rate before vaccination in liver disease patients compared to healthy individuals
Table 9. Risk of all-cause mortality in the general population of the included studies where liver patients formed a subgroup
References

For peer review only

Table 1. Differences between protocol and review

Review section	Protocol	Review
**Search strategy**	1. Our planned search terms included filters for adults and study type.<br><br>2. We planned to complement the electronic searches by manually searching the reference lists of included studies for additional references.	1. In the final search, we decided to additionally filter for publication type to increase the specificity of the search.<br><br>2. In the final search, we further complemented the search by manually searching also through the recent WHO, ECDC and CDC influenza vaccine guidelines for additional references.
**Risk of bias assessment and quality of evidence**	1. We planned to use Cochrane Collaboration's tool to assess risk of bias in all studies.<br><br>2. We planned to consider age, sex, severity and aetiology of liver disease as the most important confounders in the assessment of observational studies.	1. In the review, we used Cochrane Collaboration's tool to assess risk of bias in randomized controlled trials and followed their recommendation to use the Newcastle-Ottawa scale to assess risk of bias in observational studies.<br><br>2. In the review, based on the recommendations from the peer-review of the protocol, we additionally considered as potential confounders: chronic co-morbidities and previous seasonal influenza vaccination during the same season as additional confounders in the assessment of studies that investigated the clinical effects of a pandemic (monovalent) vaccine. In assessing the quality of evidence, we considered the lack of clear effect in the influenza season as a sign of imprecision of the effect estimate of clinical outcomes.
**Data analysis**	1. We did not plan to report serological outcome measures categorised by virus subtypes.	1. In the review, we reported the serological outcome measures categorised by virus subtype as there may be differences in antibody response between them.

For peer review only - http://bmjopen.bmj.com/site/about/guidelines.xhtml

Table 2. Full MEDLINE (Ovid) search

Search concept	Search terms
Influenza vaccine	1. exp Influenza Vaccines/
	2. exp Influenza, Human/pc [Prevention & Control]
	3. ((anti?influenza or influenza or seasonal or anti?flu or flu) adj5 (vaccin* or immuni*)).mp.
	4. ((TIV or QIV or trivalent or quadrivalent or 3?valent or 4?valent) adj5 (vaccin* or immuni*)).mp.
	5. 1 or 2 or 3 or 4
Liver disease	6. exp Liver Diseases/
	7. ((liver or hepat*) adj3 disease*).mp.
	8. ("chronic liver" or "chronic hepat*").mp.
	9. cirrho*.mp.
	10. 6 or 7 or 8 or 9
Adult participants	11. exp Adult/
	12. adult.mp.
	13. (middle?aged or aged).sh.
	14. age*.tw.
	15. 11 or 12 or 13 or 14
Randomised controlled trials	16. randomized controlled trial.pt.
	17. randomi*.ab,ti.
	18. randomly.ab,ti.
	19. controlled clinical trial.pt.
	20. trial.ab,ti.
	21. groups.ab,ti.
	22. drug therapy.fs.
	23. placebo.ab,ti.
	24. 16 or 17 or 18 or 19 or 20 or 21 or 22 or 23
	25. (review or "practice guideline" or "patient education handout" or guideline or editorial or "case reports").pt.
	26. 25 not (25 and (16 and 19))
	27. Animals/
	28. Humans/
	29. 27 not (27 and 28)
	30. 24 not (26 or 29)
Case-control and cohort studies	31. Epidemiologic Studies/
	32. exp Case control studies/
	33. exp Cohort studies/
	34. Longitudinal studies/

For peer review only - http://bmjopen.bmj.com/site/about/guidelines.xhtml

	35. Follow up studies/
	36. Prospective studies/
	37. Retrospective studies/
	38. Control groups/
	39. Matched-Pair Analysis/
	40. (Case* adj5 control*).ti,ab,kw.
	41. (Case* adj5 comparison*).ti,ab,kw.
	42. Control group*.ti,ab,kw.
	43. (Cohort adj (study or studies)).ti,ab.
	44. Cohort anal*.ti,ab.
	45. (Follow up adj (study or studies)).ti,ab.
	46. (Observational adj (study or studies)).ti,ab.
	47. Longitudinal.ti,ab.
	48. Retrospective.ti,ab.
	49. Prospective.ti,ab.
	50. 31 or 32 or 33 or 34 or 35 or 36 or 37 or 38 or 39 or 40 or 41 or 42 or 43 or 44 or 45 or 46 or 47 or 48 or 49
	51. (review or "practice guideline" or "patient education handout" or guideline or editorial or "case reports").pt.
	52. 51 not (51 and (31 or 32 or 33 or 34 or 35 or 36 or 37 or 38 or 39))
	53. Animals/
	54. Humans/
	55. 53 not (53 and 54)
	56. 50 not (52 or 55)
	57. 30 or 56
	58. 5 and 10 and 15 and 57
Results: Liver disease-specific search	59. limit 58 to medline
	60. 5 and 15 and 30
Results: Influenza vaccine studies search	61. limit 60 to medline

For peer review only - http://bmjopen.bmj.com/site/about/guidelines.xhtml

Table 3. Medical subject headings used in Embase (Ovid), Pubmed and Cochrane Central Register of Controlled Trials

Database	Medical subject headings				
	**Influenza**	**Liver disease**	**Adults**	**Study type**	**Animal/Human study**
Embase (Ovid)	exp influenza vaccine/<br>exp influenza/pc [Prevention]	exp liver disease/<br>exp liver cirrhosis/	exp adult/	randomized controlled trial/<br>controlled clinical trial/<br>epidemiology/<br>exp case control study/<br>exp cohort analysis/<br>longitudinal study/<br>follow up/<br>prospective study/<br>retrospective study/<br>control group/	exp animal/<br>exp animal cell/<br>exp animal disease/<br>exp animal experiment/<br>exp animal model/<br>exp animal tissue/<br>human/
Pubmed	Influenza Vaccines[MH]<br>"influenza human/prevention and control"[MH]	liver disease[MH]	"adult"[MH]<br>"middle aged"[MH:noexp]<br>"aged"[MH:noexp]	"Epidemiologic Studies"[MH:noexp]<br>"Case-Control Studies"[MH]<br>"retrospective studies"[MH:noexp] "Control Groups"[MH:noexp]<br>"matched-pair analysis"[MH:noexp]<br>"longitudinal studies"[MH:noexp]<br>"follow-up studies"[MH:noexp] "prospective studies"[MH:noexp]<br>"cohort studies"[MH]	"animals"[MH] "humans"[MH]
Cochrane Central Register of Controlled Trials	[mh "Influenza Vaccines"]<br>[mh "Influenza, human"/PC]	[mh "Liver Diseases"]	[mh Adult]	Not relevant	Not relevant

For peer review only

Table 4. Studies excluded due to uncertainty over whether the study included liver disease patients and the number of liver disease patients included

Study	Location	Study design	Review outcomes	Reason for uncertainty and notes
Castilla et al (2012)[1]	Spain	Cohort study	Influenza illness/ILI-related hospitalisations	The inclusion criteria included patients with liver disease. to confirm the actual study population also included liver disease patients we contacted but could not reach the corresponding author.
Castilla et al (2013)[2]	Spain	Cohort study	Influenza illness/ILI-related hospitalisations	The inclusion criteria included patients with liver disease. to confirm the actual study population also included liver disease patients we contacted but could not reach the corresponding author.
DiazGranados et al (2013)[3]	United States	Randomised, controlled trial	Serological response	Study population excluded patients with known or suspected hepatitis B or C infection and patients who abused alcohol but included patients with hepatobiliary disorders: 522 in high dose vaccination group and 257 in normal dose vaccination group. We attempted to contact but could not reach the corresponding author.
Emborg et al (2012)[4]	Denmark	Cohort study	Influenza illness/ILI-related hospitalisations	The inclusion criteria included patients with liver disease. To confirm the actual study population also included liver disease patients we contacted but could not reach the corresponding author.
Mangtani et al (2004)[5]	United Kingdom	Cohort study	Acute respiratory illness-related hospitalization<br><br>Acute respiratory illness-related mortality	The inclusion criteria included patients with liver disease as part of the risk group for influenza complications. We contacted the corresponding author to confirm the actual study population also included liver disease patients. The patient data for the study had only been provided on the level of at-risk individuals not on the level of a single disease and so the author had no access to this information.
Olafsdottir et al (2018)[6]	Iceland	Cohort study	Serological response	The study population was the general population, however, the study data collected included a record of primary biliary cirrhosis. We contacted but could not reach the corresponding author to confirm the actual study population also included liver disease patients.
Simpson et al (2013)[7]	Scotland, United Kingdom	Cohort study	All-cause hospitalisation<br><br>All-cause mortality<br><br>Acute respiratory illness-related hospitalisation (pneumonia & influenza)<br><br>Acute respiratory illness-related mortality (pneumonia & influenza)	Number of patients was not reported. Liver disease patients contributed 3727 person-seasons vaccinated/5377 person-seasons unvaccinated (over 9 seasons) to the study. The study, however, also included under 18 year-olds and ≤14 year-olds contributed 4988 person-seasons vaccinated and 281550 person-seasons unvaccinated to the study. Authors were contacted to confirm the number of patients and to obtain subgroup data, however, they were unable to provide this information. It is likely liver disease was more common among over ≥18 year-olds and so the study potentially included over 400 vaccinated and over 500 unvaccinated adult liver disease patients, however, this could not be confirmed.
Wang et al (2004)[8]	Taiwan	Cohort study	All-cause hospitalisation<br><br>Acute respiratory illness-related hospitalisation (pneumonia & influenza)<br><br>Liver disease complication-related	The inclusion criteria included patients with liver disease as part of the risk group for influenza complications. It seems that the patient data for the study had only been provided on the level of at-risk individuals not on the level of a single diseases and so the authors had no access to this information. We attempted to contact but could not reach the corresponding author to confirm.

For peer review only

			hospitalisation (chronic liver disease and cirrhosis)	
Wang et al (2007)[9]	Taiwan	Cohort study	All-cause mortality<br><br>Acute-respiratory illness-related mortality (pneumonia)<br><br>Liver disease complication-related mortality (chronic liver disease)	The inclusion criteria included patients with liver disease as part of the risk group for influenza complications. It seems that the patient data for the study had only been provided on the level of at-risk individuals not on the level of a single diseases and so the authors had no access to this information. We contacted but could not reach the corresponding author to confirm.
Zivich et al (2017)[10]	United States	Cohort study	All-cause mortality	The study included at least 45 vaccinated and 41 unvaccinated liver disease patients. The study, however, also included under 18 year-old patients (121 were ≤4 year-old and 144 were ≤24 year-old. Authors were contacted to confirm the number of patients and to obtain subgroup data, however, they no longer have access to the study data and were unable to provide this information. It is likely liver disease was more common among over ≥18 year-olds and so the study potentially included over 40 adult liver disease patients in each group. This, however, could not be confirmed.

For peer review only - http://bmjopen.bmj.com/site/about/guidelines.xhtml

For peer review only

Table 5. HI antibody responses in liver disease patients before and after vaccination

Influenza A (H1N1)						
**Study**	**Pre-vaccination GMT (95% CI)**	**Post-vaccination GMT (95% CI)**	**GMTR**	**Seroconversion rate (%)**	**Pre-vaccination seroprotection rate (%)**	**Post-vaccination seroprotection rate (%)**
Cheong et al (2006)[11]	15·6 (SE ±1·8)	114·7 (SE ±4·1)	Not reported	40/50 (80%)	5/50 (10%)	39/50 (79%)
Duchini et al (2001)[12]	20·0 (10.0, 40.0)	61·0 (30·0, 125·0)	3·0 (1·7, 5·4)	6/14 (43%)	Not reported	Not reported
Hernández-Guerra et al (2012)[13]	Not reported	229·0 (55·0, 957·0)	Treated: 43·0 (10·0, 180·0)<br>Untreated: 32·0 (7·0, 137·0)	24/25 (96%)	1/25 (4%)	24/25 (96%)
Ohfuji et al (2013)[14]	8·0 (7·0, 9·0)	226·0 (69·0-743·0)	10·3 (7·2, 14·9)	57/59 (97%)	4/79 (5%)	56/79 (71%)
Sayyad et al (2012)[15]	Cirrhotic: 31·2 (SE ±2·0)<br>HBV carrier: 24·1 (SE ±2·4)	Cirrhotic: 170·7 (SE ±1·6)<br>HBV carrier: 177.7 (SE ±1·8)	Not reported	Cirrhotic: 26/28 (93%)<br>HBV carrier: 28/31 (90%)	Cirrhotic: 19/28 (68%)<br>HBV carrier: 16/31 (52%)	Cirrhotic: 28/28 (100%)<br>HBV carrier: 31/31 (100%)
Soesman et al (2000)[16]	60·0 (37·0, 97·0)	795·0 (484·0, 1303·0)	Not reported	23/35 (66%)	26/36 (72%)	35/35 (100%)
Influenza A (H3N2)						
**Study**	**Pre-vaccination GMT (95% CI)**	**Post-vaccination GMT (95% CI)**	**GMTR**	**Seroconversion rate (%)**	**Pre-vaccination seroprotection rate (%)**	**Post-vaccination seroprotection rate (%)**
Cheong et al (2006)[11]	36·8 (SE ±3·2)	75·7 (SE ±2·6)	Not reported	23/50 (46%)	13/50 (26%)	37/50 (74%)
Duchini et al (2001)[12]	87·0 (37·0, 203.0)	216·0 (95·0, 489·0)	2·4 (1·6, 3·8)	3/14 (21%)	Not reported	Not reported
Sayyad et al (2012)[15]	Cirrhotic: 36·6 (SE ±2·4)<br>HBV carrier: 24·5 (SE ±2·3)	Cirrhotic: 121·5 (SE ±1·9)<br>HBV carrier: 111·1 (SE ±2·4)	Not reported	Cirrhotic: 20/28 (71%)<br>HBV carrier: 22/31 (71%)	Cirrhotic: 20/28 (71%)<br>HBV carrier: 16/31 (52%)	Cirrhotic: 28/28 (100%)<br>HBV carrier: 28/31 (90%)
Soesman et al (2000)[16]	34·0 (19·0, 61·0)	331·0 (207·0, 531·0)	Not reported	21/35 (60%)	21/36 (58%)	34/35 (97%)
Influenza B						
**Study**	**Pre-vaccination GMT (95% CI)**	**Post-vaccination GMT (95% CI)**	**GMTR**	**Seroconversion rate (%)**	**Pre-vaccination seroprotection rate (%)**	**Post-vaccination seroprotection rate (%)**
Cheong et al (2006)[11]	17·4 (SE ±1·7)	82·3 (SE ±2·3)	Not reported	43/50 (86%)	5/50 (10%)	44/50 (88%)
Duchini et al (2001)[12]	18·0 (9·0, 35·0)	119·0 (48·0, 292·0)	6·0 (2·6, 19·0)	9/14 (64%)	Not reported	Not reported
Sayyad et al (2012)[15]	Cirrhotic: 27·1 (SE ±2·1)<br>HBV carrier: 14·9 (SE ±2·1)	Cirrhotic: 210·6 (SE ±1·7)<br>HBV carrier: 134·3 (SE ±2·0)	Not reported	Cirrhotic: 28/28 (100%)<br>HBV carrier: 29/31 (94%)	Cirrhotic: 18/28 (64%)<br>HBV carrier: 6/31 (19%)	Cirrhotic: 28/28 (100%)<br>HBV carrier: 31/31 (100%)

For peer review only

Soesman et al (2000)[16]	12·0 (8·0, 19·0)	110·0 (61·0, 199·0)	Not reported	25/35 (71%)	10/36 (28%)	29/35 (83%)

For peer review only

Table 6. Risk of bias in the included studies

A. Risk of bias in observational studies of serological outcomes

**Study**	**Selection**[a]<br>*** = low risk<br>** = moderate risk<br>- and * = high risk	**Outcome**<br>*** = low risk<br>** = moderate risk<br>- and * = high risk
Cheong et al (2006)	*** Low	* High[b,c]
Duchini et al (2001)	*** Low	** Moderate[c]
Hernández-Guerra et al (2012)	*** Low	*** Low
Ohfuji et al (2013)	*** Low	*** Low
Sayyad et al (2012)	** Moderate[d]	** Moderate[b]
Soesman et al (2000)	** Moderate[e]	** Moderate[c]

[a] Selection was assessed considering the selection of the liver disease group only.
[b] High drop-out rate.
[c] No description of independent blind assessment of outcome.
[d] Source/selection of liver patient groups not clear.
[e] Pre-vaccination influenza infection status not clear and pre-vaccination seroprotection rates high.

B. Risk of bias in observational studies of clinical outcomes

**Study**	**Selection**<br>**** = low risk<br>** and *** = moderate risk<br>- and * = high risk	**Comparability**[a]<br>**** and ***** = low risk<br>** and *** = moderate risk<br>- and * = high risk	**Outcome**<br>*** = low risk<br>** = moderate risk<br>- and * = high risk
Campitelli et al (2010)	*** Moderate[b]	Unclear[c]	*** Low
Castilla et al (2105)	**** Low	Unclear[c]	*** Low
Ohfuji et al (2014)	*** Moderate[b]	**** Moderate[d, e]	*** Low
Su et al (2016)	**** Low	***** Low	*** Low
Vila-Córcoles et al (2007)	**** Low	Unclear[c]	*** Low

[a] Comparability was considered between vaccinated and unvaccinated groups. Confounders considered were: age, sex, liver disease aetiology, liver disease severity, chronic co-morbidities and previous seasonal influenza vaccination in the same season if study vaccine pandemic vaccine
[b] Self-reported vaccination (although in Campitelli et al 2010, vaccination was ascertained from physician billing claims, these are based on self-reporting).
[c] No information on characteristics of the vaccinated and unvaccinated groups.
[d] Sex not comparable between groups.
[e] Seasonal influenza vaccination in the same season than pandemic vaccine given to some but balance unclear between groups.

C. Risk of bias in randomised controlled studies

**Study**	**Random sequence generation**	**Allocation concealment**	**Blinding of participants and personnel**	**Blinding of outcome assessment**	**Incomplete outcome data**	**Selective reporting**	**Other bias**
Song et al (2007)	Low	Low	High[a]	Low	High[b]	Low	Unclear[c]

[a] Patients were not blinded and could not have been blinded when only the intervention group received the vaccination.
[b] High drop-out rate.
[c] Details on study funding and conflicts of interest were not provided.

For peer review only - http://bmjopen.bmj.com/site/about/guidelines.xhtml

Table 7. Risk of selective outcome reporting in the included studies

Risk of bias arising from the lack of inclusion of results when a study was excluded from a meta-analysis or not fully reported in a review because the data were unavailable.

Level of reporting: √ = Full reporting of results, × = No reporting of results, o = Partial reporting of results

Classifications and descriptions of risk of bias found in the assessment of missing or complete outcome reporting:

Clear that the outcome was measured and analysed:	Level of reporting	Risk of bias
C = Trial report states that outcome was analysed but insufficient data were presented for the trial to be included in meta-analysis or to be considered to be fully tabulated.	o	Low Risk
Clear that the outcome was measured:		
E = Clear that the outcome was measured. Judgment says outcome likely to have been analysed but not reported because of non-significant results.	×	High Risk
Unclear whether the outcome was measured:		
G = Not mentioned but clinical judgment says likely to have been measured and analysed but not reported on the basis of non-significant results.	×	High Risk
H = Not mentioned but clinical judgment says unlikely to have been measured at all.	×	Low Risk
Clear that the outcome was not measured:		
I = Clear that the outcome was not measured.	×	No Risk

For peer review only

A. Risk of selective outcome reporting in studies of serological outcomes

	**Review secondary outcomes**				**Other outcomes reported**
**Study**	**GMT**	**GMTR**	**Seroprotection %**	**Seroconversion %**	
Cheong et al (2006)	√	×<br>E – High risk[a]	o<br>C – Low risk[b]	o<br>C – Low risk[b]	Cellular immune response to vaccine (interferon-γ secretion)
Duchini et al (2001)	√	√	×<br>E – High risk[c]	√	None
Hernández-Guerra et al (2012)	o<br>C – Low risk[d]	√	o<br>C – Low risk[e]	√	Systemic adverse events, acceptance and tolerance of vaccination
Ohfuji et al (2013)	√	√	√	√	Serious adverse events
Sayyad et al (2012)	√	×<br>E – High risk[a]	o<br>C – Low risk[f]	o<br>C – Low risk[f]	None
Soesman et al (2000)	√	×<br>E – High risk[a]	√	√	Serological response (HI antibody) to a non-vaccine strain

[a] Pre- and post-vaccination GMT was measured and it is likely that their ratio was analysed but not reported.
[b] Only percentages were reported so we have assumed the highest possible number of individuals closest to the reported percentage for pre-vaccination measures and lowest possible number of individuals closest to the reported percentage for post-vaccination measures.
[c] Pre- and post-vaccination GMT was measured and seroconversion rate was analysed. It is likely that the seroprotection rate was also analysed but not reported.
[d] Pre-vaccination GMT was measured but not fully reported. It is likely this was low as only one individual had 1:40 antibody level (seroprotective level).
[e] Pre-vaccination seroprotection rate was not fully reported but the rate was low only one individual had a seroprotective level of antibodies. It was not specified in which comparison group so we have assumed it was one individual in each group.
[f] The percentages and numbers of individuals did not add up in the reported results so we have assumed the highest possible number of individuals closest to the reported percentage for pre-vaccination measures and lowest possible number of individuals closest to the reported percentage for post-vaccination measures.

For peer review only

For peer review only - http://bmjopen.bmj.com/site/about/guidelines.xhtml

B. Risk of selective outcome reporting in studies of clinical outcomes

	**Review primary outcomes**		**Review secondary outcomes**						**Other outcomes reported**
**Study**	**All-cause hospitalisation**	**All-cause mortality**	**Acute respiratory illness-related hospitalisation**	**Influenza illness/ILI-related hospitalisation**	**Liver disease complication related-hospitalisation**	**Acute respiratory illness-related mortality**	**Influenza illness/ILI-related mortality**	**Liver disease complication related-mortality**	
Campitelli et al (2010)	× H – Low risk[a]	√	o C – Low risk[b]	× I – No risk	× I – No risk	× I – No risk	× I – No risk	× I – No risk	None
Castilla et al (2105)	× I – No risk	√	× I – No risk	× I – No risk	× I – No risk	× I – No risk	× I – No risk	× I – No risk	ILI incidence
Ohfuji et al (2014)	√	× I – No risk	× I – No risk	× I – No risk	× I – No risk	× I – No risk	× I – No risk	× I – No risk	None
Song et al (2007)	× I – No risk	× G – High risk[c]	× I – No risk	× I – No risk	× I – No risk	× I – No risk	√	× I – No risk	ILI incidence, clinical manifestations of ILI, liver disease complications secondary to influenza
Su et al (2016)	√	√	× I – No risk	× I – No risk	√	× I – No risk	× I – No risk	× I – No risk	Pneumonia & Influenza incidence, heart disease related hospitalisation, respiratory failure, intensive care unit admission
Vila-Córcoles et al (2007)	× I – No risk	√	× I – No risk	× I – No risk	× I – No risk	× I – No risk	× I – No risk	× I – No risk	ILI incidence

[a] The main interest in the study was all-cause mortality and cause-specific hospitalisation outcome was used to understand the whether the mortality may have been linked to influenza. Accessing the data required an approved study protocol and it is unlikely that data to study all-cause hospitalisation was available when not included in the protocol.

[b] Study authors were unable to provide exact event numbers for this outcome as their institution's privacy policy does not allow providing data in case of five or fewer events.

[c] Authors did measure influenza-related mortality in this trial and it is likely that they also measured all-cause mortality. Authors were contacted but we were unable to obtain a response.

For peer review only

Table 8. GRADE evidence profile

Effectiveness of influenza vaccination in preventing all-cause hospitalisation and all-cause mortality in adults with chronic liver disease

**Number of studies (design)**	**Limitations**	**Inconsistency**	**Indirectness**	**Imprecision**	**Publication bias**	**No vaccine**	**Vaccine**	**Relative risk/ Rate ratio**	**Control risk/ Control rate**	**Risk difference**	**Quality**
All-cause hospitalisation, influenza season											
1 (Cohort study)	Serious limitations[a]	Not judged (only 1 study)	No serious indirectness	Serious imprecision[b]	Suspected[c]	22/276	6/132	0·57 (0·24, 1.37)	80 per 1000	-0·03 (-0·08, 0·01)	Very low
All-cause hospitalisation, all year											
1 (Cohort study)	No serious limitations	Not judged (only 1 study)	No serious indirectness	No serious imprecision	Suspected[c]	748/3646	661/4434	0·73 (0·66, 0·80)	205 per 1000	-0·06 (-0·07- 0·04)	Very low
All-cause mortality, influenza season											
3 (Cohort study)	Serious limitations[d]	Serious inconsistency[e]	No serious indirectness	Serious imprecision[f]	Suspected[c]	42/2239 person-seasons	66/3736 person-seasons	0·80 (0·43, 1·50)	17 per 1000 person-seasons	-0·22 (-0·85, 0·41)	Very low
All-cause mortality, all year											
2 (Cohort study)	Serious limitations[g]	No serious inconsistency	No serious indirectness	Serious imprecision[e,f]	Suspected[c]	318/3687 person-years	105/4718 person-years	0·41 (0·11, 1·52)	85 per 1000 person-years	-0·89 (-2·19, 0·42)	Very low

[a] Moderate risk of bias in selection and comparability of groups.
[b] Imprecision of the estimate with large 95% CIs.
[c] Due to study language restrictions in the review and exclusion of studies due to uncertainty of eligibility.
[d] Moderate risk of bias in selection in one study and unclear bias in comparability of groups in all studies.
[e] Study effect estimates varied largely.
[f] Control risks varied largely between studies.
[g] Unclear bias in comparability of groups in one study and uncertain timing of the vaccine effect due to Kaplan-Meier plot showing increasing difference in mortality rates outside the influenza season.

For peer review only - http://bmjopen.bmj.com/site/about/guidelines.xhtml

Figure 1. Seroprotection rate in liver disease patients before influenza vaccination

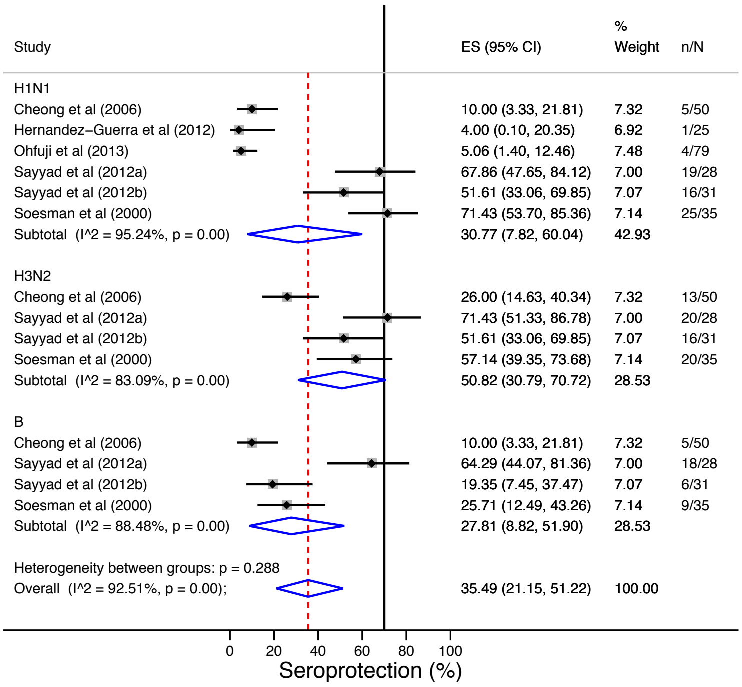

Figure 2. Sensitivity analysis: Mean difference in log HI antibody GMTs before and after vaccination in cirrhotic patients only

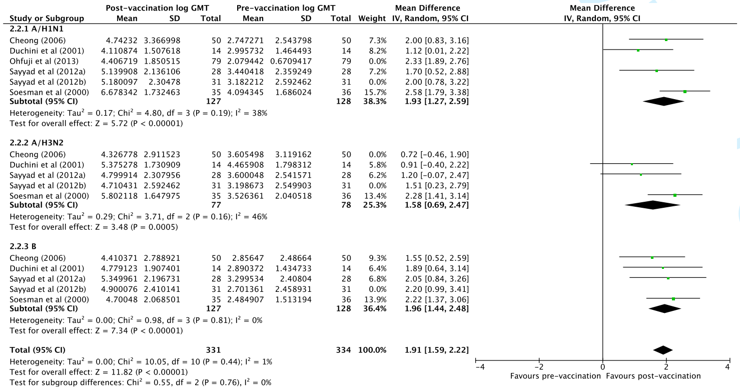

Note: Liver patient results from the studies with non-cirrhotic patients only and studies with a mix of cirrhotic and non-cirrhotic patients are visible but do not affect the outcome of analysis. Only the results from the studies with cirrhotic patients Cheong et al (2006), Duchini et al (2001) Sayyad et al (2012a) and Soesman are pooled in this analysis. Sayyad et al (2012b) includes non-cirrhotic patients only.

For peer review only - http://bmjopen.bmj.com/site/about/guidelines.xhtml

Figure 3. Sensitivity analysis: Seroconversion rate after vaccination in cirrhotic patients only

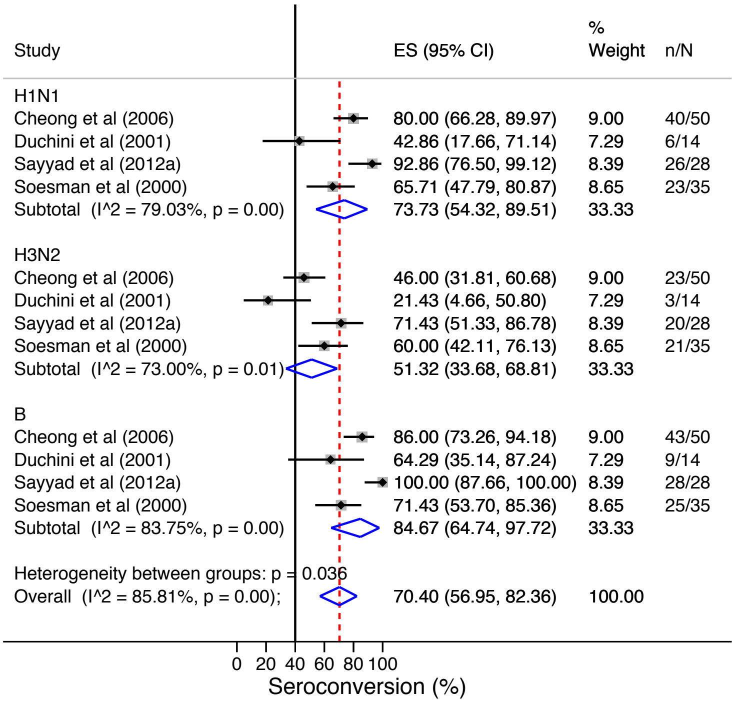

Figure 4. Sensitivity analysis: Seroprotection rate after vaccination in cirrhotic patients only

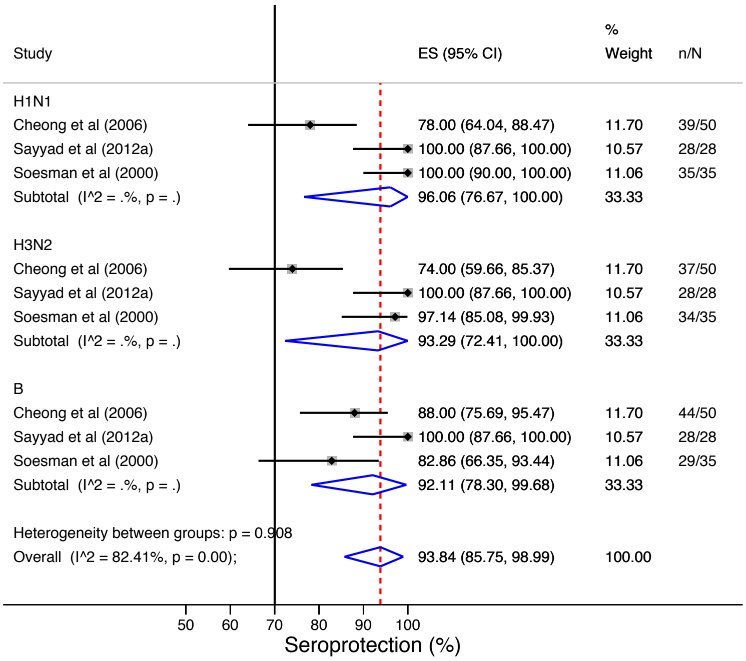

For peer review only - http://bmjopen.bmj.com/site/about/guidelines.xhtml

Figure 5. Sensitivity analysis: Seroprotection rate before vaccination in cirrhotic patients only

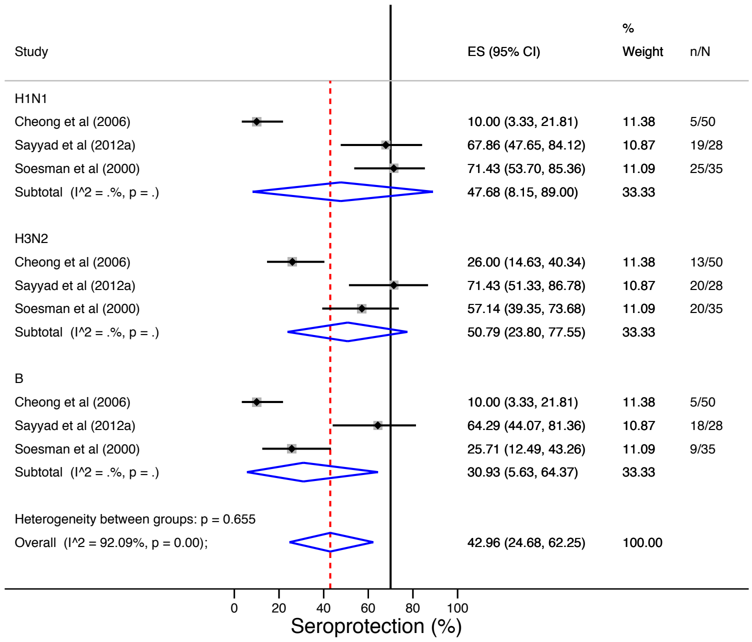

Figure 6. Additional analysis: Mean difference between HI antibody GMT after influenza vaccination in liver disease patients compared to healthy individuals

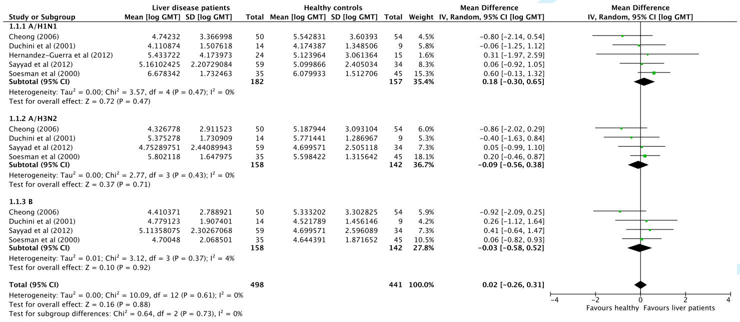

Note: Results from the non-cirrhotic and cirrhotic patient groups in Sayyad et al (2012) have been combined using the formulae recommended in Cochrane Handbook [17].

Figure 7. Additional analysis: Mean difference between geometric mean HI antibody titres before influenza vaccination in liver disease patients compared to healthy individuals

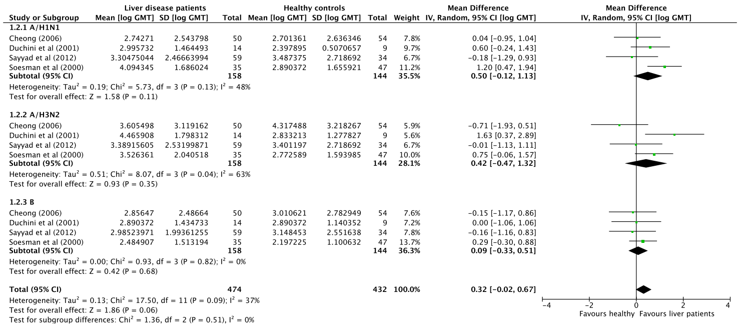

Note: Results from the non-cirrhotic and cirrhotic patient groups in Sayyad et al (2012) have been combined using the formulae recommended in Cochrane Handbook [17].

For peer review only - http://bmjopen.bmj.com/site/about/guidelines.xhtml

Figure 8. Additional analysis: Mean difference between geometric mean HI antibody titre ratio after influenza vaccination in liver disease patients compared to healthy individuals

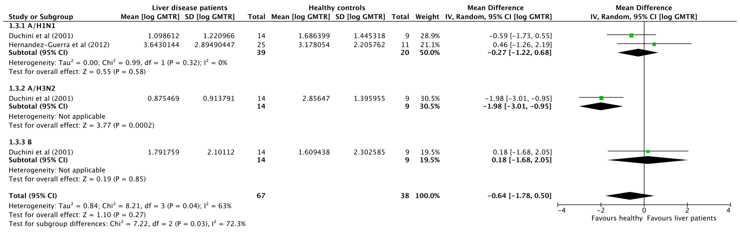

Note: Results from the non-cirrhotic and cirrhotic patient groups in Sayyad et al (2012) have been combined using the formulae recommended in Cochrane Handbook [17].

Figure 9. Additional analysis: Probability of seroconversion after influenza vaccination in liver disease patients compared to healthy individuals

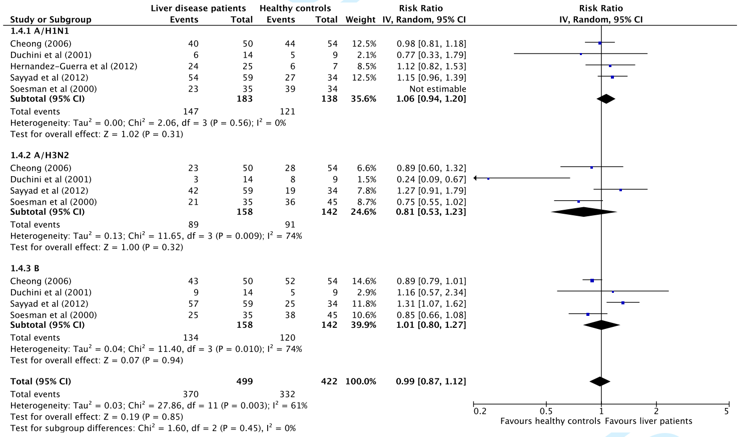

Note: Results from the non-cirrhotic and cirrhotic patient groups in Sayyad et al (2012) have been combined using the formulae recommended in Cochrane Handbook [17].

Figure 10. Additional analysis: Probability of seroprotection after influenza vaccination in liver disease patients compared to healthy individuals

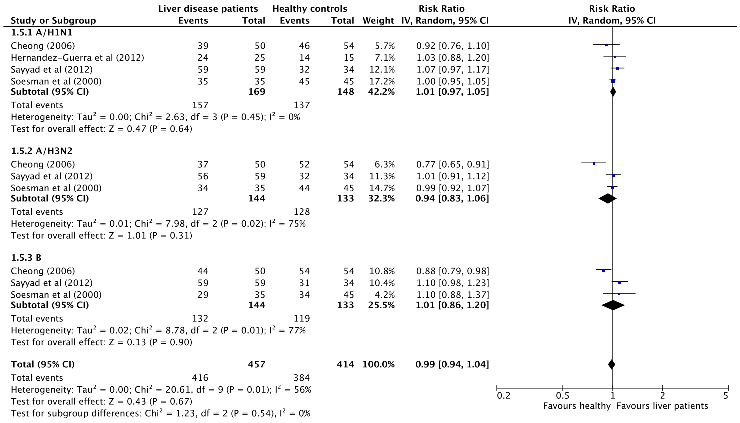

Note: Results from the non-cirrhotic and cirrhotic patient groups in Sayyad et al (2012) have been combined using the formulae recommended in Cochrane Handbook [17].

For peer review only - http://bmjopen.bmj.com/site/about/guidelines.xhtml

Figure 11. Additional analysis: Difference in seroprotection rate before vaccination in liver disease patients compared to healthy individuals

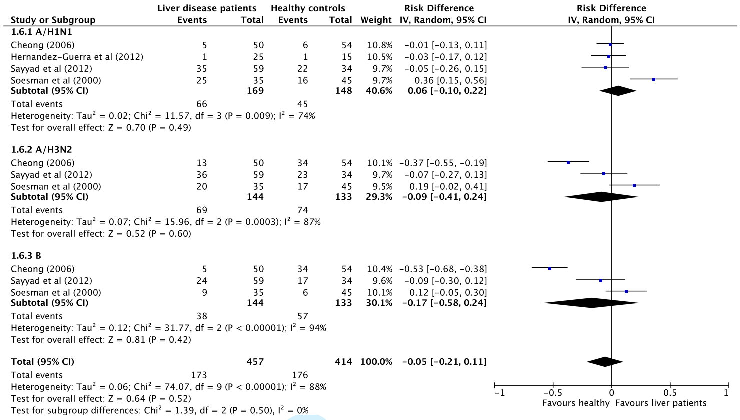

Note: Results from the non-cirrhotic and cirrhotic patient groups in Sayyad et al (2012) have been combined using the formulae recommended in Cochrane Handbook [17].

Table 9. Risk of all-cause mortality in the general population of the included studies where liver patients formed a subgroup

Study	Number of vaccinated/unvaccinated	Adjusted HR (95%CI) in the influenza season	Notes
Campitelli et al (2010)	14512/11410	0·61 (0·47–0·79)	Protective effect was present also outside the influenza season indicating presence of frailty bias.
Castilla et al (2015)	60400/42756[a]	0·84 (0·76–0·93)	Effect outside the seasons was not protective
Vila-Córcoles et al (2007	6051/5189	0·63 (0·54–0·74)	Effect outside the seasons was not protective

[a] These numbers are for the first of the two seasons studied. Number of patients and the % of vaccinated were similar for the second season.

For peer review only - http://bmjopen.bmj.com/site/about/guidelines.xhtml

References

1 Castilla J, Martinez-Artola V, Salcedo E, *et al*. Vaccine effectiveness in preventing influenza hospitalizations in Navarre, Spain, 2010-2011: Cohort and case-control study. Vaccine. 2012;**30**:195–200.

2 Castilla J, Godoy P, Dominguez A, *et al*. Influenza vaccine effectiveness in preventing outpatient, inpatient, and severe cases of laboratory-confirmed influenza. Clin. Infect. Dis. 2013;**57**:167–75. doi:10.1093/cid/cit194

3 DiazGranados CA, Dunning AJ, Jordanov E, *et al*. High-dose trivalent influenza vaccine compared to standard dose vaccine in elderly adults: Safety, immunogenicity and relative efficacy during the 2009-2010 season. *Vaccine* 2013;**31**:861–6. doi:http://dx.doi.org/10.1016/j.vaccine.2012.12.013

4 Emborg H-D, Krause TG, Hviid A, *et al*. Effectiveness of vaccine against pandemic influenza A/H1N1 among people with underlying chronic diseases: Cohort study, Denmark, 2009-10. BMJ. 2012;**344**:d7901. doi:https://doi.org/10.1136/bmj.d7901

5 Mangtani P, Cumberland P, Hodgson CR, *et al*. A cohort study of the effectiveness of influenza vaccine in older people, performed using the United Kingdom general practice research database. J. Infect. Dis. 2004;**190**:1–10.

6 Olafsdottir TA, Alexandersson KF, Sveinbjornsson G, *et al*. Age and Influenza-Specific Pre-Vaccination Antibodies Strongly Affect Influenza Vaccine Responses in the Icelandic Population whereas Disease and Medication Have Small Effects. *Front Immunol* 2017;**8**:1872. doi:10.3389/fimmu.2017.01872

7 Simpson C, Lone N, Kavanagh K, *et al*. Seasonal Influenza Vaccine Effectiveness (SIVE): an observational retrospective cohort study - exploitation of a unique community-based national-linked database to determine the effectiveness of the seasonal trivalent influenza vaccine (Structured abstrac. *Heal Technol Assess Database* 2013;**1**.

8 Wang CS, Wang ST, Lai Lee CT, *et al*. Reducing major cause-specific hospitalization rates and shortening hospital stays after influenza vaccination. *Clin Infect Dis* 2004;**39**:1604–10. doi:http://dx.doi.org/10.1086/425323

9 Wang CS, Wang ST, Lai CT, *et al*. Impact of influenza vaccination on major cause-specific mortal. Vaccine. 2007;**25**:1196–203.

10 Zivich PN, Tatham L, Lung K, *et al*. Influenza vaccination status and outcomes among influenza-associated hospitalizations in Columbus, Ohio (2012-2015). *Epidemiol Infect* 2017;**145**:3284–93. doi:10.1017/S0950268817002163

11 Cheong H-J, Song J-Y, Park J-W, *et al*. Humoral and cellular immune responses to influenza vaccine in patients with advanced cirrhosis. *Vaccine* 2006;**24**:2417–22. doi:10.1016/j.vaccine.2005.11.064

12 Duchini A, Hendry RM, Nyberg LM, *et al*. Immune response to influenza vaccine in adult liver transplant recipients. *Liver Transplant* 2001;**7**:311–3. doi:http://dx.doi.org/10.1053/jlts.2001.23010

13 Hernandez-Guerra M, Gonzalez-Mendez Y, de Molina P, *et al*. Immunogenicity and Acceptance of Influenza A (H1N1) Vaccine in a Cohort of Chronic Hepatitis C Patients Receiving Pegylated-Interferon Treatment. *PLoS One* 2012;**7**:e48610. doi:http://dx.doi.org/10.1371/journal.pone.0048610

14 Ohfuji S, Fukushima W, Tamori A, *et al*. Immunogenicity of influenza A(H1N1)pdm09 vaccine and the associated factors on lowered immune response in patients with hepatitis C. *Influenza Other Respi Viruses* 2013;**7**:456–65. doi:10.1111/j.1750-2659.2012.00424.x

15 Sayyad B, Alavian SM, Najafi F, *et al*. Efficacy of Influenza Vaccination in Patients with Cirrhosis and Inactive Carriers of Hepatitis B Virus Infection. *Iran Red Crescent Med J* 2012;**14**:623–30.

16 Soesman NMR, Rimmelzwaan GF, Nieuwkoop NJ, *et al*. Efficacy of influenza vaccination in adult liver transplant recipients. *J Med Virol* 2000;**61**:85–93. doi:https://doi.org/10.1002/(SICI)1096-9071(200005)61:1<85::AID-JMV14>3.0.CO;2-H

17 The Cochrane Collaboration. Cochrane Handbook for Systematic Reviews of Interventions. Version 5.1.0 [updated March 2011]. 2011.http://handbook-5-1.cochrane.org/ (accessed 25 Jul 2018).

For peer review only - http://bmjopen.bmj.com/site/about/guidelines.xhtml

PRISMA 2009 Checklist

Section/topic	#	Checklist item	Reported on page #
**TITLE**			
Title	1	Identify the report as a systematic review, meta-analysis, or both.	p. 1
**ABSTRACT**			
Structured summary	2	Provide a structured summary including, as applicable: background; objectives; data sources; study eligibility criteria, participants, and interventions; study appraisal and synthesis methods; results; limitations; conclusions and implications of key findings; systematic review registration number.	p. 2-3
**INTRODUCTION**			
Rationale	3	Describe the rationale for the review in the context of what is already known.	p. 5-7
Objectives	4	Provide an explicit statement of questions being addressed with reference to participants, interventions, comparisons, outcomes, and study design (PICOS).	p. 6-7
**METHODS**			
Protocol and registration	5	Indicate if a review protocol exists, if and where it can be accessed (e.g., Web address), and, if available, provide registration information including registration number.	p. 12
Eligibility criteria	6	Specify study characteristics (e.g., PICOS, length of follow-up) and report characteristics (e.g., years considered, language, publication status) used as criteria for eligibility, giving rationale.	p. 7-8
Information sources	7	Describe all information sources (e.g., databases with dates of coverage, contact with study authors to identify additional studies) in the search and date last searched.	p. 8
Search	8	Present full electronic search strategy for at least one database, including any limits used, such that it could be repeated.	p. 8, Supplementary materials p.3-5 (Tables 2 and 3)
Study selection	9	State the process for selecting studies (i.e., screening, eligibility, included in systematic review, and, if applicable, included in the meta-analysis).	p. 9
Data collection process	10	Describe method of data extraction from reports (e.g., piloted forms, independently, in duplicate) and any processes for obtaining and confirming data from investigators.	p. 9-10
Data items	11	List and define all variables for which data were sought (e.g., PICOS, funding sources) and any assumptions and simplifications made.	p. 9-10
Risk of bias in individual studies	12	Describe methods used for assessing risk of bias of individual studies (including specification of whether this was done at the study or outcome level), and how this information is to be used in any data synthesis.	p. 10

For peer review only

For peer review only - http://bmjopen.bmj.com/site/about/guidelines.xhtml

PRISMA 2009 Checklist

Summary measures	13	State the principal summary measures (e.g., risk ratio, difference in means).	p. 10-11
Synthesis of results	14	Describe the methods of handling data and combining results of studies, if done, including measures of consistency (e.g., I^2) for each meta-analysis.	p. 10-11

Section/topic	#	Checklist item	Reported on page #
Risk of bias across studies	15	Specify any assessment of risk of bias that may affect the cumulative evidence (e.g., publication bias, selective reporting within studies).	p. 12
Additional analyses	16	Describe methods of additional analyses (e.g., sensitivity or subgroup analyses, meta-regression), if done, indicating which were pre-specified.	p. 11
**RESULTS**			
Study selection	17	Give numbers of studies screened, assessed for eligibility, and included in the review, with reasons for exclusions at each stage, ideally with a flow diagram.	p. 13 (Figure 1)
Study characteristics	18	For each study, present characteristics for which data were extracted (e.g., study size, PICOS, follow-up period) and provide the citations.	p. 13-18 (Tables 1 and 2)
Risk of bias within studies	19	Present data on risk of bias of each study and, if available, any outcome level assessment (see item 12).	p. 13-14, Supplementary materials p. 9 (Table 6)
Results of individual studies	20	For all outcomes considered (benefits or harms), present, for each study: (a) simple summary data for each intervention group (b) effect estimates and confidence intervals, ideally with a forest plot.	p. 14-18, (Figures 2 and 3), Supplementary materials p.14 (Figure 1)
Synthesis of results	21	Present results of each meta-analysis done, including confidence intervals and measures of consistency.	p. 14-18 (Figures 2 and 3), Supplementary materials p.14 (Figure 1)
Risk of bias across studies	22	Present results of any assessment of risk of bias across studies (see Item 15).	p. 13-14,

For peer review only - http://bmjopen.bmj.com/site/about/guidelines.xhtml

PRISMA 2009 Checklist

			Supplementary materials p.10-13 (Tables 7 and 8)
Additional analysis	23	Give results of additional analyses, if done (e.g., sensitivity or subgroup analyses, meta-regression [see Item 16]).	p. 19, Supplementary materials p. 14-18 (Figures 2-11)
**DISCUSSION**			
Summary of evidence	24	Summarize the main findings including the strength of evidence for each main outcome; consider their relevance to key groups (e.g., healthcare providers, users, and policy makers).	p. 20 (Table 3), Supplementary materials p.13 (Table 8)
Limitations	25	Discuss limitations at study and outcome level (e.g., risk of bias), and at review-level (e.g., incomplete retrieval of identified research, reporting bias).	p. 20-22
Conclusions	26	Provide a general interpretation of the results in the context of other evidence, and implications for future research.	p. 23
**FUNDING**			
Funding	27	Describe sources of funding for the systematic review and other support (e.g., supply of data); role of funders for the systematic review.	p. 12-13, 24

From: Moher D, Liberati A, Tetzlaff J, Altman DG, The PRISMA Group (2009). Preferred Reporting Items for Systematic Reviews and Meta-Analyses: The PRISMA Statement. PLoS Med 6(7): e1000097. doi:10.1371/journal.pmed1000097

For more information, visit: **www.prisma-statement.org**.

For peer review only

MOOSE CHECKLIST *

Source: https://www.editorialmanager.com/jognn/account/MOOSE.pdf
*Modified from Stroup DF, Berlin JA, Morton SC, Olkin I, Williamson GD, Rennie D, et al. Meta-analysis of observational studies in epidemiology: a proposal for reporting. Meta-analysis Of Observational Studies in Epidemiology (MOOSE) group. JAMA 2000;283:2008–12. Copyrighted © 2000, American Medical Association. All rights reserved.

**Title**	Identify the study as a meta-analysis (or systematic review)	p. 1
**Abstract**	Use the journal's structured format	p. 2-3
**Introduction**	**Present:**	
	The clinical problem	p. 5-7
	The hypothesis	p. 6-7
	A statement of objectives that includes the study population, the condition of interest, the exposure or intervention, and the outcome(s) considered	p. 6-7
**Sources**	**Describe:**	
	Qualifications of searchers (eg, librarians and investigators)	p. 24
	Search strategy, including time period included in the synthesis and keywords	p. 8, Supplementary materials p.3-5 (Tables 2 and 3)
	Effort to include all available studies, including contact with authors	p. 8-9
	Databases and registries searched	p. 8
	Search software used, name and version, including special features used (eg, explosion)	p. 8, Supplementary materials p.3-5 (Tables 2 and 3)
	Use of hand searching (eg, reference lists of obtained articles)	p. 8
	List of citations located and those excluded, including justification	p. 13-14 (Figure 1, Tables 1 and 2), Supplementary materials p. 6-7 (Table 4)
	Method of addressing articles published in languages other than English	p. 7

For peer review only

	Method of handling abstracts and unpublished studies	p. 7
	Description of any contact with authors	p. 8-9
**Study selection**	**Describe:**	
	Types of study designs considered	p. 7
	Relevance or appropriateness of studies gathered for assessing the hypothesis to be tested	p. 7-8
	Rationale for the selection and coding of data (eg, sound clinical principles or convenience)	p. 7-11
	Documentation of how data were classified and coded (eg, multiple raters, blinding, and interrater reliability)	p. 9-10
	Assessment of confounding (eg, comparability of cases and controls in studies where appropriate	p. 10-12
	Assessment of study quality, including blinding of quality assessors; stratification or regression on possible predictors of study results	p. 10-12
	Assessment of heterogeneity	p. 11
	Statistical methods (eg, complete description of fixed or random effects models, justification of whether the chosen models account for predictors of study results, dose-response models, or cumulative meta-analysis) in sufficient detail to be replicated	p. 10-11
**Results**	**Present:**	
	A graph summarizing individual study estimates and the overall estimate	p. 14-19 (Figures 2 and 3), Supplementary materials p.14 (Figure 1)
	A table giving descriptive information for each included study	p. 13-18 (Tables 1 and 2)
	Results of sensitivity testing (eg, subgroup analysis)	p. 19, Supplementary

For peer review only

		materials p.14-18 (Figures 2-11)
	Indication of statistical uncertainty of findings	p. 14-19 (Figures 2 and 3), Supplementary materials p.14-18 (Figures 1-11)
**Discussion**	**Discuss:**	
	Strengths and weaknesses	p. 20-22
	Potential biases in the review process (eg, publication bias)	p. 20-22, Supplementary materials p. 2, 10-13 (Tables 1, 7 and 8)
	Justification for exclusion (eg, exclusion of non–English-language citations)	p. 22
	Assessment of quality of included studies	p. 20 (Table 3), Supplementary materials p.13 (Table 8)
	Consideration of alternative explanations for observed results	p. 20
	Generalization of the conclusions (ie, appropriate for the data presented and within the domain of the literature review)	p. 20-23
	Guidelines for future research	p. 23
	Disclosure of funding source	p. 12-13, 24

For peer review only

BMJ Open

The effectiveness of influenza vaccines in adults with chronic liver disease: a systematic review and meta-analysis

Journal:	*BMJ Open*
Manuscript ID	bmjopen-2019-031070.R1
Article Type:	Research
Date Submitted by the Author:	23-Jul-2019
Complete List of Authors:	Härmälä, Suvi; University College London, Institute of Health Informatics<br>Parisinos, Constantinos; University College London, Institute of Health Informatics<br>Shallcross, Laura; University College London, Institute of Health Informatics<br>O'Brien, Alastair; UCL, Division of Medicine<br>Hayward, Andrew; University College London, Institute of Epidemiology and Health Care
**Primary Subject Heading**:	Gastroenterology and hepatology
Secondary Subject Heading:	Infectious diseases, Public health
Keywords:	Cirrhosis, Vaccine effectiveness, Hospitalisation, Seroprotection, Liver disease, Influenza vaccine

SCHOLARONE™
Manuscripts

Title: The effectiveness of influenza vaccines in adults with chronic liver disease: a systematic review and meta-analysis

Authors:

Suvi Härmälä, Institute of Health Informatics, University College London, 222 Euston Road, London NW1 2DA, United Kingdom. Email: suvi.harmala.14@ucl.ac.uk

Constantinos A. Parisinos, Institute of Health Informatics, University College London, 222 Euston Road, London NW1 2DA, United Kingdom. Email: c.parisinos@ucl.ac.uk

Laura Shallcross, Institute of Health Informatics, University College London, 222 Euston Road, London NW1 2DA, United Kingdom. Email: l.shallcross@ucl.ac.uk

Alastair O'Brien, Division of Medicine, University College London, Rayne Building, 5 University Street, London WC1E 6JJ, United Kingdom. Email: a.o'brien@ucl.ac.uk

Andrew Hayward, Institute of Epidemiology and Health Care, University College London, 1-19 Torrington Place, London WC1E 7HB, United Kingdom. Email: a.hayward@ucl.ac.uk

Correspondence to: Suvi Härmälä, Institute of Health Informatics, University College London, 222 Euston Road, London NW1 2DA, United Kingdom. Tel: +44 (0) 782 430 3142. Email: suvi.harmala.14@ucl.ac.uk

For peer review only

For peer review only - http://bmjopen.bmj.com/site/about/guidelines.xhtml

ABSTRACT

Objectives: Patients with liver disease frequently require hospitalisation with infection often the trigger. Influenza vaccination is an effective infection prevention strategy in healthy and elderly but is often perceived less beneficial in liver patients. We investigated whether influenza vaccination triggered serological response and prevented hospitalisation and death in liver disease.

Design: Systematic review and meta-analysis.

Data sources: MEDLINE, EMBASE, Pubmed and CENTRAL up to January 2019.

Eligibility criteria: Randomised or observational studies of the effects of influenza vaccine in adults with liver disease.

Data extraction and synthesis: Two reviewers screened studies, extracted data and assessed risk of bias and quality of evidence. Primary outcomes were all-cause hospitalisation and mortality. Secondary outcomes were cause-specific hospitalisation and mortality, and serological vaccine response. Random effects meta-analysis was used to estimate pooled effects of vaccination.

Results: We found 10649 unique records, 286 were eligible for full-text review and 12 were included. Most patients had viral liver disease. All studies were of very low quality. Both non-cirrhotic and cirrhotic patients mounted an antibody response and influenza vaccination was associated with a reduction in risk of hospital admission from

For peer review only

205/1000 to 149/1000 (risk difference -0.06, 95% CI -0.07, -0.04) in patients with viral liver disease. Vaccinated patients were 27% less likely to be admitted to hospital compared to unvaccinated patients (risk ratio 0.73, 95% CI 0.66, 0.80). No effect against all-cause or cause-specific mortality or cause-specific hospitalisation was found.

Conclusions: The low quantity and quality of the evidence means that the protective vaccine effect may be uncertain. Considering the high risk of serious health outcomes from influenza infection in liver patients and the safety and low cost of vaccination, overall, the potential benefits of seasonal vaccination both to patients and the healthcare systems are likely to outweigh the undesirable consequences of severe influenza infection.

PROSPERO: CRD42017067277

Keywords: cirrhosis, hospitalisation, influenza vaccine, liver disease, seroprotection, vaccine effectiveness

Abbreviations: CI, confidence interval; GMT, geometric mean titre; GMTR, geometric mean titre ratio; GRADE, Grading of Recommendations Assessment, Development and Evaluation; HBV, hepatitis B virus; HCV, hepatitis C virus; HI, hemagglutinisation-inhibition; ILI, influenza-like illness; MD, mean difference; NOS, Newcastle-Ottawa scale; ORBIT, Outcome Reporting Bias in Trials; RR, risk ratio; WHO, World Health Organization

For peer review only

STRENGTHS AND LIMITATIONS OF THIS STUDY

- The review was conducted following established guidelines and a prospectively specified protocol
- The review focused on all-cause outcomes to reduce the risk of underestimating the vaccine impact that exists due to potential difficulties in identifying and accurately recording the cause of hospitalisation and death in patients with an underlying chronic disease.
- To reduce the risk of missing relevant studies and relevant data and to increase the objectivity of the review, two independent reviewers screened the studies, extracted data and assessed the risk of bias and quality of evidence.
- Differences in studies included in meta-analyses, publication bias, restriction on the publication language and varying response to queries about data on liver disease subgroups may have affected the effects observed in this review.
- Inclusion of non-randomised studies lowers the quality of evidence and limits the conclusions that can be drawn.

For peer review only

INTRODUCTION

Greater than 45 million people globally, including more than 8 million people in the US and Europe, are affected by chronic liver disease[1]. The prevalence of liver disease is increasing, driven by obesity and alcohol consumption [2],

The progression of liver disease is associated with immune dysregulation[3–6] and complications from common acute infections such as influenza cause significant morbidity and mortality. A 2-fold increased risk of hospital admission for influenza was observed in liver disease patients in 19 hospitals in Russia, Turkey, China, and Spain during the 2013/2014 season[7]. Similarly, an analysis of data on laboratory-confirmed influenza cases collected from several World Health Organization (WHO) member states during the 2009 influenza A (H1N1) pandemic found liver disease patients to have a greater than 5-fold increased risk of influenza-related hospitalisation and over 17-fold increased risk of death compared to that of healthy individuals[8]. Influenza infection, while not directly targeting the liver, may cause collateral liver damage[9] and trigger hepatic decompensation in liver disease[10].

Influenza vaccine introduces a whole or part of the influenza virion into the body stimulating antibody production against the virus, similar to an actual infection. In healthy adult and elderly populations, seasonal influenza vaccination is an effective way to prevent influenza infection[11,12]. People with liver disease, due to the high risk of severe health complications from influenza infection, are often a target of influenza vaccination policies[13] but randomised trials on vaccine effectiveness in liver patients are lacking as withholding the vaccine may place patients' safety and health at risk. For

For peer review only - http://bmjopen.bmj.com/site/about/guidelines.xhtml

example, a previous systematic review of randomised trials of influenza vaccine effectiveness in patients with chronic diseases, conducted in 2011[14], identified only one study in liver patients. Observational studies offer an alternative way of investigating vaccine effectiveness but without randomisation of patients the results are vulnerable to confounding and bias, in particular frailty bias. Frailty bias exist when some or all of the reduction in the risk of health complications may be due to the preferential receipt of vaccine by individuals who are in general more healthy. One solution to minimise the effect of frailty bias is to observe the vaccine effect both within and outside of the influenza season. If frailty bias exist, we would see higher rates of health complications in unvaccinated individuals outside of the influenza season. This would indicate that any protective effect observed within the season may be partially or fully due to the vaccinated individuals being healthier overall and an overestimation of the effect of the vaccine. Finally, in addition to measuring clinical outcomes to understand the effects of the vaccine, the antibody response elicited by the vaccine can be measured and used as a proxy for vaccine effectiveness.

There is uncertainty amongst primary and secondary care physicians whether influenza vaccines are able to trigger an appropriate antibody response and protect patients with liver disease from health complications[15], and the vaccination coverage is poor. Just over 55% of the chronic liver disease respondents to the 2016 US National Health Interview Survey[16] had been vaccinated against influenza in the past 12 months and less than 50% of working-age patients with liver disease registered with primary care practitioners in England had received influenza vaccination during the 2015/2016 season[17]. To inform vaccination policy and to help guide decision-making regarding vaccination, our review aims to provide a systematic synthesis of the available evidence

For peer review only - http://bmjopen.bmj.com/site/about/guidelines.xhtml

from both observational studies and randomised trials on the effectiveness of influenza vaccines to prevent serious health outcomes, hospitalisation and death, in adults with chronic liver disease. To address uncertainty over the vaccines' ability to trigger antibody response in liver patients, we also assess the effects of influenza vaccines on serological response in adults with chronic liver disease.

For peer review only

MATERIAL AND METHODS

Protocol and registration

This systematic review and meta-analysis followed the Preferred Reporting Items for Systematic Review and Meta-Analyses (PRISMA)[18]. The review protocol was specified in advance as part of a wider review plan that also includes a study of the effectiveness of pneumococcal vaccine. The protocol was registered in PROSPERO 13th June 2017 (registration number CRD42017067277) and published after peer-review on 16th March 2018[19]. Based on the peer-review feedback, we made minor amendments to the initial protocol (Supplementary materials, Table 1).

Eligibility criteria

Studies published in peer-reviewed journals were eligible for inclusion for this systematic review and meta-analysis if they met the following PICOS (participants, intervention, comparator, outcome and study design) criteria: study type was randomised or non-randomised controlled trial, cohort or case-control study; intervention was an injectable, inactivated whole-virus, split-virus or subunit influenza

For peer review only - http://bmjopen.bmj.com/site/about/guidelines.xhtml

vaccine; intervention was compared to placebo, alternative intervention or no vaccination (no comparison group was required for serological response); study population included at least ten ≥18 year-old adults with chronic liver disease of any severity or aetiology (by any classification) per comparison group; outcomes included all-cause hospitalisation or all-cause mortality (or supportive outcomes hospitalisation or mortality due to acute respiratory illness, influenza/ILI or liver disease complications) or serological response to influenza vaccine. We only included studies published in English. Review articles, case reports, cross-sectional studies, animal studies, editorials, clinical guidelines, studies with liver transplant patients only (response to the vaccination and clinical outcomes are likely to be strongly influenced by the immunosuppressive medication rather than the status of liver disease), studies focused on special populations (such as pregnant women, nursing home residents, or patients with other chronic diseases), test-negative case-control studies (where both cases and controls were hospitalised for influenza-related illness/ILI/acute respiratory illness, listed in Supplementary materials, Table 2) and studies retracted from publication were excluded.

Information sources and search strategy

We searched the Cochrane Central Register of Controlled Trials, Ovid MEDLINE, Ovid Embase and PubMed (originally up to 16 July 2017 and later updated up to 20 January 2019) for articles containing variations of terms “influenza”, “flu”, “seasonal”, “TIV”, “QIV”, “3-valent”, “4-valent”, “trivalent” or “quadrivalent” in combination with terms “vaccine” or “immunisation” and “liver disease”, “hepatic disease”, “chronic liver”, “chronic hepatic”, or “cirrhosis”. The search also included medical subject headings “Influenza Vaccines” (exploded), “Influenza, human /Prevention and Control”, and

For peer review only - http://bmjopen.bmj.com/site/about/guidelines.xhtml

"Liver Diseases" (exploded). The search was filtered by study design, age group of participants, and publication type. To capture studies where liver disease patients may have been included as part of a general population, the search was repeated leaving out the liver disease-specific terms and with the study design filter restricted to trials only. The full search strategy in MEDLINE and the medical subject headings for all databases searched are provided in Supplementary materials, Table 3 and Table 4. Electronic searches were complemented by manually searching recent influenza vaccine guidelines from Centers for Disease Control and Prevention, European Centre for Disease Control and Prevention, and WHO, and the reference lists of the included studies.

Study selection

Titles of the articles identified through the search of the studies of influenza vaccination in the general population were first pre-screened by one reviewer (SH). Abstracts and titles of articles meeting the pre-screening criteria and of all articles identified through the liver disease-specific search were then screened by two independent reviewers (CP and SH). Studies deemed eligible were included in the full-text review by two independent reviewers (CP and SH). Disagreements were resolved by discussion and the reasons for exclusion were recorded. In case of uncertainty over eligibility, study authors were contacted. Studies for which this uncertainty could not be resolved were excluded (Supplementary materials, Table 5).

Data collection process and data items

Using standardised online forms, two review authors (CP and SH) independently and in duplicate extracted data on: study participants (inclusion and exclusion criteria, method

For peer review only

of recruitment/selection, study population characteristics), interventions and comparators (vaccine type, comparison treatment, dose, route of delivery, number and timing of vaccinations/comparator treatments, number of individuals and follow-up time in intervention and comparison groups), outcomes (definition, time points measured and reported, unit of measurement, number of outcomes in the intervention and control group, unadjusted and adjusted effect measures, covariates that the effect measures were adjusted for, comparisons, missing data and reasons for missingness, statistical methods used and processes for randomisation), study design (study type, country and setting, date of study, study duration, aim of study and withdrawals), study quality, study bias, study funding, and conflicts of interest. We contacted study authors to obtain subgroup data, missing data, and to clarify unclear data. Effect measures were collected in the format in which they were reported and transformed for analysis as required.

For peer review only

Risk of bias in individual studies

Two review authors (CP and SH) independently assessed the risk of bias. We used the Cochrane Collaborations tool[20] to assess randomised trials and the Newcastle-Ottawa scale (NOS)[21] to assess observational studies. Age, sex, severity and aetiology of liver disease, and presence of co-morbidities were considered the most important confounders in the assessment of observational studies. Additionally, in studies of pandemic vaccine effect on clinical outcomes, we considered as a potential confounder a previous seasonal influenza vaccination in the same season the study vaccine was administered. Risk of bias assessment in serological studies focused on the study population with liver disease. Disagreements over risk of bias were resolved by discussion between reviewers.

Summary measures and synthesis of results

Where more than a single study per outcome was identified and the study designs, protocols and measures of treatment effect were considered similar enough to produce a meaningful pooled effect, we used random-effects meta-analysis to summarise the average effects of vaccination. For serological vaccine response, we compared the liver patients' haemagglutinisation inhibition (HI) antibody responses before and after vaccination. Comparisons between titres and titre ratios were presented as a mean difference (MD) of log geometric mean titre (GMT) or log geometric mean titre ratio (GMTR) with 95% confidence intervals (CI, titres and ratios were log transformed to back to their original scale to obtain standard deviations). Seroconversion (defined as the proportion of patients whose negative pre-vaccination serum converted to a HI titre >1:40, or who experienced at least a four-fold titre increase, after vaccination) and seroprotection (defined as the proportion of patients achieving a HI titre of > 1:40 after vaccination) levels were presented as prevalence rates (%) with 95% CI and comparisons between rates were presented as risk ratio (RR) with 95% CI. As reference levels, for a theoretically adequate response, we used 40% for seroconversion and 70% for seroprotection. Reported rates expressed only as percentages that did not correspond to an integer number of patients were imputed to the closest possible number of patients (closest integer above the reported non-integer for baseline and closest integer below for effect rates). Response measures were presented in categories by virus subtype. The vaccine effects on hospitalisation and mortality were compared between vaccinated and unvaccinated liver disease patients and presented as a crude RR with 95% CI (adjusted effect measures, reported separately within results, were available from two studies but on different scales). Effect measures from multiple

For peer review only - http://bmjopen.bmj.com/site/about/guidelines.xhtml

corresponding seasons were combined into an overall measure within each study. Presence of frailty bias were evaluated by contrasting the effect estimates in and out of influenza season. Person-time was presented as person-seasons for seasonal and as person-years for whole-year effect estimates. We regarded heterogeneity between studies as substantial if τ^2 was greater than 0, I^2 was more than 30% and the p-value for Q-statistic was less than 0.10. Uncertainty in the estimates of between-study variance, likely to be greater when only a small number of effect estimates are pooled together, was not quantified (option not available in the software used). Statistical analyses were performed using Stata 14.0 and RevMan 5.3.

Additional analyses

Subgroup analyses and meta-regression were not carried out due to the low number of studies. Sensitivity analyses were carried out to investigate whether the serological vaccine effects were similar in less severe (non-cirrhotic disease) and advanced liver disease (cirrhotic disease) comparing response in all patients to that in cirrhotic patients only. We were not able to conduct sensitivity analyses based on aetiology of disease as the study populations were mainly of mixed aetiologies. In studies that included a healthy control population, we compared antibody responses between liver disease patients and the controls.

Risk of bias across the studies

For each of the review outcomes, we evaluated the risk of selective outcome reporting bias using the Outcome Reporting Bias in Trials (ORBIT) classification system[22]. Presence of publication bias was assessed based on this evaluation and our review design (funnel plots or formal tests were not used because of the low number of

For peer review only

studies). The Grading of Recommendations Assessment, Development and Evaluation (GRADE) Working Group system[23] was used to assess and report the overall quality of evidence. In this assessment, we regarded a vaccine effect outside of the influenza season (suggesting the effect of frailty bias) for clinical outcomes as confounding (contributing to study limitations). All assessments were completed by two independent reviewers (CP and SH) and disagreements were resolved by discussion between reviewers.

Role of the funding source

The funder of this study had no role in study design, data collection, data analysis, data interpretation, or writing of the report. The corresponding author had full access to all the data in the study and had final responsibility for the decision to submit for publication.

Patient and public involvement

Patients and the public were not involved in the design or planning of this study.

RESULTS

The search of influenza studies in the general population provided a total of 10649 unique study records. After title screening, 1607 of these records and 608 unique records from the liver disease-specific search were included in the abstract and title screening. The full text of 286 studies was examined in detail, and of these, ten studies met the inclusion criteria for the review. An additional two studies were identified

For peer review only - http://bmjopen.bmj.com/site/about/guidelines.xhtml

through recent influenza vaccine guidelines, making the total number of included studies 12. The study flowchart is presented in Figure 1.

The included studies consisted of one randomized controlled trial and 11 cohort studies. Six studies included serological outcomes (Table 1) and six included clinical outcomes (Table 2). The studies with serological outcomes had low (4 studies) to moderate bias (2 studies) in selection of study population and low (2 studies), moderate (3 studies) and high bias (1 study) in assessment of the outcomes (bias mostly due to lack of description of independent, blind outcome assessment). Individual studies with clinical outcomes had moderate to low risk of bias in selection of the study population (in two studies vaccination status was self-reported) and low risk of bias in outcome assessment (majority used medical records). In most studies, the presence of confounding due to demographic and clinical characteristics was unclear (we did not have characteristics for the liver patient subgroups obtained from the studies conducted in the general population) and the results for control periods (non-influenza periods) were available only from two studies. High risk of selective outcome reporting was suspected in only one clinical study (outcome all-cause mortality) but mainly in serological studies (outcome GMTR). Evidence for all outcomes was judged as very low quality. Risk of bias and selective outcome reporting assessments, and the GRADE evidence profile are provided in Supplementary materials, Table 6, Table 7 and Table 8.

For peer review only

Table 1. Characteristics of the included studies on serological response to influenza vaccine

Study	Location	Design	Liver disease participants (% cirrhosis, % viral aetiology)	Sample size	Vaccine	Follow-up	Outcomes
Cheong et al (2006)[24]	South Korea	Cohort study with comparison group	Advanced cirrhosis patients (100% cirrhosis 72% viral aetiology)	50 (54 healthy controls)	Trivalent, split-virus	4 weeks	GMT<br><br>Seroconversion rate<br><br>Seroprotection rate
Duchini et al (2001)[25]	United States	Cohort study with comparison groups	Cirrhosis patients (100% cirrhosis, 56% viral aetiology	14 (9 healthy controls)	Trivalent, split-virus	6 weeks	GMT<br><br>GMTR<br><br>Seroconversion rate
Hernández-Guerra et al (2012)[26]	Canary Islands, Spain	Cohort study with comparison groups	Chronic HCV patients (0% cirrhosis, 100% viral aetiology)	25 (15 healthy controls)	Monovalent, split virus	4-11 weeks	GMT<br><br>GMTR<br><br>Seroconversion rate<br><br>Seroprotection rate
Ohfuji et al (2013)[27]	Japan	Cohort study	Chronic HCV patients (25% cirrhosis, 100% viral aetiology)	79	Monovalent, split virus	3 weeks	GMT<br><br>GMTR<br><br>Seroconversion rate<br><br>Seroprotection rate
Sayyad et al (2012)[28]	Iran	Cohort study with comparison group	Cirrhosis patients and inactive HBV carriers (47% cirrhosis, 100% viral aetiology in non-cirrhotics, aetiologies not provided in cirrhotics)	28, 31 (34 healthy controls)	Trivalent, subunit	4 weeks	GMT<br><br>Seroconversion rate<br><br>Seroprotection rate
Soesman et al (2000)[29]	The Netherlands	Cohort study with comparison groups	Cirrhosis patients (100% cirrhosis, aetiologies not provided)	36 (45 healthy controls)	Trivalent, subunit	4 weeks	GMT<br><br>Seroconversion rate<br><br>Seroprotection rate

For peer review only - http://bmjopen.bmj.com/site/about/guidelines.xhtml

Table 2. Characteristics of the included studies on clinical outcomes after influenza vaccination

Study	Location	Design	Liver disease participants	Sample size (liver disease patients)	Study period	Outcomes
Campitelli et al (2010)[30]	Canada	Cohort study	Chronic liver disease patients among the community-dwelling ≥65 year-olds	205 vaccinated/124 unvaccinated	8 influenza seasons 1996-1998 and 2001-2007	All-cause mortality<br><br>Acute respiratory illness-related hospitalisation (pneumonia & influenza)
Castilla et al (2015)[31]	Spain	Cohort study	Cirrhosis patients among the community-dwelling ≥65 year-olds	3126 person-seasons vaccinated/1804 person-seasons unvaccinated<br><br>Number of patients not reported.	2 influenza seasons 2011-2013	All-cause mortality
Ohfuji et al (2014)[32]	Japan	Cohort study	Chronic HCV hospital outpatients (64% ≥65 year-olds)	132 vaccinated/276 unvaccinated	1 influenza season	All-cause hospitalisation
Song et al (2007)[33]	South Korea	Randomised, controlled trial	Cirrhotic hospital in- and outpatients (50% ≥55 year-olds)	175 vaccinated/90 unvaccinated	1 influenza season 2004-2005	Influenza illness-related mortality (laboratory-confirmed influenza)
Su et al (2016)[34]	Taiwan	Cohort study	Chronic HBV hospital outpatients (38% ≥60 year-olds)	4434 vaccinated/3646 unvaccinated	9 influenza seasons 2000-2009	All-cause hospitalisation<br><br>All-cause mortality<br><br>Liver disease complication related hospitalisation (septicaemia, bacteraemia, viraemia)
Vila-Córcoles et al (2007)[35]	Spain	Cohort study	Cirrhosis patients among the community-dwelling ≥65 year-olds	100-117 vaccinated/73-104 unvaccinated (numbers varied per year)	4 influenza seasons, 3 non-influenza periods, 3 full years 2002-2005	All-cause mortality

For peer review only

The serological response to influenza vaccine was assessed in six studies including a total of 262 liver disease patients (Table 1). Each study employed a cohort design in which all study participants were vaccinated. Mean age of liver disease participants ranged between 42 and 65 years (one study did not report baseline characteristics of participants). Three studies enrolled exclusively cirrhotic patients in their liver disease group, two studies enrolled both patients with and without cirrhosis and one study had separate groups for cirrhotic and non-cirrhotic patients. Hepatitis C was the main cause of liver disease in three studies and hepatitis B in two studies. In one study, aetiology was not specified. Four studies immunised the participants with a trivalent seasonal vaccine and two used a monovalent pandemic vaccine. In all studies, blood samples for post-vaccination measurements were drawn after minimum of three weeks and antibody titres measured using a traditional HI assay (antibody titre data is provided in Supplementary materials, Table 9).

HI antibody levels of liver disease patients increased in response to vaccination (pooled random-effects MD of log GMTs 1·92, 95% CI [1·65, 2·19]) (Figure 2). There was no substantial heterogeneity between individual estimates (τ^2=0·04, I^2=15%, p=0·28) or levels against influenza A and B viruses (p=0·16). Seroconversion rate in liver disease patients to both A/H1N1 and B subtypes was clearly above the 40% reference level (pooled random-effects prevalence for A/H1N1 79·95%, 95% CI [68·19%, 89·70%] and pooled random-effects prevalence for B 86·82%, 95% CI [71·87%, 97·07%]) and around the 40% in the case of subtype A/H3N2 (pooled random-effects prevalence 55·53%, 95% CI [39·92%, 70·63%]). While the majority of the studies reported

For peer review only

estimates above 40%, there was substantial heterogeneity between response rates (I^2=76·55% and p<0.01 for subtype A/H1N1; I^2=72·37% and p=0·01 for subtype A/H3N2; I^2=79·99% and p<0·01 for subtype B). Seroconversion rate is presented in Figure 3. Seroprotection rate in liver disease patients was above the 70% reference level (pooled random-effects rate 93·88%, 95% CI [87·33%, 98·40%]) (Figure 4). While the majority of studies reported a seroprotection rate above 70%, there was substantial heterogeneity (I^2=83·81%, p<0.01) (Figure 4). Although some studies reported a substantial seroprotection level before vaccination, the pooled pre-vaccination seroprotection rate was clearly below 70% (Supplementary materials, Figure 1).

The effectiveness of influenza vaccine in preventing all-cause hospitalisation in liver disease patients was investigated in two cohort studies, published in 2014 and 2016[32,34]. A study of 408 participants from Japan[32] investigated pandemic vaccine (against a single virus subtype) and enrolled exclusively chronic hepatitis C virus (HCV) patients. A study of 8080 participants from Taiwan[34] investigated seasonal vaccine (against multiple virus subtypes) exclusively in chronic hepatitis B virus (HBV) patients. In the study of HCV patients, patients were prospectively recruited from those who were under clinical follow-up for their liver disease but vaccination status was self-reported[32]. In the study of HBV patients, both liver disease and vaccination status were determined from electronic medical claim records[34]. One study presented results for one influenza season and the other for an entire year after vaccination and pooled the results from a period of nine years (Figure 5). Both studies compared the rate of all-cause hospital admission (first incidence) between vaccinated and unvaccinated individuals.

For peer review only - http://bmjopen.bmj.com/site/about/guidelines.xhtml

Vaccinated HCV patients, were no less likely to be hospitalised than unvaccinated patients during an influenza season after pandemic influenza A vaccination (RR 0·57, 95% CI [0·24, 1·37], RD -0.03, 95% CI [-0.08, 0.01])[32]. In this study, hospitalisation outside the influenza season was not measured. Adjusting the effect estimate (for chronic co-morbidities, steroid treatment within the preceding 6 months and albumin level), calculated in the study as the odds ratio (crude odds ratio 0.55, 95% CI [0.22,1.39]), adjusted odds ratio 0.43, 95% CI [0.16,1.17]), did not reveal a significant effect[32]. Seasonal vaccination, however, was associated with a drop from 205/1000 patients to 149/1000 patients being hospitalised during a full year of follow-up (RD -0·06, 95% CI [-0·07, -0·04]). Vaccinated HBV patients were 27% less likely to be admitted to the hospital than unvaccinated patients (RR 0·73, 95% CI [0·66, 0·80])[34]. Both the crude and the adjusted time-to-event estimate that accounted for cirrhosis status, age, sex, pneumococcal vaccine, comorbidities and recent hospitalisation, calculated in the study as the hazard ratio (HR), showed a protective effect (crude HR 0·68, 95% CI [0·62, 0·76], adjusted HR 0.56, 95% CI [0·50, 0·62])[34]. Based on a Kaplan-Meier plot, the difference in event-free survival probability between vaccinated and unvaccinated was established during the influenza season, approximately within the first three months after vaccination[34]. The effect of influenza vaccine on cause-specific hospitalisation was investigated in two studies[30,34]. A cohort study from Canada, published in 2010, measured acute respiratory illness-related hospitalisation during influenza season (defined as influenza/pneumonia hospitalisation) using medical claims and hospital records[30]. A total of 329 liver disease patients were included as part of a general population, however, as there were five or fewer hospitalisation events in both groups, the study authors were unable to release the results (reason: institution's data policy, personal communication). A liver disease

For peer review only - http://bmjopen.bmj.com/site/about/guidelines.xhtml

complication-related hospitalisation outcome was included in the Taiwanese study of HBV patients[34]. Vaccinated liver disease patients were 57% less likely to be admitted to hospital for unspecified septicaemia, bacteraemia and viraemia than unvaccinated patients (RR 0·43, 95% CI [0·31, 0·59]). Vaccination was associated with a fall from 26/1000 patients to 11/1000 patients being hospitalised (RD -0·02, 95% [CI -0·02, -0·01]). It appeared, however, that a difference in event-free survival probability was not established during the influenza season (Kaplan-Meier plot)[34]. No studies with the outcome influenza illness/ILI-related hospitalisation were identified.

The effectiveness of influenza vaccine in preventing all-cause mortality in liver disease patients was investigated in five cohort studies[30,31,34,35], published between 2007 and 2016. Only the Taiwanese study of HBV patients, enrolled exclusively liver disease patients[34]. Two studies from Spain[31,35] and one from Canada[30] enrolled liver disease patients as part of the general, community-dwelling population. All studies ascertained liver disease diagnosis and vaccination status from electronic health or medical claim records. In one study, the record-based vaccination status was based on self-reporting. The Spanish studies followed patients over multiple seasons from the start of the study allowing them to contribute to both the unvaccinated and vaccinated follow-up time for different seasons [31,35] whereas the other two studies followed patients up for a single season/year[30,34], although this was over multiple seasons/years. All studies compared all-cause mortality rates between vaccinated and unvaccinated individuals.

Results are presented in Figure 6. Vaccinated patients were no less likely to die during influenza season (pooled random effects RR 0·80, 95% CI [0·43, 1·50], RD -0.001, 95%

For peer review only - http://bmjopen.bmj.com/site/about/guidelines.xhtml

CI [-0.01, 0.01]), or the entire year (pooled random effects RR 0·41, 95% CI [0·11, 1·52], RD -0.06, 95% CI [-0.08, 0.04]). All the studies that contributed to the influenza season-specific estimate reported non-significant effects and there was no substantial heterogeneity (τ^2=0·12, I^2=33·00%, p=0·23). The studies that contributed to the whole-year estimate were substantially heterogeneous (τ^2=0·83, I^2=94.00%, p<0.00), with the smaller Spanish study[35] reporting a non-significant effect (personal communication) and the larger Taiwanese study reporting a large protective effect (RR 0·22, 95% CI [0·17, 0·28])[34]. The Spanish study reported no effect outside the influenza period (RR 0·52, 95% CI [0·15, 1·83]). The Canadian study also measured the effect outside the non-influenza period but as there were five or fewer deaths in both groups, the authors were unable to provide the results (institution's data policy, personal communication). Based on a Kaplan-Meier plot presented in the Taiwanese study, the difference in survival probability was mainly established approximately in the first five months after vaccination[34], however, it was not clear whether the increase in death rate fully levelled off after the influenza season and it may be that this to some extent contributed to the size of the yearly effect estimate in this study. The effect of influenza vaccine on the rate of cause-specific mortality was investigated in one study[33]. The effect of influenza vaccine in preventing influenza illness/ILI-related mortality was included as an outcome in a South Korean randomised, controlled trial of cirrhotic patients, published in 2007[33]. No deaths due to laboratory-confirmed influenza occurred among the 175 vaccinated or the 90 unvaccinated patients during the influenza season[33]. No studies investigating mortality related to acute respiratory illness or liver disease complications were identified.

For peer review only - http://bmjopen.bmj.com/site/about/guidelines.xhtml

To understand whether the vaccine effects were different based on the patients' cirrhosis status, we conducted sensitivity analyses. The serological response in cirrhotic patients did not differ from the results of our main analyses (Supplementary materials, Figures 2-5). We did not have the data and therefore were not able to conduct similar analyses for the clinical outcomes.

In addition to comparing the vaccine effects within liver patients, the studies of serological response (5/6 studies) also compared the outcomes between liver disease patients and healthy controls (individuals without liver disease). The antibody titres before or after vaccination, seroconversion rate and seroprotection rate of liver patients did not differ from those of healthy individuals. Pre- to post-vaccination titre ratios (GMTRs) reported were similar between liver patients and healthy individuals except for influenza subtype A/H3N2 where the mean fold-increase in antibody levels of liver patients was lower than that of healthy individuals. The pooled results for these comparisons are provided in Supplementary materials, Figures 6-11. The studies on clinical outcomes in liver patients did not include healthy comparison groups and while we did not seek these data from the studies that included liver patients as part of a general population, the all-cause mortality results in the entire general population can be found in Supplementary materials, Table 10.

DISCUSSION

Six cohort studies from three continents (Asia, Europe and North America) investigated the serological vaccine response in liver disease patients. Two cohort studies and one

For peer review only - http://bmjopen.bmj.com/site/about/guidelines.xhtml
For peer review only

randomised controlled trial, all from Asia, investigated the vaccine effect on hospitalisation and/or mortality in liver disease patients exclusively. In addition, there are at least three cohort studies, two from Europe and one from North America, that have assessed the vaccine effect on these outcomes in a general community-dwelling elderly population including a subgroup with liver disease. Overall, the available evidence suggests that while influenza vaccine may not protect against all-cause mortality, it triggers an effective antibody response and may reduce the risk of all-cause hospitalisation in liver disease patients (findings for clinical outcomes summarised in Table 3, maximum quality obtainable is "low" as all included studies are non-randomised).

Table 3. Summary of findings for clinical outcomes

Effectiveness of influenza vaccination in preventing all-cause hospitalisation and all-cause mortality in adults with chronic liver disease

	**Patients: Adults with chronic liver disease**<br>**Setting: General population**<br>**Intervention: Influenza vaccination (injectable, inactivated)**<br>**Comparison: No vaccination**					
**Outcome**	**Risk/rate in unvaccinated** [a]	**Risk/rate in vaccinated**	**Relative effect**	**Vaccine effectiveness**	**Number of participants/person-time (number and type of studies)**	**Quality of evidence (GRADE)**
All-cause hospitalisation, influenza season (pandemic vaccine)	80 per 1000	46 per 1000 (19-110)	0.57 (0.24, 1.37)	Not significant	408 (1 observational study)	Very low[b]
All-cause hospitalisation, all year	205 per 1000	150 per 1000 (135-164)	0.73 (0.66, 0.80)	27% (20%, 34%)[c]	8080 (1 observational study)	Very low[d]
All-cause mortality, influenza season	17 per 1000 person-seasons	14 per 1000 person-seasons (7-26)	0.80 (0.43, 1.50)	Not significant	5975 person-seasons (3 observational studies)	Very low[e]

For peer review only - http://bmjopen.bmj.com/site/about/guidelines.xhtml

All-cause mortality, all year	85 per 1000 person-seasons	35 per 1000 person-seasons (9-129)	0.41 (0.11, 1.52)	Not significant	8405 person-years (2 observational studies)	Very low[f]

[a] Median risk in unvaccinated across the studies.
[b] Rated down from low to very low based on study limitations, imprecision of the estimate and suspicion of publication bias.
[c] Could be applicable to HBV patients only as the study only enrolled HBV patients.
[d] Rated down from low to very low based on suspicion of publication bias.
[e] Rated down from low to very low based on study limitations, inconsistency in absolute rates, imprecision of the estimate and suspicion of publication bias.
[f] Rated down from low to very low based on study limitations, imprecision of the estimate and suspicion of publication bias.

A previous systematic review[14] on the effectiveness of inactivated influenza vaccine in different at-risk target groups identified only one randomised study in liver patients. This study of 311 cirrhosis patients, also included in our current review, found seasonal influenza vaccine effective against laboratory-confirmed influenza but not against ILI[33]. The study included hospitalised patients and could not investigate vaccine effectiveness against hospitalisation. Vaccine effect on influenza-related mortality was investigated, however, no deaths occurred during the study. It is possible that the effects we found are not entirely due to the vaccine. Other factors that may have affected the clinical outcomes include frailty bias, competing risks (in case of our secondary, cause-specific outcomes) and differences in previous vaccinations, social support, healthcare system, lifestyle, and in the severity or type of liver disease between the vaccinated and unvaccinated populations. Serological outcomes may have been further affected by variation of the antibody assays.

The data presented are predominantly from patients with viral liver disease. We cannot therefore be certain that our findings are applicable to patients with liver disease caused by alcohol, obesity or other non-viral factors. While sensitivity analyses showed

For peer review only

that compared to non-cirrhotic liver disease, having cirrhosis did not worsen the antibody response to the vaccine, most data in the analyses of clinical outcomes come from patients with a milder form of liver disease than cirrhosis. We might expect that in severe liver disease complications from influenza are more frequent and we may have seen more outcome events if more cirrhotic patients were included. Given the proxy effectiveness in cirrhotic patients indicated by the antibody responses, this would mean that our study may have somewhat underestimated the true protective effect against clinical outcomes. However, all our data on the hospitalisation outcome are from high-income Asian countries and it is possible that the protective vaccine effect may not extend to populations from other geographical and income settings with different prevalence of co-morbidities, age distribution, influenza seasonality and vaccine formulation used.

Our review has several strengths. It was conducted in a transparent way following established guidelines and a prospectively specified protocol. The search was conducted over multiple databases and we searched for both studies in liver disease patients and studies in which liver disease patients were included as part of the general population. To reduce the risk of missing relevant studies and relevant data and to increase the objectivity of the review, two independent reviewers screened the studies, extracted data and assessed the risk of bias. We studied all-cause outcomes to take into account the complex interplay of a chronic condition and acute infections. An underlying diagnosis of chronic liver disease may influence how cause of death and hospitalisation is recorded. This potentially reduces the number of cases that are attributed to influenza and solely investigating diagnoses of influenza is likely to substantially underestimate the impact of the vaccine.

For peer review only

There are also limitations to our review. The meta-analyses combine data from studies that differ in terms of patient population, outcome definitions and vaccine used. For the studies that included liver disease patients as part of the general population, we had very limited information about the baseline characteristics of the liver patient subgroup. In addition, despite the comprehensive search, publication bias, restriction on publication language and varying response to queries about data on liver disease subgroups may have affected the effects observed. We were not able to employ translation services and may have omitted relevant studies published in languages other than English from countries such as China where influenza vaccines are manufactured and liver disease is a significant public health challenge. A number of studies potentially included liver disease patients as part of a wider study population but were unable to obtain confirmation and liver disease subgroup data from the study authors. Finally, the body of evidence identified in this review is limited both in quantity and quality, leaving room for uncertainty around the effects of the vaccination. The low quality is partly due to the non-randomised design of the included studies. This limitation may persist also in future reviews as conducting randomised controlled trials in a high-risk population are ethically challenging.

Evidence from mainly patients with viral liver disease suggests that underlying liver disease does not prevent patients from mounting an antibody response to influenza vaccine and similar proxy effectiveness is seen both in non-cirrhotic and cirrhotic patients. We also identified a protective influenza vaccine effect against yearly all-cause hospitalisation in patients with viral liver disease. Assuming this effect – estimated in a large cohort study of over 8000 patients – is due to the vaccine, seasonal influenza

For peer review only

vaccination of 14–24 viral liver disease patients could prevent one hospitalisation per year. It is uncertain whether the estimated effects extend to patients with liver disease of other than viral origin. No protective effect against all-cause mortality was found.

Current uptake of seasonal influenza vaccination is low. A recent systematic review identified low perceived vaccine effectiveness, and low concern and perceived risk of severe influenza infection as the most common barriers for seasonal influenza vaccination in patients with chronic illness[36]. Ideally, the evidence for influenza vaccine effectiveness in liver disease would come from a large randomised vaccine trial in liver disease patients. In the absence of this evidence, we must rely on proxy estimates for effectiveness from serological studies and evidence on clinical outcomes from observational studies. The best available evidence on the effectiveness of influenza vaccine in liver disease, summarised in this review, shows both proxy effectiveness and protection against all-cause hospitalization. The limited quantity and quality of this evidence means that the protective vaccine effect may be uncertain. However, considering the high risk of serious health outcomes from influenza infection in liver patients and the safety and low cost of vaccination, overall the potential benefits of seasonal vaccination both to patients and the healthcare systems are likely to outweigh the undesirable consequences of severe influenza infection. Wider collection of vaccination data in adults and liver patient -focused studies using large databases linking routinely collected data on vaccinations and health may allow us to address the remaining uncertainty about the effects of influenza vaccination in liver disease.

Contributors

For peer review only

The study was conceived by SH, LS, AO and AH. SH developed the eligibility criteria, search strategy, risk of bias assessment strategy and data extraction plan with guidance from LS, AO and AH. SH (trained in systematic methods of literature searching as part of her PhD studies) performed the literature searches and pre-screening of titles. SH and CP reviewed titles and abstracts, and full text articles, extracted data, and assessed study bias and quality. SH analysed the data, and wrote the manuscript to which all authors CP, LS, AO and AH contributed.

Funding

This work was supported by the UK Biotechnology and Biological Sciences Research Council grant [grant number BBSRC BB/M009513/1] to SH.

Data sharing statement

All data relevant to the study are included in the article or uploaded as supplementary information.

Competing interests

The authors declare that no competing interests exist.

Acknowledgements

We wish to thank the authors of the reviewed studies for kindly responding to our enquiries and for providing liver patient subgroup data where possible.

For peer review only - http://bmjopen.bmj.com/site/about/guidelines.xhtml

Figure legends

Figure 1. Study selection

Figure 2. Serological response to influenza vaccination: mean difference in log HI antibody GMTs before and after vaccination. *Note: Sayyad et al (2012a) includes cirrhotic patients and Sayyad et al (2012b) includes non-cirrhotic patients only.*

Figure 3. Serological response to influenza vaccination: seroconversion rate after vaccination. *Note: Sayyad et al (2012a) includes cirrhotic patients and Sayyad et al (2012b) includes non-cirrhotic patients only.*

Figure 4. Serological response to influenza vaccination: seroprotection rate after vaccination. *Note: Sayyad et al (2012a) includes cirrhotic patients and Sayyad et al (2012b) includes non-cirrhotic patients only.*

Figure 5. Effect of influenza vaccine on clinical outcomes: all-cause hospitalisation in vaccinated compared to unvaccinated liver disease patients. Note: Ohfuji et al (2014) investigated the effect of pandemic (monovalent) vaccine and Su et al (2016) of seasonal vaccine (against three or more virus subtypes).

Figure 6. Effect of influenza vaccine on clinical outcomes: all-cause mortality in vaccinated compared to unvaccinated liver disease patients.

For peer review only

For peer review only - http://bmjopen.bmj.com/site/about/guidelines.xhtml

For peer review only

REFERENCES

1 GBD 2016 Disease and Injury Incidence and Prevalence Collaborators. Global, regional, and national incidence, prevalence, and years lived with disability for 328 diseases and injuries for 195 countries, 1990–2016: a systematic analysis for the Global Burden of Disease Study 2016. *Lancet* 2017;**390**:1211–59. doi:10.1016/S0140-6736(17)32154-2

2 Asrani SK, Devarbhavi H, Eaton J, *et al.* Burden of liver diseases in the world. *J Hepatol* 2019;**70**:151–71. doi:10.1016/j.jhep.2018.09.014

3 Fierer J, Finley F. Deficient Serum Bactericidal Activity Against Escherichia Coli in Patients with Cirrhosis of the Liver. *J Clin Invest* 1979;**63**:912–21. doi:10.1172/JCI109391

4 Hassner A, Kletter Y, Shlag D, *et al.* Impaired monocyte function in liver cirrhosis. *Br Med J (Clin Res Ed)* 1981;**282**:1262–3.

5 Brien AJO, Fullerton JN, Massey KA, *et al.* Prostaglandin E2 Mediates Immunosuppression in Acutely Decompensated Cirrhosis. *Nat Med* 2014;**20**:518–23. doi:10.1038/nm.3516

6 Rajkovic IA, Williams R. Abnormalities of Neutrophil Phagocytosis, Intracellular Killing and Metabolic Activity in Alcoholic Cirrhosis and Hepatitis. *Hepatology* 1986;**6**:252–62. doi:10.1002/hep.1840060217

7 Puig-Barberà J, Natividad-Sancho A, Trushakova S, *et al.* Epidemiology of Hospital Admissions with Influenza during the 2013 / 2014 Northern Hemisphere Influenza Season: Results from the Global Influenza Hospital Surveillance Network. *PLoS One* 2016;**11**:e0154970. doi:10.1371/journal.pone.0154970

8 Kerkhove MD Van, Vandemaele KAH, Shinde V, *et al.* Risk Factors for Severe

For peer review only

Outcomes following 2009 Influenza A (H1N1) Infection: A Global Pooled Analysis. *PLoS Med* 2011;**8**:e1001053. doi:10.1371/journal.pmed.1001053

9 Polakos NK, Cornejo JC, Murray DA, *et al.* Kupffer Cell-Dependent Hepatitis Occurs during Influenza Infection. *Am J Pathol* 2006;**168**:1169–78. doi:10.2353/ajpath.2006.050875

10 Duchini A, Viernes ME, Nyberg LM, *et al.* Hepatic Decompensation in Patients With Cirrhosis During Infection With Influenza A. *Arch Intern Med* 2000;**160**:113–5. doi:10.1001/archinte.160.1.113

11 Demicheli V, Jefferson T, Ferroni E, *et al.* Vaccines for preventing influenza in healthy adults. *Cochrane Database Syst Rev* Published Online First: 2018. doi:10.1002/14651858.CD001269.pub6

12 Demicheli V, Jefferson T, Di Pietrantonj C, *et al.* Vaccines for preventing influenza in the elderly. *Cochrane Database Syst Rev* Published Online First: 2018. doi:10.1002/14651858.CD004876.pub4

13 European Centre for Disease Prevention and Control. Seasonal influenza vaccination and antiviral use in Europe – Overview of vaccination recommendations and coverage rates in the EU Member States for the 2013–14 and 2014–15 influenza seasons. 2016.

14 Michiels B, Govaerts F, Remmen R, *et al.* A systematic review of the evidence on the effectiveness and risks of inactivated influenza vaccines in different target groups. *Vaccine* 2011;**29**:9159–70. doi:10.1016/j.vaccine.2011.08.008

15 Leise MD, Talwalkar JA. Immunizations in chronic liver disease: what should be done and what is the evidence. *Curr Gastroenterol Rep* 2013;**15**:300. doi:10.1007/s11894-012-0300-6

16 National Center for Health Statistics. National Health Interview Survey. Public-use

For peer review only

data file and documentation. 2016.

17 Public Health England. Influenza immunisation programme for England: GP patient groups - Data collection survey Season 2015 to 2016. 2016.

18 Moher D, Liberati A, Tetzlaff J, *et al.* Academia and Clinic Annals of Internal Medicine Preferred Reporting Items for Systematic Reviews and Meta-Analyses: The PRISMA Statement. *Annu Intern Med* 2009;**151**:264–9. doi:10.1371/journal.pmed1000097

19 Härmälä S, Parisinos C, Shallcross L, *et al.* Effectiveness of pneumococcal and influenza vaccines to prevent serious health complications in adults with chronic liver disease: A protocol for a systematic review. *BMJ Open* 2018;**8**. doi:10.1136/bmjopen-2017-018223

20 The Cochrane Collaboration. Cochrane Handbook for Systematic Reviews of Interventions. Version 5.1.0 [updated March 2011]. 2011.http://handbook-5-1.cochrane.org/ (accessed 25 Jul 2018).

21 Wells G, Shea B, O'Connell D, *et al.* The Newcastle-Ottawa Scale (NOS) for assessing the quality of nonrandomised studies in meta-analyses. http://www.ohri.ca/programs/clinical_epidemiology/oxford.asp (accessed 25 Jul 2018).

22 Kirkham JJ, Dwan KM, Altman DG, *et al.* The impact of outcome reporting bias in randomised controlled trials on a cohort of systematic reviews. *BMJ* 2010;**340**:c365. doi:10.1136/bmj.c365

23 Guyatt GH, Oxman AD, Vist GE, *et al.* GRADE: an emerging consensus on rating quality of evidence and strength of recommendations. *BMJ* 2008;**336**:924–6. doi:10.1136/bmj.39489.470347.AD

24 Cheong H-J, Song J-Y, Park J-W, *et al.* Humoral and cellular immune responses to

For peer review only

influenza vaccine in patients with advanced cirrhosis. *Vaccine* 2006;**24**:2417–22. doi:10.1016/j.vaccine.2005.11.064

25 Duchini A, Hendry RM, Nyberg LM, *et al.* Immune response to influenza vaccine in adult liver transplant recipients. *Liver Transplant* 2001;**7**:311–3. doi:10.1053/jlts.2001.23010

26 Hernandez-Guerra M, Gonzalez-Mendez Y, de Molina P, *et al.* Immunogenicity and Acceptance of Influenza A (H1N1) Vaccine in a Cohort of Chronic Hepatitis C Patients Receiving Pegylated-Interferon Treatment. *PLoS One* 2012;**7**:e48610. doi:10.1371/journal.pone.0048610

27 Ohfuji S, Fukushima W, Tamori A, *et al.* Immunogenicity of influenza A(H1N1)pdm09 vaccine and the associated factors on lowered immune response in patients with hepatitis C. *Influenza Other Respi Viruses* 2013;**7**:456–65. doi:10.1111/j.1750-2659.2012.00424.x

28 Sayyad B, Alavian SM, Najafi F, *et al.* Efficacy of Influenza Vaccination in Patients with Cirrhosis and Inactive Carriers of Hepatitis B Virus Infection. *Iran Red Crescent Med J* 2012;**14**:623–30.

29 Soesman NMR, Rimmelzwaan GF, Nieuwkoop NJ, *et al.* Efficacy of influenza vaccination in adult liver transplant recipients. *J Med Virol* 2000;**61**:85–93. doi:10.1002/(SICI)1096-9071(200005)61:1<85::AID-JMV14>3.0.CO;2-H

30 Campitelli MA, Rosella LC, Stukel TA, *et al.* Influenza vaccination and all-cause mortality in community-dwelling elderly in Ontario, Canada, a cohort study. *Vaccine* 2010;**29**:240–6. doi:10.1016/j.vaccine.2010.10.049

31 Castilla J, Guevara M, Martinez-Baz I, *et al.* Enhanced estimates of the influenza vaccination effect in preventing mortality a prospective cohort study. Med. (United States). 2015;**94**:e1240.

For peer review only - http://bmjopen.bmj.com/site/about/guidelines.xhtml

32 Ohfuji S, Fukushima W, Sasaki Y, *et al.* Influenza A(H1N1)pdm09 vaccine effectiveness and other characteristics associated with hospitalization in chronic liver disease patients. *Liver Int* 2014;**34**:700–6. doi:10.1111/liv.12295

33 Song JY, Cheong HJ, Ha SH, *et al.* Clinical impact of influenza immunization in patients with liver cirrhosis. *J Clin Virol* 2007;**39**:159–63. doi:10.1016/j.jcv.2007.04.018

34 Su F-H, Huang Y-L, Sung F-C, *et al.* Annual influenza vaccination reduces total hospitalization in patients with chronic hepatitis B virus infection: A population-based analysis. *Vaccine* 2016;**34**:120–7. doi:10.1016/j.vaccine.2015.10.129

35 Vila-Córcoles A, Rodriguez T, de Diego C, *et al.* Effect of influenza vaccine status on winter mortality in Spanish community-dwelling elderly people during 2002-2005 influenza periods. Vaccine. 2007;**25**:6699–707.

36 Schmid P, Rauber D, Betsch C, *et al.* Barriers of influenza vaccination intention and behavior - A systematic review of influenza vaccine hesitancy, 2005-2016. *PLoS One* 2017;**12**:e0170550. doi:10.1371/journal.pone.0170550

For peer review only

For peer review only - http://bmjopen.bmj.com/site/about/guidelines.xhtml

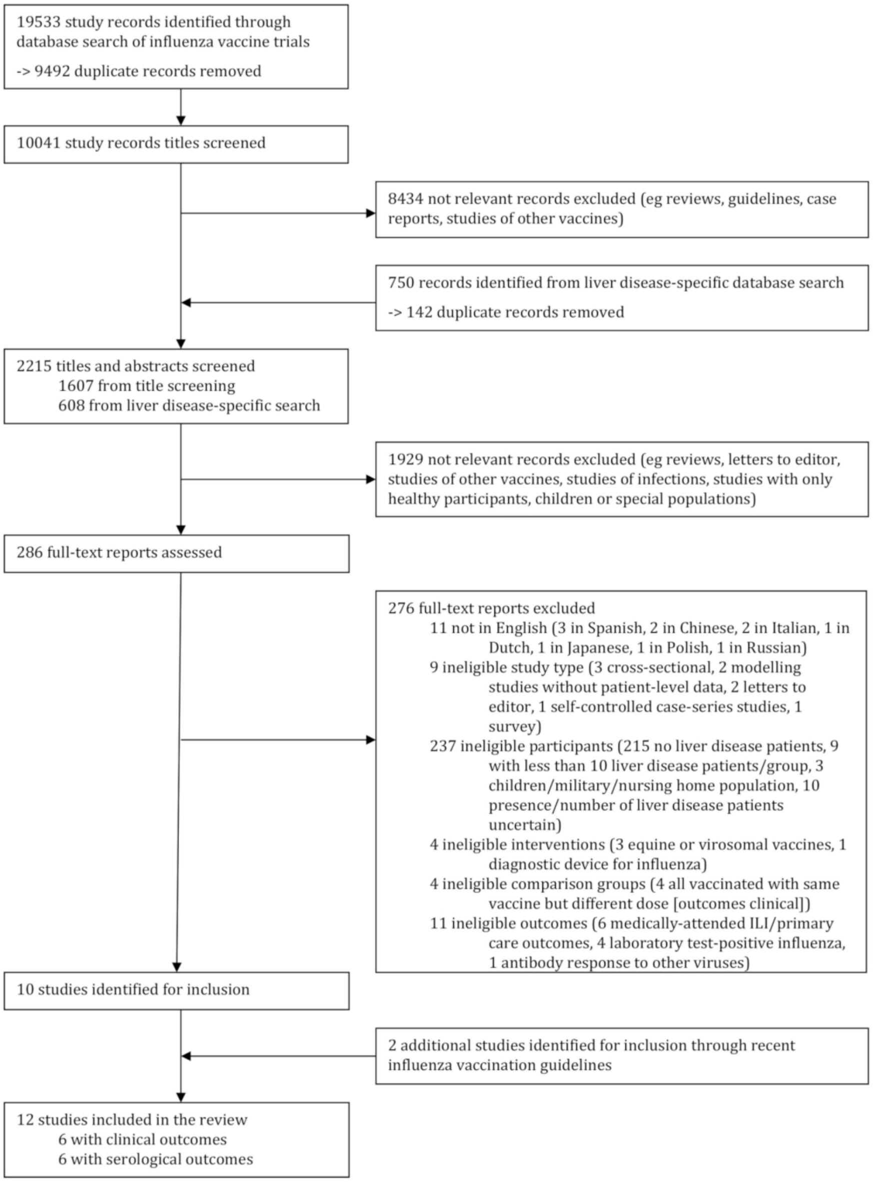

Figure 1. Study selection

150x202mm (300 x 300 DPI)

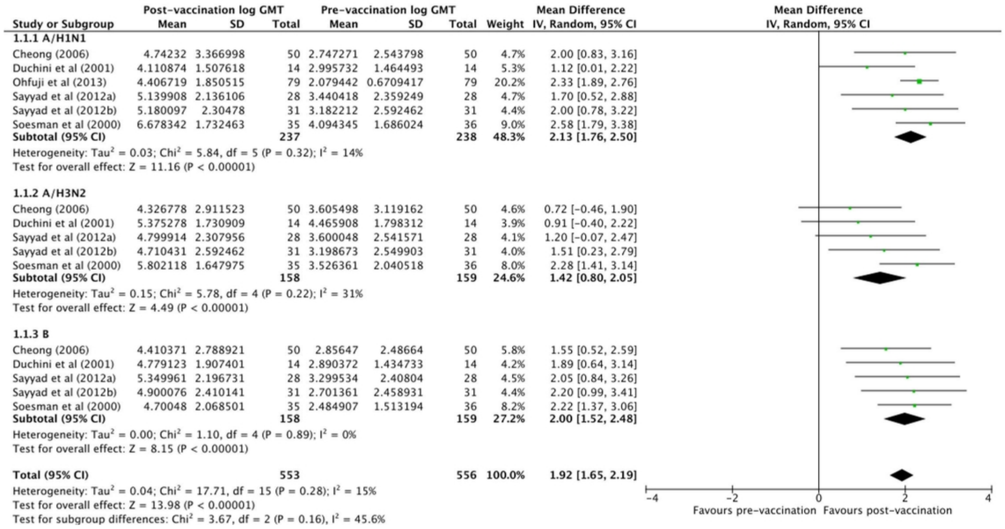

Figure 2. Serological response to influenza vaccination: mean difference in log HI antibody GMTs before and after vaccination. Note: Sayyad et al (2012a) includes cirrhotic patients and Sayyad et al (2012b) includes non-cirrhotic patients only.

96x50mm (300 x 300 DPI)

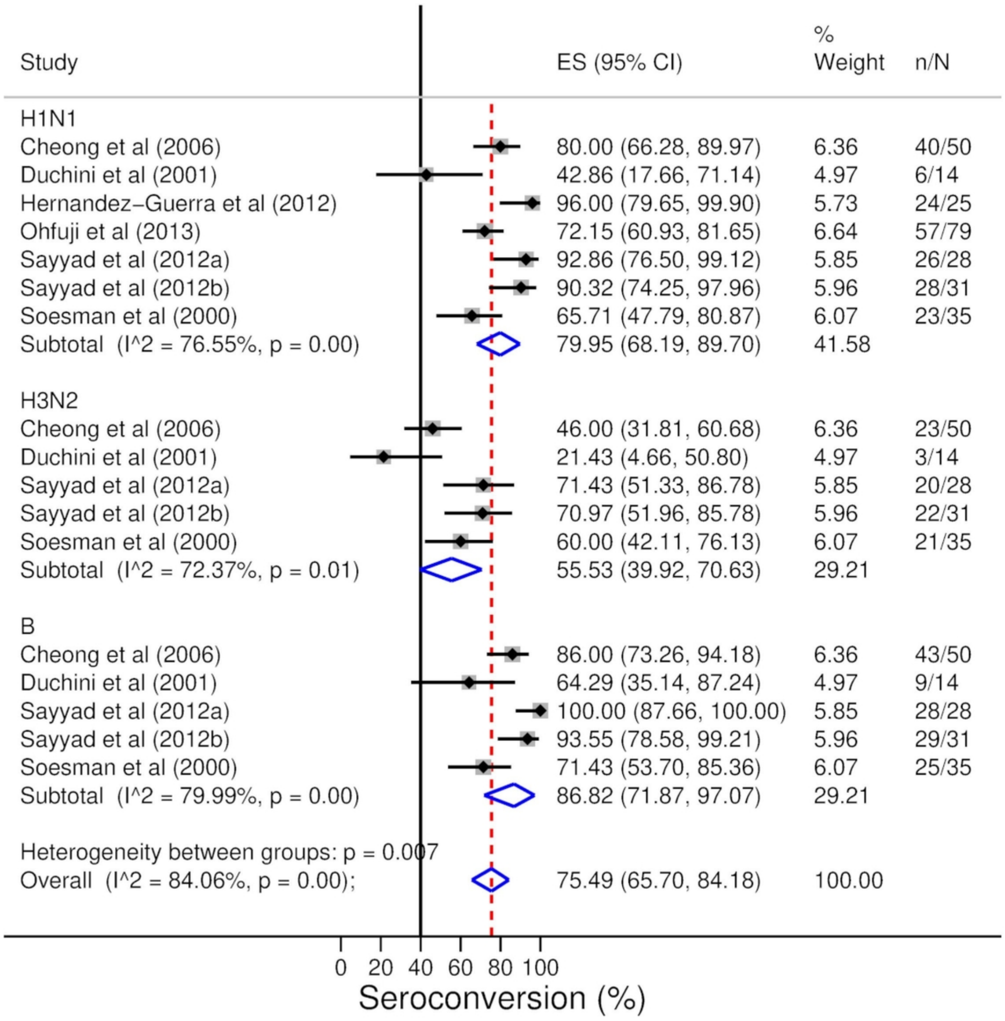

Figure 3. Serological response to influenza vaccination: seroconversion rate after vaccination. Note: Sayyad et al (2012a) includes cirrhotic patients and Sayyad et al (2012b) includes non-cirrhotic patients only.

96x97mm (300 x 300 DPI)

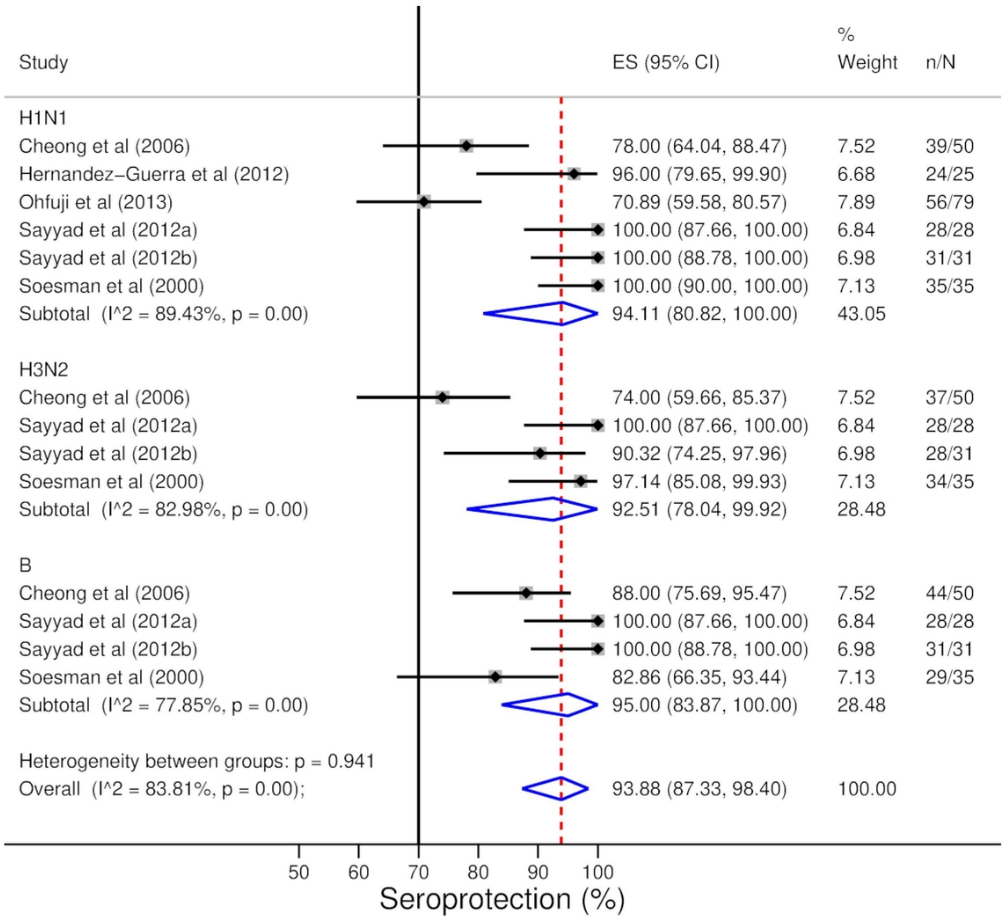

Figure 4. Serological response to influenza vaccination: seroprotection rate after vaccination. Note: Sayyad et al (2012a) includes cirrhotic patients and Sayyad et al (2012b) includes non-cirrhotic patients only.

96x87mm (300 x 300 DPI)

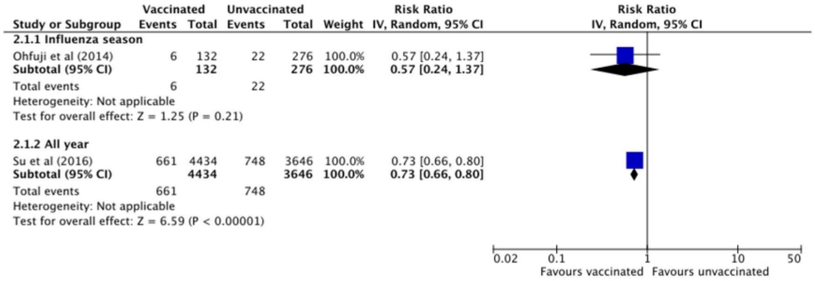

Figure 5. Effect of influenza vaccine on clinical outcomes: all-cause hospitalisation in vaccinated compared to unvaccinated liver disease patients. Note: Ohfuji et al (2014) investigated the effect of pandemic (monovalent) vaccine and Su et al (2016) of seasonal vaccine (against three or more virus subtypes).

90x47mm (300 x 300 DPI)

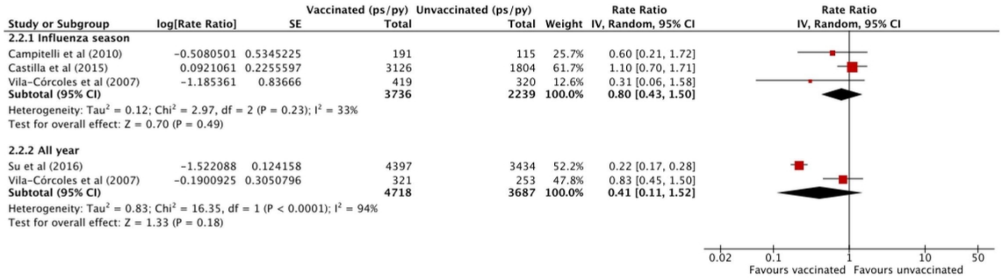

Figure 6. Effect of influenza vaccine on clinical outcomes: all-cause mortality in vaccinated compared to unvaccinated liver disease patients.

96x51mm (300 x 300 DPI)

SUPPLEMENTARY MATERIALS

Table 1. Differences between protocol and review
Table 2. Excluded test-negative case-control studies with hospitalised patients that included/are likely to have included liver patients
Table 3. Full MEDLINE (Ovid) search
Table 4. Medical subject headings used in Embase (Ovid), Pubmed and Cochrane Central Register of Controlled Trials
Table 5. Studies excluded due to uncertainty over whether the study included liver disease patients and the number of liver disease patients included
Table 6. Risk of bias in individual studies
Table 7. Risk of selective outcome reporting in the included studies
Table 8. GRADE evidence profile
Table 9. HI antibody responses in liver disease patients before and after vaccination
Figure 1. Seroprotection rate in liver disease patients before influenza vaccination
Figure 2. Sensitivity analysis: Mean difference in log HI antibody GMTs before and after vaccination in cirrhotic patients only
Figure 3. Sensitivity analysis: Seroconversion rate after vaccination in cirrhotic patients only
Figure 4. Sensitivity analysis: Seroprotection rate after vaccination in cirrhotic patients only
Figure 5. Sensitivity analysis: Seroprotection rate before vaccination in cirrhotic patients only
Figure 6. Additional analysis: Mean difference between HI antibody GMT after influenza vaccination in liver disease patients compared to healthy individuals
Figure 7. Additional analysis: Mean difference between geometric mean HI antibody titres before influenza vaccination: liver disease patients compared to healthy individuals
Figure 8. Additional analysis: Mean difference between geometric mean HI antibody titre ratio after influenza vaccination in liver disease patients compared to healthy individuals
Figure 9. Additional analysis: Probability of seroconversion after influenza vaccination in liver disease patients compared to healthy individuals
Figure 10. Additional analysis: Probability of seroprotection after influenza vaccination in liver disease patients compared to healthy individuals
Figure 11. Additional analysis: Difference in seroprotection rate before vaccination in liver disease patients compared to healthy individuals
Table 10. Risk of all-cause mortality in the general population of the included studies where liver patients formed a subgroup
References

For peer review only

Table 1. Differences between protocol and review

Review section	Protocol	Review
**Search strategy**	1. The review protocol was specified in advance as part of a wider review plan that also includes a study of the effectiveness of pneumococcal vaccine. The search strategy in this wider plan includes search terms for both influenza vaccine and pneumococcal vaccine.<br><br>2. We planned to search the Cochrane Central Register of Controlled Trials (CENTRAL), MEDLINE, EMBASE, PubMed, The Cochrane Hepato-Biliary Group Specialised Register and The Cochrane Acute Respiratory Infections Group Register of Trials.<br><br>3. Our planned search terms included filters for adults and study type.<br><br>4. We planned to complement the electronic searches by manually searching the reference lists of included studies for additional references.	1. To keep the message of the manuscript clear and concise, this manuscript focuses on influenza vaccine. The search and the results reported here include only those using search terms for influenza vaccine and not for pneumococcal vaccine.<br><br>2. MEDLINE, EMBASE and PubMed were searched as planned but instead of CENTRAL, The Cochrane Hepato-Biliary Group Specialised Register and The Cochrane Acute Respiratory Infections Group Register of Trials, only CENTRAL was searched to maintain the specificity of the search. In the preliminary searches, the number of records found in CENTRAL and the other wide focus databases was large and loosening the search terms for the two specialist sub-collections within CENTRAL was not deemed necessary (the records included in these sub-collections are also in included in CENTRAL and using the same search terms in CENTRAL and the two specialist sub-databases would result in duplicate records only).<br><br>3. In the final search, we decided to additionally filter for publication type to increase the specificity of the search.<br><br>4. In the final search, we further complemented the search by manually searching also through the recent WHO, ECDC and CDC influenza vaccine guidelines for additional references.
**Risk of bias assessment and quality of evidence**	1. We planned to use Cochrane Collaboration's tool to assess risk of bias in all studies.<br><br>2. We planned to consider age, sex, severity and aetiology of liver disease as the most important confounders in the assessment of observational studies.	1. In the review, we used Cochrane Collaboration's tool to assess risk of bias in randomized controlled trials and followed their recommendation to use the Newcastle-Ottawa scale to assess risk of bias in observational studies.<br><br>2. In the review, based on the recommendations from the peer-review of the protocol, we additionally considered as potential confounders: chronic co-morbidities and previous seasonal influenza vaccination during the same season as additional confounders in the assessment of studies that investigated the clinical effects of a pandemic (monovalent) vaccine. In assessing the quality of evidence, we considered the lack of clear effect in the influenza season as a sign of imprecision of the effect estimate of clinical outcomes.
**Data analysis**	1. We did not plan to report serological outcome measures categorised by virus subtypes.	1. In the review, we reported the serological outcome measures categorised by virus subtype as there may be differences in antibody response between them.

For peer review only

Table 2: Excluded test-negative case-control studies with hospitalised patients that included/are likely to have included liver patients

Study	Location	Cases	Controls	Liver patients
Castilla et al (2011)[1]	Spain	Patients with medically-attended influenza-like illness (MA-ILI), negative for influenza in laboratory tests.	Patients with medically-attended influenza-like illness (MA-ILI), positive for influenza in laboratory tests.	Study included hospitalised patients (34 cases, 280 controls. Eligibility criteria included patients with cirrhosis but number of patients with cirrhosis not reported.
Cheng et al (2013)[2]	Australia	Patients hospitalised for ILI, negative for influenza in laboratory tests.	Patients hospitalised for ILI, positive for influenza in laboratory tests.	Study included 38 cases and 71 controls with cirrhosis. Number of vaccinated/unvaccinated cirrhosis patients not reported.
Cheng et al (2014)[3]	Australia	Patients hospitalised for ILI, negative for influenza in laboratory tests.	Patients hospitalised for ILI, positive for influenza in laboratory tests.	Study included 38 cases with cirrhosis, number of controls with cirrhosis not reported. Number of vaccinated/unvaccinated cirrhosis patients not reported.
Choi et al (2013)[4]	South Korea	Patients with medically-attended influenza-like illness (MA-ILI), negative for influenza in laboratory tests.	Patients with medically-attended influenza-like illness (MA-ILI), positive for influenza in laboratory tests.	Study included 19 cases and 25 controls with liver disease. Number of hospitalised liver patients not reported. Number of vaccinated/unvaccinated liver patients not reported.
Choi et al (2015)[5]	South Korea	Patients with medically-attended influenza-like illness (MA-ILI), negative for influenza in laboratory tests.	Patients with medically-attended influenza-like illness (MA-ILI), positive for influenza in laboratory tests.	Study included 40 cases and 39 controls with liver disease. Number of hospitalised liver patients not reported. Number of vaccinated/unvaccinated liver patients not reported.

For peer review only

Table 3. Full MEDLINE (Ovid) search

Search concept	Search terms
Influenza vaccine	1. exp Influenza Vaccines/
	2. exp Influenza, Human/pc [Prevention & Control]
	3. ((anti?influenza or influenza or seasonal or anti?flu or flu) adj5 (vaccin* or immuni*)).mp.
	4. ((TIV or QIV or trivalent or quadrivalent or 3?valent or 4?valent) adj5 (vaccin* or immuni*)).mp.
	5. 1 or 2 or 3 or 4
Liver disease	6. exp Liver Diseases/
	7. ((liver or hepat*) adj3 disease*).mp.
	8. ("chronic liver" or "chronic hepat*").mp.
	9. cirrho*.mp.
	10. 6 or 7 or 8 or 9
Adult participants	11. exp Adult/
	12. adult.mp.
	13. (middle?aged or aged).sh.
	14. age*.tw.
	15. 11 or 12 or 13 or 14
Randomised controlled trials	16. randomized controlled trial.pt.
	17. randomi*.ab,ti.
	18. randomly.ab,ti.
	19. controlled clinical trial.pt.
	20. trial.ab,ti.
	21. groups.ab,ti.
	22. drug therapy.fs.
	23. placebo.ab,ti.
	24. 16 or 17 or 18 or 19 or 20 or 21 or 22 or 23
	25. (review or "practice guideline" or "patient education handout" or guideline or editorial or "case reports").pt.
	26. 25 not (25 and (16 and 19))
	27. Animals/
	28. Humans/
	29. 27 not (27 and 28)
	30. 24 not (26 or 29)
Case-control and cohort studies	31. Epidemiologic Studies/
	32. exp Case control studies/
	33. exp Cohort studies/
	34. Longitudinal studies/

For peer review only

	35. Follow up studies/
	36. Prospective studies/
	37. Retrospective studies/
	38. Control groups/
	39. Matched-Pair Analysis/
	40. (Case* adj5 control*).ti,ab,kw.
	41. (Case* adj5 comparison*).ti,ab,kw.
	42. Control group*.ti,ab,kw.
	43. (Cohort adj (study or studies)).ti,ab.
	44. Cohort anal*.ti,ab.
	45. (Follow up adj (study or studies)).ti,ab.
	46. (Observational adj (study or studies)).ti,ab.
	47. Longitudinal.ti,ab.
	48. Retrospective.ti,ab.
	49. Prospective.ti,ab.
	50. 31 or 32 or 33 or 34 or 35 or 36 or 37 or 38 or 39 or 40 or 41 or 42 or 43 or 44 or 45 or 46 or 47 or 48 or 49
	51. (review or "practice guideline" or "patient education handout" or guideline or editorial or "case reports").pt.
	52. 51 not (51 and (31 or 32 or 33 or 34 or 35 or 36 or 37 or 38 or 39))
	53. Animals/
	54. Humans/
	55. 53 not (53 and 54)
	56. 50 not (52 or 55)
	57. 30 or 56
	58. 5 and 10 and 15 and 57
Results: Liver disease-specific search	59. limit 58 to medline
	60. 5 and 15 and 30
Results: Influenza vaccine studies search	61. limit 60 to medline

For peer review only - http://bmjopen.bmj.com/site/about/guidelines.xhtml

Table 4. Medical subject headings used in Embase (Ovid), Pubmed and Cochrane Central Register of Controlled Trials

Database	Medical subject headings				
	**Influenza**	**Liver disease**	**Adults**	**Study type**	**Animal/Human study**
Embase (Ovid)	exp influenza vaccine/<br>exp influenza/pc [Prevention]	exp liver disease/<br>exp liver cirrhosis/	exp adult/	randomized controlled trial/<br>controlled clinical trial/<br>epidemiology/<br>exp case control study/<br>exp cohort analysis/<br>longitudinal study/<br>follow up/<br>prospective study/<br>retrospective study/<br>control group/	exp animal/<br>exp animal cell/<br>exp animal disease/<br>exp animal experiment/<br>exp animal model/<br>exp animal tissue/<br>human/
Pubmed	Influenza Vaccines[MH]<br>"influenza human/prevention and control"[MH]	liver disease[MH]	"adult"[MH]<br>"middle aged"[MH:noexp]<br>"aged"[MH:noexp]	"Epidemiologic Studies"[MH:noexp]<br>"Case-Control Studies"[MH]<br>"retrospective studies"[MH:noexp] "Control Groups"[MH:noexp]<br>"matched-pair analysis"[MH:noexp]<br>"longitudinal studies"[MH:noexp]<br>"follow-up studies"[MH:noexp] "prospective studies"[MH:noexp]<br>"cohort studies"[MH]	"animals"[MH] "humans"[MH]
Cochrane Central Register of Controlled Trials	[mh "Influenza Vaccines"]<br>[mh "Influenza, human"/PC]	[mh "Liver Diseases"]	[mh Adult]	Not relevant	Not relevant

For peer review only

Table 5. Studies excluded due to uncertainty over whether the study included liver disease patients and the number of liver disease patients included

Study	Location	Study design	Review outcomes	Reason for uncertainty and notes
Castilla et al (2012)[6]	Spain	Cohort study	Influenza illness/ILI-related hospitalisations	The inclusion criteria included patients with liver disease. to confirm the actual study population also included liver disease patients we contacted but could not reach the corresponding author.
Castilla et al (2013)[7]	Spain	Cohort study	Influenza illness/ILI-related hospitalisations	The inclusion criteria included patients with liver disease. to confirm the actual study population also included liver disease patients we contacted but could not reach the corresponding author.
DiazGranados et al (2013)[8]	United States	Randomised, controlled trial	Serological response	Study population excluded patients with known or suspected hepatitis B or C infection and patients who abused alcohol but included patients with hepatobiliary disorders: 522 in high dose vaccination group and 257 in normal dose vaccination group. We attempted to contact but could not reach the corresponding author.
Emborg et al (2012)[9]	Denmark	Cohort study	Influenza illness/ILI-related hospitalisations	The inclusion criteria included patients with liver disease. To confirm the actual study population also included liver disease patients we contacted but could not reach the corresponding author.
Mangtani et al (2004)[10]	United Kingdom	Cohort study	Acute respiratory illness-related hospitalization<br><br>Acute respiratory illness-related mortality	The inclusion criteria included patients with liver disease as part of the risk group for influenza complications. We contacted the corresponding author to confirm the actual study population also included liver disease patients. The patient data for the study had only been provided on the level of at-risk individuals not on the level of a single disease and so the author had no access to this information.
Olafsdottir et al (2018)[11]	Iceland	Cohort study	Serological response	The study population was the general population, however, the study data collected included a record of primary biliary cirrhosis. We contacted but could not reach the corresponding author to confirm the actual study population also included liver disease patients.
Simpson et al (2013)[12]	Scotland, United Kingdom	Cohort study	All-cause hospitalisation<br><br>All-cause mortality<br><br>Acute respiratory illness-related hospitalisation (pneumonia & influenza)<br><br>Acute respiratory illness-related mortality (pneumonia & influenza)	Number of patients was not reported. Liver disease patients contributed 3727 person-seasons vaccinated/5377 person-seasons unvaccinated (over 9 seasons) to the study. The study, however, also included under 18 year-olds and ≤14 year-olds contributed 4988 person-seasons vaccinated and 281550 person-seasons unvaccinated to the study. Authors were contacted to confirm the number of patients and to obtain subgroup data, however, they were unable to provide this information. It is likely liver disease was more common among over ≥18 year-olds and so the study potentially included over 400 vaccinated and over 500 unvaccinated adult liver disease patients, however, this could not be confirmed.
Wang et al (2004)[13]	Taiwan	Cohort study	All-cause hospitalisation<br><br>Acute respiratory illness-related hospitalisation (pneumonia & influenza)<br><br>Liver disease complication-related	The inclusion criteria included patients with liver disease as part of the risk group for influenza complications. It seems that the patient data for the study had only been provided on the level of at-risk individuals not on the level of a single diseases and so the authors had no access to this information. We attempted to contact but could not reach the corresponding author to confirm.

For peer review only - http://bmjopen.bmj.com/site/about/guidelines.xhtml

			hospitalisation (chronic liver disease and cirrhosis)	
Wang et al (2007)[14]	Taiwan	Cohort study	All-cause mortality<br><br>Acute-respiratory illness-related mortality (pneumonia)<br><br>Liver disease complication-related mortality (chronic liver disease)	The inclusion criteria included patients with liver disease as part of the risk group for influenza complications. It seems that the patient data for the study had only been provided on the level of at-risk individuals not on the level of a single diseases and so the authors had no access to this information. We contacted but could not reach the corresponding author to confirm.
Zivich et al (2017)[15]	United States	Cohort study	All-cause mortality	The study included at least 45 vaccinated and 41 unvaccinated liver disease patients. The study, however, also included under 18 year-old patients (121 were ≤4 year-old and 144 were ≤24 year-old. Authors were contacted to confirm the number of patients and to obtain subgroup data, however, they no longer have access to the study data and were unable to provide this information. It is likely liver disease was more common among over ≥18 year-olds and so the study potentially included over 40 adult liver disease patients in each group. This, however, could not be confirmed.

For peer review only

Table 6. Risk of bias in the included studies

A. Risk of bias in observational studies of serological outcomes

**Study**	**Selection**[a]<br>*** = low risk<br>** = moderate risk<br>- and * = high risk	**Outcome**<br>*** = low risk<br>** = moderate risk<br>- and * = high risk
Cheong et al (2006)	*** Low	* High[b,c]
Duchini et al (2001)	*** Low	** Moderate[c]
Hernández-Guerra et al (2012)	*** Low	*** Low
Ohfuji et al (2013)	*** Low	*** Low
Sayyad et al (2012)	** Moderate[d]	** Moderate[b]
Soesman et al (2000)	** Moderate[e]	** Moderate[c]

[a] Selection was assessed considering the selection of the liver disease group only.
[b] High drop-out rate.
[c] No description of independent blind assessment of outcome.
[d] Source/selection of liver patient groups not clear.
[e] Pre-vaccination influenza infection status not clear and pre-vaccination seroprotection rates high.

B. Risk of bias in observational studies of clinical outcomes

**Study**	**Selection**<br>**** = low risk<br>** and *** = moderate risk<br>- and * = high risk	**Comparability**[a]<br>**** and ***** = low risk<br>** and *** = moderate risk<br>- and * = high risk	**Outcome**<br>*** = low risk<br>** = moderate risk<br>- and * = high risk
Campitelli et al (2010)	*** Moderate[b]	Unclear[c]	*** Low
Castilla et al (2105)	**** Low	Unclear[c]	*** Low
Ohfuji et al (2014)	*** Moderate[b]	**** Moderate[d,e]	*** Low
Su et al (2016)	**** Low	***** Low	*** Low
Vila-Córcoles et al (2007)	**** Low	Unclear[c]	*** Low

[a] Comparability was considered between vaccinated and unvaccinated groups. Confounders considered were: age, sex, liver disease aetiology, liver disease severity, chronic co-morbidities and previous seasonal influenza vaccination in the same season if study vaccine pandemic vaccine.
[b] Self-reported vaccination (although in Campitelli et al 2010, vaccination was ascertained from physician billing claims, these are based on self-reporting).
[c] No information on characteristics of the vaccinated and unvaccinated groups.
[d] Sex not comparable between groups.
[e] Seasonal influenza vaccination in the same season than pandemic vaccine given to some but balance unclear between groups.

C. Risk of bias in randomised controlled studies

**Study**	**Random sequence generation**	**Allocation concealment**	**Blinding of participants and personnel**	**Blinding of outcome assessment**	**Incomplete outcome data**	**Selective reporting**	**Other bias**
Song et al (2007)	Low	Low	High[a]	Low	High[b]	Low	Unclear[c]

[a] Patients were not blinded and could not have been blinded when only the intervention group received the vaccination.
[b] High drop-out rate.
[c] Details on study funding and conflicts of interest were not provided.

For peer review only

Table 7. Risk of selective outcome reporting in the included studies

Risk of bias arising from the lack of inclusion of results when a study was excluded from a meta-analysis or not fully reported in a review because the data were unavailable.

Level of reporting: √ = Full reporting of results, × = No reporting of results, o = Partial reporting of results

Classifications and descriptions of risk of bias found in the assessment of missing or complete outcome reporting:

Clear that the outcome was measured and analysed:	Level of reporting	Risk of bias
C = Trial report states that outcome was analysed but insufficient data were presented for the trial to be included in meta-analysis or to be considered to be fully tabulated.	o	Low Risk
Clear that the outcome was measured:		
E = Clear that the outcome was measured. Judgment says outcome likely to have been analysed but not reported because of non-significant results.	×	High Risk
Unclear whether the outcome was measured:		
G = Not mentioned but clinical judgment says likely to have been measured and analysed but not reported on the basis of non-significant results.	×	High Risk
H = Not mentioned but clinical judgment says unlikely to have been measured at all.	×	Low Risk
Clear that the outcome was not measured:		
I = Clear that the outcome was not measured.	×	No Risk

For peer review only

A. Risk of selective outcome reporting in studies of serological outcomes

	Review secondary outcomes				Other outcomes reported
Study	GMT	GMTR	Seroprotection %	Seroconversion %	
Cheong et al (2006)	√	× E – High risk[a]	o C – Low risk[b]	o C – Low risk[b]	Cellular immune response to vaccine (interferon-γ secretion)
Duchini et al (2001)	√	√	× E – High risk[c]	√	None
Hernández-Guerra et al (2012)	o C – Low risk[d]	√	o C – Low risk[e]	√	Systemic adverse events, acceptance and tolerance of vaccination
Ohfuji et al (2013)	√	√	√	√	Serious adverse events
Sayyad et al (2012)	√	× E – High risk[a]	o C – Low risk[f]	o C – Low risk[f]	None
Soesman et al (2000)	√	× E – High risk[a]	√	√	Serological response (HI antibody) to a non-vaccine strain

[a] Pre- and post-vaccination GMT was measured and it is likely that their ratio was analysed but not reported.
[b] Only percentages were reported so we have assumed the highest possible number of individuals closest to the reported percentage for pre-vaccination measures and lowest possible number of individuals closest to the reported percentage for post-vaccination measures.
[c] Pre- and post-vaccination GMT was measured and seroconversion rate was analysed. It is likely that the seroprotection rate was also analysed but not reported.
[d] Pre-vaccination GMT was measured but not fully reported. It is likely this was low as only one individual had 1:40 antibody level (seroprotective level).
[e] Pre-vaccination seroprotection rate was not fully reported but the rate was low only one individual had a seroprotective level of antibodies. It was not specified in which comparison group so we have assumed it was one individual in each group.
[f] The percentages and numbers of individuals did not add up in the reported results so we have assumed the highest possible number of individuals closest to the reported percentage for pre-vaccination measures and lowest possible number of individuals closest to the reported percentage for post-vaccination measures.

For peer review only

B. Risk of selective outcome reporting in studies of clinical outcomes

	Review primary outcomes		Review secondary outcomes						Other outcomes reported
**Study**	**All-cause hospitalisation**	**All-cause mortality**	**Acute respiratory illness-related hospitalisation**	**Influenza illness/ILI-related hospitalisation**	**Liver disease complication related-hospitalisation**	**Acute respiratory illness-related mortality**	**Influenza illness/ILI-related mortality**	**Liver disease complication related-mortality**	
Campitelli et al (2010)	× H – Low risk[a]	√	o C – Low risk[b]	× I – No risk	× I – No risk	× I – No risk	× I – No risk	× I – No risk	None
Castilla et al (2105)	× I – No risk	√	× I – No risk	× I – No risk	× I – No risk	× I – No risk	× I – No risk	× I – No risk	ILI incidence
Ohfuji et al (2014)	√	× I – No risk	× I – No risk	× I – No risk	× I – No risk	× I – No risk	× I – No risk	× I – No risk	None
Song et al (2007)	× I – No risk	× G – High risk[c]	× I – No risk	× I – No risk	× I – No risk	× I – No risk	√	× I – No risk	ILI incidence, clinical manifestations of ILI, liver disease complications secondary to influenza
Su et al (2016)	√	√	× I – No risk	× I – No risk	√	× I – No risk	× I – No risk	× I – No risk	Pneumonia & Influenza incidence, heart disease related hospitalisation, respiratory failure, intensive care unit admission
Vila-Córcoles et al (2007)	× I – No risk	√	× I – No risk	× I – No risk	× I – No risk	× I – No risk	× I – No risk	× I – No risk	ILI incidence

[a] The main interest in the study was all-cause mortality and cause-specific hospitalisation outcome was used to understand the whether the mortality may have been linked to influenza. Accessing the data required an approved study protocol and it is unlikely that data to study all-cause hospitalisation was available when not included in the protocol.

[b] Study authors were unable to provide exact event numbers for this outcome as their institution's privacy policy does not allow providing data in case of five or fewer events.

[c] Authors did measure influenza-related mortality in this trial and it is likely that they also measured all-cause mortality. Authors were contacted but we were unable to obtain a response.

For peer review only

For peer review only - http://bmjopen.bmj.com/site/about/guidelines.xhtml

Table 8. GRADE evidence profile

Effectiveness of influenza vaccination in preventing all-cause hospitalisation and all-cause mortality in adults with chronic liver disease

Number of studies (design)	Limitations	Inconsistency	Indirectness	Imprecision	Publication bias	No vaccine	Vaccine	Relative risk/ Rate ratio	Control risk/ Control rate	Risk difference	Quality
All-cause hospitalisation, influenza season											
1 (Cohort study)	Serious limitations[a]	Not judged (only 1 study)	No serious indirectness	Serious imprecision[b]	Suspected[c]	22/276	6/132	0·57 (0·24, 1.37)	80 per 1000	-0·03 (-0·08, 0·01)	Very low
All-cause hospitalisation, all year											
1 (Cohort study)	No serious limitations	Not judged (only 1 study)	No serious indirectness	No serious imprecision	Suspected[c]	748/3646	661/4434	0·73 (0·66, 0·80)	205 per 1000	-0·06 (-0·07- 0·04)	Very low
All-cause mortality, influenza season											
3 (Cohort study)	Serious limitations[d]	Serious inconsistency[e]	No serious indirectness	Serious imprecision[f]	Suspected[c]	42/2239 person-seasons	66/3736 person-seasons	0·80 (0·43, 1·50)	17 per 1000 person-seasons	-0·22 (-0·85, 0·41)	Very low
All-cause mortality, all year											
2 (Cohort study)	Serious limitations[g]	No serious inconsistency	No serious indirectness	Serious imprecision[e,f]	Suspected[c]	318/3687 person-years	105/4718 person-years	0·41 (0·11, 1·52)	85 per 1000 person-years	-0·89 (-2·19, 0·42)	Very low

[a] Moderate risk of bias in selection and comparability of groups.
[b] Imprecision of the estimate with large 95% CIs.
[c] Due to study language restrictions in the review and exclusion of studies due to uncertainty of eligibility.
[d] Moderate risk of bias in selection in one study and unclear bias in comparability of groups in all studies.
[e] Study effect estimates varied largely.
[f] Control risks varied largely between studies.
[g] Unclear bias in comparability of groups in one study and uncertain timing of the vaccine effect due to Kaplan-Meier plot showing increasing difference in mortality rates outside the influenza season.

Table 9. HI antibody responses in liver disease patients before and after vaccination

Influenza A (H1N1)						
**Study**	**Pre-vaccination GMT (95% CI)**	**Post-vaccination GMT (95% CI)**	**GMTR**	**Seroconversion rate (%)**	**Pre-vaccination seroprotection rate (%)**	**Post-vaccination seroprotection rate (%)**
Cheong et al (2006)[16]	15·6 (SE ±1·8)	114·7 (SE ±4·1)	Not reported	40/50 (80%)	5/50 (10%)	39/50 (79%)
Duchini et al (2001)[17]	20·0 (10.0, 40.0)	61·0 (30·0, 125·0)	3·0 (1·7, 5·4)	6/14 (43%)	Not reported	Not reported
Hernández-Guerra et al (2012)[18]	Not reported	229·0 (55·0, 957·0)	Treated: 43·0 (10·0, 180·0)<br>Untreated: 32·0 (7·0, 137·0)	24/25 (96%)	1/25 (4%)	24/25 (96%)
Ohfuji et al (2013)[19]	8·0 (7·0, 9·0)	226·0 (69·0-743·0)	10·3 (7·2, 14·9)	57/59 (97%)	4/79 (5%)	56/79 (71%)
Sayyad et al (2012)[20]	Cirrhotic: 31·2 (SE ±2·0)<br>HBV carrier: 24·1 (SE ±2·4)	Cirrhotic: 170·7 (SE ±1·6)<br>HBV carrier: 177.7 (SE ±1·8)	Not reported	Cirrhotic: 26/28 (93%)<br>HBV carrier: 28/31 (90%)	Cirrhotic: 19/28 (68%)<br>HBV carrier: 16/31 (52%)	Cirrhotic: 28/28 (100%)<br>HBV carrier: 31/31 (100%)
Soesman et al (2000)[21]	60·0 (37·0, 97·0)	795·0 (484·0, 1303·0)	Not reported	23/35 (66%)	26/36 (72%)	35/35 (100%)
Influenza A (H3N2)						
**Study**	**Pre-vaccination GMT (95% CI)**	**Post-vaccination GMT (95% CI)**	**GMTR**	**Seroconversion rate (%)**	**Pre-vaccination seroprotection rate (%)**	**Post-vaccination seroprotection rate (%)**
Cheong et al (2006)[16]	36·8 (SE ±3·2)	75·7 (SE ±2·6)	Not reported	23/50 (46%)	13/50 (26%)	37/50 (74%)
Duchini et al (2001)[17]	87·0 (37·0, 203.0)	216·0 (95·0, 489·0)	2·4 (1·6, 3·8)	3/14 (21%)	Not reported	Not reported
Sayyad et al (2012)[20]	Cirrhotic: 36·6 (SE ±2·4)<br>HBV carrier: 24·5 (SE ±2·3)	Cirrhotic: 121·5 (SE ±1·9)<br>HBV carrier: 111·1 (SE ±2·4)	Not reported	Cirrhotic: 20/28 (71%)<br>HBV carrier: 22/31 (71%)	Cirrhotic: 20/28 (71%)<br>HBV carrier: 16/31 (52%)	Cirrhotic: 28/28 (100%)<br>HBV carrier: 28/31 (90%)
Soesman et al (2000)[21]	34·0 (19·0, 61·0)	331·0 (207·0, 531·0)	Not reported	21/35 (60%)	21/36 (58%)	34/35 (97%)
Influenza B						
**Study**	**Pre-vaccination GMT (95% CI)**	**Post-vaccination GMT (95% CI)**	**GMTR**	**Seroconversion rate (%)**	**Pre-vaccination seroprotection rate (%)**	**Post-vaccination seroprotection rate (%)**
Cheong et al (2006)[16]	17·4 (SE ±1·7)	82·3 (SE ±2·3)	Not reported	43/50 (86%)	5/50 (10%)	44/50 (88%)

For peer review only

Duchini et al (2001)[17]	18·0 (9·0, 35·0)	119·0 (48·0, 292·0)	6·0 (2·6, 19·0)	9/14 (64%)	Not reported	Not reported
Sayyad et al (2012)[20]	Cirrhotic: 27·1 (SE ±2·1)<br>HBV carrier: 14·9 (SE ±2·1)	Cirrhotic: 210·6 (SE ±1·7)<br>HBV carrier: 134·3 (SE ±2·0)	Not reported	Cirrhotic: 28/28 (100%)<br>HBV carrier: 29/31 (94%)	Cirrhotic: 18/28 (64%)<br>HBV carrier: 6/31 (19%)	Cirrhotic: 28/28 (100%)<br>HBV carrier: 31/31 (100%)
Soesman et al (2000)[21]	12·0 (8·0, 19·0)	110·0 (61·0, 199·0)	Not reported	25/35 (71%)	10/36 (28%)	29/35 (83%)

For peer review only

Figure 1. Seroprotection rate in liver disease patients before influenza vaccination

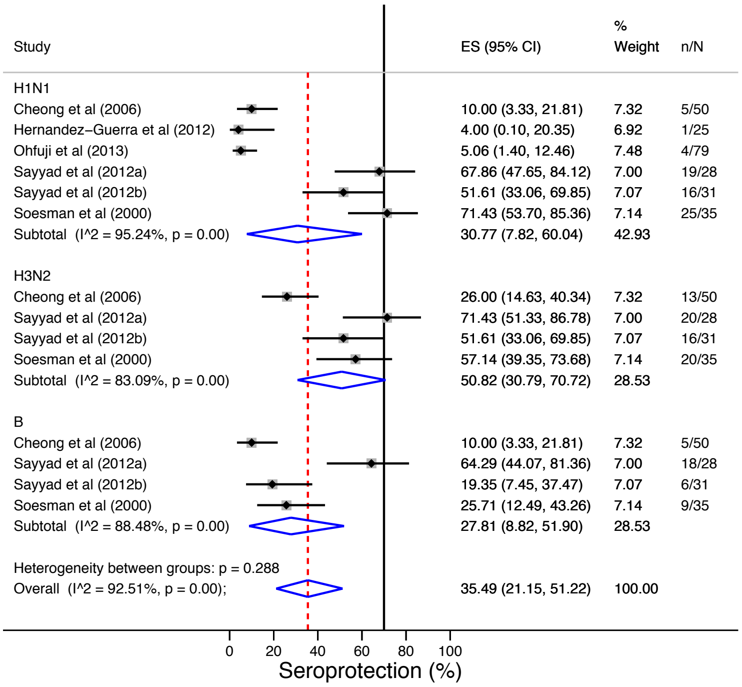

Figure 2. Sensitivity analysis: Mean difference in log HI antibody GMTs before and after vaccination in cirrhotic patients only

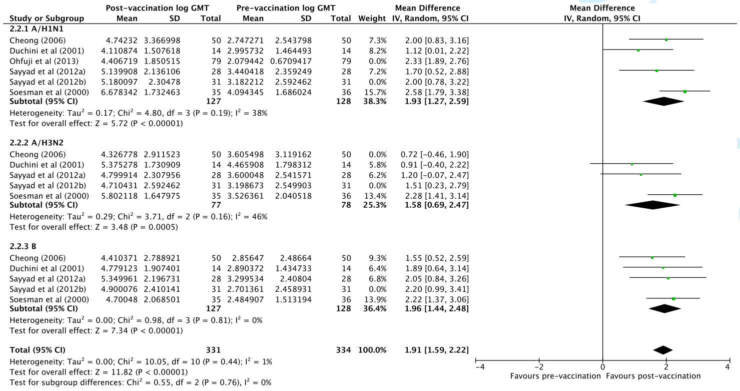

Note: Liver patient results from the studies with non-cirrhotic patients only and studies with a mix of cirrhotic and non-cirrhotic patients are visible but do not affect the outcome of analysis. Only the results from the studies with cirrhotic patients Cheong et al (2006), Duchini et al (2001) Sayyad et al (2012a) and Soesman are pooled in this analysis. Sayyad et al (2012b) includes non-cirrhotic patients only.

For peer review only - http://bmjopen.bmj.com/site/about/guidelines.xhtml

Figure 3. Sensitivity analysis: Seroconversion rate after vaccination in cirrhotic patients only

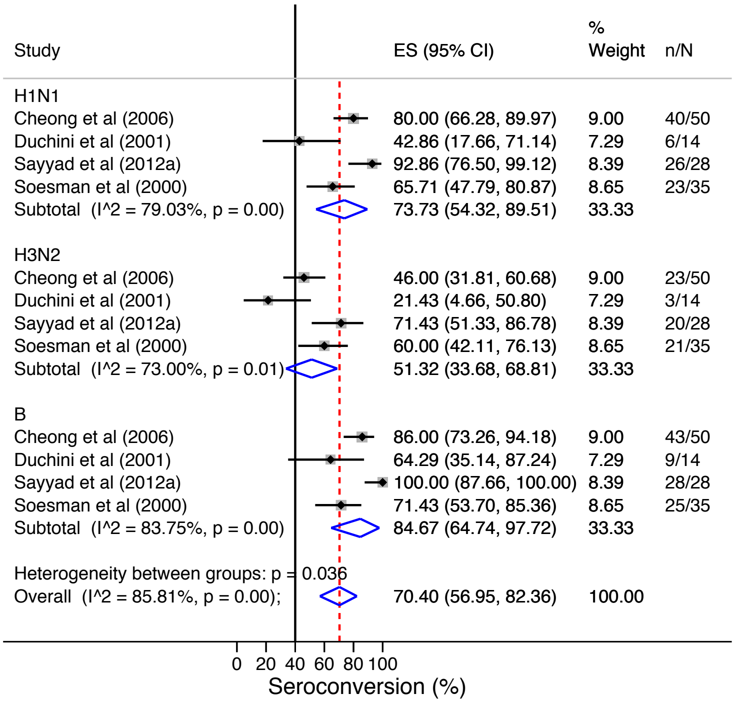

Figure 4. Sensitivity analysis: Seroprotection rate after vaccination in cirrhotic patients only

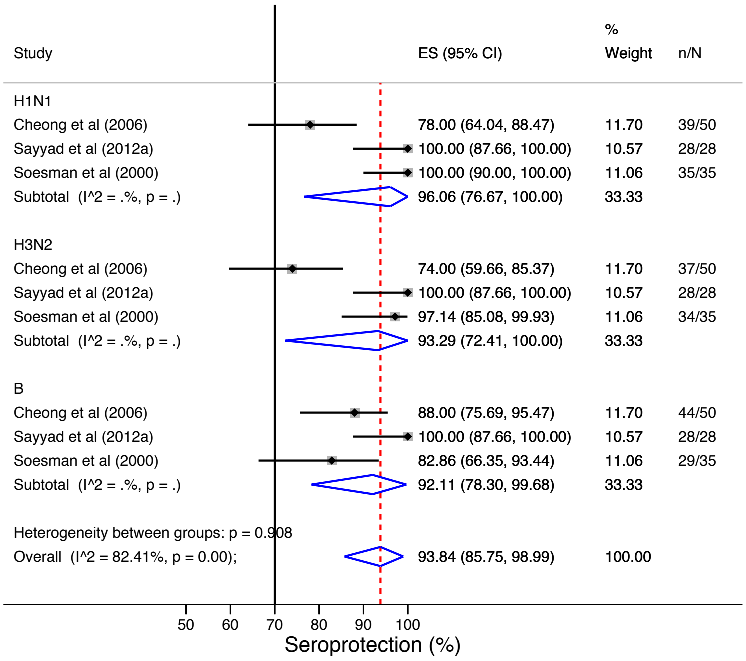

For peer review only - http://bmjopen.bmj.com/site/about/guidelines.xhtml

Figure 5. Sensitivity analysis: Seroprotection rate before vaccination in cirrhotic patients only

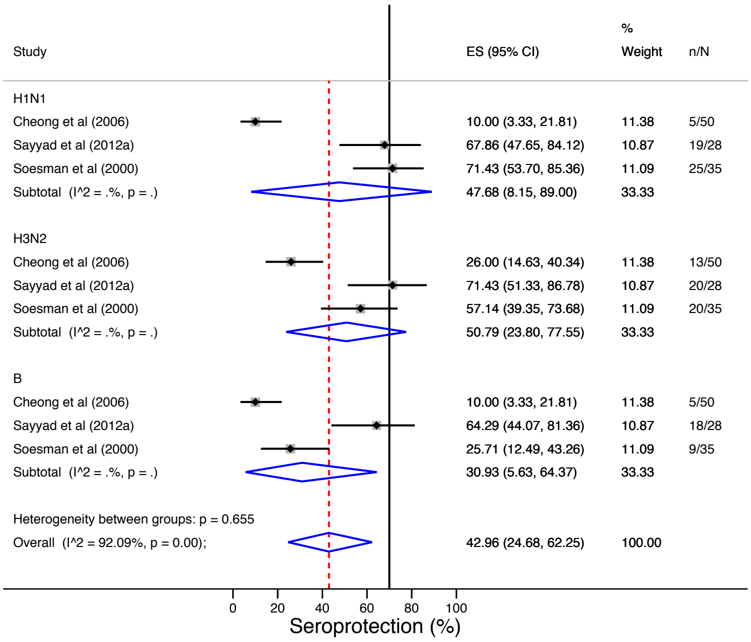

Figure 6. Additional analysis: Mean difference between HI antibody GMT after influenza vaccination in liver disease patients compared to healthy individuals

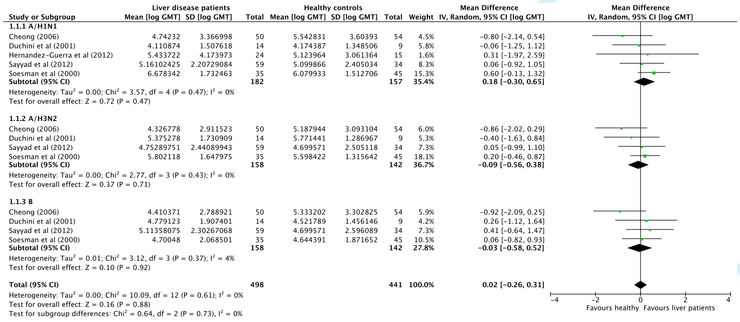

Note: Results from the non-cirrhotic and cirrhotic patient groups in Sayyad et al (2012) have been combined using the formulae recommended in Cochrane Handbook [22].

Figure 7. Additional analysis: Mean difference between geometric mean HI antibody titres before influenza vaccination in liver disease patients compared to healthy individuals

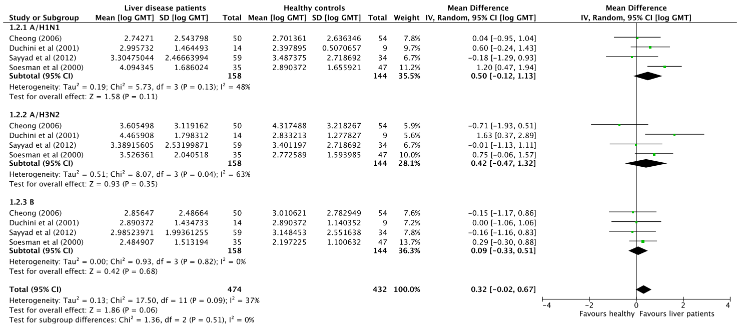

Note: Results from the non-cirrhotic and cirrhotic patient groups in Sayyad et al (2012) have been combined using the formulae recommended in Cochrane Handbook [22].

For peer review only - http://bmjopen.bmj.com/site/about/guidelines.xhtml

Figure 8. Additional analysis: Mean difference between geometric mean HI antibody titre ratio after influenza vaccination in liver disease patients compared to healthy individuals

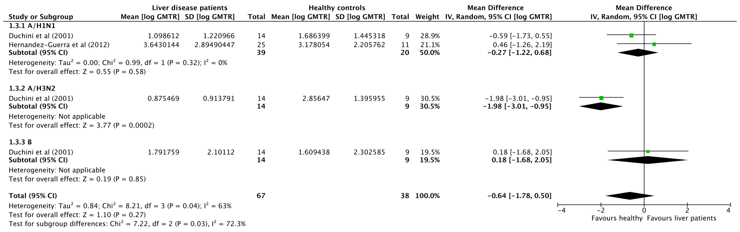

Note: Results from the non-cirrhotic and cirrhotic patient groups in Sayyad et al (2012) have been combined using the formulae recommended in Cochrane Handbook [22].

Figure 9. Additional analysis: Probability of seroconversion after influenza vaccination in liver disease patients compared to healthy individuals

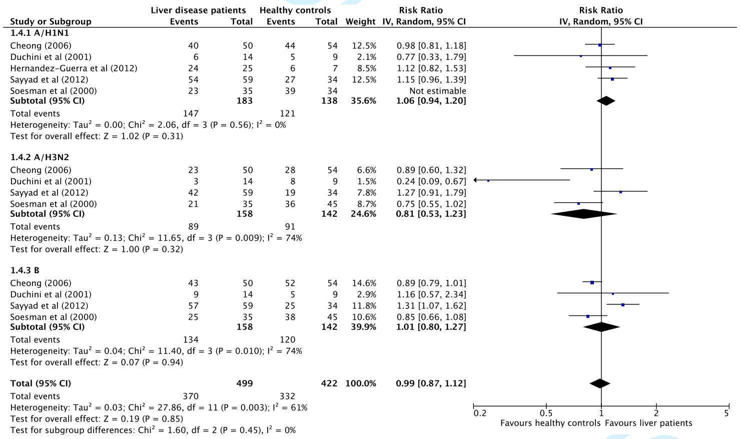

Note: Results from the non-cirrhotic and cirrhotic patient groups in Sayyad et al (2012) have been combined using the formulae recommended in Cochrane Handbook [22].

Figure 10. Additional analysis: Probability of seroprotection after influenza vaccination in liver disease patients compared to healthy individuals

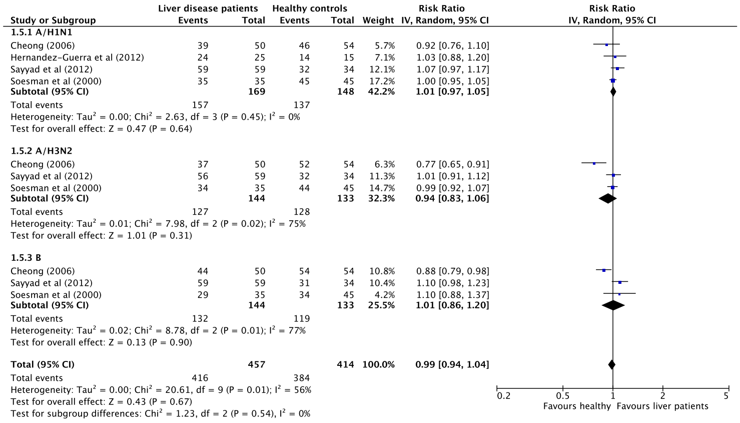

Note: Results from the non-cirrhotic and cirrhotic patient groups in Sayyad et al (2012) have been combined using the formulae recommended in Cochrane Handbook [22].

For peer review only - http://bmjopen.bmj.com/site/about/guidelines.xhtml

Figure 11. Additional analysis: Difference in seroprotection rate before vaccination in liver disease patients compared to healthy individuals

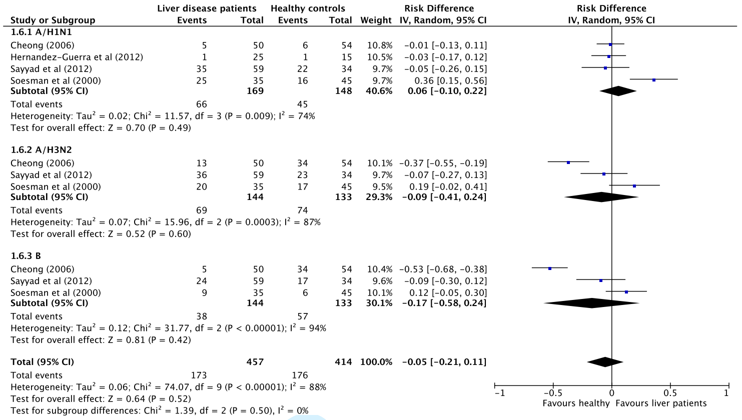

Note: Results from the non-cirrhotic and cirrhotic patient groups in Sayyad et al (2012) have been combined using the formulae recommended in Cochrane Handbook [22].

Table 10. Risk of all-cause mortality in the general population of the included studies where liver patients formed a subgroup

Study	Number of vaccinated/unvaccinated	Adjusted HR (95%CI) in the influenza season	Notes
Campitelli et al (2010)	14512/11410	0·61 (0·47–0·79)	Protective effect was present also outside the influenza season indicating presence of frailty bias.
Castilla et al (2015)	60400/42756[a]	0·84 (0·76–0·93)	Effect outside the seasons was not protective
Vila-Córcoles et al (2007	6051/5189	0·63 (0·54–0·74)	Effect outside the seasons was not protective

[a] These numbers are for the first of the two seasons studied. Number of patients and the % of vaccinated were similar for the second season.

For peer review only - http://bmjopen.bmj.com/site/about/guidelines.xhtml

For peer review only

References

1 Castilla J, Morán J, Martínez-Artola V, *et al*. Effectiveness of the monovalent influenza A(H1N1) 2009 vaccine in Navarre, Spain, 2009–2010: Cohort and case-control study. *Vaccine* 2011;**29**:5919–24. doi:10.1016/j.vaccine.2011.06.063

2 Cheng AC, Brown SG, Waterer GW, *et al*. Influenza epIdemIology, vaccIne coverage and vaccIne effectIveness in sentInel AustralIan hospItals in 2012: the Influenza ComplIcatIons Alert Network (FluCAN). *Commun Dis Intell* 2013;**37**:E246-252.

3 Cheng AC, Dwyer DE, Holmes M, *et al*. Influenza epIdemIology, vaccIne coverage and vaccIne effectIveness in sentInel AustralIan hospItals in 2013: the Influenza ComplIcatIons Alert Network. *Commun Dis Intell* 2014;**38**:E143-149.

4 Choi WS, Noh Y, Seo Y bin, *et al*. Case-Control Study of the Effectiveness of the 2010-2011 Seasonal Influenza Vaccine for Prevention of Laboratory-Confirmed Influenza Virus Infection in the Korean Adult Population. *Clin Vaccine Immunol* 2013;**20**:877–81. doi:10.1128/CVI.00009-13

5 Choi WS, Noh JY, Baek JH, *et al*. Suboptimal Effectiveness of the 2011 – 2012 Seasonal Influenza Vaccine in Adult Korean Populations. *PLoS One* 2015;**10**:e0098716. doi:10.1371/journal.pone.0098716

6 Castilla J, Martinez-Artola V, Salcedo E, *et al*. Vaccine effectiveness in preventing influenza hospitalizations in Navarre, Spain, 2010-2011: Cohort and case-control study. Vaccine. 2012;**30**:195–200.

7 Castilla J, Godoy P, Dominguez A, *et al*. Influenza vaccine effectiveness in preventing outpatient, inpatient, and severe cases of laboratory-confirmed influenza. Clin. Infect. Dis. 2013;**57**:167–75. doi:10.1093/cid/cit194

8 DiazGranados CA, Dunning AJ, Jordanov E, *et al*. High-dose trivalent influenza vaccine compared to standard dose vaccine in elderly adults: Safety, immunogenicity and relative efficacy during the 2009-2010 season. *Vaccine* 2013;**31**:861–6. doi:http://dx.doi.org/10.1016/j.vaccine.2012.12.013

9 Emborg H-D, Krause TG, Hviid A, *et al*. Effectiveness of vaccine against pandemic influenza A/H1N1 among people with underlying chronic diseases: Cohort study, Denmark, 2009-10. BMJ. 2012;**344**:d7901. doi:https://doi.org/10.1136/bmj.d7901

10 Mangtani P, Cumberland P, Hodgson CR, *et al*. A cohort study of the effectiveness of influenza vaccine in older people, performed using the United Kingdom general practice research database. J. Infect. Dis. 2004;**190**:1–10.

11 Olafsdottir TA, Alexandersson KF, Sveinbjornsson G, *et al*. Age and Influenza-Specific Pre-Vaccination Antibodies Strongly Affect Influenza Vaccine Responses in the Icelandic Population whereas Disease and Medication Have Small Effects. *Front Immunol* 2017;**8**:1872. doi:10.3389/fimmu.2017.01872

12 Simpson C, Lone N, Kavanagh K, *et al*. Seasonal Influenza Vaccine Effectiveness (SIVE): an observational retrospective cohort study - exploitation of a unique community-based national-linked database to determine the effectiveness of the seasonal trivalent influenza vaccine (Structured abstrac. *Heal Technol Assess Database* 2013;**1**.

13 Wang CS, Wang ST, Lai Lee CT, *et al*. Reducing major cause-specific hospitalization rates and shortening hospital stays after influenza vaccination. *Clin Infect Dis* 2004;**39**:1604–10. doi:http://dx.doi.org/10.1086/425323

14 Wang CS, Wang ST, Lai CT, *et al*. Impact of influenza vaccination on major cause-specific mortal. Vaccine. 2007;**25**:1196–203.

15 Zivich PN, Tatham L, Lung K, *et al*. Influenza vaccination status and outcomes among influenza-associated hospitalizations in Columbus, Ohio (2012-2015). *Epidemiol Infect* 2017;**145**:3284–93. doi:10.1017/S0950268817002163

16 Cheong H-J, Song J-Y, Park J-W, *et al*. Humoral and cellular immune responses to influenza vaccine in patients with advanced cirrhosis. *Vaccine* 2006;**24**:2417–22. doi:10.1016/j.vaccine.2005.11.064

17 Duchini A, Hendry RM, Nyberg LM, *et al*. Immune response to influenza vaccine in adult liver transplant recipients. *Liver Transplant* 2001;**7**:311–3. doi:10.1053/jlts.2001.23010

18 Hernandez-Guerra M, Gonzalez-Mendez Y, de Molina P, *et al*. Immunogenicity and Acceptance of Influenza A (H1N1) Vaccine in a Cohort of Chronic Hepatitis C Patients Receiving Pegylated-Interferon Treatment. *PLoS One* 2012;**7**:e48610. doi:10.1371/journal.pone.0048610

19 Ohfuji S, Fukushima W, Tamori A, *et al*. Immunogenicity of influenza A(H1N1)pdm09 vaccine and the associated factors on lowered immune response in patients with hepatitis C. *Influenza Other Respi Viruses* 2013;**7**:456–65. doi:10.1111/j.1750-2659.2012.00424.x

20 Sayyad B, Alavian SM, Najafi F, *et al*. Efficacy of Influenza Vaccination in Patients with Cirrhosis and Inactive Carriers of Hepatitis B Virus Infection. *Iran Red Crescent Med J* 2012;**14**:623–30.

21 Soesman NMR, Rimmelzwaan GF, Nieuwkoop NJ, *et al*. Efficacy of influenza vaccination in adult liver transplant recipients. *J Med Virol* 2000;**61**:85–93. doi:https://doi.org/10.1002/(SICI)1096-

For peer review only - http://bmjopen.bmj.com/site/about/guidelines.xhtml

9071(200005)61:1<85::AID-JMV14>3.0.CO;2-H
22 The Cochrane Collaboration. Cochrane Handbook for Systematic Reviews of Interventions. Version 5.1.0 [updated March 2011]. 2011.http://handbook-5-1.cochrane.org/ (accessed 25 Jul 2018).

For peer review only

For peer review only - http://bmjopen.bmj.com/site/about/guidelines.xhtml

PRISMA 2009 Checklist

Section/topic	#	Checklist item	Reported on page #
**TITLE**			
Title	1	Identify the report as a systematic review, meta-analysis, or both.	p. 1
**ABSTRACT**			
Structured summary	2	Provide a structured summary including, as applicable: background; objectives; data sources; study eligibility criteria, participants, and interventions; study appraisal and synthesis methods; results; limitations; conclusions and implications of key findings; systematic review registration number.	p. 2-3
**INTRODUCTION**			
Rationale	3	Describe the rationale for the review in the context of what is already known.	p. 5-7
Objectives	4	Provide an explicit statement of questions being addressed with reference to participants, interventions, comparisons, outcomes, and study design (PICOS).	p. 6-7
**METHODS**			
Protocol and registration	5	Indicate if a review protocol exists, if and where it can be accessed (e.g., Web address), and, if available, provide registration information including registration number.	p. 7
Eligibility criteria	6	Specify study characteristics (e.g., PICOS, length of follow-up) and report characteristics (e.g., years considered, language, publication status) used as criteria for eligibility, giving rationale.	p. 7-8
Information sources	7	Describe all information sources (e.g., databases with dates of coverage, contact with study authors to identify additional studies) in the search and date last searched.	p. 8-9
Search	8	Present full electronic search strategy for at least one database, including any limits used, such that it could be repeated.	p. 8-9, Supplementary materials p.4-6 (Tables 3 and 4)
Study selection	9	State the process for selecting studies (i.e., screening, eligibility, included in systematic review, and, if applicable, included in the meta-analysis).	p. 9
Data collection process	10	Describe method of data extraction from reports (e.g., piloted forms, independently, in duplicate) and any processes for obtaining and confirming data from investigators.	p. 9-10
Data items	11	List and define all variables for which data were sought (e.g., PICOS, funding sources) and any assumptions and simplifications made.	p. 9-10
Risk of bias in individual studies	12	Describe methods used for assessing risk of bias of individual studies (including specification of whether this was done at the study or outcome level), and how this information is to be used in any data synthesis.	p. 10

For peer review only - http://bmjopen.bmj.com/site/about/guidelines.xhtml

PRISMA 2009 Checklist

Summary measures	13	State the principal summary measures (e.g., risk ratio, difference in means).	p. 11-12
Synthesis of results	14	Describe the methods of handling data and combining results of studies, if done, including measures of consistency (e.g., I^2) for each meta-analysis.	p. 12-12

Section/topic	#	Checklist item	Reported on page #
Risk of bias across studies	15	Specify any assessment of risk of bias that may affect the cumulative evidence (e.g., publication bias, selective reporting within studies).	p. 12-13
Additional analyses	16	Describe methods of additional analyses (e.g., sensitivity or subgroup analyses, meta-regression), if done, indicating which were pre-specified.	p. 12
**RESULTS**			
Study selection	17	Give numbers of studies screened, assessed for eligibility, and included in the review, with reasons for exclusions at each stage, ideally with a flow diagram.	p. 13-14 (Figure 1)
Study characteristics	18	For each study, present characteristics for which data were extracted (e.g., study size, PICOS, follow-up period) and provide the citations.	p. 14-21 (Tables 1 and 2)
Risk of bias within studies	19	Present data on risk of bias of each study and, if available, any outcome level assessment (see item 12).	p. 14, Supplementary materials p. 9 (Table 6)
Results of individual studies	20	For all outcomes considered (benefits or harms), present, for each study: (a) simple summary data for each intervention group (b) effect estimates and confidence intervals, ideally with a forest plot.	p. 17-21, (Figures 2-6), Supplementary materials p.16 (Figure 1)
Synthesis of results	21	Present results of each meta-analysis done, including confidence intervals and measures of consistency.	p. 17-21 (Figures 2-6), Supplementary materials p.16 (Figure 1)
Risk of bias across studies	22	Present results of any assessment of risk of bias across studies (see Item 15).	p. 14, Supplementary materials p.10-

For peer review only

PRISMA 2009 Checklist

			13 (Tables 7 and 8)
Additional analysis	23	Give results of additional analyses, if done (e.g., sensitivity or subgroup analyses, meta-regression [see Item 16]).	p. 21-22, Supplementary materials p. 16-20 (Figures 2-11)
**DISCUSSION**			
Summary of evidence	24	Summarize the main findings including the strength of evidence for each main outcome; consider their relevance to key groups (e.g., healthcare providers, users, and policy makers).	p. 23-24 (Table 3), Supplementary materials p.13 (Table 8)
Limitations	25	Discuss limitations at study and outcome level (e.g., risk of bias), and at review-level (e.g., incomplete retrieval of identified research, reporting bias).	p. 24-26
Conclusions	26	Provide a general interpretation of the results in the context of other evidence, and implications for future research.	p. 27
**FUNDING**			
Funding	27	Describe sources of funding for the systematic review and other support (e.g., supply of data); role of funders for the systematic review.	p. 13, 28

From: Moher D, Liberati A, Tetzlaff J, Altman DG, The PRISMA Group (2009). Preferred Reporting Items for Systematic Reviews and Meta-Analyses: The PRISMA Statement. PLoS Med 6(7): e1000097. doi:10.1371/journal.pmed1000097

For more information, visit: **www.prisma-statement.org**.

For peer review only
For peer review only - http://bmjopen.bmj.com/site/about/guidelines.xhtml

**MOOSE CHECKLIST ***		
Source: https://www.editorialmanager.com/jognn/account/MOOSE.pdf<br>*Modified from Stroup DF, Berlin JA, Morton SC, Olkin I, Williamson GD, Rennie D, et al. Meta-analysis of observational studies in epidemiology: a proposal for reporting. Meta-analysis Of Observational Studies in Epidemiology (MOOSE) group. JAMA 2000;283:2008–12. Copyrighted © 2000, American Medical Association. All rights reserved.		
**Title**	Identify the study as a meta-analysis (or systematic review)	p. 1
**Abstract**	Use the journal's structured format	p. 2-3
**Introduction**	**Present:**	
	The clinical problem	p. 5-7
	The hypothesis	p. 6-7
	A statement of objectives that includes the study population, the condition of interest, the exposure or intervention, and the outcome(s) considered	p. 6-7
**Sources**	**Describe:**	
	Qualifications of searchers (eg, librarians and investigators)	p. 28
	Search strategy, including time period included in the synthesis and keywords	p. 8-9, Supplementary materials p.4-6 (Tables 3 and 4)
	Effort to include all available studies, including contact with authors	p. 9-10
	Databases and registries searched	p. 8-9
	Search software used, name and version, including special features used (eg, explosion)	p. 8-9, Supplementary materials p.4-6 (Tables 3 and 4)
	Use of hand searching (eg, reference lists of obtained articles)	p. 9
	List of citations located and those excluded, including justification	p. 13-14 (Figure 1, Tables 1 and 2), Supplementary

		materials p. 2, 7-8 (Table 2 and 5)
	Method of addressing articles published in languages other than English	p. 8
	Method of handling abstracts and unpublished studies	p. 9
	Description of any contact with authors	p. 9-10
**Study selection**	**Describe:**	
	Types of study designs considered	p. 7-8
	Relevance or appropriateness of studies gathered for assessing the hypothesis to be tested	p. 7-8
	Rationale for the selection and coding of data (eg, sound clinical principles or convenience)	p. 7-12
	Documentation of how data were classified and coded (eg, multiple raters, blinding, and interrater reliability)	p. 9-10
	Assessment of confounding (eg, comparability of cases and controls in studies where appropriate	p. 10-11
	Assessment of study quality, including blinding of quality assessors; stratification or regression on possible predictors of study results	p. 12-13
	Assessment of heterogeneity	p. 12
	Statistical methods (eg, complete description of fixed or random effects models, justification<br>of whether the chosen models account for predictors of study results, dose-response models,<br>or cumulative meta-analysis) in sufficient detail to be replicated	p. 11-12
**Results**	**Present:**	
	A graph summarizing individual study estimates and the overall estimate	p. 17-21, (Figures 2-6), Supplementary materials p.16 (Figure 1)
	A table giving descriptive information for each included study	p. 14-21 (Tables 1 and 2)

For peer review only

	Results of sensitivity testing (eg, subgroup analysis)	p. 21-22, Supplementary materials p. 16-20 (Figures 2-11)
	Indication of statistical uncertainty of findings	p. 17-21, (Figures 2-6), Supplementary materials p. 16-20 (Figures 1-11)
**Discussion**	**Discuss:**	
	Strengths and weaknesses	p. 24-26
	Potential biases in the review process (eg, publication bias)	p. 24-26, Supplementary materials p. 2, 10-13 (Tables 1, 7 and 8)
	Justification for exclusion (eg, exclusion of non–English-language citations)	p. 26
	Assessment of quality of included studies	p. 23-24 (Table 3), Supplementary materials p.13 (Table 8)
	Consideration of alternative explanations for observed results	p. 24
	Generalization of the conclusions (ie, appropriate for the data presented and within the domain of the literature review)	p. 24-25
	Guidelines for future research	p. 27
	Disclosure of funding source	p. 13, 28

For peer review only

The effectiveness of influenza vaccines in adults with chronic liver disease: a systematic review and meta-analysis

Journal:	*BMJ Open*
Manuscript ID	bmjopen-2019-031070.R2
Article Type:	Research
Date Submitted by the Author:	13-Aug-2019
Complete List of Authors:	Härmälä, Suvi; University College London, Institute of Health Informatics<br>Parisinos, Constantinos; University College London, Institute of Health Informatics<br>Shallcross, Laura; University College London, Institute of Health Informatics<br>O'Brien, Alastair; UCL, Division of Medicine<br>Hayward, Andrew; University College London, Institute of Epidemiology and Health Care
**Primary Subject Heading**:	Gastroenterology and hepatology
Secondary Subject Heading:	Infectious diseases, Public health
Keywords:	Cirrhosis, Vaccine effectiveness, Hospitalisation, Seroprotection, Liver disease, Influenza vaccine

SCHOLARONE™
Manuscripts

Title: The effectiveness of influenza vaccines in adults with chronic liver disease: a systematic review and meta-analysis

Authors:

Suvi Härmälä, Institute of Health Informatics, University College London, 222 Euston Road, London NW1 2DA, United Kingdom. Email: suvi.harmala.14@ucl.ac.uk

Constantinos A. Parisinos, Institute of Health Informatics, University College London, 222 Euston Road, London NW1 2DA, United Kingdom. Email: c.parisinos@ucl.ac.uk

Laura Shallcross, Institute of Health Informatics, University College London, 222 Euston Road, London NW1 2DA, United Kingdom. Email: l.shallcross@ucl.ac.uk

Alastair O'Brien, Division of Medicine, University College London, Rayne Building, 5 University Street, London WC1E 6JJ, United Kingdom. Email: a.o'brien@ucl.ac.uk

Andrew Hayward, Institute of Epidemiology and Health Care, University College London, 1-19 Torrington Place, London WC1E 7HB, United Kingdom. Email: a.hayward@ucl.ac.uk

Correspondence to: Suvi Härmälä, Institute of Health Informatics, University College London, 222 Euston Road, London NW1 2DA, United Kingdom. Tel: +44 (0) 782 430 3142. Email: suvi.harmala.14@ucl.ac.uk

For peer review only

ABSTRACT

Objectives: Patients with liver disease frequently require hospitalisation with infection often the trigger. Influenza vaccination is an effective infection prevention strategy in healthy and elderly but is often perceived less beneficial in liver patients. We investigated whether influenza vaccination triggered serological response and prevented hospitalisation and death in liver disease.

Design: Systematic review and meta-analysis.

Data sources: MEDLINE, EMBASE, Pubmed and CENTRAL up to January 2019.

Eligibility criteria: Randomised or observational studies of the effects of influenza vaccine in adults with liver disease.

Data extraction and synthesis: Two reviewers screened studies, extracted data and assessed risk of bias and quality of evidence. Primary outcomes were all-cause hospitalisation and mortality. Secondary outcomes were cause-specific hospitalisation and mortality, and serological vaccine response. Random effects meta-analysis was used to estimate pooled effects of vaccination.

Results: We found 10649 unique records, 286 were eligible for full-text review and 12 were included. Most patients had viral liver disease. All studies were of very low quality. Both non-cirrhotic and cirrhotic patients mounted an antibody response and influenza vaccination was associated with a reduction in risk of hospital admission from

For peer review only

205/1000 to 149/1000 (risk difference -0.06, 95% CI -0.07, -0.04) in patients with viral liver disease. Vaccinated patients were 27% less likely to be admitted to hospital compared to unvaccinated patients (risk ratio 0.73, 95% CI 0.66, 0.80). No effect against all-cause or cause-specific mortality or cause-specific hospitalisation was found.

Conclusions: The low quantity and quality of the evidence means that the protective vaccine effect may be uncertain. Considering the high risk of serious health outcomes from influenza infection in liver patients and the safety and low cost of vaccination, overall, the potential benefits of seasonal vaccination both to patients and the healthcare systems are likely to outweigh the costs and risks associated with vaccination.

PROSPERO: CRD42017067277

Keywords: cirrhosis, hospitalisation, influenza vaccine, liver disease, seroprotection, vaccine effectiveness

Abbreviations: CI, confidence interval; GMT, geometric mean titre; GMTR, geometric mean titre ratio; GRADE, Grading of Recommendations Assessment, Development and Evaluation; HBV, hepatitis B virus; HCV, hepatitis C virus; HI, hemagglutinisation-inhibition; ILI, influenza-like illness; MD, mean difference; NOS, Newcastle-Ottawa scale; ORBIT, Outcome Reporting Bias in Trials; RR, risk ratio; WHO, World Health Organization

For peer review only

STRENGTHS AND LIMITATIONS OF THIS STUDY

- The review was conducted following established guidelines and a prospectively specified protocol
- The review focused on all-cause outcomes to reduce the risk of underestimating the vaccine impact that exists due to potential difficulties in identifying and accurately recording the cause of hospitalisation and death in patients with an underlying chronic disease.
- To reduce the risk of missing relevant studies and relevant data and to increase the objectivity of the review, two independent reviewers screened the studies, extracted data and assessed the risk of bias and quality of evidence.
- Differences in studies included in meta-analyses, publication bias, restriction on the publication language and varying response to queries about data on liver disease subgroups may have affected the effects observed in this review.
- Inclusion of non-randomised studies lowers the quality of evidence and limits the conclusions that can be drawn.

For peer review only

INTRODUCTION

Greater than 45 million people globally, including more than 8 million people in the US and Europe, are affected by chronic liver disease[1]. The prevalence of liver disease is increasing, driven by obesity and alcohol consumption [2],

The progression of liver disease is associated with immune dysregulation[3–6] and complications from common acute infections such as influenza cause significant morbidity and mortality. A 2-fold increased risk of hospital admission for influenza was observed in liver disease patients in 19 hospitals in Russia, Turkey, China, and Spain during the 2013/2014 season[7]. Similarly, an analysis of data on laboratory-confirmed influenza cases collected from several World Health Organization (WHO) member states during the 2009 influenza A (H1N1) pandemic found liver disease patients to have a greater than 5-fold increased risk of influenza-related hospitalisation and over 17-fold increased risk of death compared to that of healthy individuals[8]. Influenza infection, while not directly targeting the liver, may cause collateral liver damage[9] and trigger hepatic decompensation in liver disease[10].

Influenza vaccine introduces a whole or part of the influenza virion into the body stimulating antibody production against the virus, similar to an actual infection. In healthy adult and elderly populations, seasonal influenza vaccination is an effective way to prevent influenza infection[11,12]. People with liver disease, due to the high risk of severe health complications from influenza infection, are often a target of influenza vaccination policies[13] but randomised trials on vaccine effectiveness in liver patients are lacking as withholding the vaccine may place patients' safety and health at risk. For

For peer review only

example, a previous systematic review of randomised trials of influenza vaccine effectiveness in patients with chronic diseases, conducted in 2011[14], identified only one study in liver patients. Observational studies offer an alternative way of investigating vaccine effectiveness but without randomisation of patients the results are vulnerable to confounding and bias, in particular frailty bias. Frailty bias exist when some or all of the reduction in the risk of health complications may be due to the preferential receipt of vaccine by individuals who are in general more healthy. One solution to minimise the effect of frailty bias is to observe the vaccine effect both within and outside of the influenza season. If frailty bias exist, we would see higher rates of health complications in unvaccinated individuals outside of the influenza season. This would indicate that any protective effect observed within the season may be partially or fully due to the vaccinated individuals being healthier overall and an overestimation of the effect of the vaccine. Finally, in addition to measuring clinical outcomes to understand the effects of the vaccine, the antibody response elicited by the vaccine can be measured and used as a proxy for vaccine effectiveness.

There is uncertainty amongst primary and secondary care physicians whether influenza vaccines are able to trigger an appropriate antibody response and protect patients with liver disease from health complications[15], and the vaccination coverage is poor. Just over 55% of the chronic liver disease respondents to the 2016 US National Health Interview Survey[16] had been vaccinated against influenza in the past 12 months and less than 50% of working-age patients with liver disease registered with primary care practitioners in England had received influenza vaccination during the 2015/2016 season[17]. To inform vaccination policy and to help guide decision-making regarding vaccination, our review aims to provide a systematic synthesis of the available evidence

For peer review only - http://bmjopen.bmj.com/site/about/guidelines.xhtml

from both observational studies and randomised trials on the effectiveness of influenza vaccines to prevent serious health outcomes, hospitalisation and death, in adults with chronic liver disease. To address uncertainty over the vaccines' ability to trigger antibody response in liver patients, we also assess the effects of influenza vaccines on serological response in adults with chronic liver disease.

For peer review only

MATERIAL AND METHODS

Protocol and registration

This systematic review and meta-analysis followed the Preferred Reporting Items for Systematic Review and Meta-Analyses (PRISMA)[18]. The review protocol was specified in advance as part of a wider review plan that also includes a study of the effectiveness of pneumococcal vaccine. The protocol was registered in PROSPERO 13th June 2017 (registration number CRD42017067277) and published after peer-review on 16th March 2018[19]. Based on the peer-review feedback, we made minor amendments to the initial protocol (Supplementary materials, Table 1).

Eligibility criteria

Studies published in peer-reviewed journals were eligible for inclusion for this systematic review and meta-analysis if they met the following PICOS (participants, intervention, comparator, outcome and study design) criteria: study type was randomised or non-randomised controlled trial, cohort or case-control study; intervention was an injectable, inactivated whole-virus, split-virus or subunit influenza

For peer review only - http://bmjopen.bmj.com/site/about/guidelines.xhtml

vaccine; intervention was compared to placebo, alternative intervention or no vaccination (no comparison group was required for serological response); study population included at least ten ≥18 year-old adults with chronic liver disease of any severity or aetiology (by any classification) per comparison group; outcomes included all-cause hospitalisation or all-cause mortality (or supportive outcomes hospitalisation or mortality due to acute respiratory illness, influenza/ILI or liver disease complications) or serological response to influenza vaccine. We only included studies published in English. Review articles, case reports, cross-sectional studies, animal studies, editorials, clinical guidelines, studies with liver transplant patients only (response to the vaccination and clinical outcomes are likely to be strongly influenced by the immunosuppressive medication rather than the status of liver disease), studies focused on special populations (such as pregnant women, nursing home residents, or patients with other chronic diseases), test-negative case-control studies (where both cases and controls were hospitalised for influenza-related illness/ILI/acute respiratory illness, listed in Supplementary materials, Table 2) and studies retracted from publication were excluded.

Information sources and search strategy

We searched the Cochrane Central Register of Controlled Trials, Ovid MEDLINE, Ovid Embase and PubMed (originally up to 16 July 2017 and later updated up to 20 January 2019) for articles containing variations of terms "influenza", "flu", "seasonal", "TIV", "QIV", "3-valent", "4-valent", "trivalent" or "quadrivalent" in combination with terms "vaccine" or "immunisation" and "liver disease", "hepatic disease", "chronic liver", "chronic hepatic", or "cirrhosis". The search also included medical subject headings "Influenza Vaccines" (exploded), "Influenza, human /Prevention and Control", and

For peer review only

"Liver Diseases" (exploded). The search was filtered by study design, age group of participants, and publication type. To capture studies where liver disease patients may have been included as part of a general population, the search was repeated leaving out the liver disease-specific terms and with the study design filter restricted to trials only. The full search strategy in MEDLINE and the medical subject headings for all databases searched are provided in Supplementary materials, Table 3 and Table 4. Electronic searches were complemented by manually searching recent influenza vaccine guidelines from Centers for Disease Control and Prevention, European Centre for Disease Control and Prevention, and WHO, and the reference lists of the included studies.

Study selection

Titles of the articles identified through the search of the studies of influenza vaccination in the general population were first pre-screened by one reviewer (SH). Abstracts and titles of articles meeting the pre-screening criteria and of all articles identified through the liver disease-specific search were then screened by two independent reviewers (CP and SH). Studies deemed eligible were included in the full-text review by two independent reviewers (CP and SH). Disagreements were resolved by discussion and the reasons for exclusion were recorded. In case of uncertainty over eligibility, study authors were contacted. Studies for which this uncertainty could not be resolved were excluded (Supplementary materials, Table 5).

Data collection process and data items

Using standardised online forms, two review authors (CP and SH) independently and in duplicate extracted data on: study participants (inclusion and exclusion criteria, method

For peer review only - http://bmjopen.bmj.com/site/about/guidelines.xhtml

of recruitment/selection, study population characteristics), interventions and comparators (vaccine type, comparison treatment, dose, route of delivery, number and timing of vaccinations/comparator treatments, number of individuals and follow-up time in intervention and comparison groups), outcomes (definition, time points measured and reported, unit of measurement, number of outcomes in the intervention and control group, unadjusted and adjusted effect measures, covariates that the effect measures were adjusted for, comparisons, missing data and reasons for missingness, statistical methods used and processes for randomisation), study design (study type, country and setting, date of study, study duration, aim of study and withdrawals), study quality, study bias, study funding, and conflicts of interest. We contacted study authors to obtain subgroup data, missing data, and to clarify unclear data. Effect measures were collected in the format in which they were reported and transformed for analysis as required.

Risk of bias in individual studies

Two review authors (CP and SH) independently assessed the risk of bias. We used the Cochrane Collaborations tool[20] to assess randomised trials and the Newcastle-Ottawa scale (NOS)[21] to assess observational studies. Age, sex, severity and aetiology of liver disease, and presence of co-morbidities were considered the most important confounders in the assessment of observational studies. Additionally, in studies of pandemic vaccine effect on clinical outcomes, we considered as a potential confounder a previous seasonal influenza vaccination in the same season the study vaccine was administered. Risk of bias assessment in serological studies focused on the study population with liver disease. Disagreements over risk of bias were resolved by discussion between reviewers.

For peer review only

Summary measures and synthesis of results

Where more than a single study per outcome was identified and the study designs, protocols and measures of treatment effect were considered similar enough to produce a meaningful pooled effect, we used random-effects meta-analysis to summarise the average effects of vaccination. For serological vaccine response, we compared the liver patients' haemagglutinisation inhibition (HI) antibody responses before and after vaccination. Comparisons between titres and titre ratios were presented as a mean difference (MD) of log geometric mean titre (GMT) or log geometric mean titre ratio (GMTR) with 95% confidence intervals (CI, titres and ratios were log transformed to back to their original scale to obtain standard deviations). Seroconversion (defined as the proportion of patients whose negative pre-vaccination serum converted to a HI titre >1:40, or who experienced at least a four-fold titre increase, after vaccination) and seroprotection (defined as the proportion of patients achieving a HI titre of > 1:40 after vaccination) levels were presented as prevalence rates (%) with 95% CI and comparisons between rates were presented as risk ratio (RR) with 95% CI. As reference levels, for a theoretically adequate response, we used 40% for seroconversion and 70% for seroprotection. Reported rates expressed only as percentages that did not correspond to an integer number of patients were imputed to the closest possible number of patients (closest integer above the reported non-integer for baseline and closest integer below for effect rates). Response measures were presented in categories by virus subtype. The vaccine effects on hospitalisation and mortality were compared between vaccinated and unvaccinated liver disease patients and presented as a crude RR with 95% CI (adjusted effect measures, reported separately within results, were available from two studies but on different scales). Effect measures from multiple

For peer review only - http://bmjopen.bmj.com/site/about/guidelines.xhtml

corresponding seasons were combined into an overall measure within each study. Presence of frailty bias were evaluated by contrasting the effect estimates in and out of influenza season. Person-time was presented as person-seasons for seasonal and as person-years for whole-year effect estimates. We regarded heterogeneity between studies as substantial if τ^2 was greater than 0, I^2 was more than 30% and the p-value for Q-statistic was less than 0.10. Uncertainty in the estimates of between-study variance, likely to be greater when only a small number of effect estimates are pooled together, was not quantified (option not available in the software used). Statistical analyses were performed using Stata 14.0 and RevMan 5.3.

Additional analyses

Subgroup analyses and meta-regression were not carried out due to the low number of studies. Sensitivity analyses were carried out to investigate whether the serological vaccine effects were similar in less severe (non-cirrhotic disease) and advanced liver disease (cirrhotic disease) comparing response in all patients to that in cirrhotic patients only. We were not able to conduct sensitivity analyses based on aetiology of disease as the study populations were mainly of mixed aetiologies. In studies that included a healthy control population, we compared antibody responses between liver disease patients and the controls.

Risk of bias across the studies

For each of the review outcomes, we evaluated the risk of selective outcome reporting bias using the Outcome Reporting Bias in Trials (ORBIT) classification system[22]. Presence of publication bias was assessed based on this evaluation and our review design (funnel plots or formal tests were not used because of the low number of

For peer review only - http://bmjopen.bmj.com/site/about/guidelines.xhtml

studies). The Grading of Recommendations Assessment, Development and Evaluation (GRADE) Working Group system[23] was used to assess and report the overall quality of evidence. In this assessment, we regarded a vaccine effect outside of the influenza season (suggesting the effect of frailty bias) for clinical outcomes as confounding (contributing to study limitations). All assessments were completed by two independent reviewers (CP and SH) and disagreements were resolved by discussion between reviewers.

Role of the funding source

The funder of this study had no role in study design, data collection, data analysis, data interpretation, or writing of the report. The corresponding author had full access to all the data in the study and had final responsibility for the decision to submit for publication.

Patient and public involvement

Patients and the public were not involved in the design or planning of this study.

RESULTS

The search of influenza studies in the general population provided a total of 10649 unique study records. After title screening, 1607 of these records and 608 unique records from the liver disease-specific search were included in the abstract and title screening. The full text of 286 studies was examined in detail, and of these, ten studies met the inclusion criteria for the review. An additional two studies were identified

For peer review only - http://bmjopen.bmj.com/site/about/guidelines.xhtml

through recent influenza vaccine guidelines, making the total number of included studies 12. The study flowchart is presented in Figure 1.

The included studies consisted of one randomized controlled trial and 11 cohort studies. Six studies included serological outcomes (Table 1) and six included clinical outcomes (Table 2). The studies with serological outcomes had low (4 studies) to moderate bias (2 studies) in selection of study population and low (2 studies), moderate (3 studies) and high bias (1 study) in assessment of the outcomes (bias mostly due to lack of description of independent, blind outcome assessment). Individual studies with clinical outcomes had moderate to low risk of bias in selection of the study population (in two studies vaccination status was self-reported) and low risk of bias in outcome assessment (majority used medical records). In most studies, the presence of confounding due to demographic and clinical characteristics was unclear (we did not have characteristics for the liver patient subgroups obtained from the studies conducted in the general population) and the results for control periods (non-influenza periods) were available only from two studies. High risk of selective outcome reporting was suspected in only one clinical study (outcome all-cause mortality) but mainly in serological studies (outcome GMTR). Evidence for all outcomes was judged as very low quality. Risk of bias and selective outcome reporting assessments, and the GRADE evidence profile are provided in Supplementary materials, Table 6, Table 7 and Table 8.

For peer review only - http://bmjopen.bmj.com/site/about/guidelines.xhtml

Table 1. Characteristics of the included studies on serological response to influenza vaccine

Study	Location	Design	Liver disease participants (% cirrhosis, % viral aetiology)	Sample size	Vaccine	Follow-up	Outcomes
Cheong et al (2006)[24]	South Korea	Cohort study with comparison group	Advanced cirrhosis patients (100% cirrhosis 72% viral aetiology)	50 (54 healthy controls)	Trivalent, split-virus	4 weeks	GMT<br><br>Seroconversion rate<br><br>Seroprotection rate
Duchini et al (2001)[25]	United States	Cohort study with comparison groups	Cirrhosis patients (100% cirrhosis, 56% viral aetiology	14 (9 healthy controls)	Trivalent, split-virus	6 weeks	GMT<br><br>GMTR<br><br>Seroconversion rate
Hernández-Guerra et al (2012)[26]	Canary Islands, Spain	Cohort study with comparison groups	Chronic HCV patients (0% cirrhosis, 100% viral aetiology)	25 (15 healthy controls)	Monovalent, split virus	4-11 weeks	GMT<br><br>GMTR<br><br>Seroconversion rate<br><br>Seroprotection rate
Ohfuji et al (2013)[27]	Japan	Cohort study	Chronic HCV patients (25% cirrhosis, 100% viral aetiology)	79	Monovalent, split virus	3 weeks	GMT<br><br>GMTR<br><br>Seroconversion rate<br><br>Seroprotection rate
Sayyad et al (2012)[28]	Iran	Cohort study with comparison group	Cirrhosis patients and inactive HBV carriers (47% cirrhosis, 100% viral aetiology in non-cirrhotics, aetiologies not provided in cirrhotics)	28, 31 (34 healthy controls)	Trivalent, subunit	4 weeks	GMT<br><br>Seroconversion rate<br><br>Seroprotection rate
Soesman et al (2000)[29]	The Netherlands	Cohort study with comparison groups	Cirrhosis patients (100% cirrhosis, aetiologies not provided)	36 (45 healthy controls)	Trivalent, subunit	4 weeks	GMT<br><br>Seroconversion rate<br><br>Seroprotection rate

For peer review only - http://bmjopen.bmj.com/site/about/guidelines.xhtml

Table 2. Characteristics of the included studies on clinical outcomes after influenza vaccination

Study	Location	Design	Liver disease participants	Sample size (liver disease patients)	Study period	Outcomes
Campitelli et al (2010)[30]	Canada	Cohort study	Chronic liver disease patients among the community-dwelling ≥65 year-olds	205 vaccinated/124 unvaccinated	8 influenza seasons 1996-1998 and 2001-2007	All-cause mortality<br><br>Acute respiratory illness-related hospitalisation (pneumonia & influenza)
Castilla et al (2015)[31]	Spain	Cohort study	Cirrhosis patients among the community-dwelling ≥65 year-olds	3126 person-seasons vaccinated/1804 person-seasons unvaccinated<br><br>Number of patients not reported.	2 influenza seasons 2011-2013	All-cause mortality
Ohfuji et al (2014)[32]	Japan	Cohort study	Chronic HCV hospital outpatients (64% ≥65 year-olds)	132 vaccinated/276 unvaccinated	1 influenza season	All-cause hospitalisation
Song et al (2007)[33]	South Korea	Randomised, controlled trial	Cirrhotic hospital in- and outpatients (50% ≥55 year-olds)	175 vaccinated/90 unvaccinated	1 influenza season 2004-2005	Influenza illness-related mortality (laboratory-confirmed influenza)
Su et al (2016)[34]	Taiwan	Cohort study	Chronic HBV hospital outpatients (38% ≥60 year-olds)	4434 vaccinated/3646 unvaccinated	9 influenza seasons 2000-2009	All-cause hospitalisation<br><br>All-cause mortality<br><br>Liver disease complication related hospitalisation (septicaemia, bacteraemia, viraemia)
Vila-Córcoles et al (2007)[35]	Spain	Cohort study	Cirrhosis patients among the community-dwelling ≥65 year-olds	100-117 vaccinated/73-104 unvaccinated (numbers varied per year)	4 influenza seasons, 3 non-influenza periods, 3 full years 2002-2005	All-cause mortality

For peer review only - http://bmjopen.bmj.com/site/about/guidelines.xhtml

For peer review only

The serological response to influenza vaccine was assessed in six studies including a total of 262 liver disease patients (Table 1). Each study employed a cohort design in which all study participants were vaccinated. Mean age of liver disease participants ranged between 42 and 65 years (one study did not report baseline characteristics of participants). Three studies enrolled exclusively cirrhotic patients in their liver disease group, two studies enrolled both patients with and without cirrhosis and one study had separate groups for cirrhotic and non-cirrhotic patients. Hepatitis C was the main cause of liver disease in three studies and hepatitis B in two studies. In one study, aetiology was not specified. Four studies immunised the participants with a trivalent seasonal vaccine and two used a monovalent pandemic vaccine. In all studies, blood samples for post-vaccination measurements were drawn after minimum of three weeks and antibody titres measured using a traditional HI assay (antibody titre data is provided in Supplementary materials, Table 9).

HI antibody levels of liver disease patients increased in response to vaccination (pooled random-effects MD of log GMTs 1·92, 95% CI [1·65, 2·19]) (Figure 2). There was no substantial heterogeneity between individual estimates (τ^2=0·04, I^2=15%, p=0·28) or levels against influenza A and B viruses (p=0·16). Seroconversion rate in liver disease patients to both A/H1N1 and B subtypes was clearly above the 40% reference level (pooled random-effects prevalence for A/H1N1 79·95%, 95% CI [68·19%, 89·70%] and pooled random-effects prevalence for B 86·82%, 95% CI [71·87%, 97·07%]) and around the 40% in the case of subtype A/H3N2 (pooled random-effects prevalence 55·53%, 95% CI [39·92%, 70·63%]). While the majority of the studies reported

For peer review only - http://bmjopen.bmj.com/site/about/guidelines.xhtml
For peer review only

estimates above 40%, there was substantial heterogeneity between response rates (I^2=76·55% and p<0.01 for subtype A/H1N1; I^2=72·37% and p=0·01 for subtype A/H3N2; I^2=79·99% and p<0·01 for subtype B). Seroconversion rate is presented in Figure 3. Seroprotection rate in liver disease patients was above the 70% reference level (pooled random-effects rate 93·88%, 95% CI [87·33%, 98·40%]) (Figure 4). While the majority of studies reported a seroprotection rate above 70%, there was substantial heterogeneity (I^2=83·81%, p<0.01) (Figure 4). Although some studies reported a substantial seroprotection level before vaccination, the pooled pre-vaccination seroprotection rate was clearly below 70% (Supplementary materials, Figure 1).

The effectiveness of influenza vaccine in preventing all-cause hospitalisation in liver disease patients was investigated in two cohort studies, published in 2014 and 2016[32,34]. A study of 408 participants from Japan[32] investigated pandemic vaccine (against a single virus subtype) and enrolled exclusively chronic hepatitis C virus (HCV) patients. A study of 8080 participants from Taiwan[34] investigated seasonal vaccine (against multiple virus subtypes) exclusively in chronic hepatitis B virus (HBV) patients. In the study of HCV patients, patients were prospectively recruited from those who were under clinical follow-up for their liver disease but vaccination status was self-reported[32]. In the study of HBV patients, both liver disease and vaccination status were determined from electronic medical claim records[34]. One study presented results for one influenza season and the other for an entire year after vaccination and pooled the results from a period of nine years (Figure 5). Both studies compared the rate of all-cause hospital admission (first incidence) between vaccinated and unvaccinated individuals.

For peer review only - http://bmjopen.bmj.com/site/about/guidelines.xhtml

Vaccinated HCV patients, were no less likely to be hospitalised than unvaccinated patients during an influenza season after pandemic influenza A vaccination (RR 0·57, 95% CI [0·24, 1·37], RD -0.03, 95% CI [-0.08, 0.01])[32]. In this study, hospitalisation outside the influenza season was not measured. Adjusting the effect estimate (for chronic co-morbidities, steroid treatment within the preceding 6 months and albumin level), calculated in the study as the odds ratio (crude odds ratio 0.55, 95% CI [0.22,1.39]), adjusted odds ratio 0.43, 95% CI [0.16,1.17]), did not reveal a significant effect[32]. Seasonal vaccination, however, was associated with a drop from 205/1000 patients to 149/1000 patients being hospitalised during a full year of follow-up (RD -0·06, 95% CI [-0·07, -0·04]). Vaccinated HBV patients were 27% less likely to be admitted to the hospital than unvaccinated patients (RR 0·73, 95% CI [0·66, 0·80])[34]. Both the crude and the adjusted time-to-event estimate that accounted for cirrhosis status, age, sex, pneumococcal vaccine, comorbidities and recent hospitalisation, calculated in the study as the hazard ratio (HR), showed a protective effect (crude HR 0·68, 95% CI [0·62, 0·76], adjusted HR 0.56, 95% CI [0·50, 0·62])[34]. Based on a Kaplan-Meier plot, the difference in event-free survival probability between vaccinated and unvaccinated was established during the influenza season, approximately within the first three months after vaccination[34]. The effect of influenza vaccine on cause-specific hospitalisation was investigated in two studies[30,34]. A cohort study from Canada, published in 2010, measured acute respiratory illness-related hospitalisation during influenza season (defined as influenza/pneumonia hospitalisation) using medical claims and hospital records[30]. A total of 329 liver disease patients were included as part of a general population, however, as there were five or fewer hospitalisation events in both groups, the study authors were unable to release the results (reason: institution's data policy, personal communication). A liver disease

For peer review only - http://bmjopen.bmj.com/site/about/guidelines.xhtml

complication-related hospitalisation outcome was included in the Taiwanese study of HBV patients[34]. Vaccinated liver disease patients were 57% less likely to be admitted to hospital for unspecified septicaemia, bacteraemia and viraemia than unvaccinated patients (RR 0·43, 95% CI [0·31, 0·59]). Vaccination was associated with a fall from 26/1000 patients to 11/1000 patients being hospitalised (RD -0·02, 95% [CI -0·02, -0·01]). It appeared, however, that a difference in event-free survival probability was not established during the influenza season (Kaplan-Meier plot)[34]. No studies with the outcome influenza illness/ILI-related hospitalisation were identified.

The effectiveness of influenza vaccine in preventing all-cause mortality in liver disease patients was investigated in five cohort studies[30,31,34,35], published between 2007 and 2016. Only the Taiwanese study of HBV patients, enrolled exclusively liver disease patients[34]. Two studies from Spain[31,35] and one from Canada[30] enrolled liver disease patients as part of the general, community-dwelling population. All studies ascertained liver disease diagnosis and vaccination status from electronic health or medical claim records. In one study, the record-based vaccination status was based on self-reporting. The Spanish studies followed patients over multiple seasons from the start of the study allowing them to contribute to both the unvaccinated and vaccinated follow-up time for different seasons [31,35] whereas the other two studies followed patients up for a single season/year[30,34], although this was over multiple seasons/years. All studies compared all-cause mortality rates between vaccinated and unvaccinated individuals.

Results are presented in Figure 6. Vaccinated patients were no less likely to die during influenza season (pooled random effects RR 0·80, 95% CI [0·43, 1·50], RD -0.001, 95%

For peer review only - http://bmjopen.bmj.com/site/about/guidelines.xhtml

CI [-0.01, 0.01]), or the entire year (pooled random effects RR 0·41, 95% CI [0·11, 1·52], RD -0.06, 95% CI [-0.08, 0.04]). All the studies that contributed to the influenza season-specific estimate reported non-significant effects and there was no substantial heterogeneity (τ^2=0·12, I^2=33·00%, p=0·23). The studies that contributed to the whole-year estimate were substantially heterogeneous (τ^2=0·83, I^2=94.00%, p<0.00), with the smaller Spanish study[35] reporting a non-significant effect (personal communication) and the larger Taiwanese study reporting a large protective effect (RR 0·22, 95% CI [0·17, 0·28])[34]. The Spanish study reported no effect outside the influenza period (RR 0·52, 95% CI [0·15, 1·83]). The Canadian study also measured the effect outside the non-influenza period but as there were five or fewer deaths in both groups, the authors were unable to provide the results (institution's data policy, personal communication). Based on a Kaplan-Meier plot presented in the Taiwanese study, the difference in survival probability was mainly established approximately in the first five months after vaccination[34], however, it was not clear whether the increase in death rate fully levelled off after the influenza season and it may be that this to some extent contributed to the size of the yearly effect estimate in this study. The effect of influenza vaccine on the rate of cause-specific mortality was investigated in one study[33]. The effect of influenza vaccine in preventing influenza illness/ILI-related mortality was included as an outcome in a South Korean randomised, controlled trial of cirrhotic patients, published in 2007[33]. No deaths due to laboratory-confirmed influenza occurred among the 175 vaccinated or the 90 unvaccinated patients during the influenza season[33]. No studies investigating mortality related to acute respiratory illness or liver disease complications were identified.

For peer review only - http://bmjopen.bmj.com/site/about/guidelines.xhtml

To understand whether the vaccine effects were different based on the patients' cirrhosis status, we conducted sensitivity analyses. The serological response in cirrhotic patients did not differ from the results of our main analyses (Supplementary materials, Figures 2-5). We did not have the data and therefore were not able to conduct similar analyses for the clinical outcomes.

In addition to comparing the vaccine effects within liver patients, the studies of serological response (5/6 studies) also compared the outcomes between liver disease patients and healthy controls (individuals without liver disease). The antibody titres before or after vaccination, seroconversion rate and seroprotection rate of liver patients did not differ from those of healthy individuals. Pre- to post-vaccination titre ratios (GMTRs) reported were similar between liver patients and healthy individuals except for influenza subtype A/H3N2 where the mean fold-increase in antibody levels of liver patients was lower than that of healthy individuals. The pooled results for these comparisons are provided in Supplementary materials, Figures 6-11. The studies on clinical outcomes in liver patients did not include healthy comparison groups and while we did not seek these data from the studies that included liver patients as part of a general population, the all-cause mortality results in the entire general population can be found in Supplementary materials, Table 10.

DISCUSSION

Six cohort studies from three continents (Asia, Europe and North America) investigated the serological vaccine response in liver disease patients. Two cohort studies and one

For peer review only - http://bmjopen.bmj.com/site/about/guidelines.xhtml

randomised controlled trial, all from Asia, investigated the vaccine effect on hospitalisation and/or mortality in liver disease patients exclusively. In addition, there are at least three cohort studies, two from Europe and one from North America, that have assessed the vaccine effect on these outcomes in a general community-dwelling elderly population including a subgroup with liver disease. Overall, the available evidence suggests that while influenza vaccine may not protect against all-cause mortality, it triggers an effective antibody response and may reduce the risk of all-cause hospitalisation in liver disease patients (findings for clinical outcomes summarised in Table 3, maximum quality obtainable is "low" as all included studies are non-randomised).

Table 3. Summary of findings for clinical outcomes
Effectiveness of influenza vaccination in preventing all-cause hospitalisation and all-cause mortality in adults with chronic liver disease

	**Patients: Adults with chronic liver disease**<br>**Setting: General population**<br>**Intervention: Influenza vaccination (injectable, inactivated)**<br>**Comparison: No vaccination**					
**Outcome**	**Risk/rate in unvaccinated** [a]	**Risk/rate in vaccinated**	**Relative effect**	**Vaccine effectiveness**	**Number of participants/person-time (number and type of studies)**	**Quality of evidence (GRADE)**
All-cause hospitalisation, influenza season (pandemic vaccine)	80 per 1000	46 per 1000 (19-110)	0.57 (0.24, 1.37)	Not significant	408 (1 observational study)	Very low[b]
All-cause hospitalisation, all year	205 per 1000	150 per 1000 (135-164)	0.73 (0.66, 0.80)	27% (20%, 34%)[c]	8080 (1 observational study)	Very low[d]
All-cause mortality, influenza season	17 per 1000 person-seasons	14 per 1000 person-seasons (7-26)	0.80 (0.43, 1.50)	Not significant	5975 person-seasons (3 observational studies)	Very low[e]

For peer review only - http://bmjopen.bmj.com/site/about/guidelines.xhtml

All-cause mortality, all year	85 per 1000 person-seasons	35 per 1000 person-seasons (9-129)	0.41 (0.11, 1.52)	Not significant	8405 person-years (2 observational studies)	Very low[f]

[a] Median risk in unvaccinated across the studies.
[b] Rated down from low to very low based on study limitations, imprecision of the estimate and suspicion of publication bias.
[c] Could be applicable to HBV patients only as the study only enrolled HBV patients.
[d] Rated down from low to very low based on suspicion of publication bias.
[e] Rated down from low to very low based on study limitations, inconsistency in absolute rates, imprecision of the estimate and suspicion of publication bias.
[f] Rated down from low to very low based on study limitations, imprecision of the estimate and suspicion of publication bias.

For peer review only

A previous systematic review[14] on the effectiveness of inactivated influenza vaccine in different at-risk target groups identified only one randomised study in liver patients. This study of 311 cirrhosis patients, also included in our current review, found seasonal influenza vaccine effective against laboratory-confirmed influenza but not against ILI[33]. The study included hospitalised patients and could not investigate vaccine effectiveness against hospitalisation. Vaccine effect on influenza-related mortality was investigated, however, no deaths occurred during the study. It is possible that the effects we found are not entirely due to the vaccine. Other factors that may have affected the clinical outcomes include frailty bias, competing risks (in case of our secondary, cause-specific outcomes) and differences in previous vaccinations, social support, healthcare system, lifestyle, and in the severity or type of liver disease between the vaccinated and unvaccinated populations. Serological outcomes may have been further affected by variation of the antibody assays.

The data presented are predominantly from patients with viral liver disease. We cannot therefore be certain that our findings are applicable to patients with liver disease caused by alcohol, obesity or other non-viral factors. While sensitivity analyses showed

For peer review only - http://bmjopen.bmj.com/site/about/guidelines.xhtml

that compared to non-cirrhotic liver disease, having cirrhosis did not worsen the antibody response to the vaccine, most data in the analyses of clinical outcomes come from patients with a milder form of liver disease than cirrhosis. We might expect that in severe liver disease complications from influenza are more frequent and we may have seen more outcome events if more cirrhotic patients were included. Given the proxy effectiveness in cirrhotic patients indicated by the antibody responses, this would mean that our study may have somewhat underestimated the true protective effect against clinical outcomes. However, all our data on the hospitalisation outcome are from high-income Asian countries and it is possible that the protective vaccine effect may not extend to populations from other geographical and income settings with different prevalence of co-morbidities, age distribution, influenza seasonality and vaccine formulation used.

Our review has several strengths. It was conducted in a transparent way following established guidelines and a prospectively specified protocol. The search was conducted over multiple databases and we searched for both studies in liver disease patients and studies in which liver disease patients were included as part of the general population. To reduce the risk of missing relevant studies and relevant data and to increase the objectivity of the review, two independent reviewers screened the studies, extracted data and assessed the risk of bias. We studied all-cause outcomes to take into account the complex interplay of a chronic condition and acute infections. An underlying diagnosis of chronic liver disease may influence how cause of death and hospitalisation is recorded. This potentially reduces the number of cases that are attributed to influenza and solely investigating diagnoses of influenza is likely to substantially underestimate the impact of the vaccine.

For peer review only

There are also limitations to our review. The meta-analyses combine data from studies that differ in terms of patient population, outcome definitions and vaccine used. For the studies that included liver disease patients as part of the general population, we had very limited information about the baseline characteristics of the liver patient subgroup. In addition, despite the comprehensive search, publication bias, restriction on publication language and varying response to queries about data on liver disease subgroups may have affected the effects observed. We were not able to employ translation services and may have omitted relevant studies published in languages other than English from countries such as China where influenza vaccines are manufactured and liver disease is a significant public health challenge. A number of studies potentially included liver disease patients as part of a wider study population but were unable to obtain confirmation and liver disease subgroup data from the study authors. Finally, the body of evidence identified in this review is limited both in quantity and quality, leaving room for uncertainty around the effects of the vaccination. The low quality is partly due to the non-randomised design of the included studies. This limitation may persist also in future reviews as conducting randomised controlled trials in a high-risk population are ethically challenging.

Evidence from mainly patients with viral liver disease suggests that underlying liver disease does not prevent patients from mounting an antibody response to influenza vaccine and similar proxy effectiveness is seen both in non-cirrhotic and cirrhotic patients. We also identified a protective influenza vaccine effect against yearly all-cause hospitalisation in patients with viral liver disease. Assuming this effect – estimated in a large cohort study of over 8000 patients – is due to the vaccine, seasonal influenza

For peer review only - http://bmjopen.bmj.com/site/about/guidelines.xhtml

vaccination of 14–24 viral liver disease patients could prevent one hospitalisation per year. It is uncertain whether the estimated effects extend to patients with liver disease of other than viral origin. No protective effect against all-cause mortality was found.

Current uptake of seasonal influenza vaccination is low. A recent systematic review identified low perceived vaccine effectiveness, and low concern and perceived risk of severe influenza infection as the most common barriers for seasonal influenza vaccination in patients with chronic illness[36]. Ideally, the evidence for influenza vaccine effectiveness in liver disease would come from a large randomised vaccine trial in liver disease patients. In the absence of this evidence, we must rely on proxy estimates for effectiveness from serological studies and evidence on clinical outcomes from observational studies. The best available evidence on the effectiveness of influenza vaccine in liver disease, summarised in this review, shows both proxy effectiveness and protection against all-cause hospitalization. The limited quantity and quality of this evidence means that the protective vaccine effect may be uncertain. However, considering the high risk of serious health outcomes from influenza infection in liver patients and the safety and low cost of vaccination, overall the potential benefits of seasonal vaccination both to patients and the healthcare systems are likely to outweigh the costs and risks associated with vaccination. Wider collection of vaccination data in adults and liver patient -focused studies using large databases linking routinely collected data on vaccinations and health may allow us to address the remaining uncertainty about the effects of influenza vaccination in liver disease.

Contributors

For peer review only

The study was conceived by SH, LS, AO and AH. SH developed the eligibility criteria, search strategy, risk of bias assessment strategy and data extraction plan with guidance from LS, AO and AH. SH (trained in systematic methods of literature searching as part of her PhD studies) performed the literature searches and pre-screening of titles. SH and CP reviewed titles and abstracts, and full text articles, extracted data, and assessed study bias and quality. SH analysed the data, and wrote the manuscript to which all authors CP, LS, AO and AH contributed.

Funding

This work was supported by the UK Biotechnology and Biological Sciences Research Council grant [grant number BBSRC BB/M009513/1] to SH.

Data sharing statement

All data relevant to the study are included in the article or uploaded as supplementary information.

Competing interests

The authors declare that no competing interests exist.

Acknowledgements

We wish to thank the authors of the reviewed studies for kindly responding to our enquiries and for providing liver patient subgroup data where possible.

For peer review only

Figure legends

Figure 1. Study selection

Figure 2. Serological response to influenza vaccination: mean difference in log HI antibody GMTs before and after vaccination. *Note: Sayyad et al (2012a) includes cirrhotic patients and Sayyad et al (2012b) includes non-cirrhotic patients only.*

Figure 3. Serological response to influenza vaccination: seroconversion rate after vaccination. *Note: Sayyad et al (2012a) includes cirrhotic patients and Sayyad et al (2012b) includes non-cirrhotic patients only.*

Figure 4. Serological response to influenza vaccination: seroprotection rate after vaccination. *Note: Sayyad et al (2012a) includes cirrhotic patients and Sayyad et al (2012b) includes non-cirrhotic patients only.*

Figure 5. Effect of influenza vaccine on clinical outcomes: all-cause hospitalisation in vaccinated compared to unvaccinated liver disease patients. Note: Ohfuji et al (2014) investigated the effect of pandemic (monovalent) vaccine and Su et al (2016) of seasonal vaccine (against three or more virus subtypes).

Figure 6. Effect of influenza vaccine on clinical outcomes: all-cause mortality in vaccinated compared to unvaccinated liver disease patients.

For peer review only

For peer review only

For peer review only - http://bmjopen.bmj.com/site/about/guidelines.xhtml

REFERENCES

1 GBD 2016 Disease and Injury Incidence and Prevalence Collaborators. Global, regional, and national incidence, prevalence, and years lived with disability for 328 diseases and injuries for 195 countries, 1990–2016: a systematic analysis for the Global Burden of Disease Study 2016. *Lancet* 2017;**390**:1211–59. doi:10.1016/S0140-6736(17)32154-2

2 Asrani SK, Devarbhavi H, Eaton J, *et al.* Burden of liver diseases in the world. *J Hepatol* 2019;**70**:151–71. doi:10.1016/j.jhep.2018.09.014

3 Fierer J, Finley F. Deficient Serum Bactericidal Activity Against Escherichia Coli in Patients with Cirrhosis of the Liver. *J Clin Invest* 1979;**63**:912–21. doi:10.1172/JCI109391

4 Hassner A, Kletter Y, Shlag D, *et al.* Impaired monocyte function in liver cirrhosis. *Br Med J (Clin Res Ed)* 1981;**282**:1262–3.

5 Brien AJO, Fullerton JN, Massey KA, *et al.* Prostaglandin E2 Mediates Immunosuppression in Acutely Decompensated Cirrhosis. *Nat Med* 2014;**20**:518–23. doi:10.1038/nm.3516

6 Rajkovic IA, Williams R. Abnormalities of Neutrophil Phagocytosis, Intracellular Killing and Metabolic Activity in Alcoholic Cirrhosis and Hepatitis. *Hepatology* 1986;**6**:252–62. doi:10.1002/hep.1840060217

7 Puig-Barberà J, Natividad-Sancho A, Trushakova S, *et al.* Epidemiology of Hospital Admissions with Influenza during the 2013 / 2014 Northern Hemisphere Influenza Season: Results from the Global Influenza Hospital Surveillance Network. *PLoS One* 2016;**11**:e0154970. doi:10.1371/journal.pone.0154970

8 Kerkhove MD Van, Vandemaele KAH, Shinde V, *et al.* Risk Factors for Severe

For peer review only

Outcomes following 2009 Influenza A (H1N1) Infection: A Global Pooled Analysis. *PLoS Med* 2011;**8**:e1001053. doi:10.1371/journal.pmed.1001053

9 Polakos NK, Cornejo JC, Murray DA, *et al.* Kupffer Cell-Dependent Hepatitis Occurs during Influenza Infection. *Am J Pathol* 2006;**168**:1169–78. doi:10.2353/ajpath.2006.050875

10 Duchini A, Viernes ME, Nyberg LM, *et al.* Hepatic Decompensation in Patients With Cirrhosis During Infection With Influenza A. *Arch Intern Med* 2000;**160**:113–5. doi:10.1001/archinte.160.1.113

11 Demicheli V, Jefferson T, Ferroni E, *et al.* Vaccines for preventing influenza in healthy adults. *Cochrane Database Syst Rev* Published Online First: 2018. doi:10.1002/14651858.CD001269.pub6

12 Demicheli V, Jefferson T, Di Pietrantonj C, *et al.* Vaccines for preventing influenza in the elderly. *Cochrane Database Syst Rev* Published Online First: 2018. doi:10.1002/14651858.CD004876.pub4

13 European Centre for Disease Prevention and Control. Seasonal influenza vaccination and antiviral use in Europe – Overview of vaccination recommendations and coverage rates in the EU Member States for the 2013–14 and 2014–15 influenza seasons. 2016.

14 Michiels B, Govaerts F, Remmen R, *et al.* A systematic review of the evidence on the effectiveness and risks of inactivated influenza vaccines in different target groups. *Vaccine* 2011;**29**:9159–70. doi:10.1016/j.vaccine.2011.08.008

15 Leise MD, Talwalkar JA. Immunizations in chronic liver disease: what should be done and what is the evidence. *Curr Gastroenterol Rep* 2013;**15**:300. doi:10.1007/s11894-012-0300-6

16 National Center for Health Statistics. National Health Interview Survey. Public-use

For peer review only

data file and documentation. 2016.

17 Public Health England. Influenza immunisation programme for England: GP patient groups - Data collection survey Season 2015 to 2016. 2016.

18 Moher D, Liberati A, Tetzlaff J, *et al.* Academia and Clinic Annals of Internal Medicine Preferred Reporting Items for Systematic Reviews and Meta-Analyses: The PRISMA Statement. *Annu Intern Med* 2009;**151**:264–9. doi:10.1371/journal.pmed1000097

19 Härmälä S, Parisinos C, Shallcross L, *et al.* Effectiveness of pneumococcal and influenza vaccines to prevent serious health complications in adults with chronic liver disease: A protocol for a systematic review. *BMJ Open* 2018;**8**. doi:10.1136/bmjopen-2017-018223

20 The Cochrane Collaboration. Cochrane Handbook for Systematic Reviews of Interventions. Version 5.1.0 [updated March 2011]. 2011.http://handbook-5-1.cochrane.org/ (accessed 25 Jul 2018).

21 Wells G, Shea B, O'Connell D, *et al.* The Newcastle-Ottawa Scale (NOS) for assessing the quality of nonrandomised studies in meta-analyses. http://www.ohri.ca/programs/clinical_epidemiology/oxford.asp (accessed 25 Jul 2018).

22 Kirkham JJ, Dwan KM, Altman DG, *et al.* The impact of outcome reporting bias in randomised controlled trials on a cohort of systematic reviews. *BMJ* 2010;**340**:c365. doi:10.1136/bmj.c365

23 Guyatt GH, Oxman AD, Vist GE, *et al.* GRADE: an emerging consensus on rating quality of evidence and strength of recommendations. *BMJ* 2008;**336**:924–6. doi:10.1136/bmj.39489.470347.AD

24 Cheong H-J, Song J-Y, Park J-W, *et al.* Humoral and cellular immune responses to

For peer review only

influenza vaccine in patients with advanced cirrhosis. *Vaccine* 2006;**24**:2417–22. doi:10.1016/j.vaccine.2005.11.064

25 Duchini A, Hendry RM, Nyberg LM, *et al.* Immune response to influenza vaccine in adult liver transplant recipients. *Liver Transplant* 2001;**7**:311–3. doi:10.1053/jlts.2001.23010

26 Hernandez-Guerra M, Gonzalez-Mendez Y, de Molina P, *et al.* Immunogenicity and Acceptance of Influenza A (H1N1) Vaccine in a Cohort of Chronic Hepatitis C Patients Receiving Pegylated-Interferon Treatment. *PLoS One* 2012;**7**:e48610. doi:10.1371/journal.pone.0048610

27 Ohfuji S, Fukushima W, Tamori A, *et al.* Immunogenicity of influenza A(H1N1)pdm09 vaccine and the associated factors on lowered immune response in patients with hepatitis C. *Influenza Other Respi Viruses* 2013;**7**:456–65. doi:10.1111/j.1750-2659.2012.00424.x

28 Sayyad B, Alavian SM, Najafi F, *et al.* Efficacy of Influenza Vaccination in Patients with Cirrhosis and Inactive Carriers of Hepatitis B Virus Infection. *Iran Red Crescent Med J* 2012;**14**:623–30.

29 Soesman NMR, Rimmelzwaan GF, Nieuwkoop NJ, *et al.* Efficacy of influenza vaccination in adult liver transplant recipients. *J Med Virol* 2000;**61**:85–93. doi:10.1002/(SICI)1096-9071(200005)61:1<85::AID-JMV14>3.0.CO;2-H

30 Campitelli MA, Rosella LC, Stukel TA, *et al.* Influenza vaccination and all-cause mortality in community-dwelling elderly in Ontario, Canada, a cohort study. *Vaccine* 2010;**29**:240–6. doi:10.1016/j.vaccine.2010.10.049

31 Castilla J, Guevara M, Martinez-Baz I, *et al.* Enhanced estimates of the influenza vaccination effect in preventing mortality a prospective cohort study. Med. (United States). 2015;**94**:e1240.

For peer review only

32 Ohfuji S, Fukushima W, Sasaki Y, *et al.* Influenza A(H1N1)pdm09 vaccine effectiveness and other characteristics associated with hospitalization in chronic liver disease patients. *Liver Int* 2014;**34**:700–6. doi:10.1111/liv.12295

33 Song JY, Cheong HJ, Ha SH, *et al.* Clinical impact of influenza immunization in patients with liver cirrhosis. *J Clin Virol* 2007;**39**:159–63. doi:10.1016/j.jcv.2007.04.018

34 Su F-H, Huang Y-L, Sung F-C, *et al.* Annual influenza vaccination reduces total hospitalization in patients with chronic hepatitis B virus infection: A population-based analysis. *Vaccine* 2016;**34**:120–7. doi:10.1016/j.vaccine.2015.10.129

35 Vila-Córcoles A, Rodriguez T, de Diego C, *et al.* Effect of influenza vaccine status on winter mortality in Spanish community-dwelling elderly people during 2002-2005 influenza periods. Vaccine. 2007;**25**:6699–707.

36 Schmid P, Rauber D, Betsch C, *et al.* Barriers of influenza vaccination intention and behavior - A systematic review of influenza vaccine hesitancy, 2005-2016. *PLoS One* 2017;**12**:e0170550. doi:10.1371/journal.pone.0170550

For peer review only

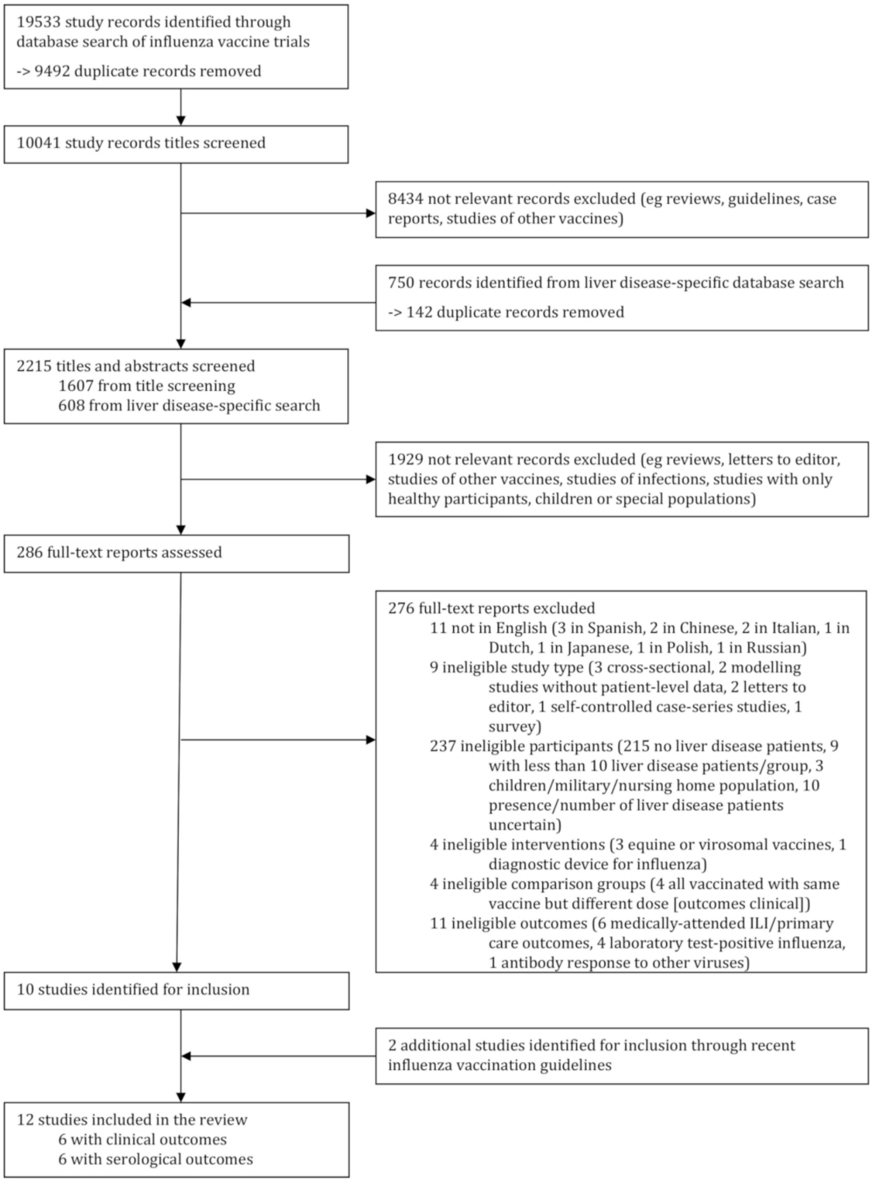

Figure 1. Study selection

150x202mm (300 x 300 DPI)

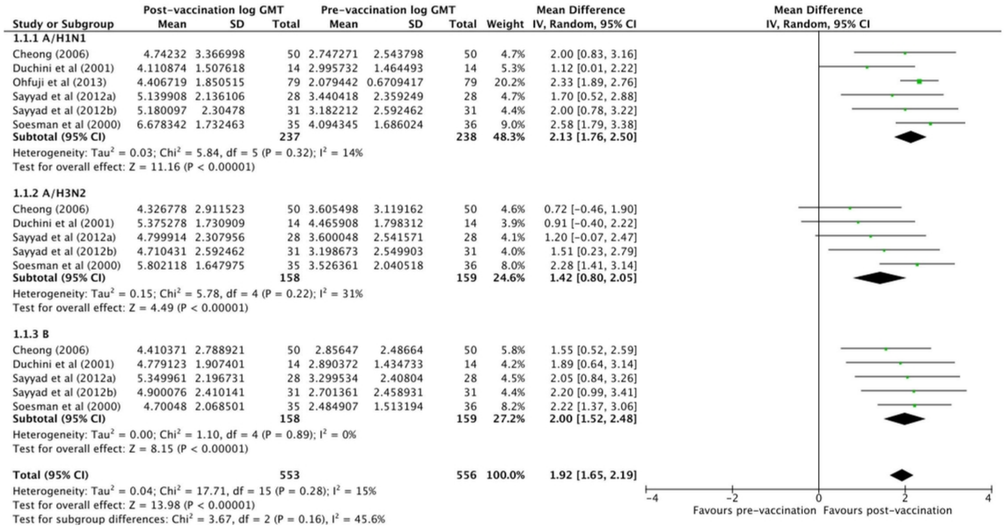

Figure 2. Serological response to influenza vaccination: mean difference in log HI antibody GMTs before and after vaccination. Note: Sayyad et al (2012a) includes cirrhotic patients and Sayyad et al (2012b) includes non-cirrhotic patients only.

96x50mm (300 x 300 DPI)

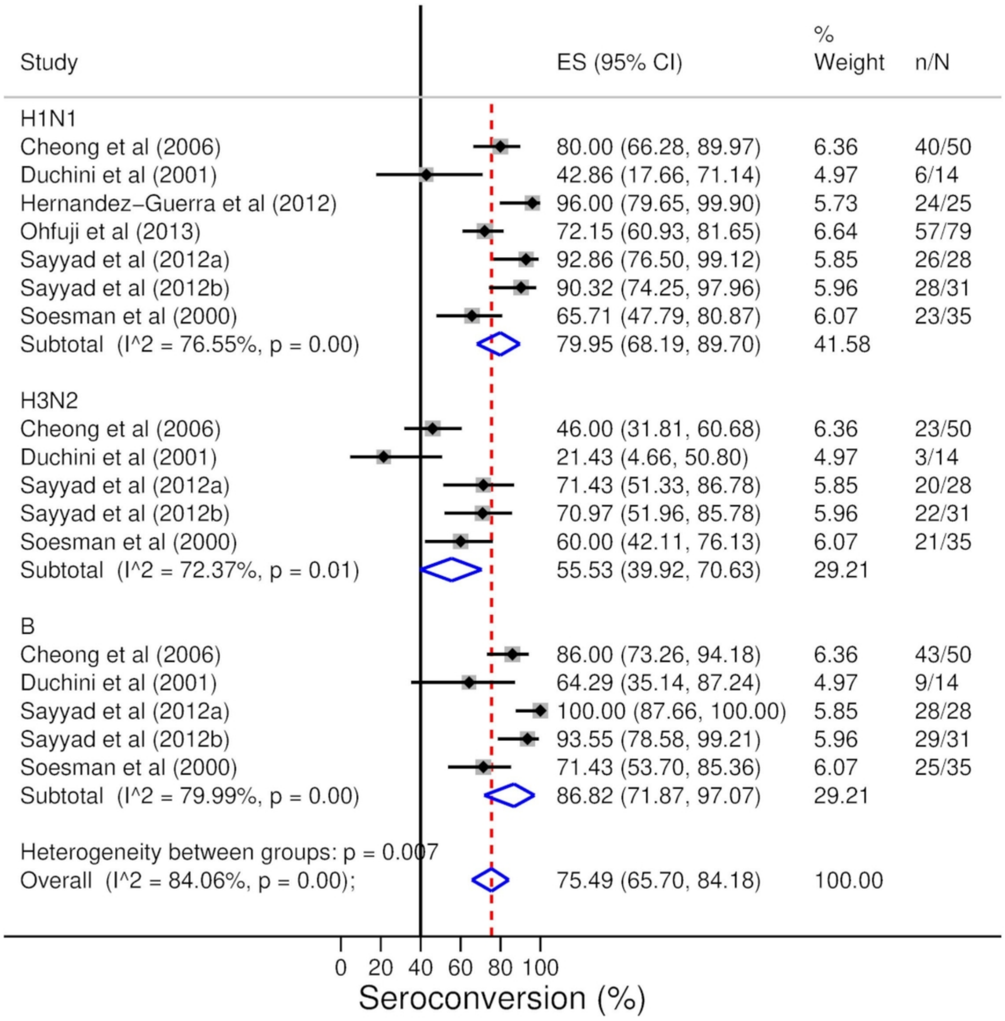

Figure 3. Serological response to influenza vaccination: seroconversion rate after vaccination. Note: Sayyad et al (2012a) includes cirrhotic patients and Sayyad et al (2012b) includes non-cirrhotic patients only.

96x97mm (300 x 300 DPI)

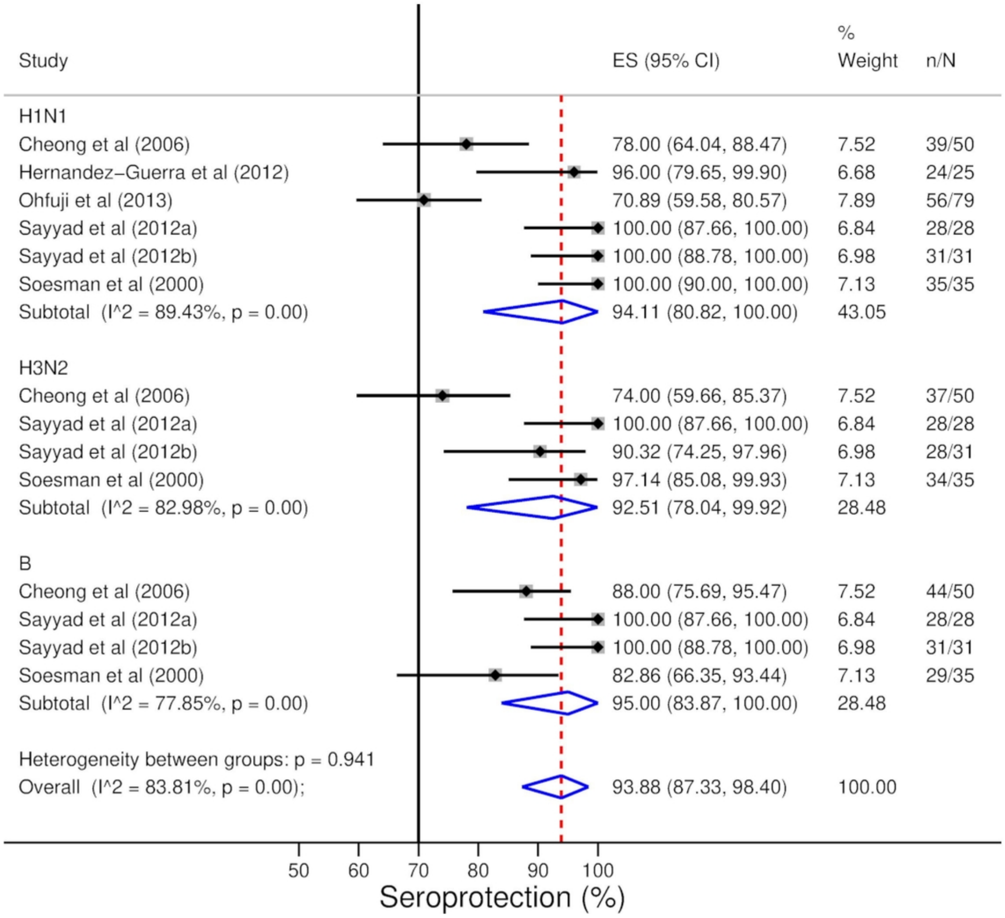

Figure 4. Serological response to influenza vaccination: seroprotection rate after vaccination. Note: Sayyad et al (2012a) includes cirrhotic patients and Sayyad et al (2012b) includes non-cirrhotic patients only.

96x87mm (300 x 300 DPI)

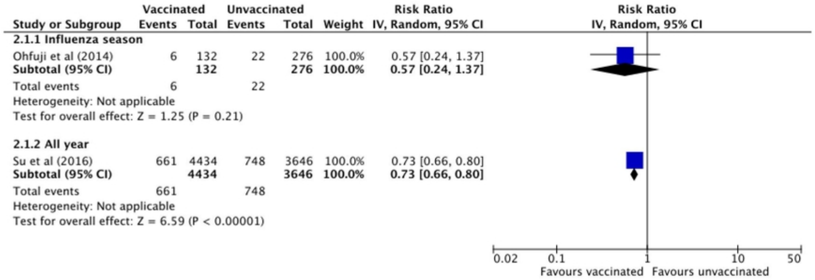

Figure 5. Effect of influenza vaccine on clinical outcomes: all-cause hospitalisation in vaccinated compared to unvaccinated liver disease patients. Note: Ohfuji et al (2014) investigated the effect of pandemic (monovalent) vaccine and Su et al (2016) of seasonal vaccine (against three or more virus subtypes).

90x47mm (300 x 300 DPI)

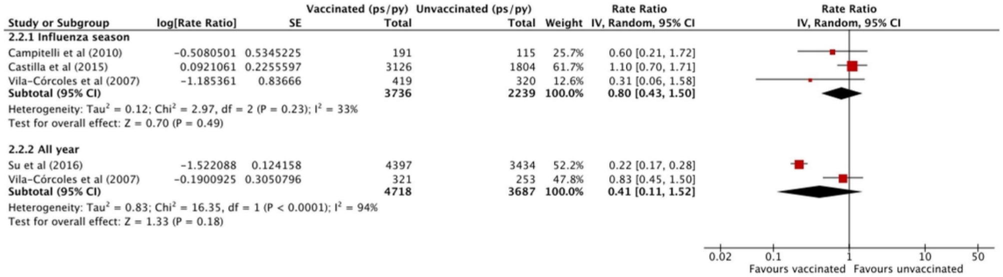

Figure 6. Effect of influenza vaccine on clinical outcomes: all-cause mortality in vaccinated compared to unvaccinated liver disease patients.

96x51mm (300 x 300 DPI)

SUPPLEMENTARY MATERIALS

Table 1. Differences between protocol and review

Table 2. Excluded test-negative case-control studies with hospitalised patients that included/are likely to have included liver patients

Table 3. Full MEDLINE (Ovid) search

Table 4. Medical subject headings used in Embase (Ovid), Pubmed and Cochrane Central Register of Controlled Trials

Table 5. Studies excluded due to uncertainty over whether the study included liver disease patients and the number of liver disease patients included

Table 6. Risk of bias in individual studies

Table 7. Risk of selective outcome reporting in the included studies

Table 8. GRADE evidence profile

Table 9. HI antibody responses in liver disease patients before and after vaccination

Figure 1. Seroprotection rate in liver disease patients before influenza vaccination

Figure 2. Sensitivity analysis: Mean difference in log HI antibody GMTs before and after vaccination in cirrhotic patients only

Figure 3. Sensitivity analysis: Seroconversion rate after vaccination in cirrhotic patients only

Figure 4. Sensitivity analysis: Seroprotection rate after vaccination in cirrhotic patients only

Figure 5. Sensitivity analysis: Seroprotection rate before vaccination in cirrhotic patients only

Figure 6. Additional analysis: Mean difference between HI antibody GMT after influenza vaccination in liver disease patients compared to healthy individuals

Figure 7. Additional analysis: Mean difference between geometric mean HI antibody titres before influenza vaccination: liver disease patients compared to healthy individuals

Figure 8. Additional analysis: Mean difference between geometric mean HI antibody titre ratio after influenza vaccination in liver disease patients compared to healthy individuals

Figure 9. Additional analysis: Probability of seroconversion after influenza vaccination in liver disease patients compared to healthy individuals

Figure 10. Additional analysis: Probability of seroprotection after influenza vaccination in liver disease patients compared to healthy individuals

Figure 11. Additional analysis: Difference in seroprotection rate before vaccination in liver disease patients compared to healthy individuals

Table 10. Risk of all-cause mortality in the general population of the included studies where liver patients formed a subgroup

References

For peer review only

Table 1. Differences between protocol and review

Review section	Protocol	Review
**Search strategy**	1. The review protocol was specified in advance as part of a wider review plan that also includes a study of the effectiveness of pneumococcal vaccine. The search strategy in this wider plan includes search terms for both influenza vaccine and pneumococcal vaccine.<br><br>2. We planned to search the Cochrane Central Register of Controlled Trials (CENTRAL), MEDLINE, EMBASE, PubMed, The Cochrane Hepato-Biliary Group Specialised Register and The Cochrane Acute Respiratory Infections Group Register of Trials.<br><br>3. Our planned search terms included filters for adults and study type.<br><br>4. We planned to complement the electronic searches by manually searching the reference lists of included studies for additional references.	1. To keep the message of the manuscript clear and concise, this manuscript focuses on influenza vaccine. The search and the results reported here include only those using search terms for influenza vaccine and not for pneumococcal vaccine.<br><br>2. MEDLINE, EMBASE and PubMed were searched as planned but instead of CENTRAL, The Cochrane Hepato-Biliary Group Specialised Register and The Cochrane Acute Respiratory Infections Group Register of Trials, only CENTRAL was searched to maintain the specificity of the search. In the preliminary searches, the number of records found in CENTRAL and the other wide focus databases was large and loosening the search terms for the two specialist sub-collections within CENTRAL was not deemed necessary (the records included in these sub-collections are also in included in CENTRAL and using the same search terms in CENTRAL and the two specialist sub-databases would result in duplicate records only).<br><br>3. In the final search, we decided to additionally filter for publication type to increase the specificity of the search.<br><br>4. In the final search, we further complemented the search by manually searching also through the recent WHO, ECDC and CDC influenza vaccine guidelines for additional references.
**Risk of bias assessment and quality of evidence**	1. We planned to use Cochrane Collaboration's tool to assess risk of bias in all studies.<br><br>2. We planned to consider age, sex, severity and aetiology of liver disease as the most important confounders in the assessment of observational studies.	1. In the review, we used Cochrane Collaboration's tool to assess risk of bias in randomized controlled trials and followed their recommendation to use the Newcastle-Ottawa scale to assess risk of bias in observational studies.<br><br>2. In the review, based on the recommendations from the peer-review of the protocol, we additionally considered as potential confounders: chronic co-morbidities and previous seasonal influenza vaccination during the same season as additional confounders in the assessment of studies that investigated the clinical effects of a pandemic (monovalent) vaccine. In assessing the quality of evidence, we considered the lack of clear effect in the influenza season as a sign of imprecision of the effect estimate of clinical outcomes.
**Data analysis**	1. We did not plan to report serological outcome measures categorised by virus subtypes.	1. In the review, we reported the serological outcome measures categorised by virus subtype as there may be differences in antibody response between them.

For peer review only - http://bmjopen.bmj.com/site/about/guidelines.xhtml

Table 2: Excluded test-negative case-control studies with hospitalised patients that included/are likely to have included liver patients

Study	Location	Cases	Controls	Liver patients
Castilla et al (2011)[1]	Spain	Patients with medically-attended influenza-like illness (MA-ILI), negative for influenza in laboratory tests.	Patients with medically-attended influenza-like illness (MA-ILI), positive for influenza in laboratory tests.	Study included hospitalised patients (34 cases, 280 controls. Eligibility criteria included patients with cirrhosis but number of patients with cirrhosis not reported.
Cheng et al (2013)[2]	Australia	Patients hospitalised for ILI, negative for influenza in laboratory tests.	Patients hospitalised for ILI, positive for influenza in laboratory tests.	Study included 38 cases and 71 controls with cirrhosis. Number of vaccinated/unvaccinated cirrhosis patients not reported.
Cheng et al (2014)[3]	Australia	Patients hospitalised for ILI, negative for influenza in laboratory tests.	Patients hospitalised for ILI, positive for influenza in laboratory tests.	Study included 38 cases with cirrhosis, number of controls with cirrhosis not reported. Number of vaccinated/unvaccinated cirrhosis patients not reported.
Choi et al (2013)[4]	South Korea	Patients with medically-attended influenza-like illness (MA-ILI), negative for influenza in laboratory tests.	Patients with medically-attended influenza-like illness (MA-ILI), positive for influenza in laboratory tests.	Study included 19 cases and 25 controls with liver disease. Number of hospitalised liver patients not reported. Number of vaccinated/unvaccinated liver patients not reported.
Choi et al (2015)[5]	South Korea	Patients with medically-attended influenza-like illness (MA-ILI), negative for influenza in laboratory tests.	Patients with medically-attended influenza-like illness (MA-ILI), positive for influenza in laboratory tests.	Study included 40 cases and 39 controls with liver disease. Number of hospitalised liver patients not reported. Number of vaccinated/unvaccinated liver patients not reported.

For peer review only

Table 3. Full MEDLINE (Ovid) search

Search concept	Search terms
Influenza vaccine	1. exp Influenza Vaccines/
	2. exp Influenza, Human/pc [Prevention & Control]
	3. ((anti?influenza or influenza or seasonal or anti?flu or flu) adj5 (vaccin* or immuni*)).mp.
	4. ((TIV or QIV or trivalent or quadrivalent or 3?valent or 4?valent) adj5 (vaccin* or immuni*)).mp.
	5. 1 or 2 or 3 or 4
Liver disease	6. exp Liver Diseases/
	7. ((liver or hepat*) adj3 disease*).mp.
	8. ("chronic liver" or "chronic hepat*").mp.
	9. cirrho*.mp.
	10. 6 or 7 or 8 or 9
Adult participants	11. exp Adult/
	12. adult.mp.
	13. (middle?aged or aged).sh.
	14. age*.tw.
	15. 11 or 12 or 13 or 14
Randomised controlled trials	16. randomized controlled trial.pt.
	17. randomi*.ab,ti.
	18. randomly.ab,ti.
	19. controlled clinical trial.pt.
	20. trial.ab,ti.
	21. groups.ab,ti.
	22. drug therapy.fs.
	23. placebo.ab,ti.
	24. 16 or 17 or 18 or 19 or 20 or 21 or 22 or 23
	25. (review or "practice guideline" or "patient education handout" or guideline or editorial or "case reports").pt.
	26. 25 not (25 and (16 and 19))
	27. Animals/
	28. Humans/
	29. 27 not (27 and 28)
	30. 24 not (26 or 29)
Case-control and cohort studies	31. Epidemiologic Studies/
	32. exp Case control studies/
	33. exp Cohort studies/
	34. Longitudinal studies/

For peer review only - http://bmjopen.bmj.com/site/about/guidelines.xhtml

	35. Follow up studies/
	36. Prospective studies/
	37. Retrospective studies/
	38. Control groups/
	39. Matched-Pair Analysis/
	40. (Case* adj5 control*).ti,ab,kw.
	41. (Case* adj5 comparison*).ti,ab,kw.
	42. Control group*.ti,ab,kw.
	43. (Cohort adj (study or studies)).ti,ab.
	44. Cohort anal*.ti,ab.
	45. (Follow up adj (study or studies)).ti,ab.
	46. (Observational adj (study or studies)).ti,ab.
	47. Longitudinal.ti,ab.
	48. Retrospective.ti,ab.
	49. Prospective.ti,ab.
	50. 31 or 32 or 33 or 34 or 35 or 36 or 37 or 38 or 39 or 40 or 41 or 42 or 43 or 44 or 45 or 46 or 47 or 48 or 49
	51. (review or "practice guideline" or "patient education handout" or guideline or editorial or "case reports").pt.
	52. 51 not (51 and (31 or 32 or 33 or 34 or 35 or 36 or 37 or 38 or 39))
	53. Animals/
	54. Humans/
	55. 53 not (53 and 54)
	56. 50 not (52 or 55)
	57. 30 or 56
	58. 5 and 10 and 15 and 57
Results: Liver disease-specific search	59. limit 58 to medline
	60. 5 and 15 and 30
Results: Influenza vaccine studies search	61. limit 60 to medline

For peer review only

Table 4. Medical subject headings used in Embase (Ovid), Pubmed and Cochrane Central Register of Controlled Trials

Database	Medical subject headings				
	**Influenza**	**Liver disease**	**Adults**	**Study type**	**Animal/Human study**
Embase (Ovid)	exp influenza vaccine/<br>exp influenza/pc [Prevention]	exp liver disease/<br>exp liver cirrhosis/	exp adult/	randomized controlled trial/<br>controlled clinical trial/<br>epidemiology/<br>exp case control study/<br>exp cohort analysis/<br>longitudinal study/<br>follow up/<br>prospective study/<br>retrospective study/<br>control group/	exp animal/<br>exp animal cell/<br>exp animal disease/<br>exp animal experiment/<br>exp animal model/<br>exp animal tissue/<br>human/
Pubmed	Influenza Vaccines[MH]<br>"influenza human/prevention and control"[MH]	liver disease[MH]	"adult"[MH]<br>"middle aged"[MH:noexp]<br>"aged"[MH:noexp]	"Epidemiologic Studies"[MH:noexp]<br>"Case-Control Studies"[MH]<br>"retrospective studies"[MH:noexp] "Control Groups"[MH:noexp]<br>"matched-pair analysis"[MH:noexp]<br>"longitudinal studies"[MH:noexp]<br>"follow-up studies"[MH:noexp] "prospective studies"[MH:noexp]<br>"cohort studies"[MH]	"animals"[MH] "humans"[MH]
Cochrane Central Register of Controlled Trials	[mh "Influenza Vaccines"]<br>[mh "Influenza, human"/PC]	[mh "Liver Diseases"]	[mh Adult]	Not relevant	Not relevant

For peer review only

Table 5. Studies excluded due to uncertainty over whether the study included liver disease patients and the number of liver disease patients included

Study	Location	Study design	Review outcomes	Reason for uncertainty and notes
Castilla et al (2012)[6]	Spain	Cohort study	Influenza illness/ILI-related hospitalisations	The inclusion criteria included patients with liver disease. to confirm the actual study population also included liver disease patients we contacted but could not reach the corresponding author.
Castilla et al (2013)[7]	Spain	Cohort study	Influenza illness/ILI-related hospitalisations	The inclusion criteria included patients with liver disease. to confirm the actual study population also included liver disease patients we contacted but could not reach the corresponding author.
DiazGranados et al (2013)[8]	United States	Randomised, controlled trial	Serological response	Study population excluded patients with known or suspected hepatitis B or C infection and patients who abused alcohol but included patients with hepatobiliary disorders: 522 in high dose vaccination group and 257 in normal dose vaccination group. We attempted to contact but could not reach the corresponding author.
Emborg et al (2012)[9]	Denmark	Cohort study	Influenza illness/ILI-related hospitalisations	The inclusion criteria included patients with liver disease. To confirm the actual study population also included liver disease patients we contacted but could not reach the corresponding author.
Mangtani et al (2004)[10]	United Kingdom	Cohort study	Acute respiratory illness-related hospitalization<br><br>Acute respiratory illness-related mortality	The inclusion criteria included patients with liver disease as part of the risk group for influenza complications. We contacted the corresponding author to confirm the actual study population also included liver disease patients. The patient data for the study had only been provided on the level of at-risk individuals not on the level of a single disease and so the author had no access to this information.
Olafsdottir et al (2018)[11]	Iceland	Cohort study	Serological response	The study population was the general population, however, the study data collected included a record of primary biliary cirrhosis. We contacted but could not reach the corresponding author to confirm the actual study population also included liver disease patients.
Simpson et al (2013)[12]	Scotland, United Kingdom	Cohort study	All-cause hospitalisation<br><br>All-cause mortality<br><br>Acute respiratory illness-related hospitalisation (pneumonia & influenza)<br><br>Acute respiratory illness-related mortality (pneumonia & influenza)	Number of patients was not reported. Liver disease patients contributed 3727 person-seasons vaccinated/5377 person-seasons unvaccinated (over 9 seasons) to the study. The study, however, also included under 18 year-olds and ≤14 year-olds contributed 4988 person-seasons vaccinated and 281550 person-seasons unvaccinated to the study. Authors were contacted to confirm the number of patients and to obtain subgroup data, however, they were unable to provide this information. It is likely liver disease was more common among over ≥18 year-olds and so the study potentially included over 400 vaccinated and over 500 unvaccinated adult liver disease patients, however, this could not be confirmed.
Wang et al (2004)[13]	Taiwan	Cohort study	All-cause hospitalisation<br><br>Acute respiratory illness-related hospitalisation (pneumonia & influenza)<br><br>Liver disease complication-related	The inclusion criteria included patients with liver disease as part of the risk group for influenza complications. It seems that the patient data for the study had only been provided on the level of at-risk individuals not on the level of a single diseases and so the authors had no access to this information. We attempted to contact but could not reach the corresponding author to confirm.

For peer review only - http://bmjopen.bmj.com/site/about/guidelines.xhtml

			hospitalisation (chronic liver disease and cirrhosis)	
Wang et al (2007)[14]	Taiwan	Cohort study	All-cause mortality<br><br>Acute-respiratory illness-related mortality (pneumonia)<br><br>Liver disease complication-related mortality (chronic liver disease)	The inclusion criteria included patients with liver disease as part of the risk group for influenza complications. It seems that the patient data for the study had only been provided on the level of at-risk individuals not on the level of a single diseases and so the authors had no access to this information. We contacted but could not reach the corresponding author to confirm.
Zivich et al (2017)[15]	United States	Cohort study	All-cause mortality	The study included at least 45 vaccinated and 41 unvaccinated liver disease patients. The study, however, also included under 18 year-old patients (121 were ≤4 year-old and 144 were ≤24 year-old. Authors were contacted to confirm the number of patients and to obtain subgroup data, however, they no longer have access to the study data and were unable to provide this information. It is likely liver disease was more common among over ≥18 year-olds and so the study potentially included over 40 adult liver disease patients in each group. This, however, could not be confirmed.

For peer review only

Table 6. Risk of bias in the included studies

A. Risk of bias in observational studies of serological outcomes

**Study**	**Selection**[a]<br>*** = low risk<br>** = moderate risk<br>- and * = high risk	**Outcome**<br>*** = low risk<br>** = moderate risk<br>- and * = high risk
Cheong et al (2006)	*** Low	* High[b,c]
Duchini et al (2001)	*** Low	** Moderate[c]
Hernández-Guerra et al (2012)	*** Low	*** Low
Ohfuji et al (2013)	*** Low	*** Low
Sayyad et al (2012)	** Moderate[d]	** Moderate[b]
Soesman et al (2000)	** Moderate[e]	** Moderate[c]

[a] Selection was assessed considering the selection of the liver disease group only.
[b] High drop-out rate.
[c] No description of independent blind assessment of outcome.
[d] Source/selection of liver patient groups not clear.
[e] Pre-vaccination influenza infection status not clear and pre-vaccination seroprotection rates high.

B. Risk of bias in observational studies of clinical outcomes

**Study**	**Selection**<br>**** = low risk<br>** and *** = moderate risk<br>- and * = high risk	**Comparability**[a]<br>**** and ***** = low risk<br>** and *** = moderate risk<br>- and * = high risk	**Outcome**<br>*** = low risk<br>** = moderate risk<br>- and * = high risk
Campitelli et al (2010)	*** Moderate[b]	Unclear[c]	*** Low
Castilla et al (2105)	**** Low	Unclear[c]	*** Low
Ohfuji et al (2014)	*** Moderate[b]	**** Moderate[d, e]	*** Low
Su et al (2016)	**** Low	***** Low	*** Low
Vila-Córcoles et al (2007)	**** Low	Unclear[c]	*** Low

[a] Comparability was considered between vaccinated and unvaccinated groups. Confounders considered were: age, sex, liver disease aetiology, liver disease severity, chronic co-morbidities and previous seasonal influenza vaccination in the same season if study vaccine pandemic vaccine.
[b] Self-reported vaccination (although in Campitelli et al 2010, vaccination was ascertained from physician billing claims, these are based on self-reporting).
[c] No information on characteristics of the vaccinated and unvaccinated groups.
[d] Sex not comparable between groups.
[e] Seasonal influenza vaccination in the same season than pandemic vaccine given to some but balance unclear between groups.

C. Risk of bias in randomised controlled studies

**Study**	**Random sequence generation**	**Allocation concealment**	**Blinding of participants and personnel**	**Blinding of outcome assessment**	**Incomplete outcome data**	**Selective reporting**	**Other bias**
Song et al (2007)	Low	Low	High[a]	Low	High[b]	Low	Unclear[c]

[a] Patients were not blinded and could not have been blinded when only the intervention group received the vaccination.
[b] High drop-out rate.
[c] Details on study funding and conflicts of interest were not provided.

For peer review only - http://bmjopen.bmj.com/site/about/guidelines.xhtml

Table 7. Risk of selective outcome reporting in the included studies

Risk of bias arising from the lack of inclusion of results when a study was excluded from a meta-analysis or not fully reported in a review because the data were unavailable.

Level of reporting: √ = Full reporting of results, × = No reporting of results, o = Partial reporting of results

Classifications and descriptions of risk of bias found in the assessment of missing or complete outcome reporting:

Clear that the outcome was measured and analysed:	Level of reporting	Risk of bias
C = Trial report states that outcome was analysed but insufficient data were presented for the trial to be included in meta-analysis or to be considered to be fully tabulated.	o	Low Risk
Clear that the outcome was measured:		
E = Clear that the outcome was measured. Judgment says outcome likely to have been analysed but not reported because of non-significant results.	×	High Risk
Unclear whether the outcome was measured:		
G = Not mentioned but clinical judgment says likely to have been measured and analysed but not reported on the basis of non-significant results.	×	High Risk
H = Not mentioned but clinical judgment says unlikely to have been measured at all.	×	Low Risk
Clear that the outcome was not measured:		
I = Clear that the outcome was not measured.	×	No Risk

For peer review only

A. Risk of selective outcome reporting in studies of serological outcomes

	Review secondary outcomes				Other outcomes reported
**Study**	**GMT**	**GMTR**	**Seroprotection %**	**Seroconversion %**	
Cheong et al (2006)	√	×<br>E – High risk[a]	o<br>C – Low risk[b]	o<br>C – Low risk[b]	Cellular immune response to vaccine (interferon-γ secretion)
Duchini et al (2001)	√	√	×<br>E – High risk[c]	√	None
Hernández-Guerra et al (2012)	o<br>C – Low risk[d]	√	o<br>C – Low risk[e]	√	Systemic adverse events, acceptance and tolerance of vaccination
Ohfuji et al (2013)	√	√	√	√	Serious adverse events
Sayyad et al (2012)	√	×<br>E – High risk[a]	o<br>C – Low risk[f]	o<br>C – Low risk[f]	None
Soesman et al (2000)	√	×<br>E – High risk[a]	√	√	Serological response (HI antibody) to a non-vaccine strain

[a] Pre- and post-vaccination GMT was measured and it is likely that their ratio was analysed but not reported.

[b] Only percentages were reported so we have assumed the highest possible number of individuals closest to the reported percentage for pre-vaccination measures and lowest possible number of individuals closest to the reported percentage for post-vaccination measures.

[c] Pre- and post-vaccination GMT was measured and seroconversion rate was analysed. It is likely that the seroprotection rate was also analysed but not reported.

[d] Pre-vaccination GMT was measured but not fully reported. It is likely this was low as only one individual had 1:40 antibody level (seroprotective level).

[e] Pre-vaccination seroprotection rate was not fully reported but the rate was low only one individual had a seroprotective level of antibodies. It was not specified in which comparison group so we have assumed it was one individual in each group.

[f] The percentages and numbers of individuals did not add up in the reported results so we have assumed the highest possible number of individuals closest to the reported percentage for pre-vaccination measures and lowest possible number of individuals closest to the reported percentage for post-vaccination measures.

For peer review only

B. Risk of selective outcome reporting in studies of clinical outcomes

	Review primary outcomes		Review secondary outcomes						Other outcomes reported
**Study**	**All-cause hospitalisation**	**All-cause mortality**	**Acute respiratory illness-related hospitalisation**	**Influenza illness/ILI-related hospitalisation**	**Liver disease complication related-hospitalisation**	**Acute respiratory illness-related mortality**	**Influenza illness/ILI-related mortality**	**Liver disease complication related-mortality**	
Campitelli et al (2010)	×<br>H – Low risk[a]	√	o<br>C – Low risk[b]	×<br>I – No risk	×<br>I – No risk	×<br>I – No risk	×<br>I – No risk	×<br>I – No risk	None
Castilla et al (2105)	×<br>I – No risk	√	×<br>I – No risk	×<br>I – No risk	×<br>I – No risk	×<br>I – No risk	×<br>I – No risk	×<br>I – No risk	ILI incidence
Ohfuji et al (2014)	√	×<br>I – No risk	×<br>I – No risk	×<br>I – No risk	×<br>I – No risk	×<br>I – No risk	×<br>I – No risk	×<br>I – No risk	None
Song et al (2007)	×<br>I – No risk	×<br>G – High risk[c]	×<br>I – No risk	×<br>I – No risk	×<br>I – No risk	×<br>I – No risk	√	×<br>I – No risk	ILI incidence, clinical manifestations of ILI, liver disease complications secondary to influenza
Su et al (2016)	√	√	×<br>I – No risk	×<br>I – No risk	√	×<br>I – No risk	×<br>I – No risk	×<br>I – No risk	Pneumonia & Influenza incidence, heart disease related hospitalisation, respiratory failure, intensive care unit admission
Vila-Córcoles et al (2007)	×<br>I – No risk	√	×<br>I – No risk	×<br>I – No risk	×<br>I – No risk	×<br>I – No risk	×<br>I – No risk	×<br>I – No risk	ILI incidence

[a] The main interest in the study was all-cause mortality and cause-specific hospitalisation outcome was used to understand the whether the mortality may have been linked to influenza. Accessing the data required an approved study protocol and it is unlikely that data to study all-cause hospitalisation was available when not included in the protocol.

[b] Study authors were unable to provide exact event numbers for this outcome as their institution's privacy policy does not allow providing data in case of five or fewer events.

[c] Authors did measure influenza-related mortality in this trial and it is likely that they also measured all-cause mortality. Authors were contacted but we were unable to obtain a response.

For peer review only

For peer review only - http://bmjopen.bmj.com/site/about/guidelines.xhtml

Table 8. GRADE evidence profile

Effectiveness of influenza vaccination in preventing all-cause hospitalisation and all-cause mortality in adults with chronic liver disease

Number of studies (design)	Limitations	Inconsistency	Indirectness	Imprecision	Publication bias	No vaccine	Vaccine	Relative risk/ Rate ratio	Control risk/ Control rate	Risk difference	Quality
All-cause hospitalisation, influenza season											
1 (Cohort study)	Serious limitations[a]	Not judged (only 1 study)	No serious indirectness	Serious imprecision[b]	Suspected[c]	22/276	6/132	0·57 (0·24, 1.37)	80 per 1000	-0·03 (-0·08, 0·01)	Very low
All-cause hospitalisation, all year											
1 (Cohort study)	No serious limitations	Not judged (only 1 study)	No serious indirectness	No serious imprecision	Suspected[c]	748/3646	661/4434	0·73 (0·66, 0·80)	205 per 1000	-0·06 (-0·07-0·04)	Very low
All-cause mortality, influenza season											
3 (Cohort study)	Serious limitations[d]	Serious inconsistency[e]	No serious indirectness	Serious imprecision[f]	Suspected[c]	42/2239 person-seasons	66/3736 person-seasons	0·80 (0·43, 1·50)	17 per 1000 person-seasons	-0·22 (-0·85, 0·41)	Very low
All-cause mortality, all year											
2 (Cohort study)	Serious limitations[g]	No serious inconsistency	No serious indirectness	Serious imprecision[e,f]	Suspected[c]	318/3687 person-years	105/4718 person-years	0·41 (0·11, 1·52)	85 per 1000 person-years	-0·89 (-2·19, 0·42)	Very low

[a] Moderate risk of bias in selection and comparability of groups.
[b] Imprecision of the estimate with large 95% CIs.
[c] Due to study language restrictions in the review and exclusion of studies due to uncertainty of eligibility.
[d] Moderate risk of bias in selection in one study and unclear bias in comparability of groups in all studies.
[e] Study effect estimates varied largely.
[f] Control risks varied largely between studies.
[g] Unclear bias in comparability of groups in one study and uncertain timing of the vaccine effect due to Kaplan-Meier plot showing increasing difference in mortality rates outside the influenza season.

For peer review only - http://bmjopen.bmj.com/site/about/guidelines.xhtml

Table 9. HI antibody responses in liver disease patients before and after vaccination

Influenza A (H1N1)						
**Study**	**Pre-vaccination GMT (95% CI)**	**Post-vaccination GMT (95% CI)**	**GMTR**	**Seroconversion rate (%)**	**Pre-vaccination seroprotection rate (%)**	**Post-vaccination seroprotection rate (%)**
Cheong et al (2006)[16]	15·6 (SE ±1·8)	114·7 (SE ±4·1)	Not reported	40/50 (80%)	5/50 (10%)	39/50 (79%)
Duchini et al (2001)[17]	20·0 (10.0, 40.0)	61·0 (30·0, 125·0)	3·0 (1·7, 5·4)	6/14 (43%)	Not reported	Not reported
Hernández-Guerra et al (2012)[18]	Not reported	229·0 (55·0, 957·0)	Treated: 43·0 (10·0, 180·0)<br>Untreated: 32·0 (7·0, 137·0)	24/25 (96%)	1/25 (4%)	24/25 (96%)
Ohfuji et al (2013)[19]	8·0 (7·0, 9·0)	226·0 (69·0-743·0)	10·3 (7·2, 14·9)	57/59 (97%)	4/79 (5%)	56/79 (71%)
Sayyad et al (2012)[20]	Cirrhotic: 31·2 (SE ±2·0)<br>HBV carrier: 24·1 (SE ±2·4)	Cirrhotic: 170·7 (SE ±1·6)<br>HBV carrier: 177.7 (SE ±1·8)	Not reported	Cirrhotic: 26/28 (93%)<br>HBV carrier: 28/31 (90%)	Cirrhotic: 19/28 (68%)<br>HBV carrier: 16/31 (52%)	Cirrhotic: 28/28 (100%)<br>HBV carrier: 31/31 (100%)
Soesman et al (2000)[21]	60·0 (37·0, 97·0)	795·0 (484·0, 1303·0)	Not reported	23/35 (66%)	26/36 (72%)	35/35 (100%)
Influenza A (H3N2)						
**Study**	**Pre-vaccination GMT (95% CI)**	**Post-vaccination GMT (95% CI)**	**GMTR**	**Seroconversion rate (%)**	**Pre-vaccination seroprotection rate (%)**	**Post-vaccination seroprotection rate (%)**
Cheong et al (2006)[16]	36·8 (SE ±3·2)	75·7 (SE ±2·6)	Not reported	23/50 (46%)	13/50 (26%)	37/50 (74%)
Duchini et al (2001)[17]	87·0 (37·0, 203.0)	216·0 (95·0, 489·0)	2·4 (1·6, 3·8)	3/14 (21%)	Not reported	Not reported
Sayyad et al (2012)[20]	Cirrhotic: 36·6 (SE ±2·4)<br>HBV carrier: 24·5 (SE ±2·3)	Cirrhotic: 121·5 (SE ±1·9)<br>HBV carrier: 111·1 (SE ±2·4)	Not reported	Cirrhotic: 20/28 (71%)<br>HBV carrier: 22/31 (71%)	Cirrhotic: 20/28 (71%)<br>HBV carrier: 16/31 (52%)	Cirrhotic: 28/28 (100%)<br>HBV carrier: 28/31 (90%)
Soesman et al (2000)[21]	34·0 (19·0, 61·0)	331·0 (207·0, 531·0)	Not reported	21/35 (60%)	21/36 (58%)	34/35 (97%)
Influenza B						
**Study**	**Pre-vaccination GMT (95% CI)**	**Post-vaccination GMT (95% CI)**	**GMTR**	**Seroconversion rate (%)**	**Pre-vaccination seroprotection rate (%)**	**Post-vaccination seroprotection rate (%)**
Cheong et al (2006)[16]	17·4 (SE ±1·7)	82·3 (SE ±2·3)	Not reported	43/50 (86%)	5/50 (10%)	44/50 (88%)

For peer review only

Duchini et al (2001)[17]	18·0 (9·0, 35·0)	119·0 (48·0, 292·0)	6·0 (2·6, 19·0)	9/14 (64%)	Not reported	Not reported
Sayyad et al (2012)[20]	Cirrhotic: 27·1 (SE ±2·1)<br>HBV carrier: 14·9 (SE ±2·1)	Cirrhotic: 210·6 (SE ±1·7)<br>HBV carrier: 134·3 (SE ±2·0)	Not reported	Cirrhotic: 28/28 (100%)<br>HBV carrier: 29/31 (94%)	Cirrhotic: 18/28 (64%)<br>HBV carrier: 6/31 (19%)	Cirrhotic: 28/28 (100%)<br>HBV carrier: 31/31 (100%)
Soesman et al (2000)[21]	12·0 (8·0, 19·0)	110·0 (61·0, 199·0)	Not reported	25/35 (71%)	10/36 (28%)	29/35 (83%)

For peer review only

Figure 1. Seroprotection rate in liver disease patients before influenza vaccination

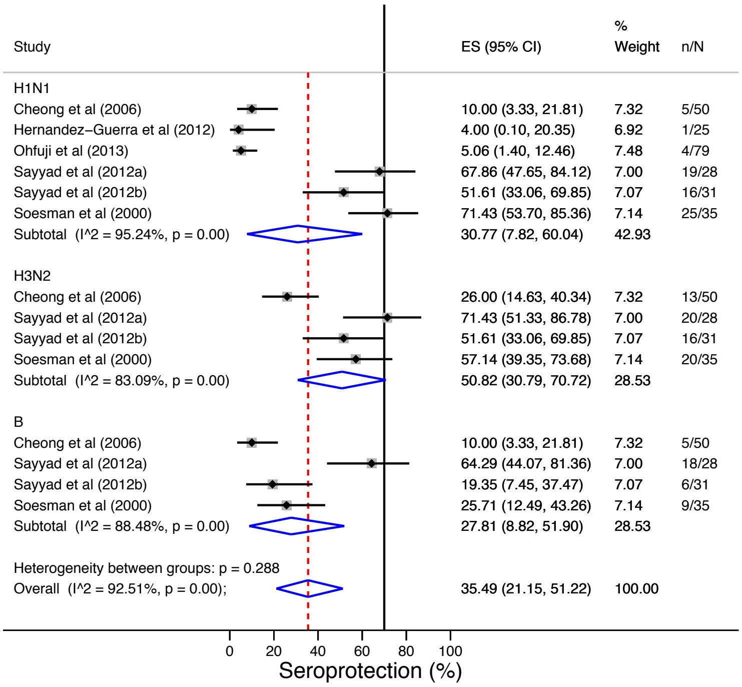

Figure 2. Sensitivity analysis: Mean difference in log HI antibody GMTs before and after vaccination in cirrhotic patients only

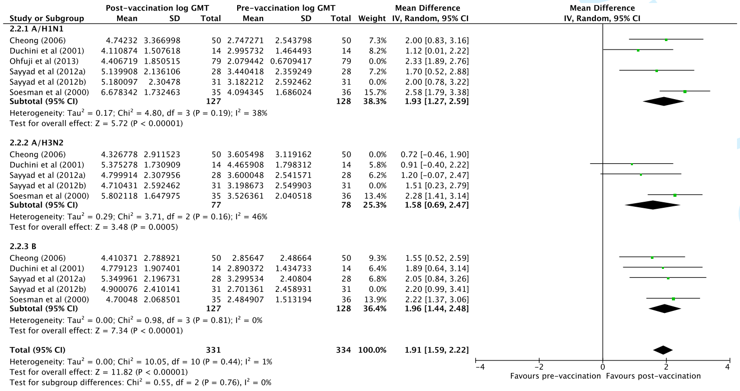

Note: Liver patient results from the studies with non-cirrhotic patients only and studies with a mix of cirrhotic and non-cirrhotic patients are visible but do not affect the outcome of analysis. Only the results from the studies with cirrhotic patients Cheong et al (2006), Duchini et al (2001) Sayyad et al (2012a) and Soesman are pooled in this analysis. Sayyad et al (2012b) includes non-cirrhotic patients only.

For peer review only - http://bmjopen.bmj.com/site/about/guidelines.xhtml

Figure 3. Sensitivity analysis: Seroconversion rate after vaccination in cirrhotic patients only

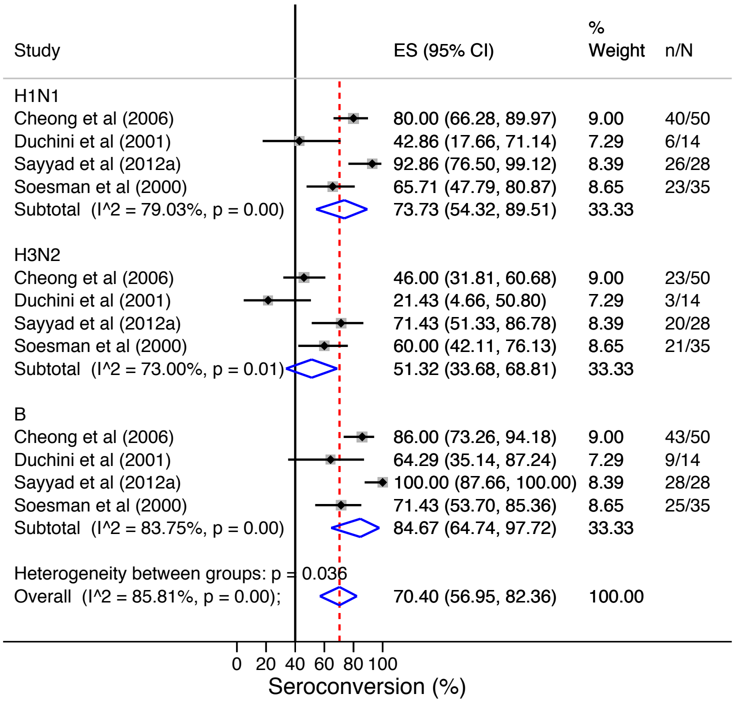

Figure 4. Sensitivity analysis: Seroprotection rate after vaccination in cirrhotic patients only

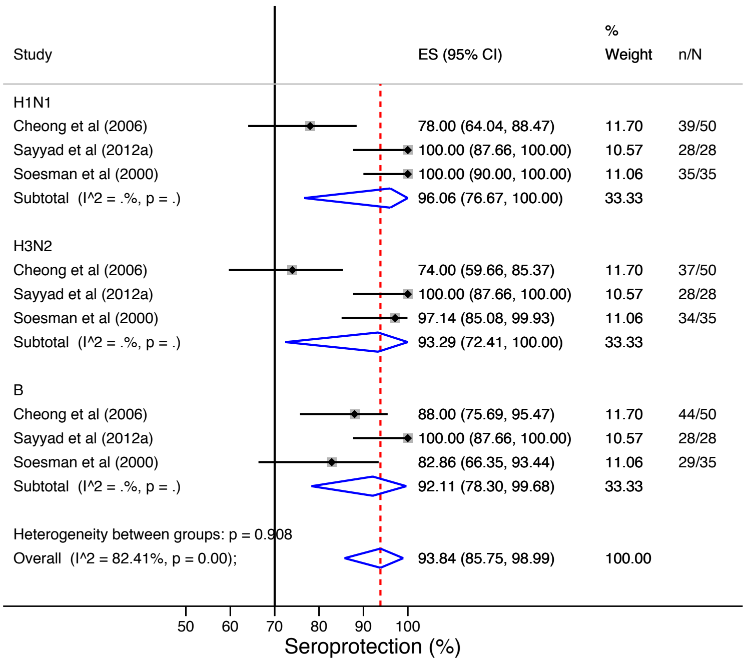

For peer review only - http://bmjopen.bmj.com/site/about/guidelines.xhtml

Figure 5. Sensitivity analysis: Seroprotection rate before vaccination in cirrhotic patients only

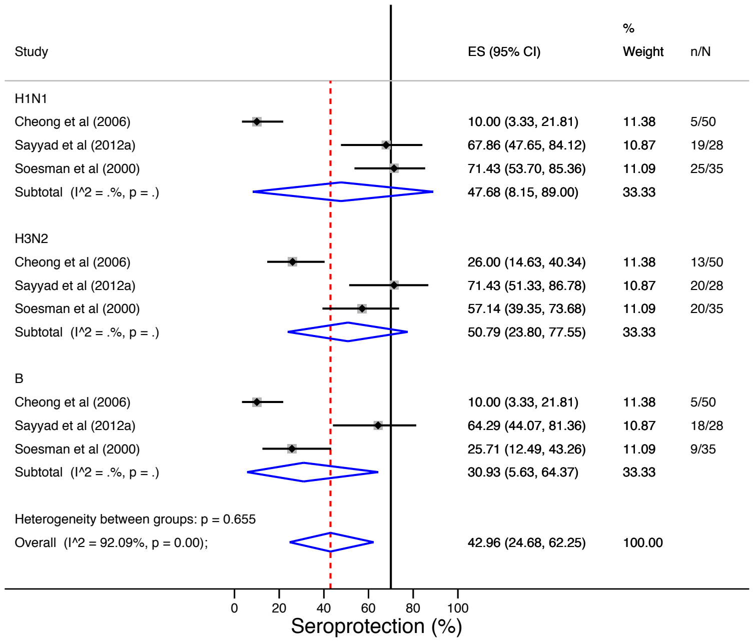

Figure 6. Additional analysis: Mean difference between HI antibody GMT after influenza vaccination in liver disease patients compared to healthy individuals

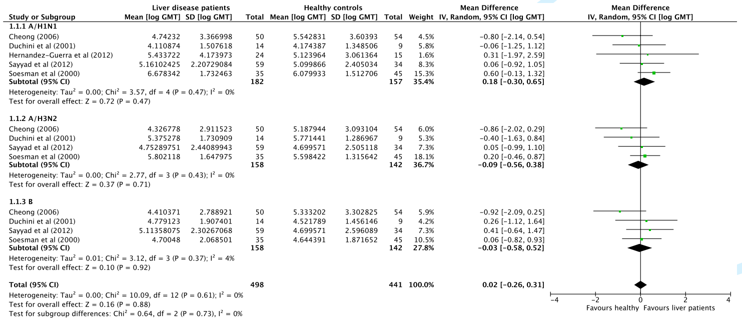

Note: Results from the non-cirrhotic and cirrhotic patient groups in Sayyad et al (2012) have been combined using the formulae recommended in Cochrane Handbook [22].

Figure 7. Additional analysis: Mean difference between geometric mean HI antibody titres before influenza vaccination in liver disease patients compared to healthy individuals

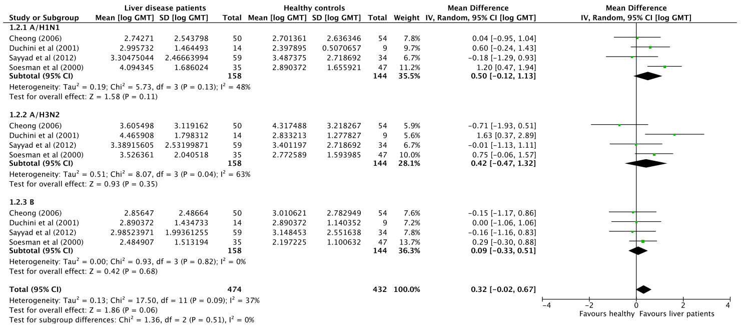

Note: Results from the non-cirrhotic and cirrhotic patient groups in Sayyad et al (2012) have been combined using the formulae recommended in Cochrane Handbook [22].

For peer review only - http://bmjopen.bmj.com/site/about/guidelines.xhtml

Figure 8. Additional analysis: Mean difference between geometric mean HI antibody titre ratio after influenza vaccination in liver disease patients compared to healthy individuals

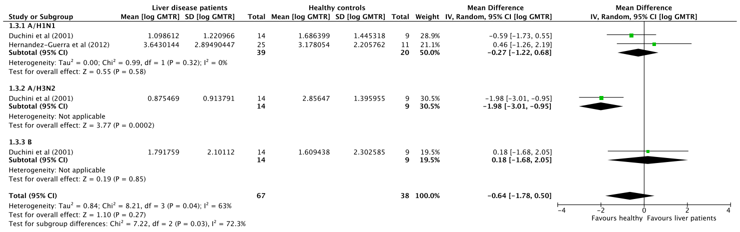

Note: Results from the non-cirrhotic and cirrhotic patient groups in Sayyad et al (2012) have been combined using the formulae recommended in Cochrane Handbook [22].

Figure 9. Additional analysis: Probability of seroconversion after influenza vaccination in liver disease patients compared to healthy individuals

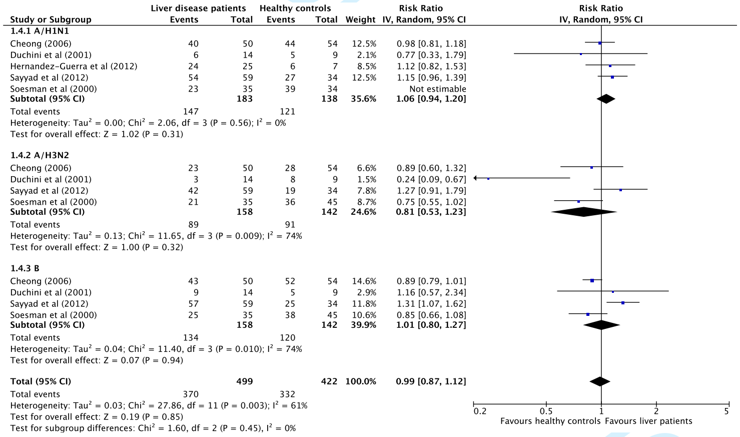

Note: Results from the non-cirrhotic and cirrhotic patient groups in Sayyad et al (2012) have been combined using the formulae recommended in Cochrane Handbook [22].

Figure 10. Additional analysis: Probability of seroprotection after influenza vaccination in liver disease patients compared to healthy individuals

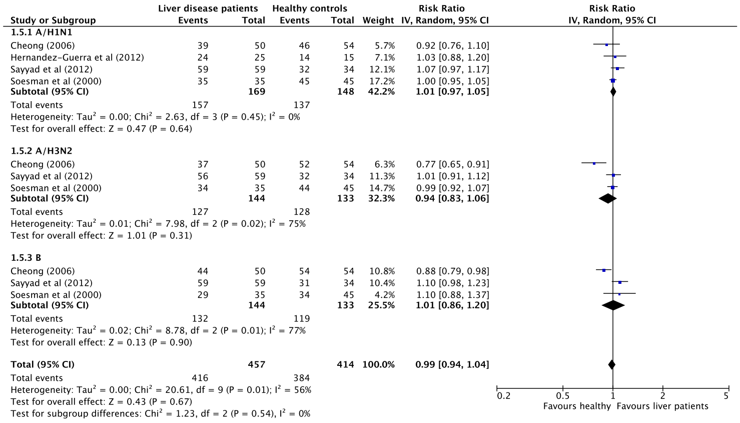

Note: Results from the non-cirrhotic and cirrhotic patient groups in Sayyad et al (2012) have been combined using the formulae recommended in Cochrane Handbook [22].

For peer review only

Figure 11. Additional analysis: Difference in seroprotection rate before vaccination in liver disease patients compared to healthy individuals

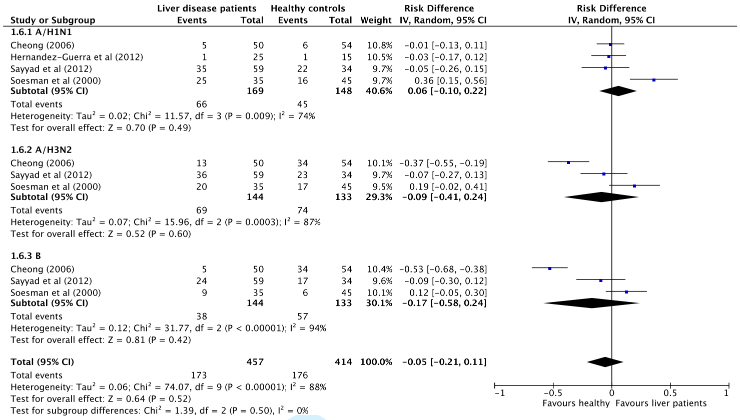

Note: Results from the non-cirrhotic and cirrhotic patient groups in Sayyad et al (2012) have been combined using the formulae recommended in Cochrane Handbook [22].

Table 10. Risk of all-cause mortality in the general population of the included studies where liver patients formed a subgroup

Study	Number of vaccinated/unvaccinated	Adjusted HR (95%CI) in the influenza season	Notes
Campitelli et al (2010)	14512/11410	0·61 (0·47–0·79)	Protective effect was present also outside the influenza season indicating presence of frailty bias.
Castilla et al (2015)	60400/42756[a]	0·84 (0·76–0·93)	Effect outside the seasons was not protective
Vila-Córcoles et al (2007	6051/5189	0·63 (0·54–0·74)	Effect outside the seasons was not protective

[a] These numbers are for the first of the two seasons studied. Number of patients and the % of vaccinated were similar for the second season.

For peer review only

References

1 Castilla J, Morán J, Martínez-Artola V, *et al*. Effectiveness of the monovalent influenza A(H1N1) 2009 vaccine in Navarre, Spain, 2009–2010: Cohort and case-control study. *Vaccine* 2011;**29**:5919–24. doi:10.1016/j.vaccine.2011.06.063

2 Cheng AC, Brown SG, Waterer GW, *et al*. Influenza epIdemIology, vaccIne coverage and vaccIne effectIveness in sentInel AustralIan hospItals in 2012: the Influenza ComplIcatIons Alert Network (FluCAN). *Commun Dis Intell* 2013;**37**:E246-252.

3 Cheng AC, Dwyer DE, Holmes M, *et al*. Influenza epIdemIology, vaccIne coverage and vaccIne effectIveness in sentInel AustralIan hospItals in 2013: the Influenza ComplIcatIons Alert Network. *Commun Dis Intell* 2014;**38**:E143-149.

4 Choi WS, Noh Y, Seo Y bin, *et al*. Case-Control Study of the Effectiveness of the 2010-2011 Seasonal Influenza Vaccine for Prevention of Laboratory-Confirmed Influenza Virus Infection in the Korean Adult Population. *Clin Vaccine Immunol* 2013;**20**:877–81. doi:10.1128/CVI.00009-13

5 Choi WS, Noh JY, Baek JH, *et al*. Suboptimal Effectiveness of the 2011 – 2012 Seasonal Influenza Vaccine in Adult Korean Populations. *PLoS One* 2015;**10**:e0098716. doi:10.1371/journal.pone.0098716

6 Castilla J, Martinez-Artola V, Salcedo E, *et al*. Vaccine effectiveness in preventing influenza hospitalizations in Navarre, Spain, 2010-2011: Cohort and case-control study. Vaccine. 2012;**30**:195–200.

7 Castilla J, Godoy P, Dominguez A, *et al*. Influenza vaccine effectiveness in preventing outpatient, inpatient, and severe cases of laboratory-confirmed influenza. Clin. Infect. Dis. 2013;**57**:167–75. doi:10.1093/cid/cit194

8 DiazGranados CA, Dunning AJ, Jordanov E, *et al*. High-dose trivalent influenza vaccine compared to standard dose vaccine in elderly adults: Safety, immunogenicity and relative efficacy during the 2009-2010 season. *Vaccine* 2013;**31**:861–6. doi:http://dx.doi.org/10.1016/j.vaccine.2012.12.013

9 Emborg H-D, Krause TG, Hviid A, *et al*. Effectiveness of vaccine against pandemic influenza A/H1N1 among people with underlying chronic diseases: Cohort study, Denmark, 2009-10. BMJ. 2012;**344**:d7901. doi:https://doi.org/10.1136/bmj.d7901

10 Mangtani P, Cumberland P, Hodgson CR, *et al*. A cohort study of the effectiveness of influenza vaccine in older people, performed using the United Kingdom general practice research database. J. Infect. Dis. 2004;**190**:1–10.

11 Olafsdottir TA, Alexandersson KF, Sveinbjornsson G, *et al*. Age and Influenza-Specific Pre-Vaccination Antibodies Strongly Affect Influenza Vaccine Responses in the Icelandic Population whereas Disease and Medication Have Small Effects. *Front Immunol* 2017;**8**:1872. doi:10.3389/fimmu.2017.01872

12 Simpson C, Lone N, Kavanagh K, *et al*. Seasonal Influenza Vaccine Effectiveness (SIVE): an observational retrospective cohort study - exploitation of a unique community-based national-linked database to determine the effectiveness of the seasonal trivalent influenza vaccine (Structured abstrac. *Heal Technol Assess Database* 2013;**1**.

13 Wang CS, Wang ST, Lai Lee CT, *et al*. Reducing major cause-specific hospitalization rates and shortening hospital stays after influenza vaccination. *Clin Infect Dis* 2004;**39**:1604–10. doi:http://dx.doi.org/10.1086/425323

14 Wang CS, Wang ST, Lai CT, *et al*. Impact of influenza vaccination on major cause-specific mortal. Vaccine. 2007;**25**:1196–203.

15 Zivich PN, Tatham L, Lung K, *et al*. Influenza vaccination status and outcomes among influenza-associated hospitalizations in Columbus, Ohio (2012-2015). *Epidemiol Infect* 2017;**145**:3284–93. doi:10.1017/S0950268817002163

16 Cheong H-J, Song J-Y, Park J-W, *et al*. Humoral and cellular immune responses to influenza vaccine in patients with advanced cirrhosis. *Vaccine* 2006;**24**:2417–22. doi:10.1016/j.vaccine.2005.11.064

17 Duchini A, Hendry RM, Nyberg LM, *et al*. Immune response to influenza vaccine in adult liver transplant recipients. *Liver Transplant* 2001;**7**:311–3. doi:10.1053/jlts.2001.23010

18 Hernandez-Guerra M, Gonzalez-Mendez Y, de Molina P, *et al*. Immunogenicity and Acceptance of Influenza A (H1N1) Vaccine in a Cohort of Chronic Hepatitis C Patients Receiving Pegylated-Interferon Treatment. *PLoS One* 2012;**7**:e48610. doi:10.1371/journal.pone.0048610

19 Ohfuji S, Fukushima W, Tamori A, *et al*. Immunogenicity of influenza A(H1N1)pdm09 vaccine and the associated factors on lowered immune response in patients with hepatitis C. *Influenza Other Respi Viruses* 2013;**7**:456–65. doi:10.1111/j.1750-2659.2012.00424.x

20 Sayyad B, Alavian SM, Najafi F, *et al*. Efficacy of Influenza Vaccination in Patients with Cirrhosis and Inactive Carriers of Hepatitis B Virus Infection. *Iran Red Crescent Med J* 2012;**14**:623–30.

21 Soesman NMR, Rimmelzwaan GF, Nieuwkoop NJ, *et al*. Efficacy of influenza vaccination in adult liver transplant recipients. *J Med Virol* 2000;**61**:85–93. doi:https://doi.org/10.1002/(SICI)1096-

For peer review only - http://bmjopen.bmj.com/site/about/guidelines.xhtml

9071(200005)61:1<85::AID-JMV14>3.0.CO;2-H
22 The Cochrane Collaboration. Cochrane Handbook for Systematic Reviews of Interventions. Version 5.1.0 [updated March 2011]. 2011.http://handbook-5-1.cochrane.org/ (accessed 25 Jul 2018).

For peer review only

PRISMA 2009 Checklist

Section/topic	#	Checklist item	Reported on page #
**TITLE**			
Title	1	Identify the report as a systematic review, meta-analysis, or both.	p. 1
**ABSTRACT**			
Structured summary	2	Provide a structured summary including, as applicable: background; objectives; data sources; study eligibility criteria, participants, and interventions; study appraisal and synthesis methods; results; limitations; conclusions and implications of key findings; systematic review registration number.	p. 2-3
**INTRODUCTION**			
Rationale	3	Describe the rationale for the review in the context of what is already known.	p. 5-7
Objectives	4	Provide an explicit statement of questions being addressed with reference to participants, interventions, comparisons, outcomes, and study design (PICOS).	p. 6-7
**METHODS**			
Protocol and registration	5	Indicate if a review protocol exists, if and where it can be accessed (e.g., Web address), and, if available, provide registration information including registration number.	p. 7
Eligibility criteria	6	Specify study characteristics (e.g., PICOS, length of follow-up) and report characteristics (e.g., years considered, language, publication status) used as criteria for eligibility, giving rationale.	p. 7-8
Information sources	7	Describe all information sources (e.g., databases with dates of coverage, contact with study authors to identify additional studies) in the search and date last searched.	p. 8-9
Search	8	Present full electronic search strategy for at least one database, including any limits used, such that it could be repeated.	p. 8-9, Supplementary materials p.4-6 (Tables 3 and 4)
Study selection	9	State the process for selecting studies (i.e., screening, eligibility, included in systematic review, and, if applicable, included in the meta-analysis).	p. 9
Data collection process	10	Describe method of data extraction from reports (e.g., piloted forms, independently, in duplicate) and any processes for obtaining and confirming data from investigators.	p. 9-10
Data items	11	List and define all variables for which data were sought (e.g., PICOS, funding sources) and any assumptions and simplifications made.	p. 9-10
Risk of bias in individual studies	12	Describe methods used for assessing risk of bias of individual studies (including specification of whether this was done at the study or outcome level), and how this information is to be used in any data synthesis.	p. 10

For peer review only - http://bmjopen.bmj.com/site/about/guidelines.xhtml

PRISMA 2009 Checklist

Summary measures	13	State the principal summary measures (e.g., risk ratio, difference in means).	p. 11-12
Synthesis of results	14	Describe the methods of handling data and combining results of studies, if done, including measures of consistency (e.g., I^2) for each meta-analysis.	p. 12-12

Section/topic	#	Checklist item	Reported on page #
Risk of bias across studies	15	Specify any assessment of risk of bias that may affect the cumulative evidence (e.g., publication bias, selective reporting within studies).	p. 12-13
Additional analyses	16	Describe methods of additional analyses (e.g., sensitivity or subgroup analyses, meta-regression), if done, indicating which were pre-specified.	p. 12
**RESULTS**			
Study selection	17	Give numbers of studies screened, assessed for eligibility, and included in the review, with reasons for exclusions at each stage, ideally with a flow diagram.	p. 13-14 (Figure 1)
Study characteristics	18	For each study, present characteristics for which data were extracted (e.g., study size, PICOS, follow-up period) and provide the citations.	p. 14-21 (Tables 1 and 2)
Risk of bias within studies	19	Present data on risk of bias of each study and, if available, any outcome level assessment (see item 12).	p. 14, Supplementary materials p. 9 (Table 6)
Results of individual studies	20	For all outcomes considered (benefits or harms), present, for each study: (a) simple summary data for each intervention group (b) effect estimates and confidence intervals, ideally with a forest plot.	p. 17-21, (Figures 2-6), Supplementary materials p.16 (Figure 1)
Synthesis of results	21	Present results of each meta-analysis done, including confidence intervals and measures of consistency.	p. 17-21 (Figures 2-6), Supplementary materials p.16 (Figure 1)
Risk of bias across studies	22	Present results of any assessment of risk of bias across studies (see Item 15).	p. 14, Supplementary materials p.10-

For peer review only

PRISMA 2009 Checklist

			13 (Tables 7 and 8)
Additional analysis	23	Give results of additional analyses, if done (e.g., sensitivity or subgroup analyses, meta-regression [see Item 16]).	p. 21-22, Supplementary materials p. 16-20 (Figures 2-11)
**DISCUSSION**			
Summary of evidence	24	Summarize the main findings including the strength of evidence for each main outcome; consider their relevance to key groups (e.g., healthcare providers, users, and policy makers).	p. 23-24 (Table 3), Supplementary materials p.13 (Table 8)
Limitations	25	Discuss limitations at study and outcome level (e.g., risk of bias), and at review-level (e.g., incomplete retrieval of identified research, reporting bias).	p. 24-26
Conclusions	26	Provide a general interpretation of the results in the context of other evidence, and implications for future research.	p. 27
**FUNDING**			
Funding	27	Describe sources of funding for the systematic review and other support (e.g., supply of data); role of funders for the systematic review.	p. 13, 28

From: Moher D, Liberati A, Tetzlaff J, Altman DG, The PRISMA Group (2009). Preferred Reporting Items for Systematic Reviews and Meta-Analyses: The PRISMA Statement. PLoS Med 6(7): e1000097. doi:10.1371/journal.pmed1000097

For more information, visit: **www.prisma-statement.org**.

For peer review only

**MOOSE CHECKLIST *** <br>Source: https://www.editorialmanager.com/jognn/account/MOOSE.pdf *Modified from Stroup DF, Berlin JA, Morton SC, Olkin I, Williamson GD, Rennie D, et al. Meta-analysis of observational studies in epidemiology: a proposal for reporting. Meta-analysis Of Observational Studies in Epidemiology (MOOSE) group. JAMA 2000;283:2008–12. Copyrighted © 2000, American Medical Association. All rights reserved.		
**Title**	Identify the study as a meta-analysis (or systematic review)	p. 1
**Abstract**	Use the journal's structured format	p. 2-3
**Introduction**	**Present:**	
	The clinical problem	p. 5-7
	The hypothesis	p. 6-7
	A statement of objectives that includes the study population, the condition of interest, the exposure or intervention, and the outcome(s) considered	p. 6-7
**Sources**	**Describe:**	
	Qualifications of searchers (eg, librarians and investigators)	p. 28
	Search strategy, including time period included in the synthesis and keywords	p. 8-9, Supplementary materials p.4-6 (Tables 3 and 4)
	Effort to include all available studies, including contact with authors	p. 9-10
	Databases and registries searched	p. 8-9
	Search software used, name and version, including special features used (eg, explosion)	p. 8-9, Supplementary materials p.4-6 (Tables 3 and 4)
	Use of hand searching (eg, reference lists of obtained articles)	p. 9
	List of citations located and those excluded, including justification	p. 13-14 (Figure 1, Tables 1 and 2), Supplementary

For peer review only

		materials p. 2, 7-8 (Table 2 and 5)
	Method of addressing articles published in languages other than English	p. 8
	Method of handling abstracts and unpublished studies	p. 9
	Description of any contact with authors	p. 9-10
**Study selection**	**Describe:**	
	Types of study designs considered	p. 7-8
	Relevance or appropriateness of studies gathered for assessing the hypothesis to be tested	p. 7-8
	Rationale for the selection and coding of data (eg, sound clinical principles or convenience)	p. 7-12
	Documentation of how data were classified and coded (eg, multiple raters, blinding, and interrater reliability)	p. 9-10
	Assessment of confounding (eg, comparability of cases and controls in studies where appropriate	p. 10-11
	Assessment of study quality, including blinding of quality assessors; stratification or regression on possible predictors of study results	p. 12-13
	Assessment of heterogeneity	p. 12
	Statistical methods (eg, complete description of fixed or random effects models, justification<br>of whether the chosen models account for predictors of study results, dose-response models,<br>or cumulative meta-analysis) in sufficient detail to be replicated	p. 11-12
**Results**	**Present:**	
	A graph summarizing individual study estimates and the overall estimate	p. 17-21, (Figures 2-6), Supplementary materials p.16 (Figure 1)
	A table giving descriptive information for each included study	p. 14-21 (Tables 1 and 2)

For peer review only

	Results of sensitivity testing (eg, subgroup analysis)	p. 21-22, Supplementary materials p. 16-20 (Figures 2-11)
	Indication of statistical uncertainty of findings	p. 17-21, (Figures 2-6), Supplementary materials p. 16-20 (Figures 1-11)
**Discussion**	**Discuss:**	
	Strengths and weaknesses	p. 24-26
	Potential biases in the review process (eg, publication bias)	p. 24-26, Supplementary materials p. 2, 10-13 (Tables 1, 7 and 8)
	Justification for exclusion (eg, exclusion of non–English-language citations)	p. 26
	Assessment of quality of included studies	p. 23-24 (Table 3), Supplementary materials p.13 (Table 8)
	Consideration of alternative explanations for observed results	p. 24
	Generalization of the conclusions (ie, appropriate for the data presented and within the domain of the literature review)	p. 24-25
	Guidelines for future research	p. 27
	Disclosure of funding source	p. 13, 28

For peer review only
